# A systematic review of immunosuppressive protocols used in AAV gene therapy for monogenic disorders

**DOI:** 10.1016/j.ymthe.2024.07.016

**Published:** 2024-07-22

**Authors:** Besarte Vrellaku, Ilda Sethw Hassan, Rebecca Howitt, Christopher P. Webster, Eli Harriss, Fraser McBlane, Corinne Betts, Jorge Schettini, Mattia Lion, John E. Mindur, Michael Duerr, Pamela J. Shaw, Janine Kirby, Mimoun Azzouz, Laurent Servais

**Affiliations:** 1 Department of Paediatrics, MDUK Oxford Neuromuscular Centre & NIHR Oxford Biomedical Research Centre, University of Oxford, Oxford, UK; 2 Sheffield Institute for Translational Neuroscience, Division of Neuroscience, School of Medicine and Population Health, University of Sheffield, Sheffield, UK; 3 The Queen’s College, University of Oxford, Oxford, UK; 4 Bodleian Health Care Libraries, University of Oxford, Oxford, UK; 5 Novartis Pharma AG, Basel, Switzerland; 6 Takeda Pharmaceuticals USA, Inc, Cambridge, MA, USA; 7 Bayer Aktiengesellschaft, CGT&Rare Diseases, Leverkusen, Deutschland; 8 Gene Therapy Innovation & Manufacturing Centre (GTIMC), University of Sheffield, Sheffield, UK; 9 Division of Child Neurology, Department of Paediatrics, Centre de Référence des Maladies Neuromusculaires, University Hospital Liège and University of Liège, Liège, Belgium

**Keywords:** adeno-associated virus, AAV, gene therapy, immunosuppression protocols, immunosuppressant, immunity, monogenic disorders, clinical trials

## Abstract

The emergence of adeno-associated virus (AAV)-based gene therapy has brought hope to patients with severe monogenic disorders. However, immune responses to AAV vectors and transgene products present challenges that require effective immunosuppressive strategies. This systematic review focuses on the immunosuppressive protocols used in 38 clinical trials and 35 real-world studies, considering a range of monogenic diseases, AAV serotypes, and administration routes. The review underscores the need for a deeper understanding of immunosuppressive regimens to enhance the safety and effectiveness of AAV-based gene therapy. Characterizing the immunological responses associated with various gene therapy treatments is crucial for optimizing treatment protocols and ensuring the safety and efficacy of forthcoming gene therapy interventions. Further research and understanding of the impact of immunosuppression on disease, therapy, and route of administration will contribute to the development of more effective and safer gene therapy approaches in the future.

## Graphical abstract

**Figure:**
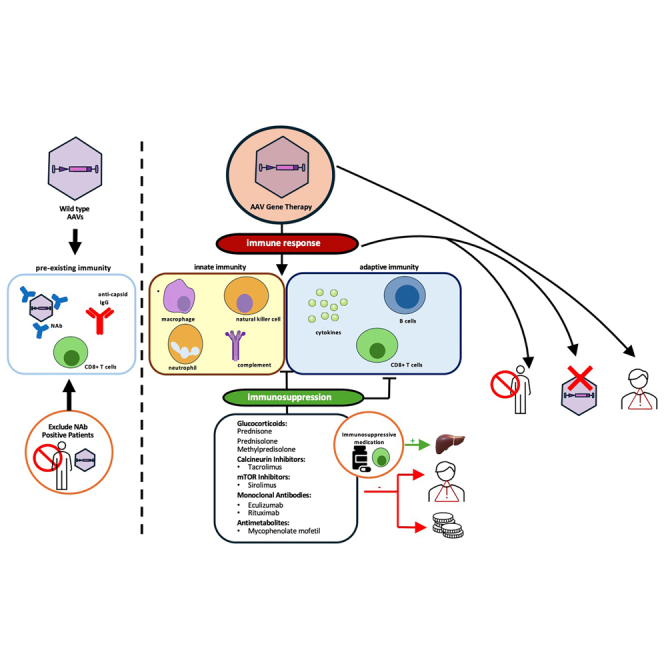

## Teaser

Through a systematic literature review, Vrellaku and colleagues emphasize the importance of minimizing immunological responses to optimize AAV gene therapy protocols. Their findings underscore the need for further research of the impact on immunosuppression on disease and therapy administration, aiming to develop safer and more effective AAV gene therapy protocols.

## Introduction

Gene therapy clinical trials using adenovirus (AdV) or adeno-associated virus (AAVs) as delivery systems have been ongoing since the late 1990s.^1^^,^^2^^,^^3^ The first phase 1 clinical trial of AdV-based gene therapy in 1996 delivered the cystic fibrosis transmembrane conductance regulator (*CFTR*) gene to patients with cystic fibrosis.^2^ However, in 1999, gene therapy research was abruptly halted due to the death of Jesse Gelsinger after treatment for ornithine-transcarbamylase (OTC) hepatic enzyme deficiency with AdV-based gene therapy.^4^ After Gelsinger received an injection of an AdV vector carrying a wild-type version of the OTC enzyme and died soon after, an immediate review was prompted which raised questions regarding the safety profile of viral vectors. It was found that blood samples from Jesse Gelsinger contained high levels of pre-existing antibodies against AdV serotype 5, and that these antibodies were able to enhance innate immune responses (in particular dendritic cells) resulting in such an extreme, and ultimately fatal, inflammatory response.^5^ This led eventually to the use of AAV rather than AdV. In 2012, alipogene tiparvovec was the first approved AAV-mediated gene therapy in Europe for treating hereditary lipoprotein lipase deficiency using an AAV1 vector.^6^ After extensive clinical development, in 2017, voretigene neparvovec (VN; AAV2-hRPE65v2) received the U.S. Food and Drug Administration (FDA) approval for pediatric patients with RPE65-associated Leber congenital amaurosis (LCA) and confirmed biallelic RPE65-mediated retinal dystrophy, which are conditions that result in progressive vision loss, making it the first-ever FDA-approved gene therapy.^7^^,^^8^^,^^9^^,^^10^ Similarly, onasemnogene abeparvovec (OA), an AAV serotype 9 (AAV9)-based gene therapy for pediatric patients with spinal muscular atrophy (SMA), was first made available in the United States in 2019 before its approved use in more than 40 countries.^11^ Rapid and early benefits of OA were demonstrated in symptomatic patients with infantile-onset SMA in the phase 3 STR1VE^12^ and STR1VE-EU^13^ trials, with evidence of sustained and durable efficacy, as well as a favorable long-term safety profile as shown in the 5-year extension of the phase 1 trial, START.^14^ Valoctocogene roxaparvovec and etranacogene dezaparvovec were approved for the treatment of hemophilia A and B in 2023 and 2022, respectively.^15^^,^^16^ Recently, the FDA approved fidancogene elaparvovec for adult patients with moderate to severe hemophilia B. In addition, delandistrogene moxeparvovec-rokl is the first gene therapy approved by the FDA for treatment of Duchenne muscular dystrophy (DMD).^17^^,^^18^

The transfer of DNA into specific cell types thus constitutes an elegant and attractive approach to target the root cause of disease in individuals who present with a severe genetic condition—mostly rare and severe monogenic disorders with haploinsufficiency. To date, the vast majority of clinical developments in gene therapy rely on AAVs, primarily for the replacement or addition of genes.^19^^,^^20^

Despite recent successful regulatory approvals, one of the challenges that remains for further successful implementation of AAV-based gene therapies is overcoming immune responses geared toward the vector or transgene. Such immune responses can lead to loss of treatment efficacy over time and can also result in severe and sometimes fatal toxicities in treated patients. Toxicities often manifest as elevated liver enzymes due to the body’s immune response against the vector components, such as the capsid or expression cassette as well as the transgene product, resulting in liver inflammation and subsequent damage to liver cells, as indicated by alanine aminotransferase (ALT) and aspartate aminotransferase (AST) release. Additionally, patients may experience thrombotic microangiopathy (TMA), including kidney injury, as well as cardiotoxicity.^21^^,^^22^ Although OA has been delivered in more than 1,800 SMA patients worldwide and demonstrates clear evidence of clinically meaningful efficacy,^12^^,^^23^ especially when administered early in young infants,^24^ several adverse events (AEs) related to the host immune response and complement activation elicited by AAV capsid proteins have been reported.^21^^,^^25^^,^^26^^,^^27^ A study reported the first fatal TMA case following administration of OA in a 6-month-old child with SMA type 1^25^ who was a carrier of a potential genetic predisposition in the complement factor I gene. The finding of severe TMA is likely due to complement recognition of the AAV capsid following OA therapy, demonstrating the broad impact of systemic AAV on immune activation and the need for dosing protocols to add immunosuppression to avoid these AEs.

Recent strategies to evade detrimental immune responses to AAV exposure include the engineering of AAV by replacing the viral genome with a therapeutic expression cassette containing inverted terminal repeats, promoters and enhancers, and a codon-optimized gene of interest, while also modifying capsid sequences to yield a recombinant AAV (rAAV) that decreases immunogenicity and prolongs transduction in host cells.^28^ However, the challenge of overcoming host immunity to better enable gene therapy remains. While AAV infection is considered non-pathogenic in humans, initial exposure to AAV induces cellular and humoral immune responses against rAAV due to capsid similarity.^29^^,^^30^ Although AAV has been known to be a non-pathogenic virus, recent reports of cases of acute severe hepatitis in children have challenged the idea that AAV is a harmless virus.^31^ More specifically, three independent studies published in March 2023 demonstrated that infection with AAV2 was linked to recent clusters of unexplained acute severe hepatitis in children.^31^^,^^32^^,^^33^^,^^34^

Roughly 30%–70% of the general population have pre-existing neutralizing antibodies (NAbs) against various serotypes, including AAV1, AAV2, AAV5, AAV6, AAV8, and AAV9, depending on the geographical location, health status, and assay type, among other factors across studies.^34^^,^^35^^,^^36^ Immune responses against AAV have also been observed across different monogenic disorders, causing the various adverse reactions described above. In addition, immune responses can sometimes hinder therapeutic effects by eliciting antibodies against AAV capsids, which reduces the expression of the transgene product. This reduction in expression can also occur if transduced cells are killed by cytotoxic T cells or if there are immune responses against the transgene product itself. Humoral immune responses, measured by different assays as either NAbs and total antibodies (TAbs), can create an immune response barrier to successful AAV transduction.^37^^,^^38^ NAbs can neutralize capsids, either by interfering with intracellular processes that lead to capsid uncoating or by blocking key epitopes needed for receptor-mediated uptake into target cells, thereby decreasing the efficacy of gene transfer therapy.^39^^,^^40^^,^^41^ Opsonization is another mechanism by which anti-AAV TAbs may impact AAV gene therapy treatment efficacy.^42^ Furthermore, T cell responses may eliminate transgene-expressing cells, resulting in hepatotoxicity and loss of transgene expression, as seen in several clinical trials.^38^^,^^43^^,^^44^ As such, the host immune response is an important factor to monitor and temper after gene therapy as it may relate to both the treatment’s safety and efficacy.

Extensive efforts to suppress immune responses to AAV have been undertaken across trials. Corticosteroids such as methylprednisolone, prednisone, and prednisolone are widely used in immunosuppressive protocols for inhibiting immune responses to AAV by the decrease of proinflammatory cytokines/chemokines and attenuating liver toxicity.^8^^,^^11^^,^^45^ Early trials of AAV gene therapy used a reactive approach for administering corticosteroids in response to instances of elevated liver enzymes, which were thought, in certain cases, to be linked to an AAV capsid-specific cytotoxic T cell response indicative of liver injury.^43^^,^^44^ Corticosteroid treatment typically resolves the elevation of liver transaminases.^12^^,^^43^ Subsequent clinical trials incorporated prophylactic immunosuppression regimens that included one or a combination of pharmacotherapies. Corticosteroids bind to glucocorticoid receptors and modify transcriptional signaling that results in global anti-inflammatory and immunosuppressive effects.^46^ Corticosteroids exert these effects through multiple mechanisms including downregulation of Toll-like receptor expression, suppression of proinflammatory cytokines, and upregulation of anti-inflammatory cytokines.^47^

Other immunosuppressants used in AAV gene therapies include sirolimus, mycophenolate mofetil, calcineurin inhibitors and rituximab. Mycophenolate mofetil inhibits T and B cell proliferation by targeting type II inosine monophosphate dehydrogenase, thus suppressing both cell-mediated and humoral immune responses.^15^^,^^45^^,^^48^^,^^49^^,^^50^ Sirolimus is also used in AAV clinical trials for immune suppression (e.g., NCT02240407) and works by inhibiting T and B cell activation and induces regulatory T cells (Tregs) through mammalian target of rapamycin (mTOR) targeting.^50^

Calcineurin inhibitors such as tacrolimus exert their immunosuppressive effects by suppressing the production of proinflammatory cytokines such as IL-2, thereby inhibiting T cell activation and proliferation and inhibition of T helper cell-dependent B cell response.^51^^,^^52^^,^^53^ AAV clinical trials also use a combination of immunosuppressant therapies, either administered before or at the time of AAV dosing, and these are continued after AAV dosing. The combination of rituximab, sirolimus, and corticosteroids was used in a recent clinical trial for GM2-gangliosidosis through CNS-directed AAV delivery^3^ with no vector-related AEs observed. Moreover, patients received glucocorticoids with or without tacrolimus to decrease the risk of vector-related immune responses.^48^

While the use of immunosuppressive regimens affords some level of control over immune responses to AAV gene therapy, these protocols also come with an increased risk of infection or viral reactivation, especially in patients who may already be in a severe clinical condition. This can question the safety and/or benefit ratio of immunosuppression—for instance, when low doses of AAV are injected locally or in a well-delineated space. Despite the importance of an adequate immunosuppressive regimen, there is no consensus or specific guidelines on which regimen is the most appropriate in terms of risk versus benefit ratio, either generally or more specifically in regard to AAV dose, serotype, route of administration, and any pre-existing or underlying condition(s). To our knowledge, no formal or informal comparison of the different immunosuppressive regimens has been conducted.

The aim of this review was to systematically catalog the various immunosuppressive protocols used across various AAV gene therapy trials, and to map these according to monogenic disease, gene therapy treatment (including vector serotype), and route of administration. Additionally, clinical trials will be mapped according to serious adverse reactions, including biological biomarkers (e.g., AST, ALT, thrombocytopenia, and lactate dehydrogenase). We also describe methods for treating immune responses and evaluate the efficacy of these protocols.

## Results

### Study characteristics

**Figure 1:**
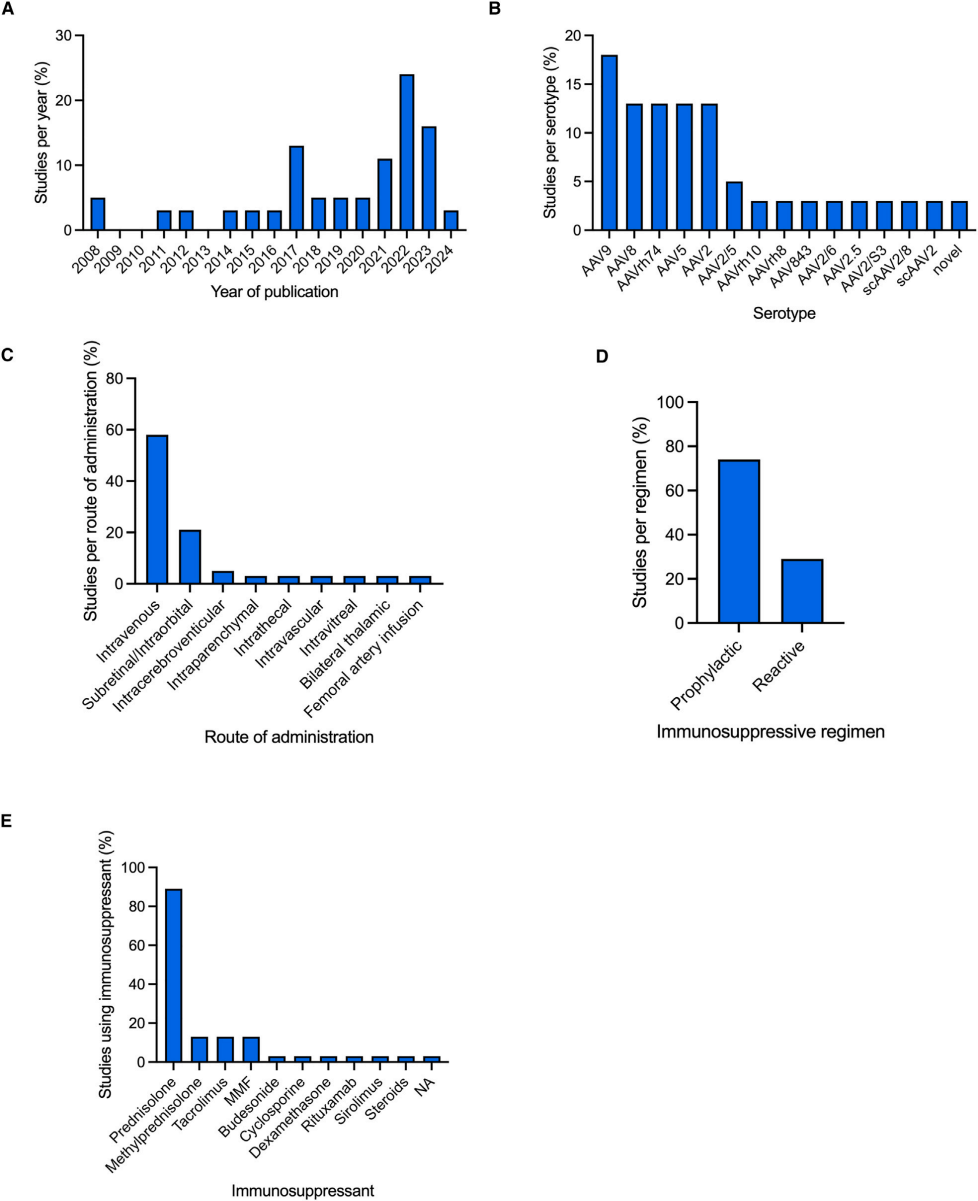
Number of studies identified during the systematic review (A–E) Year of study, (B) serotype, (C) route of administration, (D) immunosuppressive regimen, and (E) immunosuppressant used.

Table 1 depicts a summary of the 38 AAV clinical trials assessed in this review. The included studies were mainly conducted in the United States (*n* = 28), the UK (*n* = 8), Germany (*n* = 6), Australia (*n* = 5), and France (*n* = 5), with 15 other countries also contributing to these clinical trials (Table 1). The 38 studies were conducted between 2008 and 2024, but more than 50% of these studies were conducted between 2021 and 2023 (Figure 1A). Although more than 15 different diseases were included in these clinical trials, the most common were hemophilia B (*n* = 8), SMA (*n* = 7), LCA (*n* = 5), DMD (*n* = 5), and hemophilia A (*n* = 3).

**Table 1:** Overview of AAV gene therapy clinical trials

No.	Disease	AAV gene therapy	No. of participants and age ranges	Route	Highest dose	Vector type	Immunosuppressive protocol	Prophylactic or reactive regimen	Clinical evidence of immunosuppression effectiveness	NCT (phase)	Reference	Country
1	hemophilia A	valoctocogene roxaparvovec	*n* = 134; age range 18–70 years	intravenous	6 × 10^13^ vg/kg	AAV5	prednisone or prednisolone tapering dose of 60 mg per day for 8 weeks other immunosuppressants were used by 39 participants (29.1%) because of contraindications, side effects, or a poor or no response to glucocorticoid treatment (budesonide, tacrolimus, mycophenolate, methylprednisolone)	reactive	reduced ALT levels	NCT03370913 (3)	Mahlangu et al., 2023, Ozelo et al., 2022^15^^,^^137^	USA Australia Belgium Brazil France Germany Israel Italy Republic of Korea South Africa Spain Taiwan UK
2	hemophilia A	valoctocogene roxaparvovec	*n* = 9 age >18 years	intravenous	6 × 10^13^ vg/kg	AAV5	prednisolone, tapering dose of 60 mg/kg for 2 weeks, then down to 5 mg/day for 1 week	prophylactic	reduced ALT levels	NCT02576795 (1/2)	Rangarajan et al., 2017, Long et al., 2021, Fong et al., 2022, Pasi et al., 2021^59^^,^^67^^,^^81^^,^^85^	UK
3	hemophilia A	giroctocogene fitelparvovec	*n* = 11 (≥18 years)	intravenous	3 × 10^13^ vg/kg	AAV2/6	prednisone, 60 mg with dose tapering to 30 mg, 15 mg, and 5 mg/d	reactive	reduced ALT levels	NCT03061201 (1/2)	Leavitt et al., 2024^104^	USA
4	hemophilia B	BBM-H901	*n* = 10 age >18 years	intravenous	5 × 10^12^ vg/kg	dsAAV843	prednisone, 1 mg/kg	prophylactic	reduced proportion of cytotoxic T cells downregulated percentage of CD16^+^ monocytes and dendritic cells	NCT04135300 (1)	Xue et al., 2022^63^	China
5	hemophilia B	etranacogene dezaparvovec	*n* = 54 age >18 years	intravenous	2.10 × 10^13^ vg/kg	AAV5	prednisolone or methylprednisolone or prednisone, starting dose 60 mg with tapering until 5 mg/week	reactive	N/A	NCT03569891 (3)	Pipe et al., 2023^16^	USA Belgium Denmark Germany Ireland The Netherlands Sweden UK
6	hemophilia B	verbrinacogene setparvovec	*n* = 10 age >18 years	intravenous	1.28 × 10^12^ vg/kg	AAV2/S3	prednisolone with or without tacrolimus, methylprednisolone at varying doses	prophylactic and reactive	N/A	NCT03369444 and NCT03641703 (1/2)	Chowdary et al., 2022^48^	USA Ireland Italy UK
7	hemophilia B	AMT-060	*n* = 10 age >18 years	intravenous	2 × 10^13^ gc/kg	AAV5	prednisolone	reactive	reduced ALT levels	NCT02396342 (1/2)	Majowicz et al., 2019, Miesbach et al., 2018^68^^;^^86^	Denmark Germany The Netherlands
8	hemophilia B	SPK-9001	*n* = 15 ≥ 18 years	intravenous	2 × 10^12^ vg/kg	a novel, bioengineered single-stranded adeno-associated viral vector carrying human FIX variant	prednisolone, 60 mg	reactive	reduced ALT levels	NCT02484092 (1/2)	George et al., 2017^105^	USA Australia
9	hemophilia B	BAX 335 (scAAV8.FIXR338)	*n* = 30 20 to 69 years	intravenous	3 × 10^12^ vg/kg	AAV8	prednisone, 2.6–60 mg with dose tapering	reactive	although corticosteroid therapy was associated with immediate normalization of the IFN-γ ELISpot in participant 6, this signal remained elevated for weeks after the initiation of prednisone in participant 7 systemic corticosteroid administration initiated in response to ALT elevations in participants 6 and 7, and as prophylaxis in participant 8, did not stabilize FIX activity levels in these participants	NCT01687608 (1/2)	Konkle et al., 2021^82^	USA
10	hemophilia B	scAAV2/8-LP1-hFIXco	*n* = 14 ≥ 18 years	intravenous	2 × 10^12^ vg/kg	scAAV2/8	prednisolone, 60 mg with dose tapering	reactive	reduced ALT levels	NCT00979238 (1/2)	Nathwani et al., 2011, Nathwani et al., 2014^43^^,^^44^	USA UK
11	hemophilia B	etranacogene dezaparvovec (ATM-061)	*n* = 3 (ages 43, 47, and 50 years)	intravenous	2 × 10^13^ gc/kg	AAV5	prednisone, 50 mg for 5 days starting at day 94 for bronchitis treatment in 1 patient	reactive	N/A	NCT03489291 (2b)	Von Drygalski et al., 2019^106^	USA
12	DMD	rAAV2.5-CMV-minidystrophin (d3990)	*n* = 6; 5–11 years	intravenous	3 × 10^12^ vg	AAV2.5	methylprednisolone, 2 mg/kg but limited to <1 g in total	prophylactic	IFN-γ levels	NCT00428935(1)	Bowles et al., 2012^102^	USA
13	DMD	delandistrogene moxeparvovec	*n* = 4; 4 to 7 years	intravenous	2 × 10^14^ vg/kg	rAAVrh74	prednisone, 1 mg/kg with tapering over 30 days	prophylactic	liver enzyme elevation returned to normal after corticosteroids	NCT03375164 (1 and 2)	Mendell et al., 2020^66^	USA
14	DMD	delandistrogene moxeparvovec	*n* = 41; age range 4–8 years	intravenous	2 × 10^14^ vg/kg	rAAVrh74	prednisone or prednisolone, 1 mg/kg daily	prophylactic	N/A	NCT03769116 (2)	Mendell et al., 2023^57^	USA
15	DMD	delandistrogene moxeparvovec	*n* = 20; age range ≥4 to <8 years	intravenous	1.33 × 10^14^ vg/kg	rAAVrh74	prednisone or prednisolone, 1 mg/kg daily	prophylactic	N/A	NCT04626674 (1)	Zaidman et al., 2023^103^	USA
16	SMA	OA	*n* = 76; mean age 16.8	intravenous	1 × 10^14^ vg/kg	AAV9	prednisolone, 1 mg/kg	prophylactic	N/A	N/A (observational study)	Weiβ et al., 2022^83^	Germany Austria
17	SMA	OA	*n* = 15; median age 32 days (range, 9–43)	intravenous	1 × 10^14^ vg/kg	AAV9	prednisolone, initially 1 mg/kg/day, then increased to 2 mg/kg/day following protocol amendments	prophylactic	no SAEs related to gene therapy product	NCT03505099 (3)	Servais et al., 2023^22^	USA Australia Belgium Canada Japan UK
18	SMA	OA	*n* = 21; age range, 0.5–24 months	intravenous	1 × 10^14^ vg/kg	AAV9	prednisolone, 1 mg/kg/day	prophylactic	hypertension	N/A (cohort study)	D'Silva et al., 2022^109^	Australia
19	SMA	OA	*n* = 15 up to 6 months	intravenous	2.4 × 10^14^ vg	AAV9	prednisolone, 1 mg/kg/day	prophylactic	reduced ALT and AST levels	NCT02122952 (1)	Mendell et al., 2017^11^	USA
20	SMA	OA	*n* = 8 age range 10–37 months	intravenous	1.1 × 10^14^ vg	AAV9	prednisolone, 1 mg/kg/day	prophylactic	increased transaminases typically responded to steroid treatment	N/A (retrospective analysis)	Friese et al., 2021^65^	Germany
21	SMA	OA	*n* = 22 up to 180 days	intravenous	1.1 × 10^14^ vg	AAV9	prednisolone, 1 mg/kg/day	prophylactic	prevented elevation of ALT levels	NCT03306277 (3)	Day et al., 2021, Mercuri et al., 2021^12^^,^^13^	USA
22	X-linked myotubular myopathy	resamirigene bilparvovec	*n* = 26; age range, 10.0–64.7 months	intravenous	3.5 × 10^14^ vg/kg	AAV8	prednisolone (1 mg/kg) daily	prophylactic	N/A	NCT03199469 (2/3)	Shieh et al., 2023^56^	USA Canada France Germany
23	LCA2	AAV2-hRPE65v2	*n* = 12; ≥18 years	subretinal/intraorbital	1.5 × 10^11^ vg	AAV2	prednisone, 1 mg/kg/day for 10 days, followed by 0.5 mg/kg/day for 7 days	prophylactic	reduced NAb levels	NCT00516477 (1)	Maguire et al., 2008, Simonelli et al., 2010^7^,^10^	USA
24	LCA2	rAAV2-CBSB-hRPE65	*n* = 3; ages 21, 23, and 24 years	subretinal/intraorbital	5.96 × 10^10^ vg	AAV2	steroids	reactive	N/A	NCT00481546 (1)	Hauswirth et al., 2008^69^	USA
25	LHON	scAAV2-P1ND4v2	*n* = 28; 16–56 years	intravitreal	1 × 10^10^ vg/eye	scAAV2	prednisolone	reactive	management of uveitis	NCT02161380 (1)	Lam et al., 2022^107^	USA
26	age-related macular degeneration	rAAV.sFLT-1	*n* = 40; ≥55 years	subretinal/intraorbital	1 × 10^11^	AAV2	prednisolone	prophylactic	N/A	NCT01494805 (1)	Rakoczy et al., 2015^84^	Australia
27	RPE65-deficient LCA and severe early childhood-onset retinal dystrophy	rAAV2-CB-hRPE65	*n* = 12; 6 to 39 years	subretinal/intraorbital	6 × 10^11^ vg	AAV2	topical corticosteroids	prophylactic	postoperative treatment with topical corticosteroids and antibiotics no enzyme-linked immunospot response to transgene or capsid no vector DNA in the blood	NCT00749957 (1/2)	Weleber et al., 2016^108^	USA
28	RPE65-mediated inherited retinal dystrophy (LCA)	AAV8-coRPGR codon optimized RPGR	*n* = 18; ≥18 years	subretinal/intraorbital	5 × 10^12^ vp/mL	AAV8	prednisolone; 1 mg/kg at start of GT; 60–30 mg/day upon acute inflammation	prophylactic	the subretinal inflammation seems to have resolved in all cases by 6 months after a course of oral corticosteroids. The inflammation seemed to have resolved in all cases by 6 months when all patients had creased oral corticosteroid treatment no patient required secondary immunosuppressive therapy	NCT03116113 (1/2)	Cehajic-Kapetanovic et al., 2020^60^	USA UK
29	RPE65-mediated inherited retinal dystrophy (LCA)	VN (AAV2-hRPE65v2)	*n* = 31; 4–44 years	subretinal/intraorbital	1.5 × 10^11^ vg	AAV2	prednisone; 1 mg/kg/day, maximum dose 40 mg/day and tapered until 3 days before injection of the second eye when the steroid regimen was repeated	prophylactic	N/A	NCT00999609 (3)	Russell et al., 2017^8^	USA
30	X-linked retinitis pigmentosa	AAV8-RS1	*n* = 11; 23–72 years	subretinal/intraorbital	3 × 10^11^ vg	AAV8	cyclosporine: 175 mg twice daily; mycophenolate mofetil at 500 and 1,000 mg twice daily; prednisone at 60 mg	prophylactic	not efficacious	NCT02317887 (1/2)	Mishra et al., 2021^71^	USA
31	X-linked retinoschisis	cotoretigene toliparvovec	*n* = 18; 20.7–50.7 years	subretinal/intraorbital	5 × 10^11^ vg	AAV8	preoperative treatment: 1 mg/kg/day of prednisolone (beginning 2 days before gene therapy, on the day of surgery and for 7 days afterward) followed by 0.5 mg/kg/day for 7 days, 0.25 mg/kg/day for 2 days, and 0.125 mg/kg/day for 2 days postoperative (additional) treatment: prednisolone: 60 mg daily with tapering; dexamethasone: 0.1% or 0.7 mg	prophylactic	1 case of reduced visual acuity resolved with corticosteroids but 1 case did not as there had been loss of central photoreceptors	NCT03116113 (1)	von Krusenstiern et al., 2023^110^	USA UK
32	DMD	rAAVrh74.MCK.GALGT2	*n* = 2; 6.9 and 8.9 years	isolated limb infusion: injection into the femoral artery of both legs	1 × 10^14^ vg/kg	rAAVrh74	prednisone, 1 mg/kg/day	prophylactic	N/A	NCT03333590 (1/2)	Flanigan et al., 2022^64^	USA
33	LHON	scAAV2-P1ND4v2	*n* = 28; 16–56 years	intravitreal	1 × 10^10^ vg/eye	scAAV2	prednisolone	reactive	management of uveitis	NCT02161380 (1)	Lam et al., 2022^107^	USA
34	limb-girdle muscular dystrophy	scAAVrh74.tMCK.hSGCA	*n* = 6; 8–13 years	intravascular	3 × 10^12^ vg/kg	AAVrh74	prednisone, 1 mg/kg/day	prophylactic	N/A	N/A (1/2)	Mendell et al., 2019^80^	USA
35	MPS type IIIA	AAVrh.10-MPS3A	*n* = 4 (patients 1–3, aged between 5.5 and 6 years; patient 4 aged 2 years 8 months	intracerebroventicular	7.2 × 10^11^ vg	AAVrh.10	tacrolimus - 0.2 mg/kg/day mycophenolate mofetil, 1,200 mg/m^2^ initially, adapted to obtain AUC_0–12h_ > 30 mg g/L at 7 days post-treatment prednisolone - 1 mg/kg/day	prophylactic	N/A	NCT01474343 (1/2)	Tardieu et al., 2014, Tardieu et al., 2017^45^^;^^58^	France
36	MPS type IIIB	rAAV2/5-hNaGlu	*n* = 4; 20, 26, 30, and 53 months	intracerebroventicular	4 × 10^12^ vg	AAV2/5	tacrolimus - 0.2 mg/kg/day mycophenolate mofetil, 1,200 mg/m^2^/day prednisolone - 1 mg/kg/day	prophylactic	N/A	EudraCT, number 2012-000856-33, and the International Standard Clinical Trial Registry, number ISRCTN19853672 (1/2)	Tardieu et al., 2017^58^	France
37	MPS type IIIB	rAAV2/5-hNAGLU	*n* = 4; 18–60 months	intraparenchymal	4 × 10^12^ vg	AAV2/5	tacrolimus - 0.2 mg/kg/day mycophenolate mofetil, 1,200 mg/m^2^/day	prophylactic	N/A	NCT03300453 (1/2)	Gougeon et al., 2021, Deiva et al., 2021^49^^,^^50^	France
38	RPE65-Deficient LCA and severe early childhood-onset retinal dystrophy	rAAV2-CB-hRPE65	*n* = 12; 6–39 years	subretinal/intraorbital	6 × 10^11^ vg	AAV2	Topical corticosteroids	prophylactic	postoperative treatment with topical corticosteroids and antibiotics no enzyme-linked immunospot response to transgene or capsid no vector DNA in the blood	NCT00749957 (1/2)	Weleber et al., 2016^108^	USA
39	RPE65-mediated inherited retinal dystrophy (LCA)	VN (AAV2-hRPE65v2)	*n* = 31; 4 to 44 years	subretinal/intraorbital	1.5 × 10^11^ vg	AAV2	prednisone; 1 mg/kg/day, maximum dose 40 mg/day and tapered until 3 days before injection of the second eye when the steroid regimen was repeated	prophylactic	N/A	NCT00999609 (3)	Russell et al., 2017^8^	USA
40	SMA	OA	*n* = 32; 7–55 months)	intrathecal	2.4 × 10^14^ vg	AAV9	prednisolone, 1 mg/kg/day	prophylactic	N/A	NCT03381729 (1)	Finkel et al., 2023^70^	USA
41	Tay-Sachs disease	AAVrh8-HEXA and AAVrh8-HEXB	*n* = 2; 7 and 30 months	bilateral thalamic injection	4.2 × 10^13^ vg	AAVrh8	patients received a regimen that included rituximab (375 mg m^−2^), intravenous infusion of methylprednisolone (10 mg kg−^1^) and sirolimus (1 mg m^−2^). prednisone (2 mg kg^−1^ per day) was administered for 90 days, and sirolimus was maintained at 3–7 ng mL^−^1 for 180 days, both followed by a 1-month taper.	prophylactic	B cell levels decreased to <1% of total lymphocytes intravenous immunoglobulin was given as needed to maintain serum levels between 700 and 1,000 mg dL^−1^ a single dose of rituximab resulted in a reduction in B cell counts for >6 months in each patient	N/A (expanded-access clinical trial)	Flotte et al., 2022^3^	USA

AUC, area under the curve; FIX, factor IX; GTMP, no SAEs related to gene therapy product; LHON, Leber hereditary optic neuropathy; N/A. not appliable.

### Types of AAV and routes of administration

A total of 15 different AAV serotypes were used, with the most common being AAV9 (18%; *n* = 7) (Figure 1B). In 58% of cases, these AAV vectors were administered via intravenous injection or infusion (*n* = 22) (Figure 1C). However, the route of administration was somewhat dependent on the disease being treated and, therefore, the target tissue. For example, in clinical trials for ocular conditions, including LCA, Leber hereditary optic neuropathy, macular degeneration, severe early childhood onset retinal dystrophy, X-linked retinitis pigmentosa, and X-linked retinoschisis, the gene therapy vectors were administered directly into the eye via intravitreal or subretinal injection, whereas all studies relating to hemophilia A and B used intravenous delivery as the preferred route of administration. Given that these 38 studies cover more than 15 different diseases and 15 different AAV serotypes, the doses of each clinical vector are varied and depend on the disease and vector type. The highest single dose in each trial is listed in Table 1.

### Prophylactic immunosuppression

#### Clinical studies

When considering the immunosuppressive treatment used in relation to these AAV gene therapies, in 74% of studies, patients received prophylactic immunosuppression (*n* = 28) either before (up to 2 weeks in advance, though more commonly 24–72 h beforehand), on the day of, or immediately after AAV gene therapy administration (Figure 1D). In the remaining 26% of cases (*n* = 10), immunosuppressive treatments were used in response to a range of AEs and serious AEs (SAEs) associated with the gene therapy product (Table 1). A total of 8 different immunosuppressants are detailed in the 38 identified studies (Figure 1D). The most common immunosuppressants administered were corticosteroids, either prednisolone, its prodrug prednisone, or methylprednisolone. Corticosteroids were used either on their own, or in conjunction with other immunosuppressants, in 95% (36/38) of the identified studies. Other immunosuppressants listed in more than one of the identified studies were tacrolimus (5/34 studies) and mycophenolate mofetil (5/34 studies). Cyclosporine was mentioned in a single study only, as was a combination of sirolimus and rituximab (Figure 1E). Where specified, the dose of prednisolone ranged from 1 to 2 mg/kg, with a total daily dose in the range of 30–60 mg. In nearly all cases, prednisolone was administered in a tapering dose over a period of days and weeks, with stated durations ranging from 7 to 133 days. Nine studies (26%) had to increase the dose or introduce additional immunosuppressive agents beyond the initial protocol in response to immunogenicity that occurred in individual patients.

#### Real-world evidence

Table 2 depicts a summary of the 35 real-world studies in this review with the most common studies being SMA (*n* = 31), inherited retinal dystrophy (*n* = 3), and hemophilia (*n* = 1). These real-world outcomes support findings from the interventional trial program and demonstrate the effectiveness of OA in a large patient population, which was consistent with initial clinical data and published 5-year follow-up data. Observed AEs were consistent with the established safety profile of OA. Common AEs included pyrexia, vomiting, thrombocytopenia, and elevated liver enzymes. AEs related to OA were frequent and sometimes serious. Although most AEs were satisfactorily managed in clinical trials, one death was reported in an observational study by Mirea et al. in 2021.^54^

**Table 2:** Overview of AAV gene therapy in real-world studies

No.	Disease	AAV gene therapy	Vector type	Dose	Route	No. of participants at time of treatment	Age at injection	No. of patients dosed	No. of patients receiving immunosuppressants	AEs associated with AAV gene therapy	Immunosuppressive protocol	Clinical evidence of immunosuppression effectiveness	Clinical evidence of AAV gene therapy effectiveness	Reference
1	SMA	onasmnogene abeparvovec	AAV9	1.1 × 10^14^ vg/kg	intravenous	*n* = 99	median age: 10 months	*n* = 99	99	asymptomatic thrombocytopenia elevated troponin I levels elevated liver enzyme levels more pronounced transaminitis was observed in 70 patients	prednisolone (1 mg/kg/day) for 30 days; prednisolone (2 mg/kg/day) if transaminase levels exceeded 2-fold the ULNs IV methylprednisolone in 5 patients with worsening acute transmanisits *N* = 1 received mycophenolate for chronic transaminitis	reduced liver enzyme levels	after OA infusion, mean ± SD change in CHOP-INTEND score was 11.0 ± 10.3 with increased score in 66/78 patients (84.6%) patients aged <6 months had a 13.9 points higher gain in CHOP-INTEND score than patients ≥2 years, indicative of improved motor function	Gowda et al., 2024^111^
2						*n* = 168	median age: 3 months	*n* = 168	N/A	hepatotoxicity (*n* = 49/167; 29.3%), transient thrombocytopenia (*n* = 23/167; 13.8%), cardiac AEs (*n* = 22/167; 13.2%), and TMA (*n* = 1; 0.6%)	prednisolone (1 mg/kg/day)	reduced liver enzyme levels	event-free survival dramatically improved in patients; improved CHOP-INTEND scores; achieved motor milestones	Servais et al., 2024^112^
3						*n* = 76	median age: 16.8 months	*n* = 76	*n* = 76	SAEs were in 8 (11%) children, mostly subacute hepatopathy (*n* = 7 [9%]) pyrexia (*n* = 47, 62%) vomiting or loss of appetite (*n* = 41, 54%) minor upper airway infection (*n* = 6, 8%) petechiae (*n* = 1, 1%) liver enzyme elevation (*n* = 56, 74%) thrombocytopenia (*n* = 59, 78%) cardiac AEs (*n* = 2)	prednisolone (1 mg/kg/day) for ≥30 days	liver enzyme levels normalized	significant improvements in CHOP-INTEND and HMFSE scores in 49 patients achievement of motor milestones	Weiβ et al., 2022^83^
4						*n* = 9	1.7–48 months	*n* = 9	*n* = 9	fever (*n* = 6, 66.7%) pyrexia (*n* = 47, 62%) Vomiting (*n* = 6, 66.7%) diarrhea (*n* = 3, 33.3%) thrombocytopenia (*n* = 5, 55.6%) hypertransaminasemia (*n* = 7, 77.8%) liver echogenicity changes (*n* = 1, 11.1%) increased troponin I (*n* = 9, 100%)	prednisolone (1 mg/kg/day)	liver enzyme levels normalized	all patients retained autonomous respiratory capacity without the need for tracheostomy or permanent ventilation and were autonomous in feeding CHOP-INTEND scores increased in patients over time	Bitetti et al., 2013^113^
5						*n* = 1	13 months	*n* = 1	*n* = 1	unreported	prednisolone (1 mg/kg/day)	liver enzyme levels normalized	improved motor and respiratory function, decreased saliva aspirations	Nanri et al., 2024^114^
6						*n* = 1	4 months	*n* = 1	*n* = 1	fatal TMA	oral steroid (1 mg/kg/day)	unreported	not reported due to patient fatality	Guillou et al., 2022^25^
7						*n* = 13	25.4–48.0 months	*n* = 13	unreported	no SAEs were related to gene therapy	unreported	unreported	no patients have required increased respiratory support, 2 patients can now stand with assistance	Mendell et al., 2021^14^
8						*n* = 1	4.5 years	*n* = 1	*n* = 1	fever, nausea, vomiting, elevated liver enzyme levels, hepatotoxicity, rise in NT-proBNP (heart failure), jaundice, TMA	prednisolone (1 mg/kg/day)	the elevated liver and heart enzymes more than halved after 1 week with increased dose of prednisolone on day 8 after gene therapy, the patient developed TMA and this resolved within 1 week of eculizumab therapy	Unreported	Witte et al., 2022^115^
9						*n* = 1	7 weeks	*n* = 1	*n* = 1	thrombocytopenia, feeding intolerance, mild hepatic dysfunction	prednisolone (1 mg/kg/day)	transaminases were not markedly elevated, safe administration in a patient with initial AAV9 antibody titers >1:50	walks independently, climbs stairs, has no scoliosis and does not need respiratory support	Eisenkölbl and Pühringer, 2024^116^
10						*n* = 8	7–445 days	*n* = 8	*n* = 8	cardiac AEs (*n* = 2)	prednisolone (1 mg/kg/day)	reduced liver enzyme levels	improvement in CHOP-INTEND scores at 6-month review	Favia et al., 2024^117^
11						*n* = 46	7–49.5 months	*n* = 46	*n* = 46	asymptomatic elevated liver enzyme levels	prednisolone (1 mg/kg/day)	only *n* = 5 had raised liver enzymes and these were asymptomatic commented that earlier treatment (i.e., before significant motor neuron loss) led to a much smaller proportion of patients needing >60 days of steroid treatment	improvement in motor milestones with no significant functional decline some patients also had improved oral feeding and reduced need for respiratory support	Waldrop et al., 2024^118^
12						*n* = 21	1–23 months	*n* = 21	*n* = 21	modest elevated liver enzyme levels	prednisolone (1 mg/kg/day)	reduced liver enzyme levels	*n* = 2 experienced stabilization, *n* = 17 experienced improvement in motor function	Waldrop et al., 2020^119^
13						*n* = 11	3.6 weeks	*n* = 11	*n* = 11	unreported	prednisolone (1 mg/kg/day)	N/A	reduced sleep disordered breathing	Chiang et al., 2023^120^
14						*n* = 33	unreported	*n* = 33	unreported	unreported	unreported	unreported	increase in maximal mouth opening	Beri et al., 2023^121^
15						*n* = 25	4–43 months	*n* = 25	*n* = 25	elevation of liver enzymes and thrombocytopenia; low-grade fever, 1/25 vomiting, 1/25 borderline high blood pressure	prednisolone (1 mg/kg/day) with increase to 2 mg/kg/day	reduced liver enzyme levels; improved platelet levels	improved motor functionals scores; significant improvements in CHOP-INTEND scores	Chencheri et al., 2023^122^
16						*n* = 16	1.5–3.4 years	*n* = 16	unreported	unreported	unreported	unreported	10/16 had significant kyphosis, 9/16 has scoliosis	Soini et al., 2023^123^
17						*n* = 46 (another 21 patients with ≥6 month follow-up after OA infusion.)	22 days–58 months (1/67 child age of 72 months)	*n* = 46 (for 12 months)	*n* = 67	thrombocytopenia, 15 (22.4%) patients had pyrexia, 14 (20.9%) vomiting or loss of appetite, elevated liver enzyme levels	prednisolone (1 mg/kg/day); adjusted to 2 mg/kg per day when increased AST and ALT levels (more than twice normal values) were detected	reduced liver enzyme levels	overall significant CHOP-INTEND improvement between T0 and T12 (sitting) 36/46 children with 1-year follow-up (78%) did not need for nutritional support at baseline after 12 months from the OA, they all remained orally fed the remaining 10 (22%) remained tube feeding 10 patients (22%) did not need non-invasive ventilation at baseline; 9 of them (90%) remained on spontaneous breathing after 12 months, only 1 needed for non-invasive ventilation	Pane et al., 2023^124^
18						*n* = 2	18 and 21 days	*n* = 2	*n* = 2	necrotizing enterocolitis, hematochezia, elevated liver enzymes, vomiting, thrombocytosis in one patient, blood in stool	prednisolone (1 mg/kg/day) with increase to 2 mg/kg/day	reduced liver enzyme levels	unreported	Gaillard et al., 2023^125^
19						*n* = 25	11 days and 23 months	*n* = 25	*n* = 25	fever, respiratory distress, upper respiratory viral illness, thrombocytopenia, elevated liver enzyme levels	prednisolone (1 mg/kg/day)	reduced liver enzyme levels	no regression in motor abilities, gradual improvement in motor function and no recurrent infections and illnesses following OA better progress in motor function observed in patients who received OA earlier and who were presymptomatic	Tokatly Latzer et al., 2023^126^
20						*n* = 2	121 and 42 days	*n* = 2	unreported	unreported	unreported	unreported	patient 1 became bedridden despite receiving OA (extremities movement improved, predominantly in the upper extremities, and the paradoxical respiration and tongue fasciculation disappeared the CHOP INTEND score decreased to 15 before treatment but improved to 34 after treatment he did not need respiratory support and could consume food orally at the age of 2 years and 1 month but had achieved no motor development milestones other than rolling over he is undergoing physical and occupational therapy). Patient 2 achieved normal motor development (head control, rolling over, sitting without support, standing with support, independent walking, beginning to run	Sawada et al., 2022^127^
21						*n* = 9	19–527 days	*n* = 9	*n* = 9	thrombocytopenia, elevated liver enzyme levels	prednisolone (1 mg/kg/day)	reduced liver enzyme levels	improvements of motor function. 3/6 SMA type 1 patients required nutritional support, 4/6 developed scoliosis, 1/6 sleep disturbed breathing SMA type 2 patient and 2 treated at pre-symptomatic stage did not require nutritional or respiratory support and did not develop a scoliosis	Stettner et al., 2023^128^
22						*n* = 1	5 months	*n* = 1	*n* = 1	fever and loss of appetite, elevated liver enzyme levels, thrombocytopenia, hyper-ferritinemia	prednisolone (1 mg/kg/day) with increase to 2 mg/kg/day	reduced liver enzyme levels, normalized platelet count	improvement in motor function; acquired a stable sitting position, maintained kneeling position with anterior support, stand unaided with upper limbs support, no respiratory problems. Normal neurocognitive and speech profile, fed by mouth and no swallowing problems	Tosi et al., 2022^129^
23						*n* = 6 (5 type 1 and 1 type 2)	7–24 months	*n* = 6	*n* = 6	elevated liver enzyme levels	prednisolone (1 mg/kg/day)	reduced GGT, ALT and AST levels	motor function improvements, no requirements for permanent ventilatory support and no case of mortality	Lee et al., 2022^130^
24						*n* = 7	7.5–19.2 months	*n* = 7	*n* = 7	fever and/or emesis, thrombocytopenia, elevated liver enzyme levels	prednisolone (1 mg/kg/day)	reduced ALT and AST levels, normal platelet count	motor function improvements	Matesanz et al., 2021^131^
25						*n* = 9	≤2 years	*n* = 9	*n* = 9	elevated liver enzyme levels, vomiting, reduced appetite, high prothrombin time, elevated bilirubin	prednisolone (1 mg/kg/day)	reduced liver enzyme levels	significant improvements in CHOP INTEND scores	Ali et al., 2021^132^
26						*n* = 10	19 months	*n* = 10	*n* = 10	hyperthermia, vomiting, lethargy and/or loose stool, thrombocytopenia, elevated liver enzyme levels	prednisolone (1 mg/kg/day) with increase to 2 mg/kg/day	reduced liver enzyme levels, reduced platelet count	relatively safe and effective with improvements of motor skills	Nevmerzhitskaya et al., 2021^133^
27						*n* = 21	0.65–24 months	*n* = 21	*n* = 21	vomiting, transaminitis and thrombocytopenia	prednisolone (1 mg/kg/day)	reduced liver enzyme levels, reduced platelet count	stabilization or improvement in bulbar or respiratory function	D'Silva et al., 2022^109^
28						*n* = 8	10–37 months	*n* = 8	*n* = 8	temporary increase in body temperature (> 38.5°C), vomiting, elevated liver enzyme levels, asymptomatic thrombocytopenia, 2/8 patients experienced an increase of pre-existing tremor	prednisolone (1 mg/kg/day) 7/8 patients; methylprednisolone (20 mg/kg) 1/8 patients	reduced liver enzyme levels, reduced platelet count, thrombocytes normalized	sitting without support, no respiratory support and tube feeding	Friese et al., 2021^65^
29						Group 1 *n* = 7; Group 2 *n* = 6	2–6 months	*n* = 7	*n* = 7	fever, vomiting, lack of appetite, mild thrombocytopenia, elevated liver enzyme levels	prednisolone (1 mg/kg/day)	reduced liver enzyme levels	OV after nusinersen did not provide supplementary benefits for motor function or respiratory status early OV treatment results in better outcomes	Mirea et al., 2021^54^
30						*n* = 1	2 months	*n* = 1	*n* = 1	elevated liver enzyme levels	prednisolone (1 mg/kg/day)	reduced liver enzyme levels	head control at 4 months of age, independent walking at 18 months, pulling to stand, walk, and sit independently, and is reaching for objects; eating orally, gaining weight, no respiratory concerns, can say up to 10 words	Nigro et al., 2023^134^
31	SMA	onasmnogene abeparvovec (with nusinersen and risdiplam)	AAV9	1.1 × 10^14^ vg/kg	intravenous	*n* = 1	4 months	*n* = 1	*n* = 1	moderate/severe TMA, hypertransaminasemia	prednisolone (1 mg/kg/day)	liver enzyme levels normalized	nusinersen: improvement in motor and bulbar function; onasmnogene abeparvovec: Sitting with support at 2 years old and acquired independent sitting at 27 months. Patient began to eat semisolids orally; risdiplam: further improvements in both motor and bulbar functions one year after risdiplam therapy	Bitetti et al., 2023^113^
32	Inherited Retinal Dystrophy (RPE-65 mediated)	VN	AAV2	1.5 × 10^11^ vg per eye	Subretinal	*n* = 3	22 months, 2 years, 5 years	*n* = 3	*n* = 3	*n* = 3 acute subretinal deposits	prednisolone (1 mg/kg/day)	N/A	improved visual function with macular and inferior subretinal deposits improved or resolved	Lopez et al., 2023^135^
33	Inherited Retinal Dystrophy (RPE-65 mediated)	VN	AAV2	1. × 10^11^ vg per eye	Subretinal	*n* = 6	18–49 years	*n* = 6	*n*-6	retinal atrophy in 10/12 eyes (8 mild/2 severe) increased ocular pressure (3 patients: 6 eyes) increased intraocular inflammation (2 eyes) cataracts (4 eyes) glaucoma surgery (2 patients: 4 eyes) higher occurrence of retinal atrophy and increased IOP than previously reported	prednisolone (1 mg/kg/day)	N/A	best-corrected visual acuity remained stable (baseline: 1.28 (±0.71) vs. last follow-up: 1.46 (±0.60); *p* = 0.25). average white full-field stimulus testing showed a trend toward improvement (baseline: −4.41 (±10.62) dB vs. last follow-up: −11.98 (±13.83) dB; *p* = 0.18).	Kiraly et al., 2023^55^
34	Inherited Retinal Dystrophy (RPE-65 mediated)	VN	AAV2	1.5 × 10^11^ vg per eye	Subretinal	*n* = 1	39 years	*n* = 1	*n* = 1	foveal ellipsoid zone loss	prednisolone (1 mg/kg/day)	N/A	the first eye showed improvement in rod photoreceptor function with increased peripheral and low luminance vision (baseline VA: 0.9 logMAR and 2-years post-operative VA: 0.7 logMAR. 2ND eye developed loss of foveal photoreceptors. FST improvements were maintained in both eyes. macular edema resolved by 6 weeks of VN	Jalil et al., 2023^136^
35	hemophilia B	Etranacogene dezaparvovec	AAV5	2 × 10^13^ vg/kg	intravenous	*n* = 3	43, 47 and 50 years	*n* = 3	*n* = 3	headache transient elevation of C-reactive protein	N/A	N/A	stable durable FIX activity remained after 3 years discontinued FIX prophylaxis in all patients clinical phenotype from severe/moderately severe to mild/non-hemophilic 100% decrease in bleeds in 2/3 and 92% in patient 3	Von Drygalski et al., 2019^106^

FIX, factor IX; GGT, gamma-glutamyl transferase; NT-proBNP; ULN, upper limit of normal.

Elevations in liver enzymes were successfully treated with prednisolone, which can also increase the response to treatment by suppressing the antigen-specific T cell response that clear transduced cells and thus result in a loss of transgene expression.^11^ In the treatment of inherited retinal dystrophy, all patients reported subjective vision improvement after VN gene therapy. The overall safety and effectiveness of VN treatment align with previous VN clinical trials, excluding the higher occurrence of retinal atrophy and increased ocular pressure observed.^55^

### Treatment-associated AEs

AEs were observed in 30 of 38 clinical trials involving 19 AAV gene therapies (Table 3). Increased levels of liver enzymes or liver toxicity were the most frequently reported AE, which was recorded in 21 clinical trials (70%). For the purpose of this review, elevated liver enzymes or liver toxicity included any reference to elevated gamma-glutamyl transferase (GGT), ALT, AST, liver enzymes, liver toxicity, or hepatoxicity. Other frequent AEs included vomiting and nausea (*n* = 11 [37%]), pyrexia (*n* = 10 [33%]), and fatigue (*n* = 6 [20%]). Although AEs were recorded in 30 of the 38 clinical trials, SAEs were only observed in 15 of the included studies (Table 3), with the highest frequency of SAEs observed in clinical trials for SMA (*n* = 4), hemophilia A (*n* = 3), hemophilia B (*n* = 3), and DMD (*n* = 2). Four deaths were reported in patients in both low- and high-dose groups because an SAE associated with the treatment protocol in a trial for X-linked myotubular myopathy with resamirigene bilparvovec, an AAV8 clinical vector (NCT03199469).^56^ Of note, these patients had presented with cholestasis before vector dosing. Due to the clinical vector serotypes used for these diseases, the most common serotypes resulting in SAEs were AAV9 (*n* = 4, for SMA), AAV5 (*n* = 2 for hemophilia A and *n* = 1 for hemophilia B), and rhAAV74 (*n* = 2, for DMD) after systemic delivery. As detailed in Table 1, SAEs were associated with the systemic delivery of AAVs with the exception of X-linked retinitis pigmentosa. Elevations in liver enzyme levels were reported as SAEs after clinical vector administration in clinical trials for DMD (*n* = 2), hemophilia A (*n* = 2), hemophilia B (*n* = 3), mucopolysaccharidosis (MPS) type IIIB (*n* = 1), and SMA (*n* = 4). Although most studies described management of elevated liver enzyme SAEs via treatment with corticosteroids (even when corticosteroids were administered prophylactically to patients), two studies (NCT03769116 ^57^ and EudraCT 2012-000856-33^58^ did not describe any treatment for AEs observed in patients and one reported that there was no clear association between the resolution of elevated ALT levels and prednisolone use.^59^ The immunosuppressive regimen performed in the trials was prophylactic and AEs recorded in patients were resolved without intervention. All other SAEs relating to clinical vector administration in the studies are detailed in Table 3. In contrast with clinical trials for the treatment of DMD, hemophilia A and B, MPS type IIIB, and SMA, a clinical trial investigating X-linked retinitis pigmentosa (NCT03116113) did not observe any type of SAEs associated with liver enzyme levels after subretinal injection of cotoretigene toliparvovec (BIIB112/AAV8-RPGR).^60^ Instead, the SAEs recorded in the study were decreased visual acuity, noninfective retinitis, retinal detachment, and visual impairment. These inflammatory events were further managed with oral prednisolone.

**Table 3:** Treatment-associated AE overview

No.	Disease	AAV gene therapy	Serotype	No. of patients dosed	Treatment-related SAEs	Treatment-related AEs	Frequency of treatment-related AEs	Management of AEs	NCT (phase)	Reference
1	DMD	Delandistrogene moxeparvovec	rAAVrh74	2	None reported	bruising decreased lymphocyte count bleeding at femoral catheterization site vomiting NB: authors only described events as treatment-emergent and were not specified as treatment-related	100% (*n* = 2)	no treatment needed for AEs. prednisone administration to patients was prophylactic.	NCT03333590 (1/2)	Flanigan et al., 2022^64^
2		Delandistrogene moxeparvovec	rAAVrh74	4	None reported	vomiting (*n* = 9) nausea (*n* = 1) fatigue (*n* = 1) asthenia (*n* = 1) decreased appetite (*n* = 2) elevated liver enzyme levels (*n* = 4)	100% (*n* = 4)	elevated γ-glutamyl transferase resolved with corticosteroids	NCT03375164 (1 and 2)	Mendell et al., 2020^66^
3		Delandistrogene moxeparvovec	rAAVrh74	20	rhabdomyolysis elevated liver enzyme levels liver injury	vomiting decreased appetite nausea elevated liver enzyme levels abdominal pain increased blood bilirubin pain in extremity rhabdomyolysis pyrexia	AE: 100% (*n* = 21) SAE: 5% (*n* = 1)	no treatment described for AEs prednisone administration to patients was prophylactic.	NCT03769116 (2)	Mendell et al., 2023^57^
4		Delandistrogene moxeparvovec	rAAVrh74	20	elevated liver enzyme levels vomiting	vomiting decreased appetite increased glutamate dehydrogenase nausea constipation fatigue elevated liver enzyme levels increased blood creatine phosphokinase thrombocytopenia abdominal pain upper increased blood lactate dehydrogenase headache hemoglobinuria pyrexia diarrhea	AE: 90% (*n* = 18) SAE: 10% (*n* = 2)	patients received prophylactic prednisone or prednisolone (1 mg/kg) in addition to baseline corticosteroid dose, for a total ≤60 mg/day, which was continued for ≥60 days post-treatment and subsequently tapered, depending on serum γ-glutamyl transferase levels	NCT04626674 (1b)	Zaidman et al., 2023^103^
5	hemophilia A	Valoctocogene roxaparvovec	AAV5	134	elevated liver enzyme levels headache arthralgia nausea	elevated liver enzyme levels headache arthralgia nausea	AE: 92% (*n* = 123) SAE: 4% (*n* = 5)	immunosuppressants were given in relation to an alanine transferase rise median duration of elevation in ALT was 21 days there was no apparent relationship between the development of anti-AAV5 antibodies and factor VIII activity	NCT03370913 (3)	Zaidman et al., 2023^103^
6		Valoctocogene roxaparvovec	AAV5	9	elevated liver enzyme levels	elevated liver enzyme levels	AE by year: Y1: 86% (*n* = 6) Y2: 14% (*n* = 1) Y3: 14% (*n* = 1) Y4: 29% (*n* = 2) SAE by year: Y1: 17% (*n* = 1)	corticosteroids were used prophylactically or in response to elevated ALT levels (1.5× above baseline) there was no clear association between the resolution of the elevated ALT level and prednisolone use	NCT02576795 (1/2)	Rangarajan et al., 2017, Long et al., 2021, Fong et al., 2022, Pasi et al., 2021^59^^;^^67^^;^^81^^;^^85^
7		Giroctocogene fitelparvovec	AAV6	11	Pyrexia Hypotension	elevated liver enzyme levels tachycardia fatigue myalgia	SAE: 27% (*n* = 3) AE:100% (*n* = 11)	liver enzyme levels were managed with tapering corticosteroid administration hypotension and pyrexia resolved with treatment with electrolytes, norepinephrine, ondansetron, glucose, and paracetamol	NCT03061201 (1/2)	Leavitt et al., 2024^104^
8	hemophilia B	BBM-H901	dsAAV843	10	None reported	pyrexia elevated liver enzyme levels	pyrexia (10%, *n* = 1) aminotransferase elevations (10%, *n* = 1)	glucocorticoid administration	NCT04135300 (1)	Xue et al., 2022^63^
9		Etranacogene dezaparvovec	AAV5	3	None reported	headache mild elevation in C-reactive protein levels	33% (*n* = 1)	AEs resolved without intervention	NCT03489291 (2b)	Von Drygalski et al., 2019^106^
10		Etranacogene dezaparvovec	AAV6	54	None reported	Arthralgia headache fatigue elevated liver enzyme levels blood creatine kinase increase back pain influenza-like illness diarrhea nausea	69% (*n* = 37)	glucocorticoid administration for liver enzyme elevations	NCT03569891 (3)	Pipe et al., 2023^16^
11		Verbrinacogene setparvovec	AAV2/S3	10	elevated liver enzyme levels decreased coagulation FIX pulmonary sepsis arteriovenous fistula thrombosis	elevated liver enzyme levels fatigue increased coagulation FIX muscle spasms/musculoskeletal pain/myalgia dyspepsia/eructation AV fistula thrombosis decreased coagulation FIX headache pulmonary sepsis somnolence	AEs: 80% (*n* = 8) SAE: 70% (*n* = 7) NB: AEs were not observed in patients treated with low dose vector (3.84 × 10^11^ vg/kg, *n* = 2).	increase in liver enzyme levels were managed with intravenous methylprednisolone and tacrolimus	NCT03369444 and NCT03641703 (1/2)	Chowdary et al., 2022^48^
12		AMT-060	AAV5	10	elevated liver enzyme levels Pyrexia	elevated liver enzyme levels pyrexia anxiety drug ineffective palpitations headache prostatitis rash	AEs: 60% (*n* = 6) SAEs: 30% (*n* = 3)	tapering course of prednisolone	NCT02396342 (1/2)	Majowicz et al., 2019, Miesbach et al., 2018^68^^;^^86^
13		SPK-9001	A novel, bioengineered single-stranded adeno-associated viral vector carrying human FIX variant	10	none reported	elevated liver enzyme levels	10% (*n* = 1)	2 patients required 60 mg prednisone in the context of ALT rises/immune responses this was tapered down over 119 and 130 days respectively	NCT02484092 (1/2)	George et al., 2017^105^
14		BAX 335 (scAAV8.FIXR338)	AAV8	7	None reported	fatigue feeling flushed headache infleunza-like symptoms ankle swelling elevated liver enzyme levels high blood pressure abscess	57% (*n* = 4)	prednisone administration upon detection of high liver enzyme levels	NCT01687608 (1/2)	Konkle et al., 2021^82^
15		scAAV2/8-LP1-hFIXco	rAAVrh74	10	elevated liver enzyme levels	lethargy elevated liver enzyme levels anemia	100% (*n* = 10)	prednisone, 60 mg/patient with subsequent tapering of the dose patient 5: 9 weeks patient 6: 4 weeks	NCT00979238 (1/2)	Nathwani et al., 2011, Nathwani et al., 2014^43^^;^^44^
16	LHON	scAAV2-P1ND4v2	AAV2	28	none reported	Uveitis	29% (*n* = 8)	Topical prednisolone	NCT02161380 (1)	Lam et al., 2022^107^
17	MPS type IIIB	rAAV2/5-hNaGlu	AAV2/5	4	respiratory tract infection elevated liver enzyme levels diarrhea	upper respiratory tract infection minor anesthesia-related diarrhea or gastroenteritis elevated liver enzyme levels behavior anemia transient hydroelectric disorder tonsillectomy adenoidectomy minimum mitral insufficiency transient loss of appetite NB: authors only described events as treatment-emergent and were not specified as treatment-related	100% (*n* = 4)	no treatment described for AEs. prednisone administration to patients was prophylactic.	EudraCT, number 2012-000856-33, and the International Standard Clinical Trial Registry, number ISRCTN19853672 (1/2)	Tardieuc et al., 2017^58^
18		rAAV2/5-hNaGlu	AAV2/5	4	none reported	upper respiratory tract infection diarrhea or gastroenteritis elevated liver enzyme levels anemia behavior bronchitis cough pyrexia conjunctivitis sleeping disorders Atopic dermatitis NB: Authors only described events as treatment-emergent and were not specified as treatment-related	100% (*n* = 4)	no treatment described for AEs prednisone administration to patients was prophylactic.	NCT03300453 (1/2)	Gougeon et al., 2021, Deiva et al., 2021^49^^;^^50^
19	RPE65-Deficient LCA and Severe Early-Childhood Onset Retinal Dystrophy	rAAV2-CB-hRPE65	AAV2	12	none reported	ocular hyperemia photopsia	25% (*n* = 3)	no treatment described for AEs topical corticosteroid administration to patients was prophylactic	NCT00749957 (1/2)	Weleber et al., 2016^108^
20	RPE65-mediated inherited retinal dystrophy (LCA)	AAV8-coRPGR codon optimized RPGR	AAV8	18	none reported	anterior uveitis subretinal inflammation NB: this was only observed in patients in medium and high dose groups	33% (*n* = 6)	no treatment described for AEs prednisolone administration to patients was prophylactic	NCT03116113 (1/2)	Cehajic-Kapetanovic et al., 2020^60^
21	SMA	OA	AAV9	76	subacute hepatopathy elevated liver enzyme levels acute liver dysfunction	pyrexia vomiting thrombocytopenia rashes	74% (*n* = 56)	escalating prednisolone to 2 mg/kg per day for about 4 weeks from 1 mg/kg	N/A (observational study)	Weiβ et al., 2022^83^
22		OA	AAV9	15	none reported	liver toxicity thrombocytopenia increased troponin	53% (*n* = 8)	prednisolone, varying doses depending on AE and patient	NCT03505099 (3)	Strauss et al., 2022^24^
23		OA	AAV9	32	elevated liver enzyme levels (*n* = 1)	hypertension elevated liver enzyme levels lymphadenopathy pyrexia vomiting prolonged thromboplastin time increased blood creatine phosphokinase cardiac murmur abnormal hair growth hepatomegaly pericardial effusion sinus tachycardia	37.5% (*n* = 12)	increased prednisolone dose administered to patient with elevated liver enzymes other AEs were resolved without intervention	NCT03381729 (1)	Finkel et al., 2023^70^
24		OA	AAV9	21	None reported	vomiting elevated liver enzyme levels	100% (*n* = 21)	antiemetic medication oral and enteral feeding to maintain hydration systemic corticosteroid administration	N/A (cohort study)	D'Silva et al., 2022^109^
25		OA	AAV9	15	elevated liver enzyme levels (*n* = 2)	elevated liver enzyme levels below SAE cutoff point (*n* = 2)	27% (*n* = 4)	prednisolone treatment	NCT02122952 (1)	Mendell et al., 2017^11^
26		OA	AAV9	8	None reported	elevated liver enzymes pyrexia vomiting reduced appetite exacerbation of hand tremor thrombocytopenia Increase in troponin I/T, CRP, and monocyte counts	100% (*n* = 8)	prednisolone dose increased above 1 mg/kg	N/A (retrospective analysis)	Friese et al., 2021^65^
27		OA	AAV9	33	pyrexia elevated liver enzyme levels gastroenteritis rhinovirus infection virus infection feeding disorder hypernatremia thrombocytopenia abnormal coagulation test	pyrexia upper respiratory infection elevated liver enzyme levels vomiting constipation gastroenteritis rhinovirus infection virus infection respiratory tract infection cough diarrhea pneumonia gastro-esophageal reflux disease nasopharyngitis hypertension	AE: 73% (*n* = 24) SAE: 18% (*n* = 6)	following the recommendation to increase prophylactic prednisolone dosing for the first 3 days from 1 to 2 mg/kg per day, p (27%) patients received an initial dose of 2 mg/kg per day the duration of prednisolone dosing ranged from 54 to 235 days (median 65.0 [IQR 13.0]; mean 80.9 [SD 41.2]) and mean daily dose ranged from 0.5 to 1.6 mg/kg per day 2 patients switched to an equivalent dose of hydrocortisone (using a conversion ratio of 1:4) as an alternative to prednisolone on day 165 and day 132 (extended use after the tapering period was prescribed to treat elevated liver enzymes).	NCT03306277 (3)	Day et al., 2021, Mercuri et al., 2021^12^^;^^13^
28	X-linked myotubular myopathy	Resamirigene bilparvovec	AAV8	24	increased total bilirubin values elevation in liver enzyme levels NB: these resulted in death in 4 participants	pyrexia creatine phosphokinase increase respiratory tract infection	AE: 96% (*n* = 23) SAE: 46% (*n* = 11)	prednisone administration was prophylactic. participants presenting with SAEs resulting in death were provided with high-dose prednisolone and other immune-modulating therapies (e.g., prolonged or increased dose of prednisolone, anakinra, tocilizumab, and ruxolitinib) with no apparent benefit.	NCT03199469 (2/3)	Shieh et al., 2023^56^
29	X-linked retinitis pigmentosa	Cotoretigene toliparvovec	AAV8	18	ocular inflammation leading to reduced visual acuity	noninfective retinitis corneal deposits	AE: 39% (*n* = 7) SAE: 11% (*n* = 2)	ocular inflammation-associated SAE were treated with corticosteroids in one participant	NCT03116113 (1)	von Krusenstiern et al., 2023^110^
30	X-linked retinoschisis	AAV8-RS1	AAV8	11	None reported	ocular inflammation mild vitritis anterior chamber inflammation	55% (*n* = 6)	subject 9 was pretreated with prednisone and topical steroid at 2 days before dosing and continued beyond day 14 subjects 10 and 11 were treated with cyclosporine at 175 mg twice daily beginning 3 weeks before dosing, and MMF at 500 mg twice daily at 3 weeks and 1000 mg twice daily at 2 weeks before dosing plus prednisone at 60 mg 2 days before vector dosing	NCT02317887 (1/2)	Mishra et al., 2021^71^

FIX, factor IX; LHON, Leber hereditary optic neuropathy.

The risk of adverse immune responses from gene therapy is generally related to the type of vector that is used as well as, dose, delivery route,^61^ and transgene sequence, which has been found to occur in clinical trials for DMD gene therapy.^62^We have summarized the immune responses associated with several vectors (AAV1-2, 2/3, 2.5, 2/8, 5, 8, 9, rh8, rh.10, rh.74, 843, and γ-RV), routes (intramuscular, subretinal, intravitreal, intracranial, intraparenchymal, intracerebro-ventricular, low respiratory tract, and cell therapy intravenous) and immunosuppression protocols (Table 4).

**Table 4:** Immunosuppression-associated AEs overview

No.	Disease	AAV gene therapy	Vector type	Dose	Route	No. of participants at time of treatment	Age at injection	No of patients dosed	No. of patients receiving immunosuppressants	AEs associated with AAV gene therapy	Immunosuppressive protocol	Clinical evidence of immunosuppression effectiveness	Clinical evidence of AAV gene therapy effectiveness	Reference
1	SMA	onasmnogene abeparvovec	AAV9	1.1 × 10^14^ vg/kg	intravenous	*n* = 99	median age: 10 months	*n* = 99	99	asymptomatic thrombocytopenia elevated troponin I levels elevated liver enzyme levels more pronounced transaminitis was observed in 70 patients	prednisolone (1 mg/kg/day) for 30 days; prednisolone (2 mg/kg/day) if transaminase levels exceeded 2-fold the ULNs; IV methylprednisolone in 5 patients with worsening acute transmanisits *n* = 1 received mycophenolate for chronic transaminitis	reduced liver enzyme levels	after OA infusion, mean ± SD change in CHOP-INTEND score was 11.0 ± 10.3 with increased score in 66/78 patients (84.6%); patients aged <6 months had a 13.9 points higher gain in CHOP-INTEND score than patients ≥2 years, indicative of improved motor function.	Gowda et al., 2024^111^
2						*n* = 168	median age: 3 months	*n* = 168	N/A	hepatotoxicity (*n* = 49/167; 29.3%), transient thrombocytopenia (*n* = 23/167; 13.8%), cardiac AEs (*n* = 22/167; 13.2%), and TMA (*n* = 1; 0.6%)	prednisolone (1 mg/kg/day)	reduced liver enzyme levels	event-free survival dramatically improved in patients; improved CHOP-INTEND scores; achieved motor milestones	Servais et al., 2024^112^
3						*n* = 76	median age: 16.8 months	*n* = 76	*n* = 76	SAEs were in 8 (11%) children, mostly subacute hepatopathy (*n* = 7 [9%]). pyrexia (*n* = 47, 62%) vomiting or loss of appetite (*n* = 41, 54%) minor upper airway infection (*n* = 6, 8%) petechiae (*n* = 1, 1%) liver enzyme elevation (*n* = 56, 74%) thrombocytopenia (*n* = 59, 78%) cardiac AEs (*n* = 2)	prednisolone (1 mg/kg/day) for ≥30 days	liver enzyme levels normalized	significant improvements in CHOP-INTEND and HMFSE scores in 49 patients Achievement of motor milestones	Weiβ et al., 2022^83^
4						*n* = 9	1.7–48 months	*n* = 9	*n* = 9	fever (*n* = 6, 66.7%) pyrexia (*n* = 47, 62%) vomiting (*n* = 6, 66.7%) diarrhea (*n* = 3, 33.3%) thrombocytopenia (*n* = 5, 55.6%) hypertransaminasemia (*n* = 7, 77.8%) liver echogenicity changes (*n* = 1, 11.1%) Increased troponin I (*n* = 9, 100%)	prednisolone (1 mg/kg/day)	liver enzyme levels normalized	all patients retained autonomous respiratory capacity without the need for tracheostomy or permanent ventilation and were autonomous in feeding. CHOP-INTEND scores increased in patients over time	Bitetti et al., 2013^113^
5						*n* = 1	13 months	*n* = 1	*n* = 1	unreported	prednisolone (1 mg/kg/day)	liver enzyme levels normalized	improved motor and respiratory function, decreased saliva aspirations	Nanri et al., 2024^114^
6						*N* = 1	4 months	*n* = 1	*n* = 1	fatal TMA	Oral steroid (1 mg/kg/day)	unreported	not reported due to patient fatality	Guillou et al., 2022^25^
7						*n* = 13	25.4–48.0 months	*n* = 13	unreported	no SAEs were related to gene therapy	unreported	unreported	no patients have required increased respiratory support, 2 patients can now stand with assistance	Mendell et al., 2021^14^
8						*n* = 1	4.5 years	*n* = 1	*n* = 1	fever, nausea, vomiting, elevated liver enzyme levels, hepatotoxicity, rise in NT-proBNP (heart failure), jaundice, TMA	prednisolone (1 mg/kg/day)	the elevated liver and heart enzymes more than halved after one week with increased dose of prednisolone on day 8 after gene therapy the patient developed TMA and this resolved within 1 week of eculizumab therapy	unreported	Witte et al., 2022^115^
9						*n* = 1	7 weeks	*n* = 1	*n* = 1	thrombocytopenia, feeding intolerance, mild hepatic dysfunction	prednisolone (1 mg/kg/day)	transaminases were not markedly elevated, safe administration in a patient with initial AAV9 antibody titers >1:50	walks independently, climbs stairs, has no scoliosis and does not need respiratory support	Eisenkölbl and Pühringer, 2024^116^
10						*N* = 8	7-445 days	*n* = 8	*n* = 8	cardiac AEs (*n* = 2)	prednisolone (1 mg/kg/day)	reduced liver enzyme levels	improvement in CHOP-INTEND scores at 6 month review	Favia et al., 2024^117^
11						*n* = 46	7–49.5 months	*n* = 46	*n* = 46	asymptomatic elevated liver enzyme levels	prednisolone (1 mg/kg/day)	only *n* = 5 had raised liver enzymes and these were asymptomatic commented that earlier treatment (i.e., before significant motor neuron loss) led to a much smaller proportion of patients needing >60 days of steroid treatment	improvement in motor milestones with no significant functional decline. Some patients also had improved oral feeding and reduced need for respiratory support	Waldrop et al., 2024^118^
12						*n* = 21	1–23 months	*n* = 21	*n* = 21	modest elevated liver enzyme levels	prednisolone (1 mg/kg/day)	reduced liver enzyme levels	*n* = 2 experienced stabilization, *n* = 17 experienced improvement in motor function	Waldrop et al., 2020^119^
13						*n* = 11	3.6 weeks	*n* = 11	*n* = 11	unreported	prednisolone (1 mg/kg/day)	N/A	reduced sleep disordered breathing	Chiang et al., 2023^120^
14						*n* = 33	unreported	*n* = 33	unreported	unreported	unreported	unreported	increase in maximal mouth opening	Beri et al., 2023^121^
15						*n* = 25	4–43 months	*n* = 25	*n* = 25	elevation of liver enzymes and thrombocytopenia; low-grade fever, 1/25 vomiting, 1/25 borderline high blood pressure	prednisolone (1 mg/kg/day) with increase to 2 mg/kg/day	reduced liver enzyme levels; improved platelet levels	improved motor functionals scores; significant improvements in CHOP-INTEND scores	Chencheri et al., 2023^122^
16						*n* = 16	1.5–3.4 years	*n* = 16	unreported	unreported	unreported	unreported	10/16 had significant kyphosis, 9/16 has scoliosis	Soini et al., 2023^123^
17						*n* = 46 (another 21 patients with ≥6 month follow-up after OA infusion.)	22 days–58 months (1/67 child age of 72 months)	*n* = 46 (for 12 months)	*n* = 67	thrombocytopenia,15 (22.4%) patients had pyrexia,14 (20.9%) vomiting or loss of appetite, elevated liver enzyme levels	prednisolone (1 mg/kg/day); adjusted to 2 mg/kg per day when increased AST and ALT levels (more than twice normal values) were detected	reduced liver enzyme levels	overall significant CHOP-INTEND improvement between T0 and T12 (sitting). 36/46 children with one-year follow-up (78%) did not need for nutritional support at baseline; after 12 months from the OA, they all remained orally fed. The remaining ten (22%) remained tube feeding 10 patients (22%) did not need non-invasive ventilation at baseline; nine of them (90%) remained on spontaneous breathing after 12 months, only one needed for non-invasive ventilation	Pane et al., 2023^124^
18						*n* = 2	18 and 21 days	*n* = 2	*n* = 2	necrotizing enterocolitis, hematochezia, elevated liver enzymes, vomiting, thrombocytosis in one patient, blood in stool	prednisolone (1 mg/kg/day) with increase to 2 mg/kg/day	reduced liver enzyme levels	unreported	Gaillard et al., 2023^125^
19						*n* = 25	11 days and 23 months	*n* = 25	*n* = 25	fever, respiratory distress, upper respiratory viral illness, thrombocytopenia, elevated liver enzyme levels	prednisolone (1 mg/kg/day)	reduced liver enzyme levels	no regression in motor abilities, gradual improvement in motor function and no recurrent infections and illnesses following OA better progress in motor function observed in patients who received OA earlier and who were presymptomatic	Tokatly Latzer et al., 2023^126^
20						*n* = 2	121 and 42 days	*n* = 2	unreported	unreported	unreported	unreported	patient 1 became bedridden despite receiving OA (extremities movement improved, predominantly in the upper extremities, and the paradoxical respiration and tongue fasciculation disappeared the CHOP INTEND score decreased to 15 before treatment but improved to 34 after treatment he did not need respiratory support and could consume food orally at the age of 2 years and 1 month but had achieved no motor development milestones other than rolling over he is undergoing physical and occupational therapy). Patient 2 achieved normal motor development (head control, rolling over, sitting without support, standing with support, independent walking, beginning to run	Sawada et al., 2022^127^
21						*n* = 9	19–527 days	*n* = 9	*n* = 9	thrombocytopenia, elevated liver enzyme levels	prednisolone (1 mg/kg/day)	reduced liver enzyme levels	improvements of motor function 3/6 SMA type 1 patients required nutritional support, 4/6 developed scoliosis, 1/6 sleep disturbed breathing. SMA type 2 patient and two treated at pre-symptomatic stage did not require nutritional or respiratory support and did not develop a scoliosis	Stettner et al., 2023^128^
22						*n* = 1	5 months	*n* = 1	*n* = 1	fever and loss of appetite, elevated liver enzyme levels, thrombocytopenia, hyper-ferritinemia	prednisolone (1 mg/kg/day) with increase to 2 mg/kg/day	reduced liver enzyme levels, normalized platelet count	improvement in motor function; acquired a stable sitting position, maintained kneeling position with anterior support, stand unaided with upper limbs support, no respiratory problems normal neurocognitive and speech profile, fed by mouth and no swallowing problems	Tosi et al., 2022^129^
23						*n* = 6 (5 type 1 and 1 type 2)	7–24 months	*n* = 6	*n* = 6	elevated liver enzyme levels	prednisolone (1 mg/kg/day)	reduced GGT, ALT and AST levels	motor function improvements, no requirements for permanent ventilatory support and no case of mortality	Lee et al., 2022^130^
24						*n* = 7	7.5–19.2 months	*n* = 7	*n* = 7	fever and/or emesis, thrombocytopenia, elevated liver enzyme levels	prednisolone (1 mg/kg/day)	reduced ALT and AST levels, normal platelet count	motor function improvements	Matesanz et al., 2021^131^
25						*n* = 9	≤2 years	*n* = 9	*n* = 9	elevated liver enzyme levels, vomiting, reduced appetite, high prothrombin time, elevated bilirubin	prednisolone (1 mg/kg/day)	reduced liver enzyme levels	significant improvements in CHOP INTEND scores	Ali et al., 2021^132^
26						*n* = 10	19 months	*n* = 10	*n* = 10	hyperthermia, vomiting, lethargy and/or loose stool, thrombocytopenia, elevated liver enzyme levels	prednisolone (1 mg/kg/day) with increase to 2 mg/kg/day	reduced liver enzyme levels, reduced platelet count	relatively safe and effective with improvements of motor skills	Nevmerzhitskaya et al., 2021^133^
27						*n* = 21	0.65–24 months	*n* = 21	*n* = 21	vomiting, transaminitis and thrombocytopenia	prednisolone (1 mg/kg/day)	reduced liver enzyme levels, reduced platelet count	stabilization or improvement in bulbar or respiratory function	D'Silva et al., 2022^109^
28						*n* = 8	10–37 months	*n* = 8	*n* = 8	temporary increase in body temperature (above 38.5°C), vomiting, elevated liver enzyme levels, asymptomatic thrombocytopenia, 2/8 patients experienced an increase of pre-existing tremor	prednisolone (1 mg/kg/day) 7/8 patients; methylprednisolone (20 mg/kg) 1/8 patients	reduced liver enzyme levels, reduced platelet count, thrombocytes normalized	sitting without support, no respiratory support and tube feeding	Friese et al., 2021^65^
29						Group 1 *n* = 7; Group 2 *n* = 6	2–6 months	*n* = 7	*n* = 7	fever, vomiting, lack of appetite, mild thrombocytopenia, elevated liver enzyme levels	prednisolone (1 mg/kg/day)	reduced liver enzyme levels	OV after nusinersen did not provide supplementary benefits for motor function or respiratory status. Early OV treatment results in better outcomes	Mirea et al., 2021^54^
30						*n* = 1	2 months	*n* = 1	*n* = 1	elevated liver enzyme levels	prednisolone (1 mg/kg/day)	reduced liver enzyme levels	head control at 4 months of age, independent walking at 18 months, pulling to stand, walk, and sit independently, and is reaching for objects; eating orally, gaining weight, no respiratory concerns, can say up to 10 words	Nigro et al., 2023^134^
31						*n* = 1	4 months	*n* = 1	*n* = 1	moderate/severe TMA, hypertransaminasemia	prednisolone (1 mg/kg/day)	liver enzyme levels normalized	nusinersen: improvement in motor and bulbar function; onasmnogene abeparvovec: Sitting with support at 2 years old and acquired independent sitting at 27 months patient began to eat semisolids orally; risdiplam: further improvements in both motor and bulbar functions one year after risdiplam therapy	Bitetti et al., 2023^113^

No.	Disease	AAV gene therapy	Vector type	Dose	Route	No. of participants at time of treatment	Age at injection	No of patients dosed	No. of patients receiving immunosuppressants	AEs associated with AAV gene therapy	Immunosuppressive protocol	Clinical evidence of immunosuppression effectiveness	Clinical evidence of AAV gene therapy effectiveness	Reference
1	inherited retinal dystrophy (RPE-65 mediated)	VN	AAV2	1.5 × 10^11^ vg per eye	Subretinal	*n* = 3	22 months, 2 years, 5 years	*n* = 3	*n* = 3	*n* = 3 acute subretinal deposits	prednisolone (1 mg/kg/day)	N/A	Improved visual function with macular and inferior subretinal deposits improved or resolved	Lopez et al., 2023^135^
2	inherited retinal dystrophy (RPE-65 mediated)	VN	AAV2	1.5 × 10^11^ vg per eye	Subretinal	*n* = 6	18–49 years	*n* = 6	*n*-6	retinal atrophy in 10/12 eyes (8 mild/2 severe) increased ocular pressure (3 patients: 6 eyes) Increased intraocular inflammation (2 eyes) cataracts (4 eyes) glaucoma surgery (2 patients: 4 eyes) higher occurrence of retinal atrophy and increased IOP than previously reported	prednisolone (1 mg/kg/day)	N/A	best-corrected visual acuity remained stable (baseline: 1.28 (±0.71) vs. last follow-up: 1.46 (±0.60); *p* = 0.25). Average white Full-Field Stimulus Testing (FST) showed a trend toward improvement (baseline: −4.41 (±10.62) dB vs. last follow-up: −11.98 (±13.83) dB; *p* = 0.18).	Kiraly et al., 2023^55^
3	inherited retinal dystrophy (RPE-65 mediated)	VN	AAV2	1.5 × 10^11^ vg per eye	subretinal	*n* = 1	39 years	*n* = 1	*n* = 1	foveal ellipsoid zone loss	prednisolone (1 mg/kg/day)	N/A	the first eye showed improvement in rod photoreceptor function with increased peripheral and low luminance vision (baseline VA: 0.9 logMAR and 2-years post-operative VA: 0.7 logMAR. 2ND eye developed loss of foveal photoreceptors. FST improvements were maintained in both eyes. Macular oedema resolved by 6 weeks of VN	Jalil et al., 2023^136^
4	hemophilia B	etranacogene dezaparvovec	AAV5	2 × 10^13^ vg/kg	intravenous	*n* = 3	43, 47 and 50 years	*n* = 3	*n* = 3	headache transient elevation of C-reactive protein	N/A	N/A	stable durable FIX activity remained after 3 years discontinued FIX prophylaxis in all patients clinical phenotype from severe/moderately severe to mild/nonhemophilic 100% decrease in bleeds in 2/3 and 92% in patient 3	Von Drygalski et al., 2019^106^

GGT, gamma-glutamyl transferase, FIX, factor IX; N/A, not applicable; ULN, upper limit of normal.

### Immunosuppression-associated AEs

In addition to treatment-related AEs, we also evaluated the frequency of AEs associated with immunosuppressive protocols, including prophylactic and therapeutic immunosuppression, that were used in the clinical trials. Of the 38 clinical trials that were assessed in this review, only 8 studies (21%) reported having any AEs in patients after the administration of immunosuppressive drugs (Table 4). Three of the clinical trials resulting in immunosuppression-associated AEs were studies for hemophilia B gene therapy (however, a different AAV gene therapy was used in each case) and another two were for DMD, while studies for hemophilia A, MPS type IIIB, and SMA presented with immunosuppression-associated AEs in one clinical trial each. The immunosuppressive protocols administered to patients were in response to the following most common AEs observed: increased liver enzyme levels, headache, nausea, and fever. Although the AEs resolved after immunosuppressive treatment, a variety of AEs were reported after immunosuppression. This was more apparent in a clinical trial using valoctocogene roxaparvovec (AAV5-hFVIII-SQ; NCT03370913), where 71% of patients showed AEs to glucocorticoids or other immunosuppressants,^15^ and trials using verbrinacogene setparvovec (AAV2/S3) gene therapy (NCT03369444 and NCT03641703),^48^ where AEs were recorded in all patients after prednisolone or methylprednisolone treatment and accounted for 24% of all AEs observed.^48^ However, with the exception of clinical trial NCT03300453,^49^^,^^50^ the remaining clinical trials listed in Table 4 reported that AEs after prednisone treatment were only documented in up to two patients.^63^ The most common AEs observed in patients after immunosuppressive treatment were insomnia (NCT03370913,^57^ NCT04135300,^63^ NCT03369444, and NCT03641703^48^ and acne (NCT03370913,^57^ NCT04135300,^63^ and NCT00979238^43^^,^^44^). Of note, the authors of NCT03369444 and NCT03641703 stated that the AEs recorded in the patients were consistent with the known safety profiles of glucocorticoids and tacrolimus.^48^ In addition, one subject from clinical trials for DMD and SMA, respectively, presented with AEs after prednisone or prednisolone treatment. Clinical trial NCT03333590^64^ recorded a patient with a cushingoid face and weight gain, whereas a study by Friese et al.^65^ recorded a patient presenting with temporary arterial hypertension.

### AAV gene therapy and associated immunological responses

The use of AAV gene therapy in subjects with pre-existing immunity or memory response to gene therapy-related viruses could affect the efficacy and the safety of the treatment, constituting one of the major obstacles for gene therapy. Innate and adaptive immunity against the vector capsid or transgenic product may contribute, depending on the magnitude, by varying degrees to immune-mediated rejection and immunotoxicity.^6^

Independent of the AAV serotypes and administration route used during these clinical trials, a significant increase in NAbs was reported. Moreover, the presence of an immunosuppressant regimen during these clinical trials could not prevent the development of anti-AAV antibodies. An evaluation of the cellular immune response showed a significant increase in interferon (IFN)-γ antigen-specific T cells after treatment, and an elevation of inflammatory cytokines (e.g., IFN-γ, TNF-α) in serum was associated with vector reactogenicity.^2^^,^^10^^,^^13^^,^^44^^,^^50^^,^^61^^,^^62^^,^^64^^,^^66^^,^^67^^,^^68^^,^^69^^,^^70^^,^^71^^,^^72^^,^^73^^,^^74^^,^^75^^,^^76^^,^^77^ Interestingly, only two studies have reported Treg induction, although all patients developed high titers of binding IgG and NAbs.^63^^,^^72^

Currently, the European Medicines Agency^78^ and FDA^79^ require an evaluation of humoral immunity by determining both the titers and avidities of antibodies against the AAV transgene product during clinical development. For treatments that include redosing, a comprehensive evaluation of the cellular and humoral responses must be performed and documented with concurrent safety and efficacy data (see Immunogenicity).^80^ Ultimately, however, few studies have reported an evaluation of the titers as the reciprocal of the highest sample dilution that resulted in inhibition of 50% against the AAV vectors used or transgene products.^10^^,^^11^^,^^12^^,^^13^^,^^16^^,^^44^^,^^63^^,^^67^^,^^68^^,^^69^^,^^81^^,^^82^^,^^83^^,^^84^^,^^85^^,^^86^

Efforts to suppress the immune response to AAV or transgene product have not been consistent. The most common immunosuppressants, corticosteroids, are not always successful in inhibiting the immune response to AAV or attenuating liver toxicity, and there is no correlation with vector or dosing route used. However, mycophenolate mofetil in combination with tacrolimus has provided a safety profile after a long-term follow-up in all children treated in NCT03300453.^49^^,^^50^

## Discussion

The results of this review provide valuable insights into the immunosuppressive protocols and immunological responses associated with various gene therapy treatments. The immune response to AAV gene therapy is complex and can be triggered by vector capsids, genomes, and transgene protein products.^16^ Immune responses to AAV are ubiquitous and have been seen across various disease states, routes of administration, and capsid serotypes.^21^^,^^72^ The route of administration directly influences the vector dose and immunosuppressive protocols. For example, subretinal AAV vector administration performed in several clinical studies of gene transfer for RPE65 deficiency^7^^,^^9^ was generally associated with little to no detectable immune response to the capsid or the transgene.

AEs were observed in 30 clinical trials, with the most frequent being increased liver enzyme levels, vomiting and nausea, fever, and fatigue. SAEs were observed in eight studies, with SMA and hemophilia B being the main diseases associated with SAEs. Corticosteroids, including prednisolone, were used to manage these events, even in studies where corticosteroids were administered prophylactically. Interestingly, a phase 1/2 clinical trial for hemophilia B gene therapy did not find a clear association between the resolution of elevated liver enzyme levels and prednisolone use.^59^

To decrease AAV immunogenicity, one focus is on decreasing different aspects of immune responses against the capsid to improve patient safety profile and safeguard long-term transgene expression.^73^ The use of less seroprevalent capsids may reduce the recognition of preformed NAbs.^73^ One particularly promising strategy is capsid engineering, which has the potential to develop next-generation vectors with multiple improvements compared to current vectors. Current AAV vectors still face limitations in delivering efficiently to specific tissues, often requiring high doses. Higher vector doses have been associated with hepatotoxicity, TMA, and other immune-mediated AEs.^73^ Additionally, enhancing tropism and transduction efficiency alone would allow for lower doses and significantly decrease toxicity from AAV immunity.^21^

One important obstacle is neutralizing anti-AAV antibodies that display a major short- and long-term barrier in delivering gene therapy. Humoral responses against AAV capsid proteins are evaluated in patients before AAV treatment in some trials, and patients with detectable pre-existing antibodies to AAV are excluded. Rates of NAb prevalence in the general population are quoted as 30%–70% in literature against all various serotypes, with the highest prevalence against AAV2, followed by AAV1.^36^^,^^75^^,^^76^ In addition, patients who develop *de novo* antibodies after systemic AAV gene therapy have no access to eventual redosing attempts in case of loss of therapy efficacy. Even though gene therapies are designed to be administered only once, the immune response is one factor that jeopardizes the long-term efficacy of transgene expression, which highlights the need for potential redosing strategies for patients. For example, in a recently approved therapy for hemophilia A (Roctavian), factor VIII activity levels declined over 3 years from 52.6 to 18.2 IU/dL. Interestingly, 79% of these 134 patients received corticosteroids in response to ALT elevations. Despite the introduction of corticosteroids, an overall decrease in factor VIII levels was present.^87^ Not only do immune responses prevent the repeated administration of AAV, but AAV immunity can lead to severe toxicity and SAEs, potentially resulting in patient death.^21^

To enable redosing, pre-clinical work (including plasmapheresis monoclonal antibody directed to plasma cells and imlifidase injection) has been conducted to evaluate how animals with pre-existing AAV antibodies could be successfully and safely transfected with a transgene carried by an AAV vector.^88^^,^^89^ In the version of a potential treatment with a second dose of gene therapy, protocols have been developed to tamper the immune reaction during the infusion of the first dose, such as co-administration of sirolimus nanoparticles.^73^^,^^74^^,^^75^^,^^76^^,^^77^^,^^87^^,^^88^^,^^89^^,^^90^ Research findings have shown that incorporating sirolimus into synthetic vaccine particles, co-administered with AAV vectors, hinders the induction of cell-mediated and anti-capsid humoral responses. This results in the inhibition of CD8+ T cell infiltration in the liver, reduction of B and T cell activation, and suppression of memory T cell response in both mice and non-human primates.^88^ To our best knowledge, this has not yet been translated *in vivo* in humans.

In this review, a variety of immunosuppressive treatments were noted, including different corticosteroids, tacrolimus, mycophenolate mofetil, cyclosporine, sirolimus, and rituximab. Corticosteroids were the most widely used immunosuppressants for AAV gene therapy clinical studies (Table 3), either alone or in combination with other drugs, for their global inhibitory effects on innate and adaptive immunity. Corticosteroids are used as immunosuppressants for approved gene therapy products^91^ (VN^8^ and OA^11^), due to their established safety profiles and effects in preventing or alleviating immune responses. However, the drawbacks of corticosteroids are immunosuppression-associated side effects and non-specific immunosuppression, in particular increased susceptibility to infection.^92^^,^^93^ Therefore, careful monitoring and appropriate prophylactic measures should be taken to minimize the risk of infections in patients receiving immunosuppressive treatments. The doses of prednisolone seen in this review ranged from 1 to 2 mg/kg with a total daily dose in the range of 30–60 mg/day. The duration of corticosteroid treatment ranged dramatically between 7 and 133 days, with other immunosuppressants being used for up to 365 days. This highlights the disparity between studies on the longevity of immunosuppression and differing opinions of where the balance of reducing AAV gene therapy immunogenicity versus the side effects of long-term immunosuppression should be struck. Work is ongoing to refine the immunosuppression regimen with the goal of decreasing vector-related immune response in the early period after treatment. For example, Chowdary et al.^48^ adopted a prophylactic immunosuppression regimen to improve the predictability of the dose-response and to increase the chances that normal factor IX levels would be reached and maintained in patients with hemophilia B.

In a review from Oh et al.^93^ investigating steroid-associated side effects in patients with primary proteinuric kidney disease, 62% of patients exposed to steroids developed one SAE. The rate of hypertension was 1.4 times higher than before steroid exposure. Interestingly, the risk of metabolic complications (e.g., diabetes mellitus, overweight, obesity) was also significantly higher after steroid therapy exposure. Taken together, a patient’s adjusted risk of these complications following steroid exposure was 1.5–1.8 times that of the risk before exposure. Other studies, such as Movahedi et al., which analyzed UK and US national databases for nearly 22,000 patients with rheumatoid arthritis, showed that the hazard ratio of steroid-associated diabetes was 1.30 and 1.61, respectively.^94^ Another small Japanese study showed corticosteroid-induced diabetes developed in 17–42 patients (40.5%) undergoing steroid therapy for primary renal disease.^95^ In our analysis, often the duration and the dosing regimen of steroid exposure was not always mentioned, and the overall sample size of patients undergoing gene therapy is rather small. In the hemophilia study with valoctocogene roxaparvovec, which has the highest number of patients, the authors reported similar AE rates comparable with the aforementioned publications. In summary, steroid use is related to side effects in gene therapy. Due to the given limitations, the full spectrum and rate of AEs in the context of gene therapy cannot be retrieved based on the current available data, but the available data is in keeping with known side effect profiles seen in high-dose steroid use in other conditions.

Although a wide range of immunosuppressive agents were identified in the study, there is insufficient data available to determine if any of these conferred an additional safety benefit. Only one-quarter of studies reported antibody titers against the vector and transgene, and even fewer evaluated other immune cell responses, e.g., Tregs. Different immunosuppressive drugs have varying modes of action, for example, corticosteroids are non-specific,^91^ mycophenolate motefil depletes both T and B cell populations,^50^^,^^93^ sirolimus inhibits T and B cell activation and induces Tregs through targeting mTOR,^93^ whereas rituximab is an anti-CD20 monoclonal antibody that depletes B cells by inducing apoptosis.^96^ Therefore, better characterization of the different immune cell responses in the future would help identify if any of these agents are more efficacious in decreasing gene therapy immunogenicity and would also identify if additional cell populations need targeting. No agent identified in these studies specifically targeted innate immune responses, although this could be an important direction for the future. As well as engineering transgene to lower their immunogenic CpG content,^97^ there are now C3 inhibitors, e.g., APL-9, which have been used during AAV delivery with the aim of dampening the complement pathway.^98^ Some studies already used a combination of immunosuppressive agents to broaden the cell populations they covered; however, with this, the increased likelihood of side effects must be considered. Balancing the risk-benefit profile of immunosuppression continues to be a great challenge for the gene therapy field going forward. Recent prospective trials tested the combination of mTOR inhibitor sirolimus in combination with rituximab.^3^^,^^99^ Preclinical hemophilia A mice models showed that under this combined immunosuppressive therapy, there was no increase in inhibitors following rechallenging with factor VIII protein.^100^ The combined therapy with rituximab and sirolimus was also used in Pompe disease and GM2-gangliosidosis to enable re-administration of AAV.^3^^,^^101^ Despite this first promising data, this drug combination must show its benefit in future trials for patients without sacrificing patient safety.

**Figure 2:**
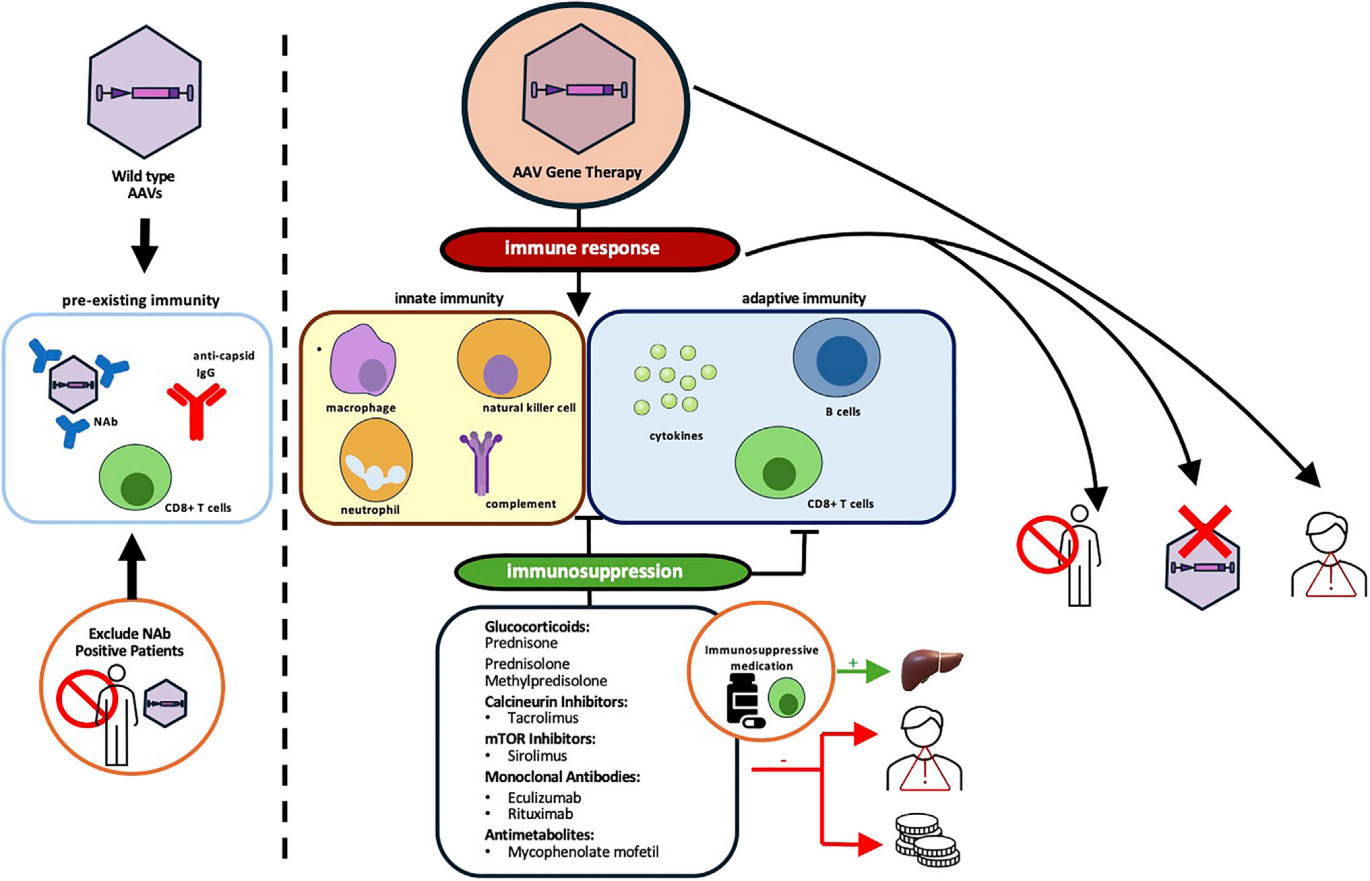
Graphic summary of findings AAV-based gene therapy treatment can induce pre-existing immunity, innate immunity, and adaptive immune response against AAV vectors and transgenic products, inhibiting AAV expression. Patients with pre-existing immunity are unable to receive AAV-based gene therapy treatment. Immunosuppression protocols are used to suppress the immune response and elevate treatment-related AEs, such as the normalization of liver enzymes. Immunosuppression, although potentially essential for some patients to ensure therapeutic benefits of AAV-mediate gene transfer, comes at high costs.

Gene therapy using AAV vectors shows promise for treating rare genetic diseases. However, the high costs and risks associated with developing these therapies must be considered in the real-world deployment of these therapies (Figure 2). Conducting robust clinical trials in rare diseases can present complexity and challenges but are necessary to assess the risks and benefits. Improving safety, immunosuppressive regimens, patient risk assessment, and comparing gene therapy to other treatments are key factors to consider.

### Conclusion

This review provides valuable information on the immunosuppressive protocols used alongside various gene therapy treatments. Understanding and minimizing immunological responses is crucial for optimizing treatment protocols and ensuring the safety and efficacy of gene therapy interventions. Further research and adherence to evaluation recommendations will contribute to the development of more effective and safer gene therapy approaches in the future.

## Methods

**Figure 3:**
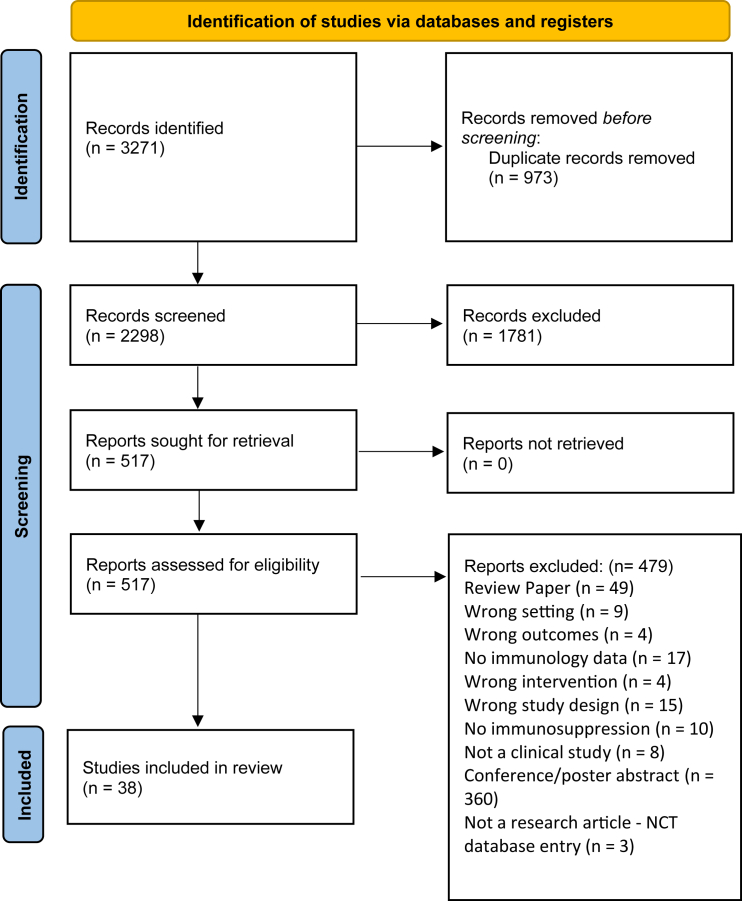
PRISMA flow of the selection process of clinical trials

An information specialist (E.H.) searched the following bibliographic databases on May 3, 2024, for studies published from database inception to the search date: Ovid Embase, Ovid MEDLINE, and the Cochrane Register of Controlled Trials. We searched using terms related to monogenic disorders, gene therapy, cell therapy with genetically modified cells, immune modulation, and immune suppression, limited to clinical trial records only. The search strategies for Ovid Embase and Ovid MEDLINE used adapted versions of the Cochrane Highly Sensitive Search Strategies for identifying controlled trials. The full strategies are available in the appendix. All references were exported to EndNote X21 (Thomson Reuters, New York, NY, USA), and duplicates were removed manually. Covidence software was used to manage screening and eligibility. A primary search of the indicated electronic databases resulted in a total of 3,271 articles (Figure 3). After the removal of duplicates, the remaining studies were screened based on their titles and abstracts by two independent researchers. A total of 517 articles remained after this screening stage and were then evaluated based on their full texts, resulting in 38 clinical trial studies in the final meta-analysis. We excluded literature reviews and conference articles.

**Figure 4:**
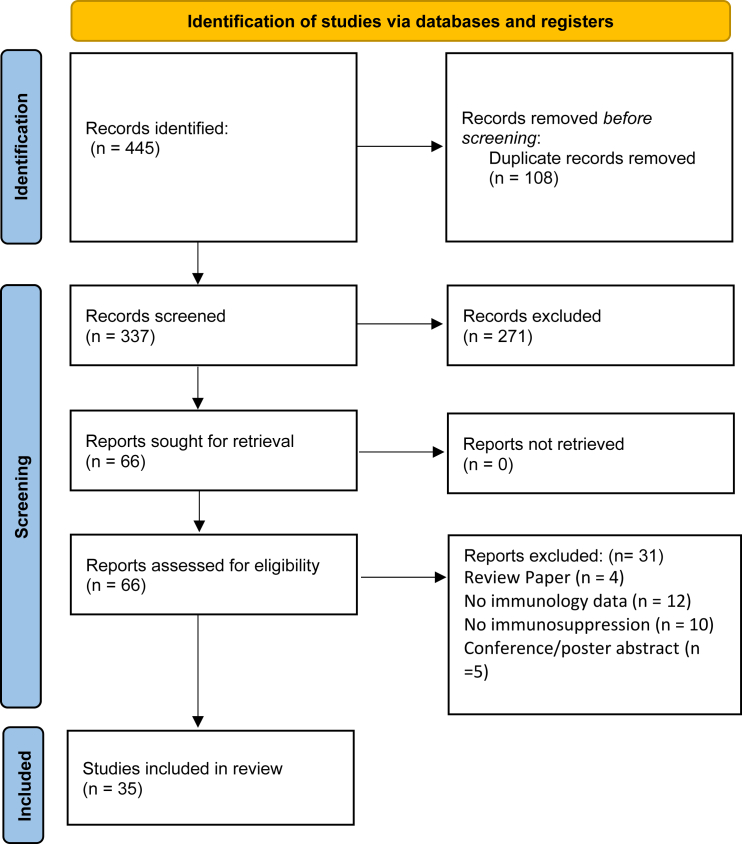
PRISMA flow of the selection process of real-world studies

In addition, 445 real-world studies, case reports, or observational studies were found through a systematic search in the same three databases and registry on May 3, 2024, for studies published from database inception to the search date. We searched using terms related to monogenic disorders, gene therapy, cell therapy with genetically modified cells, immune modulation, and immune suppression, limited to observational studies, case reports, and real-world studies. Conference abstracts and posters were included from Ovid Embase. Again, the full strategies are available in the Appendix. All references were exported to EndNote 21 (Thomson Reuters), and duplicates were removed manually. As shown in the PRISMA flow diagram^138^ (Figure 4), 337 articles were screened after duplications were eliminated. A total of 35 real-world studies were included in the meta-analysis.

## Supplemental information

Document S1. Tables S1 and S2

Document S2. Article plus supplemental information
