# Supplementary material for: A systematic review of immunosuppressive protocols used in AAV gene therapy for monogenic disorders
Source: Mol Ther. 2024 Jul 22;32(10):3220–59. doi: 10.1016/j.ymthe.2024.07.016 (PMC11489562; doi:10.1016/j.ymthe.2024.07.016)
Supplement: Document S1. Tables S1 and S2 [file mmc1.pdf]

YMTHE, Volume *32*

Supplemental Information

A systematic review of immunosuppressive protocols used in AAV gene therapy for monogenic disorders

Besarte Vrellaku, Ilda Sethw Hassan, Rebecca Howitt, Christopher P. Webster, Eli Harriss, Fraser McBlane, Corinne Betts, Jorge Schettini, Mattia Lion, John E. Mindur, Michael Duerr, Pamela J. Shaw, Janine Kirby, Mimoun Azzouz, and Laurent Servais

Table S1: Search Results for immunogenicity and immune monitoring from clinical trials.

	Search results on 15/11/2021	Search results on 29/03/2023	Search results on 05/03/2024
Ovid Embase	1837	2243	2586
Ovid Medline	406	480	536
Cochrane CENTRAL	48	59	728
Total	2291	2782	3850
Total after deduplication	1923	2302	-
Unique since 15/11/2021	-	396	-
Unique since 29/03/2023	-	-	**952**

Database: Embase 1974 to present

Search Strategy:
1 exp gene therapy/ (102165)
2 targeted gene repair/ (445)
3 ("DNA therap*" or (gene* adj2 therap*) or "gene* transfer*" or transgene or "gene replacement*" or "gene-edit*" or "gene silencing" or "gene repair*" or "gene correction*").ti,ab. (212907)
4 1 or 2 or 3 (251960)
5 (Randomized controlled trial/ or Controlled clinical study/ or random$.ti,ab. or randomization/ or intermethod comparison/ or placebo.ti,ab. or (compare or compared or comparison).ti. or ((evaluated or evaluate or evaluating or assessed or assess) and (compare or compared or comparing or comparison)).ab. or (open adj label).ti,ab. or ((double or single or doubly or singly) adj (blind or blinded or blindly)).ti,ab. or double blind procedure/ or parallel group$1.ti,ab. or (crossover or cross over).ti,ab. or ((assign$ or match or matched or allocation) adj5 (alternate or group$1 or intervention$1 or patient$1 or subject$1 or participant$1)).ti,ab. or (assigned or allocated).ti,ab. or (controlled adj7 (study or design or trial)).ti,ab. or (volunteer or volunteers).ti,ab. or human experiment/ or trial.ti.) not (((random$ adj sampl$ adj7 ("cross section$" or questionnaire$1 or survey$ or database$1)).ti,ab. not (comparative study/ or controlled study/ or randomi?ed controlled.ti,ab. or randomly assigned.ti,ab.)) or (Cross-sectional study/ not (randomized controlled trial/ or controlled clinical study/ or controlled study/ or randomi?ed controlled.ti,ab. or control group$1.ti,ab.)) or (((case adj control$) and random$) not randomi?ed controlled).ti,ab. or (Systematic review not (trial or study)).ti. or (nonrandom$ not random$).ti,ab. or "Random field$".ti,ab. or (random cluster adj3 sampl$).ti,ab. or ((review.ab. and review.pt.) not trial.ti.) or ("we searched".ab. and (review.ti. or review.pt.)) or "update review".ab. or (databases adj4 searched).ab.) (6133950)
6 clinical trial/ or phase 1 clinical trial/ or phase 2 clinical trial/ or phase 3 clinical trial/ (1222173)
7 ("clinical trial*" or "phase i" or "phase 1" or "phase ii" or "phase 2" or "phase iii" or "phase 3" or "interventional trial*" or "paediatric trial*" or "pediatric trial*" or "pivotal trial*" or "open label*" or "orphan drug*" or "rare disease*").ti,ab. or rare disease/ or orphan drug/ (1137498)
8 5 or 6 or 7 (7071304)

9 epidural drug administration/ or intracisternal drug administration/ or intrathecal drug administration/ (27063)
10 intraocular drug administration/ or intracorneal drug administration/ or intravitreal drug administration/ or subretinal drug administration/ (10616)
11 exp intraspinal drug administration/ (29464)
12 exp intraocular drug administration/ (12131)
13 drug infusion/ (17165)
14 exp intracerebral drug administration/ (31720)
15 intravenous drug administration/ (384430)
16 (Intravenous* or intra-venous* or IV or systemic or infusion* or intramuscular* or intra-muscular* or IM or intrathecal* or intra-thecal* or "cerebrospinal fluid*" or CSF or intra-CSF or epidural* or spinal* or ophthalmol* or subretinal* or sub-retinal* or intravitreal* or intra-vitreal* or intraorbital* or intra-orbital* or intracerebroventricular* or intra-cerebroventricular* or ICV or "i.c.v. injection*" or ICVI or intracerebral* or intra-cerebral* or IC or intraventricular* or intra-ventricular* or intraparenchyma* or intra-parenchyma* or "intra-cisterna magna" or ICM).ti,ab. (3195173)
17 9 or 10 or 11 or 12 or 13 or 14 or 15 or 16 (3407200)
18 rare disease/ (50265)
19 orphan drug/ (4013)
20 exp spinal muscular atrophy/ (71765)
21 centronuclear myopathy/ (1323)
22 myotonic dystrophy/ (9744)
23 exp muscular dystrophy/ (52657)
24 spastic paraplegia/ (5101)
25 exp gangliosidosis/ (4390)
26 multiple sclerosis/ (161878)
27 parkinson disease/ or autosomal dominant parkinson disease/ (196772)
28 Alzheimer disease/ (257013)
29 juvenile neuronal ceroid lipofuscinosis/ (295)
30 giant axonal neuropathy/ (320)
31 hereditary motor sensory neuropathy/ (13412)
32 exp hemophilia/ (46582)
33 exp sickle cell anemia/ (47775)
34 glycogen storage disease type 2/ (5209)
35 Canavan disease/ (818)
36 Fabry disease/ (9593)
37 exp mucopolysaccharidosis/ (14039)
38 familial hypercholesterolemia/ (12739)
39 exp congestive cardiomyopathy/ (55227)
40 cystic fibrosis/ (86136)
41 alpha 1 antitrypsin deficiency/ (6642)
42 ciliary dyskinesia/ (3703)
43 exp macular degeneration/ (27716)
44 Leber congenital amaurosis/ (2306)
45 exp monogenic disorder/ (573870)
46 ("rare disease*" or "orphan drug*" or "monogenic disease*" or "monogenic disorder*" or "spinal muscular atrophy" or "spine muscle atrophy" or "myotubular myopathy" or "centronuclear myopathy" or "myotonic dystrophy" or "curschmann steinert disease" or "deleage disease" or "dystrophic myotonica" or "dystrophic myotonia" or "myotonia atrophica" or "myotonia dystrophica" or "myotonic atrophia" or "myotonic atrophy" or

"myotonic dystrophia" or "myotonic muscular dystrophy" or "myotonic muscular dystrophy" or "proximal myotonic myopathy" or "steinert disease" or "steinert myopathy" or "muscular dystrophy" or "muscle dystrophy" or "muscle dystrophia" or "motor neuron* disease" or MND or "Spastic paraplegia" or "spastic paraparesis" or gangliosidosis or "ganglioside lipidosis" or gangliosidoses or "multiple sclerosis" or "chariot disease" or "disseminated sclerosis" or "insular sclerosis" or MS or Parkinson* or "paralysis agitans" or Alzheimer* or Alzeimer* or "diffuse cortical sclerosis" or Batten* or "juvenile neuronal ceroid lipofuscinosis" or "Giant axonal neuropathy" or GAN or GAN-1 or "Amyotrophic lateral sclerosis" or ALS or "lou gehrig*" or Charcot-Marie-Tooth or "hereditary motor sensory neuropathy" or "charcot-marie" or "dejerine sottas" or "familial spastic para*" or "hereditary spastic para*" or Struempell or strumpel* or SCID-X or Haemophili* or hemophili* or "sickle cell" or drepanocyt* or "haemoglobin ss" or "hemoglobin ss" or "sickle anaemia" or "sickle anemia" or Pompe or "glycogen storage disease type 2" or "cardiomuscular glycogenosis" or "diffuse glycogenosis" or "glycogen storage disease type ii" or (glycogenos* adj2 (ii or targeted gene repair)) or Canavan* or "AADC deficien*" or Fabry* or "alpha galactosidase deficiency syndrome" or Mucopolysaccharidos* or "familial hypercholesterol*" or "buerger gruetz" or "burger grutz" or "familial hyperbetalipoprotein*" or "amilial hypercholester*" or "dilated cardiomyopathy" or "congestic cardiomyopathy" or "congestive heart disease" or "congestive myocardiopathy" or "cystic fibrosis" or "cystic pancreas fibrosis" or "fibrocystic disease" or "Alpha-1 antitrypsin deficiency" or "alpha 1 proteinase inhibitor deficiency" or "alpha1 antitrypsin deficiency" or "antitrypsin alpha 1 deficiency" or "ciliary dyskinesia" or "ciliary immotility" or "ciliatry motility disorders" or ciliostasis or "macula* degeneration" or "macula* atrophy" or "macular dystrophy" or LCA or Leber).ti,ab. (1546055)
47 exp retina dystrophy/ (5296)
48 retinitis pigmentosa/ (13362)
49 color blindness/ (2021)
50 Duchenne muscular dystrophy/ (20330)
51 Becker muscular dystrophy/ (3590)
52 sarcoglycanopathy/ (348)
53 Friedreich ataxia/ (5028)
54 metachromatic leukodystrophy/ (2429)
55 adenylosuccinate lyase/ (454)
56 happy puppet syndrome/ (3332)
57 Werdnig Hoffmann disease/ (1410)
58 spinal muscular atrophy type 2/ (635)
59 Kugelberg Welander disease/ (714)
60 limb girdle muscular dystrophy/ (4278)
61 GM1 gangliosidosis/ (1100)
62 aromatic levo amino acid decarboxylase/ (3428)
63 globoid cell leukodystrophy/ (2158)
64 Rett syndrome/ (6379)
65 (LCA* or SMA or DMD or XMTM or LGMD or CMT or PD or MPS or Ho-FH or DCM or CF or AATD or PCD).ti,ab. (548959)
66 ("retinal dystroph*" or "Retinitis Pigmentosa" or RP or "retinal pigment* dystroph*" or "tapeto retinal degeneration" or "tapetoretinal degeneration" or "tapetoretinal dystrophy").ti,ab. (79169)
67 (achromatognosia or achromatopia or achromatopsia or "colour blind*" or "color blind*" or "color agnosia" or "colour agnosia" or "scoterythrous vision" or "duchenne syndrome" or "duchenne type muscular dystrophy" or "duchenne muscular dystrophy" or "morbus

duchenne" or "pseudo hypertrophic myopathic progressive muscular dystrophy").ti,ab. (18435)
68 ("backer muscular dystrophy" or "becker muscular dystrophy" or "dystrophic becker disease" or "becker dystrophy" or "becker type muscular dystrophy").ti,ab. (2569)
69 (sarcoglycanopath* or Friedreich* or "hereditary spinal ataxia" or "spinal hereditary ataxia" or "spinal heredoataxia").ti,ab. (4744)
70 ("cerebroside sulfatase deficiency syndrome" or "cerebroside sulfate storage disease" or "cerebroside sulphate storage disease" or "metachrom* leucodystrophy" or "metachrom* leukodystrophy" or "sulfatide lipidosis" or sulfatidosis or "sulphatide lipidosis").ti,ab. (1839)
71 ("adenylosuccinate lyase" or adenylosuccinase or "adenylosuccinate amp lyase" or "adenylsuccinate lyase" or "e.c. 4.3.2.2" or "succinyladenylate lyase").ti,ab. (381)
72 ("Angelman syndrome" or "happy puppet syndrome").ti,ab. (2211)
73 ("hereditary progressive spinal muscular atrophy" or "hereditary spinal progressive muscular atrophy" or "hoffmann werdnig" or "werdnig hoffman" or ((infantile or "type 1" or "type I") adj3 "musc* atrophy")).ti,ab. (1090)
74 (Dubowitz or (("type 2" or "type II") adj3 "musc* atrophy")).ti,ab. (702)
75 (kugelberg* or ((benign or juvenile or "type 3" or "type III") adj3 "musc* atrophy")).ti,ab. (584)
76 ("limb girdle dystroph*" or "limb-girdle muscular dystroph*").ti,ab. (3733)
77 ("GM1 gangliosidosis" or "gangliosidosis G(M1)" or "gangliosidosis gm 1" or "gangliosidosis GM1" or "gm 1 gangliosidosis").ti,ab. (924)
78 ("Aromatic l-amino acid decarboxylase" or "3,4 dihydroxy l phenylalanine carboxylyase" or "3,4 dihydroxyphenylalanine decarboxylase" or "5 hydroxy levo tryptophan carboxylyase" or "5 hydroxytrypthophan decarboxylase" or "5 hydroxytryptophan carboxylyase" or "5 hydroxytryptophan decarboxylase" or "5 hydroxytryptophane decarboxylase" or "aromatic amino acid decarboxylase*" or "aromatic aminoacid decarboxylase*" or "aromatic l amino acid carboxy lyase" or "aromatic l amino acid decarboxylase*" or "aromatic l aminoacid decarboxylase" or "aromatic levo aminoacid decarboxylase" or "dihydroxyphenylalanine decarboxylase" or "DOPA decarboxylase" or "e.c. 4.1.1.28" or "hydroxytryptophan decarboxylase" or "l 3,4 dihydroxyphenylalanine decarboxylase" or "l dopa decarboxylase" or "l tryptophan carboxylyase" or "levo dopa decarboxylase" or "levodopa decarboxylase" or "tryptophan decarboxylase").ti,ab. (4204)
79 (krabbe or "beta galactosidase deficiency syndrome" or "galactosylceramidase deficiency syndrome" or "galactosylceramide lipidosis" or "globoid cell dystrophy" or "globoid cell leucodystrophy" or "globoid cell leukodystrophy" or "mckusick 24520").ti,ab. (1625)
80 (rett or rett's).ti,ab. (5514)
81 18 or 19 or 20 or 21 or 22 or 23 or 24 or 25 or 26 or 27 or 28 or 29 or 30 or 31 or 32 or 33 or 34 or 35 or 36 or 37 or 38 or 39 or 40 or 41 or 42 or 43 or 44 or 45 or 46 or 47 or 48 or 49 or 50 or 51 or 52 or 53 or 54 or 55 or 56 or 57 or 58 or 59 or 60 or 61 or 62 or 63 or 64 or 65 or 66 or 67 or 68 or 69 or 70 or 71 or 72 or 73 or 74 or 75 or 76 or 77 or 78 or 79 or 80 (2541161)
82 exp pharmacovigilance/ (23261)
83 (Pharmacovigilan* or pharmaco-vigilan* or toxicit* or SUSAR or SAE or AR or SAR or "adverse reaction*" or "adverse event*" or antibodies or immunosuppress* or immunotherap* or "immune management" or immunomodulat* or "subclinical immune response*" or immunologic* or aminotransaminase* or ((liver or hepatic) adj2 (damag* or injur* or inflamm*)) or leukemia or leukaemia or cancer* or tumor* or tumour* or humoral or "cellular immunity" or "innate immunity" or immunogenicity or "complement activation" or cytokine* or "pre-existing immunity" or ADA or cell-mediated or binding-antibod* or "induced response*" or pre-existing or preexisting or boosted or "capsid response*" or

redosing or "cell-mediated immun*" or "T-cell immunity" or tolerance or tolerization or TNF-alpha or prednisolone or eculizumab or "complement activation" or transaminitis or hypertransaminasemia or thrombocytopenia or microangiopathy).ti,ab. (8022126)
84 exp immunosuppressive treatment/ (260883)
85 exp leukemia/ (378133)
86 exp neoplasm/ (5678672)
87 exp liver injury/ (81741)
88 exp immunogenicity/ (89304)
89 exp cytokine/ (1961953)
90 prednisolone/ (153065)
91 eculizumab/ (9820)
92 exp thrombocytopenia/ (232160)
93 exp microangiopathy/ or hypertransaminasemia/ (79139)
94 spinal ganglion/ (26536)
95 exp hemolytic anemia/ (140088)
96 kidney injury/ or acute kidney failure/ (175604)
97 exp liver toxicity/ (103332)
98 (TMA or "dorsal root ganglion" or "dorsal root spinal ganglion" or "spinal ganglia" or "spine ganglion" or DRG or "hemolytic anemia" or "hemolytic anaemia" or "haemolytic anemia" or "haemolytic anaemia" or "acute kidney damage" or "acute kidney injury" or "acute kidney failure" or "acute renal damage" or "acute renal failure" or "elevated liver enzyme*" or "elevated hepatic enzyme*" or hepatotoxic* or "hepato toxic*" or "liver cell toxicit*" or "liver intoxication" or "liver poison*" or "liver toxic*" or "abnormal T2 hyperintensit*").ti,ab. (222334)
99 82 or 83 or 84 or 85 or 86 or 87 or 88 or 89 or 90 or 91 or 92 or 93 or 94 or 95 or 96 or 97 or 98 (10809812)
100 4 and 8 and 17 and 81 and 99 (2586)

This search strategy uses an adapted version of the RCT filter for Ovid MEDLINE from the Cochrane Highly Sensitive Search Strategy for identifying controlled trials in MEDLINE, courtesy of: https://libraryguides.mcgill.ca/epib629/rct-filters#s-lg-box-wrapper-13740118
Source: Box 3.c., Technical Supplement to Chapter 4: Searching for and Selecting Studies. Cochrane Handbook for Systematic Reviews of Interventions Version 6.

Database: Medline (Ovid MEDLINE® Epub Ahead of Print, In-Process & Other Non-Indexed Citations, Ovid MEDLINE® Daily and Ovid MEDLINE®) 1946 to present
Search Strategy:
1 exp Genetic Therapy/ (54409)
2 ("DNA therap*" or (gene* adj2 therap*) or "gene* transfer*" or transgene or "gene replacement*" or "gene-edit*" or "gene silencing" or "gene repair*" or "gene correction*").ti,ab. (162791)
3 1 or 2 (181624)
4 ((randomized controlled trial or controlled clinical trial).pt. or randomized.ab. or randomised.ab. or placebo.ab. or drug therapy.fs. or randomly.ab. or trial.ab. or groups.ab.) not (exp animals/ not humans.sh.) (5177269)
5 clinical trial/ or clinical trial, phase i/ or clinical trial, phase ii/ or clinical trial, phase iii/ (602002)

6 ("clinical trial*" or "phase i" or "phase 1" or "phase ii" or "phase 2" or "phase iii" or "phase 3" or "interventional trial*" or "paediatric trial*" or "pediatric trial*" or "pivotal trial*" or "open label*" or "orphan drug*" or "rare disease*").ti,ab. (709665)
7 Rare Diseases/ (14380)
8 Orphan Drug Production/ (1465)
9 4 or 5 or 6 or 7 or 8 (5540535)
10 Epidural Space/ (4886)
11 exp administration, intravenous/ or administration, ophthalmic/ (150896)
12 exp injections, intraocular/ or injections, intravenous/ or exp injections, spinal/ (110553)
13 (Intravenous* or intra-venous* or IV or systemic or infusion* or intramuscular* or intra-muscular* or IM or intrathecal* or intra-thecal* or "cerebrospinal fluid*" or CSF or intra-CSF or epidural* or spinal* or ophthalmol* or subretinal* or sub-retinal* or intravitreal* or intra-vitreal* or intraorbital* or intra-orbital* or intracerebroventricular* or intra-cerebroventricular* or ICV or "i.c.v. injection*" or ICVI or intracerebral* or intra-cerebral* or IC or intraventricular* or intra-ventricular* or intraparenchyma* or intra-parenchyma* or "intra-cisterna magna" or ICM).ti,ab. (2316440)
14 10 or 11 or 12 or 13 (2362480)
15 Rare Diseases/ (14380)
16 Orphan Drug Production/ (1465)
17 exp Muscular Atrophy, Spinal/ (6639)
18 exp Myopathies, Structural, Congenital/ (1624)
19 exp Muscular Dystrophies/ (30356)
20 exp Paraplegia/ (13603)
21 exp Gangliosidoses/ (2695)
22 exp Multiple Sclerosis/ (71739)
23 Parkinson Disease/ (84852)
24 Alzheimer Disease/ (123730)
25 Neuronal Ceroid-Lipofuscinoses/ (2273)
26 exp "Hereditary Sensory and Motor Neuropathy"/ (8261)
27 hemophilia a/ or hemophilia b/ (25211)
28 exp Anemia, Sickle Cell/ (26145)
29 Glycogen Storage Disease Type II/ (1995)
30 Canavan Disease/ (313)
31 Fabry Disease/ (4224)
32 exp Mucopolysaccharidoses/ (7094)
33 Hyperlipoproteinemia Type II/ (7796)
34 Cardiomyopathy, Dilated/ (17408)
35 Cystic Fibrosis/ (40582)
36 alpha 1-Antitrypsin Deficiency/ (3789)
37 exp Ciliary Motility Disorders/ (2699)
38 exp Macular Degeneration/ (31035)
39 Leber Congenital Amaurosis/ (586)
40 ("rare disease*" or "orphan drug*" or "monogenic disease*" or "monogenic disorder*" or "spinal muscular atrophy" or "spine muscle atrophy" or "myotubular myopathy" or "centronuclear myopathy" or "myotonic dystrophy" or "curschmann steinert disease" or "deleage disease" or "dystrophic myotonica" or "dystrophic myotonia" or "myotonia

atrophica" or "myotonia dystrophica" or "myotonic atrophia" or "myotonic atrophy" or "myotonic dystrophia" or "myotonic muscular dystrophy" or "myotonic muscular dystrophy" or "proximal myotonic myopathy" or "steinert disease" or "steinert myopathy" or "muscular dystrophy" or "muscle dystrophy" or "muscle dystrophia" or "motor neuron* disease" or MND or "Spastic paraplegia" or "spastic paraparesis" or gangliosidosis or "ganglioside lipidosis" or gangliosidoses or "multiple sclerosis" or "chariot disease" or "disseminated sclerosis" or "insular sclerosis" or MS or Parkinson* or "paralysis agitans" or Alzheimer* or Alzeimer* or "diffuse cortical sclerosis" or Batten* or "juvenile neuronal ceroid lipofuscinosis" or "Giant axonal neuropathy" or GAN or GAN-1 or "Amyotrophic lateral sclerosis" or ALS or "lou gehrig*" or Charcot-Marie-Tooth or "hereditary motor sensory neuropathy" or "charcot-marie" or "dejerine sottas" or "familial spastic para*" or "hereditary spastic para*" or Struempell or strumpel* or SCID-X or Haemophili* or hemophili* or "sickle cell" or drepanocyt* or "haemoglobin ss" or "hemoglobin ss" or "sickle anaemia" or "sickle anemia" or Pompe or "glycogen storage disease type 2" or "cardiomuscular glycogenosis" or "diffuse glycogenosis" or "glycogen storage disease type ii" or (glycogenos* adj2 (ii or targeted gene repair)) or Canavan* or "AADC deficien*" or Fabry* or "alpha galactosidase deficiency syndrome" or Mucopolysaccharidos* or "familial hypercholesterol*" or "buerger gruetz" or "burger grutz" or "familial hyperbetalipoprotein*" or "amilial hypercholester*" or "dilated cardiomyopathy" or "congestic cardiomyopathy" or "congestive heart disease" or "congestive myocardiopathy" or "cystic fibrosis" or "cystic pancreas fibrosis" or "fibrocystic disease" or "Alpha-1 antitrypsin deficiency" or "alpha 1 proteinase inhibitor deficiency" or "alpha1 antitrypsin deficiency" or "antitrypsin alpha 1 deficiency" or "ciliary dyskinesia" or "ciliary immotility" or "ciliatry motility disorders" or ciliostasis or "macula* degeneration" or "macula* atrophy" or "macular dystrophy" or LCA or Leber).ti,ab. (1119488)
41 exp Retinal Dystrophies/ (10905)
42 exp Retinitis Pigmentosa/ (10845)
43 Color Vision Defects/ (4266)
44 Friedreich Ataxia/ (2824)
45 Leukodystrophy, Metachromatic/ (1330)
46 Adenylosuccinate Lyase/ (267)
47 Angelman Syndrome/ (1406)
48 Leukodystrophy, Globoid Cell/ (1091)
49 Rett Syndrome/ (3003)
50 (LCA* or SMA or DMD or XMTM or LGMD or CMT or PD or MPS or Ho-FH or DCM or CF or AATD or PCD).ti,ab. (340964)
51 ("retinal dystroph*" or "Retinitis Pigmentosa" or RP or "retinal pigment* dystroph*" or "tapeto retinal degeneration" or "tapetoretinal degeneration" or "tapetoretinal dystrophy").ti,ab. (49225)
52 (achromatognosia or achromatopia or achromatopsia or "colour blind*" or "color blind*" or "color agnosia" or "colour agnosia" or "scoterythrous vision" or "duchenne syndrome" or "duchenne type muscular dystrophy" or "duchenne muscular dystrophy" or "morbus duchenne" or "pseudo hypertrophic myopathic progressive muscular dystrophy").ti,ab. (13233)
53 ("backer muscular dystrophy" or "becker muscular dystrophy" or "dystrophic becker disease" or "becker dystrophy" or "becker type muscular dystrophy").ti,ab. (1795)

54 (sarcoglycanopath* or Friedreich* or "hereditary spinal ataxia" or "spinal hereditary ataxia" or "spinal heredoataxia").ti,ab. (3759)
55 ("cerebroside sulfatase deficiency syndrome" or "cerebroside sulfate storage disease" or "cerebroside sulphate storage disease" or "metachrom* leucodystrophy" or "metachrom* leukodystrophy" or "sulfatide lipidosis" or sulfatidosis or "sulphatide lipidosis").ti,ab. (1483)
56 ("adenylosuccinate lyase" or adenylosuccinase or "adenylosuccinate amp lyase" or "adenylsuccinate lyase" or "e.c. 4.3.2.2" or "succinyladenylate lyase").ti,ab. (346)
57 ("Angelman syndrome" or "happy puppet syndrome").ti,ab. (1726)
58 ("hereditary progressive spinal muscular atrophy" or "hereditary spinal progressive muscular atrophy" or "hoffmann werdnig" or "werdnig hoffman" or ((infantile or "type 1" or "type I") adj3 "musc* atrophy")).ti,ab. (680)
59 (Dubowitz or (("type 2" or "type II") adj3 "musc* atrophy")).ti,ab. (472)
60 (kugelberg* or ((benign or juvenile or "type 3" or "type III") adj3 "musc* atrophy")).ti,ab. (515)
61 ("limb girdle dystroph*" or "limb-girdle muscular dystroph*").ti,ab. (2360)
62 ("GM1 gangliosidosis" or "gangliosidosis G(M1)" or "gangliosidosis gm 1" or "gangliosidosis GM1" or "gm 1 gangliosidosis").ti,ab. (784)
63 ("Aromatic l-amino acid decarboxylase" or "3,4 dihydroxy l phenylalanine carboxylyase" or "3,4 dihydroxyphenylalanine decarboxylase" or "5 hydroxy levo tryptophan carboxylyase" or "5 hydroxytrypthophan decarboxylase" or "5 hydroxytryptophan carboxylyase" or "5 hydroxytryptophan decarboxylase" or "5 hydroxytryptophane decarboxylase" or "aromatic amino acid decarboxylase*" or "aromatic aminoacid decarboxylase*" or "aromatic l amino acid carboxy lyase" or "aromatic l amino acid decarboxylase*" or "aromatic l aminoacid decarboxylase" or "aromatic levo aminoacid decarboxylase" or "dihydroxyphenylalanine decarboxylase" or "DOPA decarboxylase" or "e.c. 4.1.1.28" or "hydroxytryptophan decarboxylase" or "l 3,4 dihydroxyphenylalanine decarboxylase" or "l dopa decarboxylase" or "l tryptophan carboxylyase" or "levo dopa decarboxylase" or "levodopa decarboxylase" or "tryptophan decarboxylase").ti,ab. (3606)
64 (krabbe or "beta galactosidase deficiency syndrome" or "galactosylceramidase deficiency syndrome" or "galactosylceramide lipidosis" or "globoid cell dystrophy" or "globoid cell leucodystrophy" or "globoid cell leukodystrophy" or "mckusick 24520").ti,ab. (1224)
65 (rett or rett's).ti,ab. (4265)
66 15 or 16 or 17 or 18 or 19 or 20 or 21 or 22 or 23 or 24 or 25 or 26 or 27 or 28 or 29 or 30 or 31 or 32 or 33 or 34 or 35 or 36 or 37 or 38 or 39 or 40 or 41 or 42 or 43 or 44 or 45 or 46 or 47 or 48 or 49 or 50 or 51 or 52 or 53 or 54 or 55 or 56 or 57 or 58 or 59 or 60 or 61 or 62 or 63 or 64 or 65 (1506235)
67 pharmacovigilance/ (3515)
68 (Pharmacovigilan* or pharmaco-vigilan* or toxicit* or SUSAR or SAE or AR or SAR or "adverse reaction*" or "adverse event*" or antibodies or immunosuppress* or immunotherap* or "immune management" or immunomodulat* or "subclinical immune response*" or immunologic* or aminotransaminase* or ((liver or hepatic) adj2 (damag* or injur* or inflamm*)) or leukemia or leukaemia or cancer* or tumor* or tumour* or humoral or "cellular immunity" or "innate immunity" or immunogenicity or "complement activation" or cytokine* or "pre-existing immunity" or ADA or cell-mediated or binding-antibod* or "induced response*" or pre-existing or preexisting or boosted or "capsid response*" or redosing or "cell-mediated immun*" or "T-cell immunity" or tolerance or tolerization or

TNF-alpha or prednisolone or eculizumab or "complement activation" or transaminitis or hypertransaminasemia or thrombocytopenia or microangiopathy).ti,ab. (5956431)
69 exp Immunosuppressive Agents/ (348675)
70 exp Leukemia/ (257251)
71 exp Neoplasms/ (3941650)
72 exp "Chemical and Drug Induced Liver Injury"/ (34033)
73 exp Immunogenetic Phenomena/ (49969)
74 exp Cytokines/ (805065)
75 exp Prednisolone/ (54238)
76 exp Thrombocytopenia/ (54496)
77 Ganglia, Spinal/ (20340)
78 exp Anemia, Hemolytic/ (82573)
79 exp Acute Kidney Injury/ (57686)
80 (TMA or "dorsal root ganglion" or "dorsal root spinal ganglion" or "spinal ganglia" or "spine ganglion" or DRG or "hemolytic anemia" or "hemolytic anaemia" or "haemolytic anemia" or "haemolytic anaemia" or "acute kidney damage" or "acute kidney injury" or "acute kidney failure" or "acute renal damage" or "acute renal failure" or "elevated liver enzyme*" or "elevated hepatic enzyme*" or hepatotoxic* or "hepato toxic*" or "liver cell toxicit*" or "liver intoxication" or "liver poison*" or "liver toxic*" or "abnormal T2 hyperintensit*").ti,ab. (148467)
81 67 or 68 or 69 or 70 or 71 or 72 or 73 or 74 or 75 or 76 or 77 or 78 or 79 or 80 (7851538)
82 3 and 9 and 14 and 66 and 81 (536)

Cochrane Central Register of Controlled Trials
Issue 2 of 12, February 2024
#1 MeSH descriptor: [Genetic Therapy] explode all trees 358
#2 ((DNA NEXT therap*) or (gene* near/2 therap*) or (gene* NEXT transfer*) or transgene or (gene NEXT replacement*) or (gene* next edit*) or "gene silencing" or (gene NEXT repair*) or (gene NEXT correction*)):ti,ab,kw 18478
#3 #1 OR #2 18491
#4 MeSH descriptor: [Infusion Pumps] explode all trees 1637
#5 MeSH descriptor: [Epidural Space] explode all trees 294
#6 MeSH descriptor: [Administration, Intravenous] explode all trees 22798
#7 MeSH descriptor: [Administration, Ophthalmic] explode all trees 180
#8 MeSH descriptor: [Injections, Intraocular] explode all trees 1494
#9 MeSH descriptor: [Injections, Intravenous] explode all trees 9025
#10 MeSH descriptor: [Injections, Spinal] explode all trees 1823
#11 (Intravenous* or intra-venous* or IV or systemic or infusion* or intramuscular* or intra-muscular* or IM or intrathecal* or (intra NEXT thecal*) or (cerebrospinal NEXT fluid*) or CSF or intra-CSF or epidural* or spinal* or ophthalmol* or subretinal* or sub-retinal* or intravitreal* or intra-vitreal* or intraorbital* or intra-orbital* or intracerebroventricular* or intra-cerebroventricular* or ICV or (i.c.v. NEXT injection*) or ICVI or intracerebral* or intra-cerebral* or IC or intraventricular* or (intra NEXT ventricular*) or intraparenchyma* or (intra NEXT parenchyma*) or "intra-cisterna magna" or ICM):ti,ab,kw 338369
#12 #4 or #5 or #6 or #7 or #8 or #9 or #10 or #11 338571
#13 MeSH descriptor: [Rare Diseases] explode all trees 77
#14 MeSH descriptor: [Orphan Drug Production] explode all trees 19

#15 MeSH descriptor: [Muscular Atrophy, Spinal] explode all trees 148
#16 MeSH descriptor: [Myopathies, Structural, Congenital] explode all trees 6
#17 MeSH descriptor: [Muscular Dystrophies] explode all trees 659
#18 MeSH descriptor: [Paraplegia] explode all trees 279
#19 MeSH descriptor: [Gangliosidoses] explode all trees 11
#20 MeSH descriptor: [Multiple Sclerosis] explode all trees 5178
#21 MeSH descriptor: [Parkinson Disease] explode all trees 6009
#22 MeSH descriptor: [Alzheimer Disease] explode all trees 5232
#23 MeSH descriptor: [Neuronal Ceroid-Lipofuscinoses] explode all trees 11
#24 MeSH descriptor: [Hereditary Sensory and Motor Neuropathy] explode all trees 119
#25 MeSH descriptor: [Hemophilia A] explode all trees 641
#26 MeSH descriptor: [Anemia, Sickle Cell] explode all trees 1061
#27 MeSH descriptor: [Glycogen Storage Disease Type II] explode all trees 56
#28 MeSH descriptor: [Canavan Disease] explode all trees 3
#29 MeSH descriptor: [Fabry Disease] explode all trees 110
#30 MeSH descriptor: [Mucopolysaccharidoses] explode all trees 106
#31 MeSH descriptor: [Hyperlipoproteinemia Type II] explode all trees 664
#32 MeSH descriptor: [Cardiomyopathy, Dilated] explode all trees 669
#33 MeSH descriptor: [Cystic Fibrosis] explode all trees 2368
#34 MeSH descriptor: [alpha 1-Antitrypsin Deficiency] explode all trees 120
#35 MeSH descriptor: [Ciliary Motility Disorders] explode all trees 32
#36 MeSH descriptor: [Macular Degeneration] explode all trees 3542
#37 MeSH descriptor: [Leber Congenital Amaurosis] explode all trees 14
#38 ((rare NEXT disease*) or (orphan NEXT drug*) or (monogenic NEXT disease*) or (monogenic NEXT disorder*) or "spinal muscular atrophy" or "spine muscle atrophy" or "myotubular myopathy" or "centronuclear myopathy" or "myotonic dystrophy" or "curschmann steinert disease" or "deleage disease" or "dystrophic myotonica" or "dystrophic myotonia" or "myotonia atrophica" or "myotonia dystrophica" or "myotonic atrophia" or "myotonic atrophy" or "myotonic dystrophia" or "myotonic muscular dystrophy" or "myotonic muscular dystrophy" or "proximal myotonic myopathy" or "steinert disease" or "steinert myopathy" or "muscular dystrophy" or "muscle dystrophy" or "muscle dystrophia" or (neuron* NEXT disease) or MND or "Spastic paraplegia" or "spastic paraparesis" or gangliosidosis or "ganglioside lipidosis" or gangliosidoses or "multiple sclerosis" or "chariot disease" or "disseminated sclerosis" or "insular sclerosis" or MS or Parkinson* or "paralysis agitans" or Alzheimer* or Alzeimer* or "diffuse cortical sclerosis" or Batten* or "juvenile neuronal ceroid lipofuscinosis" or "Giant axonal neuropathy" or GAN or GAN-1 or "Amyotrophic lateral sclerosis" or ALS or (lou NEXT gehrig*) or Charcot-Marie-Tooth or "hereditary motor sensory neuropathy" or charcot-marie or "dejerine sottas" or (spastic NEXT para*) or Struempell or strumpel* or SCID-X or Haemophili* or hemophili* or "sickle cell" or drepanocyt* or "haemoglobin ss" or "hemoglobin ss" or "sickle anaemia" or "sickle anemia" or Pompe or "glycogen storage disease type 2" or "cardiomuscular glycogenosis" or "diffuse glycogenosis" or "glycogen storage disease type ii" or (glycogenos* NEAR/2 (ii or targeted gene repair)) or Canavan* or (AADC NEXT deficien*) or Fabry* or "alpha galactosidase deficiency syndrome" or Mucopolysaccharidos* or (familial NEXT hypercholesterol*) or "buerger gruetz" or "burger grutz" or (familial NEXT hyperbetalipoprotein*) or (amilial NEXT hypercholester*) or "dilated cardiomyopathy" or "congestic cardiomyopathy" or "congestive heart disease" or "congestive myocardiopathy" or "cystic fibrosis" or "cystic pancreas fibrosis" or "fibrocystic disease" or "Alpha-1 antitrypsin deficiency" or "alpha 1 proteinase inhibitor deficiency" or "alpha1 antitrypsin deficiency" or "antitrypsin alpha 1 deficiency" or "ciliary dyskinesia" or "ciliary immotility" or "ciliatry

motility disorders" or ciliostasis or (macula* NEXT degeneration) or (macula* NEXT atrophy) or "macular dystrophy" or LCA or Leber):ti,ab,kw 79421
#39 MeSH descriptor: [Retinal Dystrophies] explode all trees 164
#40 MeSH descriptor: [Retinitis Pigmentosa] explode all trees 157
#41 MeSH descriptor: [Color Vision Defects] explode all trees 50
#42 MeSH descriptor: [Friedreich Ataxia] explode all trees 98
#43 MeSH descriptor: [Leukodystrophy, Metachromatic] explode all trees 6
#44 MeSH descriptor: [Adenylosuccinate Lyase] explode all trees 0
#45 MeSH descriptor: [Angelman Syndrome] explode all trees 27
#46 MeSH descriptor: [Leukodystrophy, Globoid Cell] explode all trees 2
#47 MeSH descriptor: [Rett Syndrome] explode all trees 64
#48 (LCA* or SMA or DMD or XMTM or LGMD or CMT or PD or MPS or Ho-FH or DCM or CF or AATD or PCD):ti,ab,kw 54647
#49 ((retinal NEXT dystroph*) or "Retinitis Pigmentosa" or RP or (pigment* NEXT dystroph*) or "tapeto retinal degeneration" or "tapetoretinal degeneration" or "tapetoretinal dystrophy"):ti,ab,kw 3262
#50 (achromatognosia or achromatopia or achromatopsia or (colour NEXT blind*) or (color NEXT blind*) or "color agnosia" or "colour agnosia" or "scoterythrous vision" or "duchenne syndrome" or "duchenne type muscular dystrophy" or "duchenne muscular dystrophy" or "morbus duchenne" or "pseudo hypertrophic myopathic progressive muscular dystrophy"):ti,ab,kw 914
#51 ("backer muscular dystrophy" or "becker muscular dystrophy" or "dystrophic becker disease" or "becker dystrophy" or "becker type muscular dystrophy"):ti,ab,kw 84
#52 (sarcoglycanopath* or Friedreich* or "hereditary spinal ataxia" or "spinal hereditary ataxia" or "spinal heredoataxia"):ti,ab,kw 177
#53 ("cerebroside sulfatase deficiency syndrome" or "cerebroside sulfate storage disease" or "cerebroside sulphate storage disease" or (metachrom* NEXT leucodystrophy) or (metachrom* NEXT leukodystrophy) or "sulfatide lipidosis" or sulfatidosis or "sulphatide lipidosis"):ti,ab,kw 9
#54 ("adenylosuccinate lyase" or adenylosuccinase or "adenylosuccinate amp lyase" or "adenylsuccinate lyase" or "e.c. 4.3.2.2" or "succinyladenylate lyase"):ti,ab,kw 1
#55 ("Angelman syndrome" or "happy puppet syndrome"):ti,ab,kw 42
#56 ("hereditary progressive spinal muscular atrophy" or "hereditary spinal progressive muscular atrophy" or "hoffmann werdnig" or "werdnig hoffman" or ((infantile or "type 1" or "type I") near/4 atrophy)):ti,ab,kw 33
#57 (Dubowitz or (("type 2" or "type II") near/4 atrophy)):ti,ab,kw 78
#58 (kugelberg* or ((benign or juvenile or "type 3" or "type III") near/4 atrophy)):ti,ab,kw 74
#59 ((girdle NEXT dystroph*) or ("limb-girdle muscular" NEXT dystroph*)):ti,ab,kw 41
#60 ("GM1 gangliosidosis" or "gangliosidosis G(M1)" or "gangliosidosis gm 1" or "gangliosidosis GM1" or "gm 1 gangliosidosis"):ti,ab,kw 7
#61 ("Aromatic l-amino acid decarboxylase" or "3,4 dihydroxy l phenylalanine carboxylyase" or "3,4 dihydroxyphenylalanine decarboxylase" or "5 hydroxy levo tryptophan carboxylyase" or "5 hydroxytrypthophan decarboxylase" or "5 hydroxytryptophan carboxylyase" or "5 hydroxytryptophan decarboxylase" or "5 hydroxytryptophane decarboxylase" or ("aromatic amino acid" NEXT decarboxylase*) or ("aromatic aminoacid" NEXT decarboxylase*) or "aromatic l amino acid carboxy lyase" or ("aromatic l amino acid" NEXT decarboxylase*) or "aromatic l aminoacid decarboxylase" or "aromatic levo aminoacid decarboxylase" or "dihydroxyphenylalanine decarboxylase" or "DOPA decarboxylase" or "e.c. 4.1.1.28" or "hydroxytryptophan decarboxylase" or "l 3,4

dihydroxyphenylalanine decarboxylase" or "l dopa decarboxylase" or "l tryptophan carboxylyase" or "levo dopa decarboxylase" or "levodopa decarboxylase" or "tryptophan decarboxylase"):ti,ab,kw 241

#62 (krabbe or "beta galactosidase deficiency syndrome" or "galactosylceramidase deficiency syndrome" or "galactosylceramide lipidosis" or "globoid cell dystrophy" or "globoid cell leucodystrophy" or "globoid cell leukodystrophy" or "mckusick 24520"):ti,ab,kw 8

#63 (rett or rett's):ti,ab,kw 121

#64 #13 or #14 or #15 or #16 or #17 or #18 or #19 or #20 or #21 or #22 or #23 or #24 or #25 or #26 or #27 or #28 or #29 or #30 or #31 or #32 or #33 or #34 or #35 or #36 or #37 or #38 or #39 or #40 or #41 or #42 or #43 or #44 or #45 or #46 or #47 or #48 or #49 or #50 or #51 or #52 or #53 or #54 or #55 or #56 or #57 or #58 or #59 or #60 or #61 or #62 or #63 125949

#65 MeSH descriptor: [Pharmacovigilance] explode all trees 42

#66 (Pharmacovigilan* or pharmaco-vigilan* or toxicit* or SUSAR or SAE or AR or SAR or (adverse NEXT reaction*) or (adverse NEXT event*) or antibodies or immunosuppress* or immunotherap* or "immune management" or immunomodulat* or ("subclinical immune" NEXT response*) or immunologic* or aminotransaminase* or ((liver or hepatic) NEAR/2 (damag* or injur* or inflamm*)) or leukemia or leukaemia or cancer* or tumor* or tumour* or humoral or "cellular immunity" or "innate immunity" or immunogenicity or "complement activation" or cytokine* or "pre-existing immunity" or ADA or cell-mediated or (binding NEXT antibod*) or (induced NEXT response*) or pre-existing or preexisting or boosted or (capsid NEXT response*) or redosing or (cell-mediated NEXT immun*) or "T-cell immunity" or tolerance or tolerization or TNF-alpha or prednisolone or eculizumab or "complement activation" or transaminitis or hypertransaminasemia or thrombocytopenia or microangiopathy):ti,ab,kw 526912

#67 MeSH descriptor: [Immunosuppressive Agents] explode all trees 6937

#68 MeSH descriptor: [Leukemia] explode all trees 6529

#69 MeSH descriptor: [Neoplasms] explode all trees 123839

#70 MeSH descriptor: [Chemical and Drug Induced Liver Injury] explode all trees 474

#71 MeSH descriptor: [Immunogenetic Phenomena] explode all trees 135

#72 MeSH descriptor: [Cytokines] explode all trees 27506

#73 MeSH descriptor: [Prednisolone] explode all trees 5996

#74 MeSH descriptor: [Thrombocytopenia] explode all trees 1790

#75 MeSH descriptor: [Ganglia, Spinal] explode all trees 71

#76 MeSH descriptor: [Anemia, Hemolytic] explode all trees 1840

#77 MeSH descriptor: [Acute Kidney Injury] explode all trees 2347

#78 (TMA or "dorsal root ganglion" or "dorsal root spinal ganglion" or "spinal ganglia" or "spine ganglion" or DRG or "hemolytic anemia" or "hemolytic anaemia" or "haemolytic anemia" or "haemolytic anaemia" or "acute kidney damage" or "acute kidney injury" or "acute kidney failure" or "acute renal damage" or "acute renal failure" or ("elevated liver" NEXT enzyme*) or ("elevated hepatic" NEXT enzyme*) or hepatotoxic* or (hepato NEXT toxic*) or ("liver cell" NEXT toxicit*) or "liver intoxication" or (liver NEXT poison*) or (liver NEXT toxic*) or ("abnormal T2" NEXT hyperintensit*)):ti,ab,kw 11367

#79 #65 or #66 or #67 or #68 or #69 or #70 or #71 or #72 or #73 or #74 or #75 or #76 or #77 or #78 560567

#80 #3 and #12 and #64 and #79 732

Table S2. Search Results for Real-World Studies.

Ovid Embase	433
Ovid Medline	44
Cochrane CENTRAL	31
Total	508
Total after deduplication	445

Database: Embase 1974 to present

Database: Medline (Ovid MEDLINE® Epub Ahead of Print, In-Process & Other Non-Indexed Citations, Ovid MEDLINE® Daily and Ovid MEDLINE®) 1946 to present

Search Strategy:

----

1 exp gene therapy/ (102165)
2 targeted gene repair/ (445)
3 ("DNA therap*" or (gene* adj2 therap*) or "gene* transfer*" or transgene or "gene replacement*" or "gene-edit*" or "gene silencing" or "gene repair*" or "gene correction*").ti,ab. (212907)
4 1 or 2 or 3 (251960)
5 epidural drug administration/ or intracisternal drug administration/ or intrathecal drug administration/ (27063)
6 intraocular drug administration/ or intracorneal drug administration/ or intravitreal drug administration/ or subretinal drug administration/ (10616)
7 exp intraspinal drug administration/ (29464)
8 exp intraocular drug administration/ (12131)
9 drug infusion/ (17165)
10 exp intracerebral drug administration/ (31720)
11 intravenous drug administration/ (384430)
12 (Intravenous* or intra-venous* or IV or systemic or infusion* or intramuscular* or intra-muscular* or IM or intrathecal* or intra-thecal* or "cerebrospinal fluid*" or CSF or intra-CSF or epidural* or spinal* or ophthalmol* or subretinal* or sub-retinal* or intravitreal* or intra-vitreal* or intraorbital* or intra-orbital* or intracerebroventricular* or intra-cerebroventricular* or ICV or "i.c.v. injection*" or ICVI or intracerebral* or intra-cerebral* or IC or intraventricular* or intra-ventricular* or intraparenchyma* or intra-parenchyma* or "intra-cisterna magna" or ICM).ti,ab. (3195173)
13 5 or 6 or 7 or 8 or 9 or 10 or 11 or 12 (3407200)
14 rare disease/ (50265)
15 orphan drug/ (4013)
16 exp spinal muscular atrophy/ (71765)
17 centronuclear myopathy/ (1323)
18 myotonic dystrophy/ (9744)
19 exp muscular dystrophy/ (52657)
20 spastic paraplegia/ (5101)
21 exp gangliosidosis/ (4390)

22 multiple sclerosis/ (161878)
23 parkinson disease/ or autosomal dominant parkinson disease/ (196772)
24 Alzheimer disease/ (257013)
25 juvenile neuronal ceroid lipofuscinosis/ (295)
26 giant axonal neuropathy/ (320)
27 hereditary motor sensory neuropathy/ (13412)
28 exp hemophilia/ (46582)
29 exp sickle cell anemia/ (47775)
30 glycogen storage disease type 2/ (5209)
31 Canavan disease/ (818)
32 Fabry disease/ (9593)
33 exp mucopolysaccharidosis/ (14039)
34 familial hypercholesterolemia/ (12739)
35 exp congestive cardiomyopathy/ (55227)
36 cystic fibrosis/ (86136)
37 alpha 1 antitrypsin deficiency/ (6642)
38 ciliary dyskinesia/ (3703)
39 exp macular degeneration/ (27716)
40 Leber congenital amaurosis/ (2306)
41 exp monogenic disorder/ (573870)
42 ("rare disease*" or "orphan drug*" or "monogenic disease*" or "monogenic disorder*" or "spinal muscular atrophy" or "spine muscle atrophy" or "myotubular myopathy" or "centronuclear myopathy" or "myotonic dystrophy" or "curschmann steinert disease" or "deleage disease" or "dystrophic myotonica" or "dystrophic myotonia" or "myotonia atrophica" or "myotonia dystrophica" or "myotonic atrophia" or "myotonic atrophy" or "myotonic dystrophia" or "myotonic muscular dystrophy" or "myotonic muscular dystrophy" or "proximal myotonic myopathy" or "steinert disease" or "steinert myopathy" or "muscular dystrophy" or "muscle dystrophy" or "muscle dystrophia" or "motor neuron* disease" or MND or "Spastic paraplegia" or "spastic paraparesis" or gangliosidosis or "ganglioside lipidosis" or gangliosidoses or "multiple sclerosis" or "chariot disease" or "disseminated sclerosis" or "insular sclerosis" or MS or Parkinson* or "paralysis agitans" or Alzheimer* or Alzeimer* or "diffuse cortical sclerosis" or Batten* or "juvenile neuronal ceroid lipofuscinosis" or "Giant axonal neuropathy" or GAN or GAN-1 or "Amyotrophic lateral sclerosis" or ALS or "lou gehrig*" or Charcot-Marie-Tooth or "hereditary motor sensory neuropathy" or "charcot-marie" or "dejerine sottas" or "familial spastic para*" or "hereditary spastic para*" or Struempell or strumpel* or SCID-X or Haemophili* or hemophili* or "sickle cell" or drepanocyt* or "haemoglobin ss" or "hemoglobin ss" or "sickle anaemia" or "sickle anemia" or Pompe or "glycogen storage disease type 2" or "cardiomuscular glycogenosis" or "diffuse glycogenosis" or "glycogen storage disease type ii" or (glycogenos* adj2 (ii or targeted gene repair)) or Canavan* or "AADC deficien*" or Fabry* or "alpha galactosidase deficiency syndrome" or Mucopolysaccharidos* or "familial hypercholesterol*" or "buerger gruetz" or "burger grutz" or "familial hyperbetalipoprotein*" or "amilial hypercholester*" or "dilated cardiomyopathy" or "congestic cardiomyopathy" or "congestive heart disease" or "congestive myocardiopathy" or "cystic fibrosis" or "cystic pancreas fibrosis" or "fibrocystic disease" or "Alpha-1 antitrypsin deficiency" or "alpha 1 proteinase inhibitor deficiency" or "alpha1 antitrypsin deficiency" or "antitrypsin alpha 1 deficiency" or "ciliary dyskinesia" or "ciliary immotility" or "ciliatry motility disorders" or ciliostasis or "macula* degeneration" or "macula* atrophy" or "macular dystrophy" or LCA or Leber).ti,ab. (1546055)
43 exp retina dystrophy/ (5296)

44 retinitis pigmentosa/ (13362)
45 color blindness/ (2021)
46 Duchenne muscular dystrophy/ (20330)
47 Becker muscular dystrophy/ (3590)
48 sarcoglycanopathy/ (348)
49 Friedreich ataxia/ (5028)
50 metachromatic leukodystrophy/ (2429)
51 adenylosuccinate lyase/ (454)
52 happy puppet syndrome/ (3332)
53 Werdnig Hoffmann disease/ (1410)
54 spinal muscular atrophy type 2/ (635)
55 Kugelberg Welander disease/ (714)
56 limb girdle muscular dystrophy/ (4278)
57 GM1 gangliosidosis/ (1100)
58 aromatic levo amino acid decarboxylase/ (3428)
59 globoid cell leukodystrophy/ (2158)
60 Rett syndrome/ (6379)
61 (LCA* or SMA or DMD or XMTM or LGMD or CMT or PD or MPS or Ho-FH or DCM or CF or AATD or PCD).ti,ab. (548959)
62 ("retinal dystroph*" or "Retinitis Pigmentosa" or RP or "retinal pigment* dystroph*" or "tapeto retinal degeneration" or "tapetoretinal degeneration" or "tapetoretinal dystrophy").ti,ab. (79169)
63 (achromatognosia or achromatopia or achromatopsia or "colour blind*" or "color blind*" or "color agnosia" or "colour agnosia" or "scoterythrous vision" or "duchenne syndrome" or "duchenne type muscular dystrophy" or "duchenne muscular dystrophy" or "morbus duchenne" or "pseudo hypertrophic myopathic progressive muscular dystrophy").ti,ab. (18435)
64 ("backer muscular dystrophy" or "becker muscular dystrophy" or "dystrophic becker disease" or "becker dystrophy" or "becker type muscular dystrophy").ti,ab. (2569)
65 (sarcoglycanopath* or Friedreich* or "hereditary spinal ataxia" or "spinal hereditary ataxia" or "spinal heredoataxia").ti,ab. (4744)
66 ("cerebroside sulfatase deficiency syndrome" or "cerebroside sulfate storage disease" or "cerebroside sulphate storage disease" or "metachrom* leucodystrophy" or "metachrom* leukodystrophy" or "sulfatide lipidosis" or sulfatidosis or "sulphatide lipidosis").ti,ab. (1839)
67 ("adenylosuccinate lyase" or adenylosuccinase or "adenylosuccinate amp lyase" or "adenylsuccinate lyase" or "e.c. 4.3.2.2" or "succinyladenylate lyase").ti,ab. (381)
68 ("Angelman syndrome" or "happy puppet syndrome").ti,ab. (2211)
69 ("hereditary progressive spinal muscular atrophy" or "hereditary spinal progressive muscular atrophy" or "hoffmann werdnig" or "werdnig hoffman" or ((infantile or "type 1" or "type I") adj3 "musc* atrophy")).ti,ab. (1090)
70 (Dubowitz or (("type 2" or "type II") adj3 "musc* atrophy")).ti,ab. (702)
71 (kugelberg* or ((benign or juvenile or "type 3" or "type III") adj3 "musc* atrophy")).ti,ab. (584)
72 ("limb girdle dystroph*" or "limb-girdle muscular dystroph*").ti,ab. (3733)
73 ("GM1 gangliosidosis" or "gangliosidosis G(M1)" or "gangliosidosis gm 1" or "gangliosidosis GM1" or "gm 1 gangliosidosis").ti,ab. (924)
74 ("Aromatic l-amino acid decarboxylase" or "3,4 dihydroxy l phenylalanine carboxylyase" or "3,4 dihydroxyphenylalanine decarboxylase" or "5 hydroxy levo tryptophan carboxylyase" or "5 hydroxytrypthophan decarboxylase" or "5 hydroxytryptophan carboxylyase" or "5 hydroxytryptophan decarboxylase" or "5 hydroxytryptophane decarboxylase" or "aromatic

amino acid decarboxylase*" or "aromatic aminoacid decarboxylase*" or "aromatic l amino acid carboxy lyase" or "aromatic l amino acid decarboxylase*" or "aromatic l aminoacid decarboxylase" or "aromatic levo aminoacid decarboxylase" or "dihydroxyphenylalanine decarboxylase" or "DOPA decarboxylase" or "e.c. 4.1.1.28" or "hydroxytryptophan decarboxylase" or "l 3,4 dihydroxyphenylalanine decarboxylase" or "l dopa decarboxylase" or "l tryptophan carboxylyase" or "levo dopa decarboxylase" or "levodopa decarboxylase" or "tryptophan decarboxylase").ti,ab. (4204)
75 (krabbe or "beta galactosidase deficiency syndrome" or "galactosylceramidase deficiency syndrome" or "galactosylceramide lipidosis" or "globoid cell dystrophy" or "globoid cell leucodystrophy" or "globoid cell leukodystrophy" or "mckusick 24520").ti,ab. (1625)
76 (rett or rett's).ti,ab. (5514)
77 14 or 15 or 16 or 17 or 18 or 19 or 20 or 21 or 22 or 23 or 24 or 25 or 26 or 27 or 28 or 29 or 30 or 31 or 32 or 33 or 34 or 35 or 36 or 37 or 38 or 39 or 40 or 41 or 42 or 43 or 44 or 45 or 46 or 47 or 48 or 49 or 50 or 51 or 52 or 53 or 54 or 55 or 56 or 57 or 58 or 59 or 60 or 61 or 62 or 63 or 64 or 65 or 66 or 67 or 68 or 69 or 70 or 71 or 72 or 73 or 74 or 75 or 76 (2541161)
78 exp pharmacovigilance/ (23261)
79 (Pharmacovigilan* or pharmaco-vigilan* or toxicit* or SUSAR or SAE or AR or SAR or "adverse reaction*" or "adverse event*" or antibodies or immunosuppress* or immunotherap* or "immune management" or immunomodulat* or "subclinical immune response*" or immunologic* or aminotransaminase* or ((liver or hepatic) adj2 (damag* or injur* or inflamm*)) or leukemia or leukaemia or cancer* or tumor* or tumour* or humoral or "cellular immunity" or "innate immunity" or immunogenicity or "complement activation" or cytokine* or "pre-existing immunity" or ADA or cell-mediated or binding-antibod* or "induced response*" or pre-existing or preexisting or boosted or "capsid response*" or redosing or "cell-mediated immun*" or "T-cell immunity" or tolerance or tolerization or TNF-alpha or prednisolone or eculizumab or "complement activation" or transaminitis or hypertransaminasemia or thrombocytopenia or microangiopathy).ti,ab. (8022126)
80 exp immunosuppressive treatment/ (260883)
81 exp leukemia/ (378133)
82 exp neoplasm/ (5678672)
83 exp liver injury/ (81741)
84 exp immunogenicity/ (89304)
85 exp cytokine/ (1961953)
86 prednisolone/ (153065)
87 eculizumab/ (9820)
88 exp thrombocytopenia/ (232160)
89 exp microangiopathy/ or hypertransaminasemia/ (79139)
90 spinal ganglion/ (26536)
91 exp hemolytic anemia/ (140088)
92 kidney injury/ or acute kidney failure/ (175604)
93 exp liver toxicity/ (103332)
94 (TMA or "dorsal root ganglion" or "dorsal root spinal ganglion" or "spinal ganglia" or "spine ganglion" or DRG or "hemolytic anemia" or "hemolytic anaemia" or "haemolytic anemia" or "haemolytic anaemia" or "acute kidney damage" or "acute kidney injury" or "acute kidney failure" or "acute renal damage" or "acute renal failure" or "elevated liver enzyme*" or "elevated hepatic enzyme*" or hepatotoxic* or "hepato toxic*" or "liver cell toxicit*" or "liver intoxication" or "liver poison*" or "liver toxic*" or "abnormal T2 hyperintensit*").ti,ab. (222334)
95 78 or 79 or 80 or 81 or 82 or 83 or 84 or 85 or 86 or 87 or 88 or 89 or 90 or 91 or 92 or 93

or 94 (10809812)
96 4 and 13 and 77 and 95 (5391)
97 case report/ (2972354)
98 observational study/ (361533)
99 ("case report*" or real-world or observational).ti,ab,kf. (1237582)
100 97 or 98 or 99 (3729719)
101 96 and 100 (433)

Database: Medline (Ovid MEDLINE® Epub Ahead of Print, In-Process & Other Non-Indexed Citations, Ovid MEDLINE® Daily and Ovid MEDLINE®) 1946 to present
Search Strategy:

----

1 exp Genetic Therapy/ (54409)
2 ("DNA therap*" or (gene* adj2 therap*) or "gene* transfer*" or transgene or "gene replacement*" or "gene-edit*" or "gene silencing" or "gene repair*" or "gene correction*").ti,ab. (162791)
3 1 or 2 (181624)
4 Epidural Space/ (4886)
5 exp administration, intravenous/ or administration, ophthalmic/ (150896)
6 exp injections, intraocular/ or injections, intravenous/ or exp injections, spinal/ (110553)
7 (Intravenous* or intra-venous* or IV or systemic or infusion* or intramuscular* or intra-muscular* or IM or intrathecal* or intra-thecal* or "cerebrospinal fluid*" or CSF or intra-CSF or epidural* or spinal* or ophthalmol* or subretinal* or sub-retinal* or intravitreal* or intra-vitreal* or intraorbital* or intra-orbital* or intracerebroventricular* or intra-cerebroventricular* or ICV or "i.c.v. injection*" or ICVI or intracerebral* or intra-cerebral* or IC or intraventricular* or intra-ventricular* or intraparenchyma* or intra-parenchyma* or "intra-cisterna magna" or ICM).ti,ab. (2316440)
8 4 or 5 or 6 or 7 (2362480)
9 Rare Diseases/ (14380)
10 Orphan Drug Production/ (1465)
11 exp Muscular Atrophy, Spinal/ (6639)
12 exp Myopathies, Structural, Congenital/ (1624)
13 exp Muscular Dystrophies/ (30356)
14 exp Paraplegia/ (13603)
15 exp Gangliosidoses/ (2695)
16 exp Multiple Sclerosis/ (71739)
17 Parkinson Disease/ (84852)
18 Alzheimer Disease/ (123730)
19 Neuronal Ceroid-Lipofuscinoses/ (2273)
20 exp "Hereditary Sensory and Motor Neuropathy"/ (8261)
21 hemophilia a/ or hemophilia b/ (25211)
22 exp Anemia, Sickle Cell/ (26145)
23 Glycogen Storage Disease Type II/ (1995)
24 Canavan Disease/ (313)

25 Fabry Disease/ (4224)
26 exp Mucopolysaccharidoses/ (7094)
27 Hyperlipoproteinemia Type II/ (7796)
28 Cardiomyopathy, Dilated/ (17408)
29 Cystic Fibrosis/ (40582)
30 alpha 1-Antitrypsin Deficiency/ (3789)
31 exp Ciliary Motility Disorders/ (2699)
32 exp Macular Degeneration/ (31035)
33 Leber Congenital Amaurosis/ (586)
34 ("rare disease*" or "orphan drug*" or "monogenic disease*" or "monogenic disorder*" or "spinal muscular atrophy" or "spine muscle atrophy" or "myotubular myopathy" or "centronuclear myopathy" or "myotonic dystrophy" or "curschmann steinert disease" or "deleage disease" or "dystrophic myotonica" or "dystrophic myotonia" or "myotonia atrophica" or "myotonia dystrophica" or "myotonic atrophia" or "myotonic atrophy" or "myotonic dystrophia" or "myotonic muscular dystrophy" or "myotonic muscular dystrophy" or "proximal myotonic myopathy" or "steinert disease" or "steinert myopathy" or "muscular dystrophy" or "muscle dystrophy" or "muscle dystrophia" or "motor neuron* disease" or MND or "Spastic paraplegia" or "spastic paraparesis" or gangliosidosis or "ganglioside lipidosis" or gangliosidoses or "multiple sclerosis" or "chariot disease" or "disseminated sclerosis" or "insular sclerosis" or MS or Parkinson* or "paralysis agitans" or Alzheimer* or Alzeimer* or "diffuse cortical sclerosis" or Batten* or "juvenile neuronal ceroid lipofuscinosis" or "Giant axonal neuropathy" or GAN or GAN-1 or "Amyotrophic lateral sclerosis" or ALS or "lou gehrig*" or Charcot-Marie-Tooth or "hereditary motor sensory neuropathy" or "charcot-marie" or "dejerine sottas" or "familial spastic para*" or "hereditary spastic para*" or Struempell or strumpel* or SCID-X or Haemophili* or hemophili* or "sickle cell" or drepanocyt* or "haemoglobin ss" or "hemoglobin ss" or "sickle anaemia" or "sickle anemia" or Pompe or "glycogen storage disease type 2" or "cardiomuscular glycogenosis" or "diffuse glycogenosis" or "glycogen storage disease type ii" or (glycogenos* adj2 (ii or targeted gene repair)) or Canavan* or "AADC deficien*" or Fabry* or "alpha galactosidase deficiency syndrome" or Mucopolysaccharidos* or "familial hypercholesterol*" or "buerger gruetz" or "burger grutz" or "familial hyperbetalipoprotein*" or "amilial hypercholester*" or "dilated cardiomyopathy" or "congestic cardiomyopathy" or "congestive heart disease" or "congestive myocardiopathy" or "cystic fibrosis" or "cystic pancreas fibrosis" or "fibrocystic disease" or "Alpha-1 antitrypsin deficiency" or "alpha 1 proteinase inhibitor deficiency" or "alpha1 antitrypsin deficiency" or "antitrypsin alpha 1 deficiency" or "ciliary dyskinesia" or "ciliary immotility" or "ciliatry motility disorders" or ciliostasis or "macula* degeneration" or "macula* atrophy" or "macular dystrophy" or LCA or Leber).ti,ab. (1119488)
35 exp Retinal Dystrophies/ (10905)
36 exp Retinitis Pigmentosa/ (10845)
37 Color Vision Defects/ (4266)
38 Friedreich Ataxia/ (2824)
39 Leukodystrophy, Metachromatic/ (1330)
40 Adenylosuccinate Lyase/ (267)
41 Angelman Syndrome/ (1406)
42 Leukodystrophy, Globoid Cell/ (1091)

43 Rett Syndrome/ (3003)
44 (LCA* or SMA or DMD or XMTM or LGMD or CMT or PD or MPS or Ho-FH or DCM or CF or AATD or PCD).ti,ab. (340964)
45 ("retinal dystroph*" or "Retinitis Pigmentosa" or RP or "retinal pigment* dystroph*" or "tapeto retinal degeneration" or "tapetoretinal degeneration" or "tapetoretinal dystrophy").ti,ab. (49225)
46 (achromatognosia or achromatopia or achromatopsia or "colour blind*" or "color blind*" or "color agnosia" or "colour agnosia" or "scoterythrous vision" or "duchenne syndrome" or "duchenne type muscular dystrophy" or "duchenne muscular dystrophy" or "morbus duchenne" or "pseudo hypertrophic myopathic progressive muscular dystrophy").ti,ab. (13233)
47 ("backer muscular dystrophy" or "becker muscular dystrophy" or "dystrophic becker disease" or "becker dystrophy" or "becker type muscular dystrophy").ti,ab. (1795)
48 (sarcoglycanopath* or Friedreich* or "hereditary spinal ataxia" or "spinal hereditary ataxia" or "spinal heredoataxia").ti,ab. (3759)
49 ("cerebroside sulfatase deficiency syndrome" or "cerebroside sulfate storage disease" or "cerebroside sulphate storage disease" or "metachrom* leucodystrophy" or "metachrom* leukodystrophy" or "sulfatide lipidosis" or sulfatidosis or "sulphatide lipidosis").ti,ab. (1483)
50 ("adenylosuccinate lyase" or adenylosuccinase or "adenylosuccinate amp lyase" or "adenylsuccinate lyase" or "e.c. 4.3.2.2" or "succinyladenylate lyase").ti,ab. (346)
51 ("Angelman syndrome" or "happy puppet syndrome").ti,ab. (1726)
52 ("hereditary progressive spinal muscular atrophy" or "hereditary spinal progressive muscular atrophy" or "hoffmann werdnig" or "werdnig hoffman" or ((infantile or "type 1" or "type I") adj3 "musc* atrophy")).ti,ab. (680)
53 (Dubowitz or (("type 2" or "type II") adj3 "musc* atrophy")).ti,ab. (472)
54 (kugelberg* or ((benign or juvenile or "type 3" or "type III") adj3 "musc* atrophy")).ti,ab. (515)
55 ("limb girdle dystroph*" or "limb-girdle muscular dystroph*").ti,ab. (2360)
56 ("GM1 gangliosidosis" or "gangliosidosis G(M1)" or "gangliosidosis gm 1" or "gangliosidosis GM1" or "gm 1 gangliosidosis").ti,ab. (784)
57 ("Aromatic l-amino acid decarboxylase" or "3,4 dihydroxy l phenylalanine carboxylyase" or "3,4 dihydroxyphenylalanine decarboxylase" or "5 hydroxy levo tryptophan carboxylyase" or "5 hydroxytrypthophan decarboxylase" or "5 hydroxytryptophan carboxylyase" or "5 hydroxytryptophan decarboxylase" or "5 hydroxytryptophane decarboxylase" or "aromatic amino acid decarboxylase*" or "aromatic aminoacid decarboxylase*" or "aromatic l amino acid carboxy lyase" or "aromatic l amino acid decarboxylase*" or "aromatic l aminoacid decarboxylase" or "aromatic levo aminoacid decarboxylase" or "dihydroxyphenylalanine decarboxylase" or "DOPA decarboxylase" or "e.c. 4.1.1.28" or "hydroxytryptophan decarboxylase" or "l 3,4 dihydroxyphenylalanine decarboxylase" or "l dopa decarboxylase" or "l tryptophan carboxylyase" or "levo dopa decarboxylase" or "levodopa decarboxylase" or "tryptophan decarboxylase").ti,ab. (3606)
58 (krabbe or "beta galactosidase deficiency syndrome" or "galactosylceramidase deficiency syndrome" or "galactosylceramide lipidosis" or "globoid cell dystrophy" or "globoid cell leucodystrophy" or "globoid cell leukodystrophy" or "mckusick 24520").ti,ab. (1224)
59 (rett or rett's).ti,ab. (4265)
60 9 or 10 or 11 or 12 or 13 or 14 or 15 or 16 or 17 or 18 or 19 or 20 or 21 or 22 or 23 or 24

or 25 or 26 or 27 or 28 or 29 or 30 or 31 or 32 or 33 or 34 or 35 or 36 or 37 or 38 or 39 or 40 or 41 or 42 or 43 or 44 or 45 or 46 or 47 or 48 or 49 or 50 or 51 or 52 or 53 or 54 or 55 or 56 or 57 or 58 or 59 (1506235)
61 pharmacovigilance/ (3515)
62 (Pharmacovigilan* or pharmaco-vigilan* or toxicit* or SUSAR or SAE or AR or SAR or "adverse reaction*" or "adverse event*" or antibodies or immunosuppress* or immunotherap* or "immune management" or immunomodulat* or "subclinical immune response*" or immunologic* or aminotransaminase* or ((liver or hepatic) adj2 (damag* or injur* or inflamm*)) or leukemia or leukaemia or cancer* or tumor* or tumour* or humoral or "cellular immunity" or "innate immunity" or immunogenicity or "complement activation" or cytokine* or "pre-existing immunity" or ADA or cell-mediated or binding-antibod* or "induced response*" or pre-existing or preexisting or boosted or "capsid response*" or redosing or "cell-mediated immun*" or "T-cell immunity" or tolerance or tolerization or TNF-alpha or prednisolone or eculizumab or "complement activation" or transaminitis or hypertransaminasemia or thrombocytopenia or microangiopathy).ti,ab. (5956431)
63 exp Immunosuppressive Agents/ (348675)
64 exp Leukemia/ (257251)
65 exp Neoplasms/ (3941650)
66 exp "Chemical and Drug Induced Liver Injury"/ (34033)
67 exp Immunogenetic Phenomena/ (49969)
68 exp Cytokines/ (805065)
69 exp Prednisolone/ (54238)
70 exp Thrombocytopenia/ (54496)
71 Ganglia, Spinal/ (20340)
72 exp Anemia, Hemolytic/ (82573)
73 exp Acute Kidney Injury/ (57686)
74 (TMA or "dorsal root ganglion" or "dorsal root spinal ganglion" or "spinal ganglia" or "spine ganglion" or DRG or "hemolytic anemia" or "hemolytic anaemia" or "haemolytic anemia" or "haemolytic anaemia" or "acute kidney damage" or "acute kidney injury" or "acute kidney failure" or "acute renal damage" or "acute renal failure" or "elevated liver enzyme*" or "elevated hepatic enzyme*" or hepatotoxic* or "hepato toxic*" or "liver cell toxicit*" or "liver intoxication" or "liver poison*" or "liver toxic*" or "abnormal T2 hyperintensit*").ti,ab. (148467)
75 61 or 62 or 63 or 64 or 65 or 66 or 67 or 68 or 69 or 70 or 71 or 72 or 73 or 74 (7851538)
76 3 and 8 and 60 and 75 (1510)
77 case reports/ (2388500)
78 Observational Study/ (152822)
79 ("case report*" or real-world or observational).ti,ab,kf. (885009)
80 77 or 78 or 79 (2902175)
81 76 and 80 (44)

Cochrane Central Register of Controlled Trials
Issue 2 of 12, February 2024

#1 MeSH descriptor: [Genetic Therapy] explode all trees 358

#2 ((DNA NEXT therap*) or (gene* near/2 therap*) or (gene* NEXT transfer*) or transgene or (gene NEXT replacement*) or (gene* next edit*) or "gene silencing" or (gene NEXT repair*) or (gene NEXT correction*)):ti,ab,kw 18478
#3 #1 OR #2 18491
#4 MeSH descriptor: [Infusion Pumps] explode all trees 1637
#5 MeSH descriptor: [Epidural Space] explode all trees 294
#6 MeSH descriptor: [Administration, Intravenous] explode all trees 22798
#7 MeSH descriptor: [Administration, Ophthalmic] explode all trees 180
#8 MeSH descriptor: [Injections, Intraocular] explode all trees 1494
#9 MeSH descriptor: [Injections, Intravenous] explode all trees 9025
#10 MeSH descriptor: [Injections, Spinal] explode all trees 1823
#11 (Intravenous* or intra-venous* or IV or systemic or infusion* or intramuscular* or intra-muscular* or IM or intrathecal* or (intra NEXT thecal*) or (cerebrospinal NEXT fluid*) or CSF or intra-CSF or epidural* or spinal* or ophthalmol* or subretinal* or sub-retinal* or intravitreal* or intra-vitreal* or intraorbital* or intra-orbital* or intracerebroventricular* or intra-cerebroventricular* or ICV or (i.c.v. NEXT injection*) or ICVI or intracerebral* or intra-cerebral* or IC or intraventricular* or (intra NEXT ventricular*) or intraparenchyma* or (intra NEXT parenchyma*) or "intra-cisterna magna" or ICM):ti,ab,kw 338369
#12 #4 or #5 or #6 or #7 or #8 or #9 or #10 or #11 338571
#13 MeSH descriptor: [Rare Diseases] explode all trees 77
#14 MeSH descriptor: [Orphan Drug Production] explode all trees 19
#15 MeSH descriptor: [Muscular Atrophy, Spinal] explode all trees 148
#16 MeSH descriptor: [Myopathies, Structural, Congenital] explode all trees 6
#17 MeSH descriptor: [Muscular Dystrophies] explode all trees 659
#18 MeSH descriptor: [Paraplegia] explode all trees 279
#19 MeSH descriptor: [Gangliosidoses] explode all trees 11
#20 MeSH descriptor: [Multiple Sclerosis] explode all trees 5178
#21 MeSH descriptor: [Parkinson Disease] explode all trees 6009
#22 MeSH descriptor: [Alzheimer Disease] explode all trees 5232
#23 MeSH descriptor: [Neuronal Ceroid-Lipofuscinoses] explode all trees 11
#24 MeSH descriptor: [Hereditary Sensory and Motor Neuropathy] explode all trees 119
#25 MeSH descriptor: [Hemophilia A] explode all trees 641
#26 MeSH descriptor: [Anemia, Sickle Cell] explode all trees 1061
#27 MeSH descriptor: [Glycogen Storage Disease Type II] explode all trees 56
#28 MeSH descriptor: [Canavan Disease] explode all trees 3
#29 MeSH descriptor: [Fabry Disease] explode all trees 110
#30 MeSH descriptor: [Mucopolysaccharidoses] explode all trees 106
#31 MeSH descriptor: [Hyperlipoproteinemia Type II] explode all trees 664
#32 MeSH descriptor: [Cardiomyopathy, Dilated] explode all trees 669
#33 MeSH descriptor: [Cystic Fibrosis] explode all trees 2368
#34 MeSH descriptor: [alpha 1-Antitrypsin Deficiency] explode all trees 120
#35 MeSH descriptor: [Ciliary Motility Disorders] explode all trees 32
#36 MeSH descriptor: [Macular Degeneration] explode all trees 3542
#37 MeSH descriptor: [Leber Congenital Amaurosis] explode all trees 14
#38 ((rare NEXT disease*) or (orphan NEXT drug*) or (monogenic NEXT disease*) or (monogenic NEXT disorder*) or "spinal muscular atrophy" or "spine muscle atrophy" or "myotubular myopathy" or "centronuclear myopathy" or "myotonic dystrophy" or "curschmann steinert disease" or "deleage disease" or "dystrophic myotonica" or "dystrophic myotonia" or "myotonia atrophica" or "myotonia dystrophica" or "myotonic atrophia" or

"myotonic atrophy" or "myotonic dystrophia" or "myotonic muscular dystrophy" or "myotonic muscular dystrophy" or "proximal myotonic myopathy" or "steinert disease" or "steinert myopathy" or "muscular dystrophy" or "muscle dystrophy" or "muscle dystrophia" or (neuron* NEXT disease) or MND or "Spastic paraplegia" or "spastic paraparesis" or gangliosidosis or "ganglioside lipidosis" or gangliosidoses or "multiple sclerosis" or "chariot disease" or "disseminated sclerosis" or "insular sclerosis" or MS or Parkinson* or "paralysis agitans" or Alzheimer* or Alzeimer* or "diffuse cortical sclerosis" or Batten* or "juvenile neuronal ceroid lipofuscinosis" or "Giant axonal neuropathy" or GAN or GAN-1 or "Amyotrophic lateral sclerosis" or ALS or (lou NEXT gehrig*) or Charcot-Marie-Tooth or "hereditary motor sensory neuropathy" or charcot-marie or "dejerine sottas" or (spastic NEXT para*) or Struempell or strumpel* or SCID-X or Haemophili* or hemophili* or "sickle cell" or drepanocyt* or "haemoglobin ss" or "hemoglobin ss" or "sickle anaemia" or "sickle anemia" or Pompe or "glycogen storage disease type 2" or "cardiomuscular glycogenosis" or "diffuse glycogenosis" or "glycogen storage disease type ii" or (glycogenos* NEAR/2 (ii or targeted gene repair)) or Canavan* or (AADC NEXT deficien*) or Fabry* or "alpha galactosidase deficiency syndrome" or Mucopolysaccharidos* or (familial NEXT hypercholesterol*) or "buerger gruetz" or "burger grutz" or (familial NEXT hyperbetalipoprotein*) or (amilial NEXT hypercholester*) or "dilated cardiomyopathy" or "congestic cardiomyopathy" or "congestive heart disease" or "congestive myocardiopathy" or "cystic fibrosis" or "cystic pancreas fibrosis" or "fibrocystic disease" or "Alpha-1 antitrypsin deficiency" or "alpha 1 proteinase inhibitor deficiency" or "alpha1 antitrypsin deficiency" or "antitrypsin alpha 1 deficiency" or "ciliary dyskinesia" or "ciliary immotility" or "ciliatry motility disorders" or ciliostasis or (macula* NEXT degeneration) or (macula* NEXT atrophy) or "macular dystrophy" or LCA or Leber):ti,ab,kw 79421
#39 MeSH descriptor: [Retinal Dystrophies] explode all trees 164
#40 MeSH descriptor: [Retinitis Pigmentosa] explode all trees 157
#41 MeSH descriptor: [Color Vision Defects] explode all trees 50
#42 MeSH descriptor: [Friedreich Ataxia] explode all trees 98
#43 MeSH descriptor: [Leukodystrophy, Metachromatic] explode all trees 6
#44 MeSH descriptor: [Adenylosuccinate Lyase] explode all trees 0
#45 MeSH descriptor: [Angelman Syndrome] explode all trees 27
#46 MeSH descriptor: [Leukodystrophy, Globoid Cell] explode all trees 2
#47 MeSH descriptor: [Rett Syndrome] explode all trees 64
#48 (LCA* or SMA or DMD or XMTM or LGMD or CMT or PD or MPS or Ho-FH or DCM or CF or AATD or PCD):ti,ab,kw 54647
#49 ((retinal NEXT dystroph*) or "Retinitis Pigmentosa" or RP or (pigment* NEXT dystroph*) or "tapeto retinal degeneration" or "tapetoretinal degeneration" or "tapetoretinal dystrophy"):ti,ab,kw 3262
#50 (achromatognosia or achromatopia or achromatopsia or (colour NEXT blind*) or (color NEXT blind*) or "color agnosia" or "colour agnosia" or "scoterythrous vision" or "duchenne syndrome" or "duchenne type muscular dystrophy" or "duchenne muscular dystrophy" or "morbus duchenne" or "pseudo hypertrophic myopathic progressive muscular dystrophy"):ti,ab,kw 914
#51 ("backer muscular dystrophy" or "becker muscular dystrophy" or "dystrophic becker disease" or "becker dystrophy" or "becker type muscular dystrophy"):ti,ab,kw 84
#52 (sarcoglycanopath* or Friedreich* or "hereditary spinal ataxia" or "spinal hereditary ataxia" or "spinal heredoataxia"):ti,ab,kw 177
#53 ("cerebroside sulfatase deficiency syndrome" or "cerebroside sulfate storage disease" or "cerebroside sulphate storage disease" or (metachrom* NEXT leucodystrophy) or

(metachrom* NEXT leukodystrophy) or "sulfatide lipidosis" or sulfatidosis or "sulphatide lipidosis"):ti,ab,kw 9
#54 ("adenylosuccinate lyase" or adenylosuccinase or "adenylosuccinate amp lyase" or "adenylsuccinate lyase" or "e.c. 4.3.2.2" or "succinyladenylate lyase"):ti,ab,kw 1
#55 ("Angelman syndrome" or "happy puppet syndrome"):ti,ab,kw 42
#56 ("hereditary progressive spinal muscular atrophy" or "hereditary spinal progressive muscular atrophy" or "hoffmann werdnig" or "werdnig hoffman" or ((infantile or "type 1" or "type I") near/4 atrophy)):ti,ab,kw 33
#57 (Dubowitz or (("type 2" or "type II") near/4 atrophy)):ti,ab,kw 78
#58 (kugelberg* or ((benign or juvenile or "type 3" or "type III") near/4 atrophy)):ti,ab,kw 74
#59 ((girdle NEXT dystroph*) or ("limb-girdle muscular" NEXT dystroph*)):ti,ab,kw 41
#60 ("GM1 gangliosidosis" or "gangliosidosis G(M1)" or "gangliosidosis gm 1" or "gangliosidosis GM1" or "gm 1 gangliosidosis"):ti,ab,kw 7
#61 ("Aromatic l-amino acid decarboxylase" or "3,4 dihydroxy l phenylalanine carboxylyase" or "3,4 dihydroxyphenylalanine decarboxylase" or "5 hydroxy levo tryptophan carboxylyase" or "5 hydroxytrypthophan decarboxylase" or "5 hydroxytryptophan carboxylyase" or "5 hydroxytryptophan decarboxylase" or "5 hydroxytryptophane decarboxylase" or ("aromatic amino acid" NEXT decarboxylase*) or ("aromatic aminoacid" NEXT decarboxylase*) or "aromatic l amino acid carboxy lyase" or ("aromatic l amino acid" NEXT decarboxylase*) or "aromatic l aminoacid decarboxylase" or "aromatic levo aminoacid decarboxylase" or "dihydroxyphenylalanine decarboxylase" or "DOPA decarboxylase" or "e.c. 4.1.1.28" or "hydroxytryptophan decarboxylase" or "l 3,4 dihydroxyphenylalanine decarboxylase" or "l dopa decarboxylase" or "l tryptophan carboxylyase" or "levo dopa decarboxylase" or "levodopa decarboxylase" or "tryptophan decarboxylase"):ti,ab,kw 241
#62 (krabbe or "beta galactosidase deficiency syndrome" or "galactosylceramidase deficiency syndrome" or "galactosylceramide lipidosis" or "globoid cell dystrophy" or "globoid cell leucodystrophy" or "globoid cell leukodystrophy" or "mckusick 24520"):ti,ab,kw 8
#63 (rett or rett's):ti,ab,kw 121
#64 #13 or #14 or #15 or #16 or #17 or #18 or #19 or #20 or #21 or #22 or #23 or #24 or #25 or #26 or #27 or #28 or #29 or #30 or #31 or #32 or #33 or #34 or #35 or #36 or #37 or #38 or #39 or #40 or #41 or #42 or #43 or #44 or #45 or #46 or #47 or #48 or #49 or #50 or #51 or #52 or #53 or #54 or #55 or #56 or #57 or #58 or #59 or #60 or #61 or #62 or #63 125949
#65 MeSH descriptor: [Pharmacovigilance] explode all trees 42
#66 (Pharmacovigilan* or pharmaco-vigilan* or toxicit* or SUSAR or SAE or AR or SAR or (adverse NEXT reaction*) or (adverse NEXT event*) or antibodies or immunosuppress* or immunotherap* or "immune management" or immunomodulat* or ("subclinical immune" NEXT response*) or immunologic* or aminotransaminase* or ((liver or hepatic) NEAR/2 (damag* or injur* or inflamm*)) or leukemia or leukaemia or cancer* or tumor* or tumour* or humoral or "cellular immunity" or "innate immunity" or immunogenicity or "complement activation" or cytokine* or "pre-existing immunity" or ADA or cell-mediated or (binding NEXT antibod*) or (induced NEXT response*) or pre-existing or preexisting or boosted or (capsid NEXT response*) or redosing or (cell-mediated NEXT immun*) or "T-cell immunity" or tolerance or tolerization or TNF-alpha or prednisolone or eculizumab or "complement activation" or transaminitis or hypertransaminasemia or thrombocytopenia or microangiopathy):ti,ab,kw 526912
#67 MeSH descriptor: [Immunosuppressive Agents] explode all trees 6937

#68 MeSH descriptor: [Leukemia] explode all trees 6529
#69 MeSH descriptor: [Neoplasms] explode all trees 123839
#70 MeSH descriptor: [Chemical and Drug Induced Liver Injury] explode all trees 474
#71 MeSH descriptor: [Immunogenetic Phenomena] explode all trees 135
#72 MeSH descriptor: [Cytokines] explode all trees 27506
#73 MeSH descriptor: [Prednisolone] explode all trees 5996
#74 MeSH descriptor: [Thrombocytopenia] explode all trees 1790
#75 MeSH descriptor: [Ganglia, Spinal] explode all trees 71
#76 MeSH descriptor: [Anemia, Hemolytic] explode all trees 1840
#77 MeSH descriptor: [Acute Kidney Injury] explode all trees 2347
#78 (TMA or "dorsal root ganglion" or "dorsal root spinal ganglion" or "spinal ganglia" or "spine ganglion" or DRG or "hemolytic anemia" or "hemolytic anaemia" or "haemolytic anemia" or "haemolytic anaemia" or "acute kidney damage" or "acute kidney injury" or "acute kidney failure" or "acute renal damage" or "acute renal failure" or ("elevated liver" NEXT enzyme*) or ("elevated hepatic" NEXT enzyme*) or hepatotoxic* or (hepato NEXT toxic*) or ("liver cell" NEXT toxicit*) or "liver intoxication" or (liver NEXT poison*) or (liver NEXT toxic*) or ("abnormal T2" NEXT hyperintensit*)):ti,ab,kw 11367
#79 #65 or #66 or #67 or #68 or #69 or #70 or #71 or #72 or #73 or #74 or #75 or #76 or #77 or #78 560567
#80 #3 and #12 and #64 and #79 732
#81 (realworld* OR (real NEXT world*)):ti,ab,kw 8856
#82 (observational OR (case NEXT report*)):ti,ab,kw 37146
#83 #81 or #82 44896
#84 #80 and #83 32
