# Supplementary material for: A systematic review of immunosuppressive protocols used in AAV gene therapy for monogenic disorders
Source: Mol Ther. 2024 Jul 22;32(10):3220–59. doi: 10.1016/j.ymthe.2024.07.016 (PMC11489562; doi:10.1016/j.ymthe.2024.07.016)
Supplement: Document S2. Article plus supplemental information [file mmc2.pdf]

Molecular Therapy
Review

# A systematic review of immunosuppressive protocols used in AAV gene therapy for monogenic disorders

Besarte Vrellaku,[1,11] Ilda Sethw Hassan,[2,11] Rebecca Howitt,[3,11] Christopher P. Webster,[2] Eli Harriss,[4] Fraser McBlane,[5] Corinne Betts,[1] Jorge Schettini,[1] Mattia Lion,[6,10] John E. Mindur,[6,10] Michael Duerr,[7] Pamela J. Shaw,[2] Janine Kirby,[2] Mimoun Azzouz,[2,8,10] and Laurent Servais[1,9,10]

[1]Department of Paediatrics, MDUK Oxford Neuromuscular Centre & NIHR Oxford Biomedical Research Centre, University of Oxford, Oxford, UK; [2]Sheffield Institute for Translational Neuroscience, Division of Neuroscience, School of Medicine and Population Health, University of Sheffield, Sheffield, UK; [3]The Queen's College, University of Oxford, Oxford, UK; [4]Bodleian Health Care Libraries, University of Oxford, Oxford, UK; [5]Novartis Pharma AG, Basel, Switzerland; [6]Takeda Pharmaceuticals USA, Inc, Cambridge, MA, USA; [7]Bayer Aktiengesellschaft, CGT&Rare Diseases, Leverkusen, Deutschland; [8]Gene Therapy Innovation & Manufacturing Centre (GTIMC), University of Sheffield, Sheffield, UK; [9]Division of Child Neurology, Department of Paediatrics, Centre de Référence des Maladies Neuromusculaires, University Hospital Liège and University of Liège, Liège, Belgium

**The emergence of adeno-associated virus (AAV)-based gene therapy has brought hope to patients with severe monogenic disorders. However, immune responses to AAV vectors and transgene products present challenges that require effective immunosuppressive strategies. This systematic review focuses on the immunosuppressive protocols used in 38 clinical trials and 35 real-world studies, considering a range of monogenic diseases, AAV serotypes, and administration routes. The review underscores the need for a deeper understanding of immunosuppressive regimens to enhance the safety and effectiveness of AAV-based gene therapy. Characterizing the immunological responses associated with various gene therapy treatments is crucial for optimizing treatment protocols and ensuring the safety and efficacy of forthcoming gene therapy interventions. Further research and understanding of the impact of immunosuppression on disease, therapy, and route of administration will contribute to the development of more effective and safer gene therapy approaches in the future.**

## INTRODUCTION

Gene therapy clinical trials using adenovirus (AdV) or adeno-associated virus (AAVs) as delivery systems have been ongoing since the late 1990s.[1–3] The first phase 1 clinical trial of AdV-based gene therapy in 1996 delivered the cystic fibrosis transmembrane conductance regulator (*CFTR*) gene to patients with cystic fibrosis.[2] However, in 1999, gene therapy research was abruptly halted due to the death of Jesse Gelsinger after treatment for ornithine-transcarbamylase (OTC) hepatic enzyme deficiency with AdV-based gene therapy.[4] After Gelsinger received an injection of an AdV vector carrying a wild-type version of the OTC enzyme and died soon after, an immediate review was prompted which raised questions regarding the safety profile of viral vectors. It was found that blood samples from Jesse Gelsinger contained high levels of pre-existing antibodies against AdV serotype 5, and that these antibodies were able to enhance innate immune responses (in particular dendritic cells) resulting in such an extreme, and ultimately fatal, inflammatory response.[5] This led eventually to the use of AAV rather than AdV. In 2012, alipogene tiparvovec was the first approved AAV-mediated gene therapy in Europe for treating hereditary lipoprotein lipase deficiency using an AAV1 vector.[6] After extensive clinical development, in 2017, voretigene neparvovec (VN; AAV2-hRPE65v2) received the U.S. Food and Drug Administration (FDA) approval for pediatric patients with RPE65-associated Leber congenital amaurosis (LCA) and confirmed biallelic RPE65-mediated retinal dystrophy, which are conditions that result in progressive vision loss, making it the first-ever FDA-approved gene therapy.[7–10] Similarly, onasemnogene abeparvovec (OA), an AAV serotype 9 (AAV9)-based gene therapy for pediatric patients with spinal muscular atrophy (SMA), was first made available in the United States in 2019 before its approved use in more than 40 countries.[11] Rapid and early benefits of OA were demonstrated in symptomatic patients with infantile-onset SMA in the phase 3 STR1VE[12] and STR1VE-EU[13] trials, with evidence of sustained and durable efficacy, as well as a favorable long-term safety profile as shown in the 5-year extension of the phase 1 trial, START.[14] Valoctocogene roxaparvovec and etranacogene dezaparvovec were approved for the treatment of hemophilia A and B in 2023 and 2022, respectively.[15,16] Recently, the FDA approved fidancogene

https://doi.org/10.1016/j.ymthe.2024.07.016.

[10]These authors contributed equally

[11]These authors contributed equally

**Correspondence:** Mimoun Azzouz, Sheffield Institute for Translational Neuroscience, Division of Neuroscience, School of Medicine and Population Health, University of Sheffield, Sheffield, UK.
**E-mail:** m.azzouz@sheffield.ac.uk
**Correspondence:** Laurent Servais, Department of Paediatrics, MDUK Oxford Neuromuscular Centre & NIHR Oxford Biomedical Research Centre, University of Oxford, Oxford, UK.
**E-mail:** laurent.servais@paediatrics.ox.ac.uk

 © 2024 The Authors.
Published by Elsevier Inc. on behalf of The American Society of Gene and Cell Therapy.
This is an open access article under the CC BY license (http://creativecommons.org/licenses/by/4.0/).

elaparvovec for adult patients with moderate to severe hemophilia B. In addition, delandistrogene moxeparvovec-rokl is the first gene therapy approved by the FDA for treatment of Duchenne muscular dystrophy (DMD).[17,18]

The transfer of DNA into specific cell types thus constitutes an elegant and attractive approach to target the root cause of disease in individuals who present with a severe genetic condition—mostly rare and severe monogenic disorders with haploinsufficiency. To date, the vast majority of clinical developments in gene therapy rely on AAVs, primarily for the replacement or addition of genes.[19,20]

Despite recent successful regulatory approvals, one of the challenges that remains for further successful implementation of AAV-based gene therapies is overcoming immune responses geared toward the vector or transgene. Such immune responses can lead to loss of treatment efficacy over time and can also result in severe and sometimes fatal toxicities in treated patients. Toxicities often manifest as elevated liver enzymes due to the body's immune response against the vector components, such as the capsid or expression cassette as well as the transgene product, resulting in liver inflammation and subsequent damage to liver cells, as indicated by alanine aminotransferase (ALT) and aspartate aminotransferase (AST) release. Additionally, patients may experience thrombotic microangiopathy (TMA), including kidney injury, as well as cardiotoxicity.[21,22] Although OA has been delivered in more than 1,800 SMA patients worldwide and demonstrates clear evidence of clinically meaningful efficacy,[12,23] especially when administered early in young infants,[24] several adverse events (AEs) related to the host immune response and complement activation elicited by AAV capsid proteins have been reported.[21,25–27] A study reported the first fatal TMA case following administration of OA in a 6-month-old child with SMA type 1[25] who was a carrier of a potential genetic predisposition in the complement factor I gene. The finding of severe TMA is likely due to complement recognition of the AAV capsid following OA therapy, demonstrating the broad impact of systemic AAV on immune activation and the need for dosing protocols to add immunosuppression to avoid these AEs.

Recent strategies to evade detrimental immune responses to AAV exposure include the engineering of AAV by replacing the viral genome with a therapeutic expression cassette containing inverted terminal repeats, promoters and enhancers, and a codon-optimized gene of interest, while also modifying capsid sequences to yield a recombinant AAV (rAAV) that decreases immunogenicity and prolongs transduction in host cells.[28] However, the challenge of overcoming host immunity to better enable gene therapy remains. While AAV infection is considered non-pathogenic in humans, initial exposure to AAV induces cellular and humoral immune responses against rAAV due to capsid similarity.[29,30] Although AAV has been known to be a non-pathogenic virus, recent reports of cases of acute severe hepatitis in children have challenged the idea that AAV is a harmless virus.[31] More specifically, three independent studies published in March 2023 demonstrated that infection with AAV2 was linked to recent clusters of unexplained acute severe hepatitis in children.[31–34]

Roughly 30%–70% of the general population have pre-existing neutralizing antibodies (NAbs) against various serotypes, including AAV1, AAV2, AAV5, AAV6, AAV8, and AAV9, depending on the geographical location, health status, and assay type, among other factors across studies.[34–36] Immune responses against AAV have also been observed across different monogenic disorders, causing the various adverse reactions described above. In addition, immune responses can sometimes hinder therapeutic effects by eliciting antibodies against AAV capsids, which reduces the expression of the transgene product. This reduction in expression can also occur if transduced cells are killed by cytotoxic T cells or if there are immune responses against the transgene product itself. Humoral immune responses, measured by different assays as either NAbs and total antibodies (TAbs), can create an immune response barrier to successful AAV transduction.[37,38] NAbs can neutralize capsids, either by interfering with intracellular processes that lead to capsid uncoating or by blocking key epitopes needed for receptor-mediated uptake into target cells, thereby decreasing the efficacy of gene transfer therapy.[39–41] Opsonization is another mechanism by which anti-AAV TAbs may impact AAV gene therapy treatment efficacy.[42] Furthermore, T cell responses may eliminate transgene-expressing cells, resulting in hepatotoxicity and loss of transgene expression, as seen in several clinical trials.[38,43,44] As such, the host immune response is an important factor to monitor and temper after gene therapy as it may relate to both the treatment's safety and efficacy.

Extensive efforts to suppress immune responses to AAV have been undertaken across trials. Corticosteroids such as methylprednisolone, prednisone, and prednisolone are widely used in immunosuppressive protocols for inhibiting immune responses to AAV by the decrease of proinflammatory cytokines/chemokines and attenuating liver toxicity.[8,11,45] Early trials of AAV gene therapy used a reactive approach for administering corticosteroids in response to instances of elevated liver enzymes, which were thought, in certain cases, to be linked to an AAV capsid-specific cytotoxic T cell response indicative of liver injury.[43,44] Corticosteroid treatment typically resolves the elevation of liver transaminases.[12,43] Subsequent clinical trials incorporated prophylactic immunosuppression regimens that included one or a combination of pharmacotherapies. Corticosteroids bind to glucocorticoid receptors and modify transcriptional signaling that results in global anti-inflammatory and immunosuppressive effects.[46] Corticosteroids exert these effects through multiple mechanisms including downregulation of Toll-like receptor expression, suppression of proinflammatory cytokines, and upregulation of anti-inflammatory cytokines.[47]

Other immunosuppressants used in AAV gene therapies include sirolimus, mycophenolate mofetil, calcineurin inhibitors and rituximab. Mycophenolate mofetil inhibits T and B cell proliferation by targeting type II inosine monophosphate dehydrogenase, thus suppressing both cell-mediated and humoral immune responses.[15,45,48–50]

Sirolimus is also used in AAV clinical trials for immune suppression (e.g., NCT02240407) and works by inhibiting T and B cell activation and induces regulatory T cells (Tregs) through mammalian target of rapamycin (mTOR) targeting.[50]

Calcineurin inhibitors such as tacrolimus exert their immunosuppressive effects by suppressing the production of proinflammatory cytokines such as IL-2, thereby inhibiting T cell activation and proliferation and inhibition of T helper cell-dependent B cell response.[51–53] AAV clinical trials also use a combination of immunosuppressant therapies, either administered before or at the time of AAV dosing, and these are continued after AAV dosing. The combination of rituximab, sirolimus, and corticosteroids was used in a recent clinical trial for GM2-gangliosidosis through CNS-directed AAV delivery[3] with no vector-related AEs observed. Moreover, patients received glucocorticoids with or without tacrolimus to decrease the risk of vector-related immune responses.[48]

While the use of immunosuppressive regimens affords some level of control over immune responses to AAV gene therapy, these protocols also come with an increased risk of infection or viral reactivation, especially in patients who may already be in a severe clinical condition. This can question the safety and/or benefit ratio of immunosuppression—for instance, when low doses of AAV are injected locally or in a well-delineated space. Despite the importance of an adequate immunosuppressive regimen, there is no consensus or specific guidelines on which regimen is the most appropriate in terms of risk versus benefit ratio, either generally or more specifically in regard to AAV dose, serotype, route of administration, and any pre-existing or underlying condition(s). To our knowledge, no formal or informal comparison of the different immunosuppressive regimens has been conducted.

The aim of this review was to systematically catalog the various immunosuppressive protocols used across various AAV gene therapy trials, and to map these according to monogenic disease, gene therapy treatment (including vector serotype), and route of administration. Additionally, clinical trials will be mapped according to serious adverse reactions, including biological biomarkers (e.g., AST, ALT, thrombocytopenia, and lactate dehydrogenase). We also describe methods for treating immune responses and evaluate the efficacy of these protocols.

## RESULTS

### Study characteristics

Table 1 depicts a summary of the 38 AAV clinical trials assessed in this review. The included studies were mainly conducted in the United States ($n$ = 28), the UK ($n$ = 8), Germany ($n$ = 6), Australia ($n$ = 5), and France ($n$ = 5), with 15 other countries also contributing to these clinical trials (Table 1). The 38 studies were conducted between 2008 and 2024, but more than 50% of these studies were conducted between 2021 and 2023 (Figure 1A). Although more than 15 different diseases were included in these clinical trials, the most common were hemophilia B ($n$ = 8), SMA ($n$ = 7), LCA ($n$ = 5), DMD ($n$ = 5), and hemophilia A ($n$ = 3).

### Types of AAV and routes of administration

A total of 15 different AAV serotypes were used, with the most common being AAV9 (18%; $n$ = 7) (Figure 1B). In 58% of cases, these AAV vectors were administered via intravenous injection or infusion ($n$ = 22) (Figure 1C). However, the route of administration was somewhat dependent on the disease being treated and, therefore, the target tissue. For example, in clinical trials for ocular conditions, including LCA, Leber hereditary optic neuropathy, macular degeneration, severe early childhood onset retinal dystrophy, X-linked retinitis pigmentosa, and X-linked retinoschisis, the gene therapy vectors were administered directly into the eye via intravitreal or subretinal injection, whereas all studies relating to hemophilia A and B used intravenous delivery as the preferred route of administration. Given that these 38 studies cover more than 15 different diseases and 15 different AAV serotypes, the doses of each clinical vector are varied and depend on the disease and vector type. The highest single dose in each trial is listed in Table 1.

### Prophylactic immunosuppression

#### *Clinical studies*

When considering the immunosuppressive treatment used in relation to these AAV gene therapies, in 74% of studies, patients received prophylactic immunosuppression ($n$ = 28) either before (up to 2 weeks in advance, though more commonly 24–72 h beforehand), on the day of, or immediately after AAV gene therapy administration (Figure 1D). In the remaining 26% of cases ($n$ = 10), immunosuppressive treatments were used in response to a range of AEs and serious AEs (SAEs) associated with the gene therapy product (Table 1). A total of 8 different immunosuppressants are detailed in the 38 identified studies (Figure 1D). The most common immunosuppressants administered were corticosteroids, either prednisolone, its prodrug prednisone, or methylprednisolone. Corticosteroids were used either on their own, or in conjunction with other immunosuppressants, in 95% (36/38) of the identified studies. Other immunosuppressants listed in more than one of the identified studies were tacrolimus (5/34 studies) and mycophenolate mofetil (5/34 studies). Cyclosporine was mentioned in a single study only, as was a combination of sirolimus and rituximab (Figure 1E). Where specified, the dose of prednisolone ranged from 1 to 2 mg/kg, with a total daily dose in the range of 30–60 mg. In nearly all cases, prednisolone was administered in a tapering dose over a period of days and weeks, with stated durations ranging from 7 to 133 days. Nine studies (26%) had to increase the dose or introduce additional immunosuppressive agents beyond the initial protocol in response to immunogenicity that occurred in individual patients.

#### *Real-world evidence*

Table 2 depicts a summary of the 35 real-world studies in this review with the most common studies being SMA ($n$ = 31), inherited retinal dystrophy ($n$ = 3), and hemophilia ($n$ = 1). These real-world outcomes support findings from the interventional trial program and demonstrate the effectiveness of OA in a large patient population, which was consistent with initial clinical data and published 5-year follow-up data. Observed AEs were consistent with the established

**Table 1. Overview of AAV gene therapy clinical trials**

| No. | Disease | AAV gene therapy | No. of participants and age ranges | Route | Highest dose | Vector type | Immunosuppressive protocol | Prophylactic or reactive regimen | Clinical evidence of immunosuppression effectiveness | NCT (phase) | Reference | Country |
|---|---|---|---|---|---|---|---|---|---|---|---|---|
| 1 | hemophilia A | valoctocogene roxaparvovec | *n* = 134; age range 18–70 years | intravenous | $6 \times 10^{13}$ vg/kg | AAV5 | prednisone or prednisolone tapering dose of 60 mg per day for 8 weeks other immunosuppressants were used by 39 participants (29.1%) because of contraindications, side effects, or a poor or no response to glucocorticoid treatment (budesonide, tacrolimus, mycophenolate, methylprednisolone) | reactive | reduced ALT levels | NCT03370913 (3) | Mahlangu et al., 2023, Ozelo et al., 2022[15,137] | USA Australia Belgium Brazil France Germany Israel Italy Republic of Korea South Africa Spain Taiwan UK |
| 2 | hemophilia A | valoctocogene roxaparvovec | *n* = 9 age >18 years | intravenous | $6 \times 10^{13}$ vg/kg | AAV5 | prednisolone, tapering dose of 60 mg/kg for 2 weeks, then down to 5 mg/day for 1 week | prophylactic | reduced ALT levels | NCT02576795 (1/2) | Rangarajan et al., 2017, Long et al., 2021, Fong et al., 2022, Pasi et al., 2021[59,67,81,85] | UK |
| 3 | hemophilia A | giroctocogene fitelparvovec | *n* = 11 (≥18 years) | intravenous | $3 \times 10^{13}$ vg/kg | AAV2/6 | prednisone, 60 mg with dose tapering to 30 mg, 15 mg, and 5 mg/d | reactive | reduced ALT levels | NCT03061201 (1/2) | Leavitt et al., 2024[104] | USA |
| 4 | hemophilia B | BBM-H901 | *n* = 10 age >18 years | intravenous | $5 \times 10^{12}$ vg/kg | dsAAV843 | prednisone, 1 mg/kg | prophylactic | reduced proportion of cytotoxic T cells downregulated percentage of $CD16^{+}$ monocytes and dendritic cells | NCT04135300 (1) | Xue et al., 2022[63] | China |
| 5 | hemophilia B | etranacogene dezaparvovec | *n* = 54 age >18 years | intravenous | $2.10 \times 10^{13}$ vg/kg | AAV5 | prednisolone or methylprednisolone or prednisone, starting dose 60 mg with tapering until 5 mg/week | reactive | N/A | NCT03569891 (3) | Pipe et al., 2023[16] | USA Belgium Denmark Germany Ireland The Netherlands Sweden UK |
| 6 | hemophilia B | verbrinacogene setparvovec | *n* = 10 age >18 years | intravenous | $1.28 \times 10^{12}$ vg/kg | AAV2/S3 | prednisolone with or without tacrolimus, methylprednisolone at varying doses | prophylactic and reactive | N/A | NCT03369444 and NCT03641703 (1/2) | Chowdary et al., 2022[48] | USA Ireland Italy UK |
| 7 | hemophilia B | AMT-060 | *n* = 10 age >18 years | intravenous | $2 \times 10^{13}$ gc/kg | AAV5 | prednisolone | reactive | reduced ALT levels | NCT02396342 (1/2) | Majowicz et al., 2019, Miesbach et al., 2018[68,86] | Denmark Germany The Netherlands |

(*Continued on next page*)

**Table 1. *Continued***

| No. | Disease | AAV gene therapy | No. of participants and age ranges | Route | Highest dose | Vector type | Immunosuppressive protocol | Prophylactic or reactive regimen | Clinical evidence of immunosuppression effectiveness | NCT (phase) | Reference | Country |
|---|---|---|---|---|---|---|---|---|---|---|---|---|
| 8 | hemophilia B | SPK-9001 | $n = 15 \geq 18$ years | intravenous | $2 \times 10^{12}$ vg/kg | a novel, bioengineered single-stranded adeno-associated viral vector carrying human FIX variant | prednisolone, 60 mg | reactive | reduced ALT levels | NCT02484092 (1/2) | George et al., 2017[105] | USA Australia |
| 9 | hemophilia B | BAX 335 (scAAV8.FIXR338) | $n = 30$ 20 to 69 years | intravenous | $3 \times 10^{12}$ vg/kg | AAV8 | prednisone, 2.6–60 mg with dose tapering | reactive | although corticosteroid therapy was associated with immediate normalization of the IFN-γ ELISpot in participant 6, this signal remained elevated for weeks after the initiation of prednisone in participant 7 systemic corticosteroid administration initiated in response to ALT elevations in participants 6 and 7, and as prophylaxis in participant 8, did not stabilize FIX activity levels in these participants | NCT01687608 (1/2) | Konkle et al., 2021[82] | USA |
| 10 | hemophilia B | scAAV2/8-LP1-hFIXco | $n = 14 \geq 18$ years | intravenous | $2 \times 10^{12}$ vg/kg | scAAV2/8 | prednisolone, 60 mg with dose tapering | reactive | reduced ALT levels | NCT00979238 (1/2) | Nathwani et al., 2011, Nathwani et al., 2014[43,44] | USA UK |
| 11 | hemophilia B | etranacogene dezaparvovec (ATM-061) | $n = 3$ (ages 43, 47, and 50 years) | intravenous | $2 \times 10^{13}$ gc/kg | AAV5 | prednisone, 50 mg for 5 days starting at day 94 for bronchitis treatment in 1 patient | reactive | N/A | NCT03489291 (2b) | Von Drygalski et al., 2019[106] | USA |
| 12 | DMD | rAAV2.5-CMV-minidystrophin (d3990) | $n = 6$; 5–11 years | intravenous | $3 \times 10^{12}$ vg | AAV2.5 | methylprednisolone, 2 mg/kg but limited to <1 g in total | prophylactic | IFN-γ levels | NCT00428935(1) | Bowles et al., 2012[102] | USA |
| 13 | DMD | delandistrogene moxeparvovec | $n = 4$; 4 to 7 years | intravenous | $2 \times 10^{14}$ vg/kg | rAAVrh74 | prednisone, 1 mg/kg with tapering over 30 days | prophylactic | liver enzyme elevation returned to normal after corticosteroids | NCT03375164 (1 and 2) | Mendell et al., 2020[66] | USA |
| 14 | DMD | delandistrogene moxeparvovec | $n = 41$; age range 4–8 years | intravenous | $2 \times 10^{14}$ vg/kg | rAAVrh74 | prednisone or prednisolone, 1 mg/kg daily | prophylactic | N/A | NCT03769116 (2) | Mendell et al., 2023[57] | USA |
| 15 | DMD | delandistrogene moxeparvovec | $n = 20$; age range ≥4 to <8 years | intravenous | $1.33 \times 10^{14}$ vg/kg | rAAVrh74 | prednisone or prednisolone, 1 mg/kg daily | prophylactic | N/A | NCT04626674 (1) | Zaidman et al., 2023[103] | USA |

(*Continued on next page*)

**Table 1. *Continued***

| No. | Disease | AAV gene therapy | No. of participants and age ranges | Route | Highest dose | Vector type | Immunosuppressive protocol | Prophylactic or reactive regimen | Clinical evidence of immunosuppression effectiveness | NCT (phase) | Reference | Country |
|---|---|---|---|---|---|---|---|---|---|---|---|---|
| 16 | SMA | OA | *n* = 76; mean age 16.8 | intravenous | $1 \times 10^{14}$ vg/kg | AAV9 | prednisolone, 1 mg/kg | prophylactic | N/A | N/A (observational study) | Weiβ et al., 2022[83] | Germany Austria |
| 17 | SMA | OA | *n* = 15; median age 32 days (range, 9–43) | intravenous | $1 \times 10^{14}$ vg/kg | AAV9 | prednisolone, initially 1 mg/kg/day, then increased to 2 mg/kg/day following protocol amendments | prophylactic | no SAEs related to gene therapy product | NCT03505099 (3) | Servais et al., 2023[22] | USA Australia Belgium Canada Japan UK |
| 18 | SMA | OA | *n* = 21; age range, 0.5–24 months | intravenous | $1 \times 10^{14}$ vg/kg | AAV9 | prednisolone, 1 mg/kg/day | prophylactic | hypertension | N/A (cohort study) | D'Silva et al., 2022[109] | Australia |
| 19 | SMA | OA | *n* = 15 up to 6 months | intravenous | $2.4 \times 10^{14}$ vg | AAV9 | prednisolone, 1 mg/kg/day | prophylactic | reduced ALT and AST levels | NCT02122952 (1) | Mendell et al., 2017[11] | USA |
| 20 | SMA | OA | *n* = 8 age range 10–37 months | intravenous | $1.1 \times 10^{14}$ vg | AAV9 | prednisolone, 1 mg/kg/day | prophylactic | increased transaminases typically responded to steroid treatment | N/A (retrospective analysis) | Friese et al., 2021[65] | Germany |
| 21 | SMA | OA | *n* = 22 up to 180 days | intravenous | $1.1 \times 10^{14}$ vg | AAV9 | prednisolone, 1 mg/kg/day | prophylactic | prevented elevation of ALT levels | NCT03306277 (3) | Day et al., 2021, Mercuri et al., 2021[12,13] | USA |
| 22 | X-linked myotubular myopathy | resamirigene bilparvovec | *n* = 26; age range, 10.0–64.7 months | intravenous | $3.5 \times 10^{14}$ vg/kg | AAV8 | prednisolone (1 mg/kg) daily | prophylactic | N/A | NCT03199469 (2/3) | Shieh et al., 2023[56] | USA Canada France Germany |
| 23 | LCA2 | AAV2-hRPE65v2 | *n* = 12; ≥18 years | subretinal/intraorbital | $1.5 \times 10^{11}$ vg | AAV2 | prednisone, 1 mg/kg/day for 10 days, followed by 0.5 mg/kg/day for 7 days | prophylactic | reduced NAb levels | NCT00516477 (1) | Maguire et al., 2008, Simonelli et al., 2010[7,10] | USA |
| 24 | LCA2 | rAAV2-CBSB-hRPE65 | *n* = 3; ages 21, 23, and 24 years | subretinal/intraorbital | $5.96 \times 10^{10}$ vg | AAV2 | steroids | reactive | N/A | NCT00481546 (1) | Hauswirth et al., 2008[69] | USA |
| 25 | LHON | scAAV2-P1ND4v2 | *n* = 28; 16–56 years | intravitreal | $1 \times 10^{10}$ vg/eye | scAAV2 | prednisolone | reactive | management of uveitis | NCT02161380 (1) | Lam et al., 2022[107] | USA |
| 26 | age-related macular degeneration | rAAV.sFLT-1 | *n* = 40; ≥55 years | subretinal/intraorbital | $1 \times 10^{11}$ | AAV2 | prednisolone | prophylactic | N/A | NCT01494805 (1) | Rakoczy et al., 2015[84] | Australia |
| 27 | RPE65-deficient LCA and severe early childhood-onset retinal dystrophy | rAAV2-CB-hRPE65 | *n* = 12; 6 to 39 years | subretinal/intraorbital | $6 \times 10^{11}$ vg | AAV2 | topical corticosteroids | prophylactic | postoperative treatment with topical corticosteroids and antibiotics<br>no enzyme-linked immunospot response to transgene or capsid<br>no vector DNA in the blood | NCT00749957 (1/2) | Weleber et al., 2016[108] | USA |

(*Continued on next page*)

Table 1. *Continued*

| No. | Disease | AAV gene therapy | No. of participants and age ranges | Route | Highest dose | Vector type | Immunosuppressive protocol | Prophylactic or reactive regimen | Clinical evidence of immunosuppression effectiveness | NCT (phase) | Reference | Country |
|---|---|---|---|---|---|---|---|---|---|---|---|---|
| 28 | RPE65-mediated inherited retinal dystrophy (LCA) | AAV8-coRPGR codon optimized RPGR | *n* = 18; ≥18 years | subretinal/intraorbital | $5 \times 10^{12}$ vp/mL | AAV8 | prednisolone; 1 mg/kg at start of GT; 60–30 mg/day upon acute inflammation | prophylactic | the subretinal inflammation seems to have resolved in all cases by 6 months after a course of oral corticosteroids. The inflammation seemed to have resolved in all cases by 6 months when all patients had creased oral corticosteroid treatment<br>no patient required secondary immunosuppressive therapy | NCT03116113 (1/2) | Cehajic-Kapetanovic et al., 2020[60] | USA<br>UK |
| 29 | RPE65-mediated inherited retinal dystrophy (LCA) | VN (AAV2-hRPE65v2) | *n* = 31; 4–44 years | subretinal/intraorbital | $1.5 \times 10^{11}$ vg | AAV2 | prednisone; 1 mg/kg/day, maximum dose 40 mg/day and tapered until 3 days before injection of the second eye when the steroid regimen was repeated | prophylactic | N/A | NCT00999609 (3) | Russell et al., 2017[8] | USA |
| 30 | X-linked retinitis pigmentosa | AAV8-RS1 | *n* = 11; 23–72 years | subretinal/intraorbital | $3 \times 10^{11}$ vg | AAV8 | cyclosporine: 175 mg twice daily; mycophenolate mofetil at 500 and 1,000 mg twice daily; prednisone at 60 mg | prophylactic | not efficacious | NCT02317887 (1/2) | Mishra et al., 2021[71] | USA |
| 31 | X-linked retinoschisis | cotoretigene toliparvovec | *n* = 18; 20.7–50.7 years | subretinal/intraorbital | $5 \times 10^{11}$ vg | AAV8 | preoperative treatment: 1 mg/kg/day of prednisolone (beginning 2 days before gene therapy, on the day of surgery and for 7 days afterward) followed by 0.5 mg/kg/day for 7 days, 0.25 mg/kg/day for 2 days, and 0.125 mg/kg/day for 2 days<br><br>postoperative (additional) treatment: prednisolone: 60 mg daily with tapering; dexamethasone: 0.1% or 0.7 mg | prophylactic | 1 case of reduced visual acuity resolved with corticosteroids but 1 case did not as there had been loss of central photoreceptors | NCT03116113 (1) | von Krusenstiern et al., 2023[110] | USA<br>UK |

(*Continued on next page*)

**Table 1. *Continued***

| No. | Disease | AAV gene therapy | No. of participants and age ranges | Route | Highest dose | Vector type | Immunosuppressive protocol | Prophylactic or reactive regimen | Clinical evidence of immunosuppression effectiveness | NCT (phase) | Reference | Country |
|---|---|---|---|---|---|---|---|---|---|---|---|---|
| 32 | DMD | rAAVrh74.MCK.GALGT2 | *n* = 2; 6.9 and 8.9 years | isolated limb infusion: injection into the femoral artery of both legs | $1 \times 10^{14}$ vg/kg | rAAVrh74 | prednisone, 1 mg/kg/day | prophylactic | N/A | NCT03333590 (1/2) | Flanigan et al., 2022[64] | USA |
| 33 | LHON | scAAV2-P1ND4v2 | *n* = 28; 16–56 years | intravitreal | $1 \times 10^{10}$ vg/eye | scAAV2 | prednisolone | reactive | management of uveitis | NCT02161380 (1) | Lam et al., 2022[107] | USA |
| 34 | limb-girdle muscular dystrophy | scAAVrh74.tMCK.hSGCA | *n* = 6; 8–13 years | intravascular | $3 \times 10^{12}$ vg/kg | AAVrh74 | prednisone, 1 mg/kg/day | prophylactic | N/A | N/A (1/2) | Mendell et al., 2019[80] | USA |
| 35 | MPS type IIIA | AAVrh.10-MPS3A | *n* = 4 (patients 1–3, aged between 5.5 and 6 years; patient 4 aged 2 years 8 months | intracerebroventicular | $7.2 \times 10^{11}$ vg | AAVrh.10 | tacrolimus - 0.2 mg/kg/day<br><br>mycophenolate mofetil, 1,200 mg/$m^2$ initially, adapted to obtain $AUC_{0-12h}$ > 30 mg g/L at 7 days post-treatment<br><br>prednisolone - 1 mg/kg/day | prophylactic | N/A | NCT01474343 (1/2) | Tardieu et al., 2014, Tardieu et al., 2017[45;58] | France |
| 36 | MPS type IIIB | rAAV2/5-hNaGlu | *n* = 4; 20, 26, 30, and 53 months | intracerebroventicular | $4 \times 10^{12}$ vg | AAV2/5 | tacrolimus - 0.2 mg/kg/day<br>mycophenolate mofetil, 1,200 mg/$m^2$/day<br>prednisolone - 1 mg/kg/day | prophylactic | N/A | EudraCT, number 2012-000856-33, and the International Standard Clinical Trial Registry, number ISRCTN19853672 (1/2) | Tardieu et al., 2017[58] | France |
| 37 | MPS type IIIB | rAAV2/5-hNAGLU | *n* = 4; 18–60 months | intraparenchymal | $4 \times 10^{12}$ vg | AAV2/5 | tacrolimus - 0.2 mg/kg/day<br>mycophenolate mofetil, 1,200 mg/$m^2$/day | prophylactic | N/A | NCT03300453 (1/2) | Gougeon et al., 2021, Deiva et al., 2021[49,50] | France |
| 38 | RPE65-Deficient LCA and severe early childhood-onset retinal dystrophy | rAAV2-CB-hRPE65 | *n* = 12; 6–39 years | subretinal/intraorbital | $6 \times 10^{11}$ vg | AAV2 | Topical corticosteroids | prophylactic | postoperative treatment with topical corticosteroids and antibiotics<br>no enzyme-linked immunospot response to transgene or capsid<br>no vector DNA in the blood | NCT00749957 (1/2) | Weleber et al., 2016[108] | USA |

(*Continued on next page*)

**Table 1. *Continued***

| No. | Disease | AAV gene therapy | No. of participants and age ranges | Route | Highest dose | Vector type | Immunosuppressive protocol | Prophylactic or reactive regimen | Clinical evidence of immunosuppression effectiveness | NCT (phase) | Reference | Country |
|---|---|---|---|---|---|---|---|---|---|---|---|---|
| 39 | RPE65-mediated inherited retinal dystrophy (LCA) | VN (AAV2-hRPE65v2) | $n$ = 31; 4 to 44 years | subretinal/intraorbital | $1.5 \times 10^{11}$ vg | AAV2 | prednisone; 1 mg/kg/day, maximum dose 40 mg/day and tapered until 3 days before injection of the second eye when the steroid regimen was repeated | prophylactic | N/A | NCT00999609 (3) | Russell et al., 2017[8] | USA |
| 40 | SMA | OA | $n$ = 32; 7–55 months) | intrathecal | $2.4 \times 10^{14}$ vg | AAV9 | prednisolone, 1 mg/kg/day | prophylactic | N/A | NCT03381729 (1) | Finkel et al., 2023[70] | USA |
| 41 | Tay-Sachs disease | AAVrh8-HEXA and AAVrh8-HEXB | $n$ = 2; 7 and 30 months | bilateral thalamic injection | $4.2 \times 10^{13}$ vg | AAVrh8 | patients received a regimen that included rituximab (375 mg $m^{-2}$), intravenous infusion of methylprednisolone (10 mg kg$-^{1}$) and sirolimus (1 mg $m^{-2}$). prednisone (2 mg $kg^{-1}$ per day) was administered for 90 days, and sirolimus was maintained at 3–7 ng $mL^{-}1$ for 180 days, both followed by a 1-month taper. | prophylactic | B cell levels decreased to <1% of total lymphocytes intravenous immunoglobulin was given as needed to maintain serum levels between 700 and 1,000 mg $dL^{-1}$ a single dose of rituximab resulted in a reduction in B cell counts for >6 months in each patient | N/A (expanded-access clinical trial) | Flotte et al., 2022[3] | USA |

AUC, area under the curve; FIX, factor IX; GTMP, no SAEs related to gene therapy product; LHON, Leber hereditary optic neuropathy; N/A. not appliable.

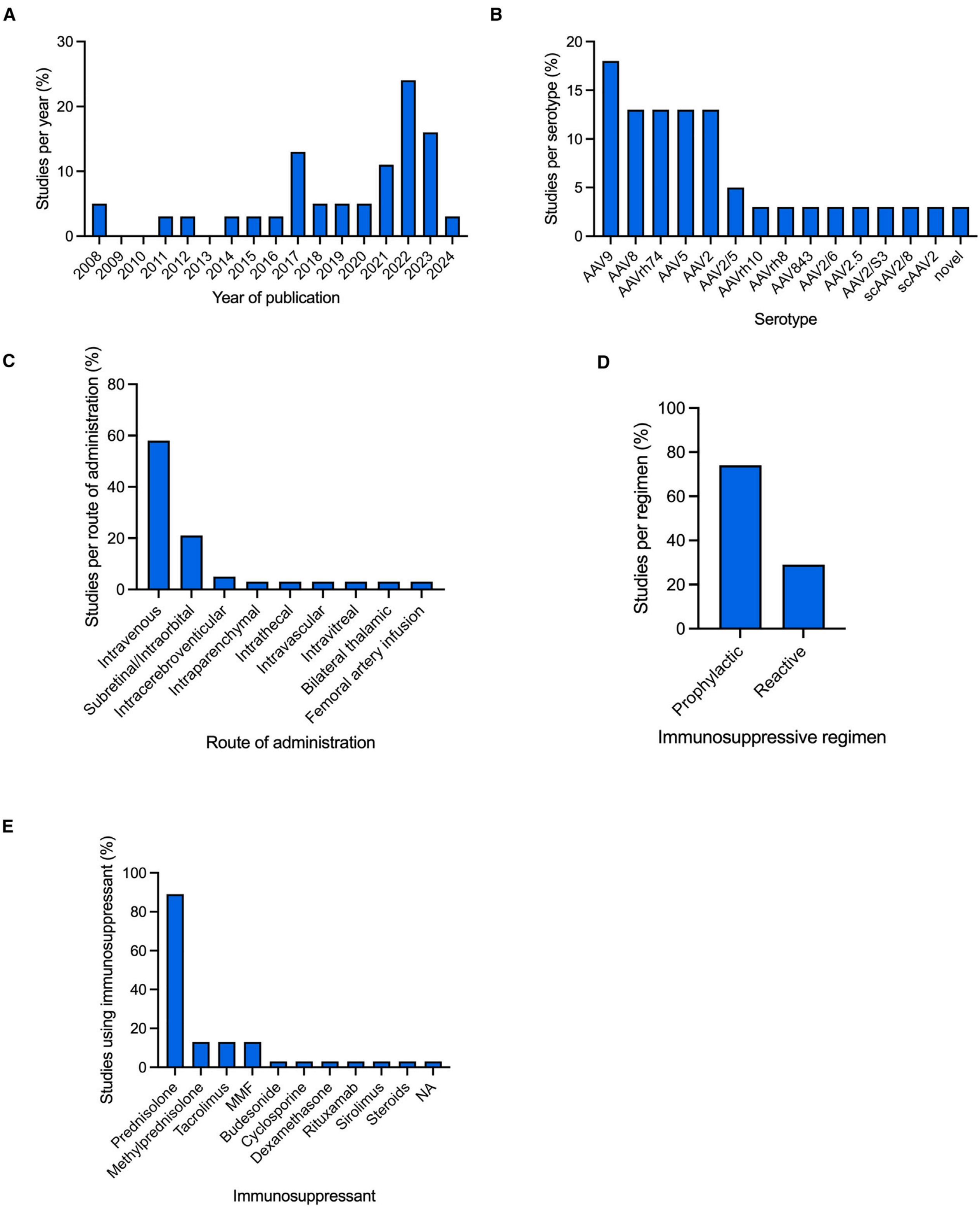


**Figure 1. Number of studies identified during the systematic review**

(A–E) Year of study, (B) serotype, (C) route of administration, (D) immunosuppressive regimen, and (E) immunosuppressant used.

**Table 2. Overview of AAV gene therapy in real-world studies**

| No. | Disease | AAV gene therapy | Vector type | Dose | Route | No. of participants at time of treatment | Age at injection | No. of patients dosed | No. of patients receiving immunosuppressants | AEs associated with AAV gene therapy | Immunosuppressive protocol | Clinical evidence of immunosuppression effectiveness | Clinical evidence of AAV gene therapy effectiveness | Reference |
|---|---|---|---|---|---|---|---|---|---|---|---|---|---|---|
| 1 | | | | | | *n* = 99 | median age: 10 months | *n* = 99 | 99 | asymptomatic thrombocytopenia<br>elevated troponin I levels<br>elevated liver enzyme levels<br>more pronounced transaminitis was observed in 70 patients | prednisolone (1 mg/kg/day) for 30 days; prednisolone (2 mg/kg/day) if transaminase levels exceeded 2-fold the ULNs<br>IV methylprednisolone in 5 patients with worsening acute transmanisits<br>*N* = 1 received mycophenolate for chronic transaminitis | reduced liver enzyme levels | after OA infusion, mean ± SD change in CHOP-INTEND score was 11.0 ± 10.3 with increased score in 66/78 patients (84.6%)<br>patients aged <6 months had a 13.9 points higher gain in CHOP-INTEND score than patients ≥2 years, indicative of improved motor function | Gowda et al., 2024[111] |
| 2 | | | | | | *n* = 168 | median age: 3 months | *n* = 168 | N/A | hepatotoxicity (*n* = 49/167; 29.3%), transient thrombocytopenia (*n* = 23/167; 13.8%), cardiac AEs (*n* = 22/167; 13.2%), and TMA (*n* = 1; 0.6%) | prednisolone (1 mg/kg/day) | reduced liver enzyme levels | event-free survival dramatically improved in patients; improved CHOP-INTEND scores; achieved motor milestones | Servais et al., 2024[112] |
| 3 | SMA | onasmnogene abeparvovec | AAV9 | $1.1 \times 10^{14}$ vg/kg | intravenous | *n* = 76 | median age: 16.8 months | *n* = 76 | *n* = 76 | SAEs were in 8 (11%) children, mostly subacute hepatopathy (*n* = 7 [9%])<br>pyrexia (*n* = 47, 62%)<br>vomiting or loss of appetite (*n* = 41, 54%)<br>minor upper airway infection (*n* = 6, 8%)<br>petechiae (*n* = 1, 1%)<br>liver enzyme elevation (*n* = 56, 74%)<br>thrombocytopenia (*n* = 59, 78%)<br>cardiac AEs (*n* = 2) | prednisolone (1 mg/kg/day) for ≥30 days | liver enzyme levels normalized | significant improvements in CHOP-INTEND and HMFSE scores in 49 patients<br>achievement of motor milestones | Weiβ et al., 2022[83] |
| 4 | | | | | | *n* = 9 | 1.7–48 months | *n* = 9 | *n* = 9 | fever (*n* = 6, 66.7%)<br>pyrexia (*n* = 47, 62%)<br>Vomiting (*n* = 6, 66.7%)<br>diarrhea (*n* = 3, 33.3%)<br>thrombocytopenia (*n* = 5, 55.6%)<br>hypertransaminasemia (*n* = 7, 77.8%)<br>liver echogenicity changes (*n* = 1, 11.1%)<br>increased troponin I (*n* = 9, 100%) | prednisolone (1 mg/kg/day) | liver enzyme levels normalized | all patients retained autonomous respiratory capacity without the need for tracheostomy or permanent ventilation and were autonomous in feeding<br>CHOP-INTEND scores increased in patients over time | Bitetti et al., 2013[113] |
| 5 | | | | | | *n* = 1 | 13 months | *n* = 1 | *n* = 1 | unreported | prednisolone (1 mg/kg/day) | liver enzyme levels normalized | improved motor and respiratory function, decreased saliva aspirations | Nanri et al., 2024[114] |
| 6 | | | | | | *n* = 1 | 4 months | *n* = 1 | *n* = 1 | fatal TMA | oral steroid (1 mg/kg/day) | unreported | not reported due to patient fatality | Guillou et al., 2022[25] |
| 7 | | | | | | *n* = 13 | 25.4–48.0 months | *n* = 13 | unreported | no SAEs were related to gene therapy | unreported | unreported | no patients have required increased | Mendell et al., 2021[14] |

(*Continued on next page*)

**Table 2. *Continued***

| No. | Disease | AAV gene therapy | Vector type | Dose | Route | No. of participants at time of treatment | Age at injection | No. of patients dosed | No. of patients receiving immunosuppressants | AEs associated with AAV gene therapy | Immunosuppressive protocol | Clinical evidence of immunosuppression effectiveness | Clinical evidence of AAV gene therapy effectiveness | Reference |
|---|---|---|---|---|---|---|---|---|---|---|---|---|---|---|
| | | | | | | | | | | | | | respiratory support, 2 patients can now stand with assistance | |
| 8 | | | | | | $n$ = 1 | 4.5 years | $n$ = 1 | $n$ = 1 | fever, nausea, vomiting, elevated liver enzyme levels, hepatotoxicity, rise in NT-proBNP (heart failure), jaundice, TMA | prednisolone (1 mg/kg/day) | the elevated liver and heart enzymes more than halved after 1 week with increased dose of prednisolone on day 8 after gene therapy, the patient developed TMA and this resolved within 1 week of eculizumab therapy | Unreported | Witte et al., 2022[115] |
| 9 | | | | | | $n$ = 1 | 7 weeks | $n$ = 1 | $n$ = 1 | thrombocytopenia, feeding intolerance, mild hepatic dysfunction | prednisolone (1 mg/kg/day) | transaminases were not markedly elevated, safe administration in a patient with initial AAV9 antibody titers >1:50 | walks independently, climbs stairs, has no scoliosis and does not need respiratory support | Eisenkölbl and Pühringer, 2024[116] |
| 10 | | | | | | $n$ = 8 | 7–445 days | $n$ = 8 | $n$ = 8 | cardiac AEs ($n$ = 2) | prednisolone (1 mg/kg/day) | reduced liver enzyme levels | improvement in CHOP-INTEND scores at 6-month review | Favia et al., 2024[117] |
| 11 | | | | | | $n$ = 46 | 7–49.5 months | $n$ = 46 | $n$ = 46 | asymptomatic elevated liver enzyme levels | prednisolone (1 mg/kg/day) | only $n$ = 5 had raised liver enzymes and these were asymptomatic commented that earlier treatment (i.e., before significant motor neuron loss) led to a much smaller proportion of patients needing >60 days of steroid treatment | improvement in motor milestones with no significant functional decline some patients also had improved oral feeding and reduced need for respiratory support | Waldrop et al., 2024[118] |
| 12 | | | | | | $n$ = 21 | 1–23 months | $n$ = 21 | $n$ = 21 | modest elevated liver enzyme levels | prednisolone (1 mg/kg/day) | reduced liver enzyme levels | $n$ = 2 experienced stabilization, $n$ = 17 experienced improvement in motor function | Waldrop et al., 2020[119] |
| 13 | | | | | | $n$ = 11 | 3.6 weeks | $n$ = 11 | $n$ = 11 | unreported | prednisolone (1 mg/kg/day) | N/A | reduced sleep disordered breathing | Chiang et al., 2023[120] |
| 14 | | | | | | $n$ = 33 | unreported | $n$ = 33 | unreported | unreported | unreported | unreported | increase in maximal mouth opening | Beri et al., 2023[121] |
| 15 | | | | | | $n$ = 25 | 4–43 months | $n$ = 25 | $n$ = 25 | elevation of liver enzymes and thrombocytopenia; low-grade fever, 1/25 vomiting, 1/25 borderline high blood pressure | prednisolone (1 mg/kg/day) with increase to 2 mg/kg/day | reduced liver enzyme levels; improved platelet levels | improved motor functionals scores; significant improvements in CHOP-INTEND scores | Chencheri et al., 2023[122] |

(*Continued on next page*)

**Table 2. Continued**

| No. | Disease | AAV gene therapy | Vector type | Dose | Route | No. of participants at time of treatment | Age at injection | No. of patients dosed | No. of patients receiving immunosuppressants | AEs associated with AAV gene therapy | Immunosuppressive protocol | Clinical evidence of immunosuppression effectiveness | Clinical evidence of AAV gene therapy effectiveness | Reference |
|---|---|---|---|---|---|---|---|---|---|---|---|---|---|---|
| 16 | | | | | | $n$ = 16 | 1.5–3.4 years | $n$ = 16 | unreported | unreported | unreported | unreported | 10/16 had significant kyphosis, 9/16 has scoliosis | Soini et al., 2023[123] |
| 17 | | | | | | $n$ = 46 (another 21 patients with ≥6 month follow-up after OA infusion.) | 22 days–58 months (1/67 child age of 72 months) | $n$ = 46 (for 12 months) | $n$ = 67 | thrombocytopenia, 15 (22.4%) patients had pyrexia, 14 (20.9%) vomiting or loss of appetite, elevated liver enzyme levels | prednisolone (1 mg/kg/day); adjusted to 2 mg/kg per day when increased AST and ALT levels (more than twice normal values) were detected | reduced liver enzyme levels | overall significant CHOP-INTEND improvement between T0 and T12 (sitting)<br>36/46 children with 1-year follow-up (78%) did not need for nutritional support at baseline after 12 months from the OA, they all remained orally fed the remaining 10 (22%) remained tube feeding<br>10 patients (22%) did not need non-invasive ventilation at baseline; 9 of them (90%) remained on spontaneous breathing after 12 months, only 1 needed for non-invasive ventilation | Pane et al., 2023[124] |
| 18 | | | | | | $n$ = 2 | 18 and 21 days | $n$ = 2 | $n$ = 2 | necrotizing enterocolitis, hematochezia, elevated liver enzymes, vomiting, thrombocytosis in one patient, blood in stool | prednisolone (1 mg/kg/day) with increase to 2 mg/kg/day | reduced liver enzyme levels | unreported | Gaillard et al., 2023[125] |
| 19 | | | | | | $n$ = 25 | 11 days and 23 months | $n$ = 25 | $n$ = 25 | fever, respiratory distress, upper respiratory viral illness, thrombocytopenia, elevated liver enzyme levels | prednisolone (1 mg/kg/day) | reduced liver enzyme levels | no regression in motor abilities, gradual improvement in motor function and no recurrent infections and illnesses following OA<br>better progress in motor function observed in patients who received OA earlier and who were presymptomatic | Tokatly Latzer et al., 2023[126] |
| 20 | | | | | | $n$ = 2 | 121 and 42 days | $n$ = 2 | unreported | unreported | unreported | unreported | patient 1 became bedridden despite receiving OA (extremities movement improved, predominantly in the upper extremities, and the paradoxical respiration and | Sawada et al., 2022[127] |

(*Continued on next page*)

**Table 2. Continued**

| No. | Disease | AAV gene therapy | Vector type | Dose | Route | No. of participants at time of treatment | Age at injection | No. of patients dosed | No. of patients receiving immunosuppressants | AEs associated with AAV gene therapy | Immunosuppressive protocol | Clinical evidence of immunosuppression effectiveness | Clinical evidence of AAV gene therapy effectiveness | Reference |
|---|---|---|---|---|---|---|---|---|---|---|---|---|---|---|
| | | | | | | | | | | | | | tongue fasciculation disappeared the CHOP INTEND score decreased to 15 before treatment but improved to 34 after treatment he did not need respiratory support and could consume food orally at the age of 2 years and 1 month but had achieved no motor development milestones other than rolling over he is undergoing physical and occupational therapy). Patient 2 achieved normal motor development (head control, rolling over, sitting without support, standing with support, independent walking, beginning to run | Stettner et al., 2023[128] |
| 21 | | | | | | *n* = 9 | 19–527 days | *n* = 9 | *n* = 9 | thrombocytopenia, elevated liver enzyme levels | prednisolone (1 mg/kg/day) | reduced liver enzyme levels | improvements of motor function. 3/6 SMA type 1 patients required nutritional support, 4/6 developed scoliosis, 1/6 sleep disturbed breathing SMA type 2 patient and 2 treated at pre-symptomatic stage did not require nutritional or respiratory support and did not develop a scoliosis | |
| 22 | | | | | | *n* = 1 | 5 months | *n* = 1 | *n* = 1 | fever and loss of appetite, elevated liver enzyme levels, thrombocytopenia, hyper-ferritinemia | prednisolone (1 mg/kg/day) with increase to 2 mg/kg/day | reduced liver enzyme levels, normalized platelet count | improvement in motor function; acquired a stable sitting position, maintained kneeling position with anterior support, stand unaided with upper limbs support, no respiratory problems. Normal neurocognitive and speech profile, fed by mouth and no swallowing problems | Tosi et al., 2022[129] |

(*Continued on next page*)

**Table 2. Continued**

| No. | Disease | AAV gene therapy | Vector type | Dose | Route | No. of participants at time of treatment | Age at injection | No. of patients dosed | No. of patients receiving immunosuppressants | AEs associated with AAV gene therapy | Immunosuppressive protocol | Clinical evidence of immunosuppression effectiveness | Clinical evidence of AAV gene therapy effectiveness | Reference |
|---|---|---|---|---|---|---|---|---|---|---|---|---|---|---|
| 23 | | | | | | $n$ = 6 (5 type 1 and 1 type 2) | 7–24 months | $n$ = 6 | $n$ = 6 | elevated liver enzyme levels | prednisolone (1 mg/kg/day) | reduced GGT, ALT and AST levels | motor function improvements, no requirements for permanent ventilatory support and no case of mortality | Lee et al., 2022[130] |
| 24 | | | | | | $n$ = 7 | 7.5–19.2 months | $n$ = 7 | $n$ = 7 | fever and/or emesis, thrombocytopenia, elevated liver enzyme levels | prednisolone (1 mg/kg/day) | reduced ALT and AST levels, normal platelet count | motor function improvements | Matesanz et al., 2021[131] |
| 25 | | | | | | $n$ = 9 | ≤2 years | $n$ = 9 | $n$ = 9 | elevated liver enzyme levels, vomiting, reduced appetite, high prothrombin time, elevated bilirubin | prednisolone (1 mg/kg/day) | reduced liver enzyme levels | significant improvements in CHOP INTEND scores | Ali et al., 2021[132] |
| 26 | | | | | | $n$ = 10 | 19 months | $n$ = 10 | $n$ = 10 | hyperthermia, vomiting, lethargy and/or loose stool, thrombocytopenia, elevated liver enzyme levels | prednisolone (1 mg/kg/day) with increase to 2 mg/kg/day | reduced liver enzyme levels, reduced platelet count | relatively safe and effective with improvements of motor skills | Nevmerzhitskaya et al., 2021[133] |
| 27 | | | | | | $n$ = 21 | 0.65–24 months | $n$ = 21 | $n$ = 21 | vomiting, transaminitis and thrombocytopenia | prednisolone (1 mg/kg/day) | reduced liver enzyme levels, reduced platelet count | stabilization or improvement in bulbar or respiratory function | D'Silva et al., 2022[109] |
| 28 | | | | | | $n$ = 8 | 10–37 months | $n$ = 8 | $n$ = 8 | temporary increase in body temperature (> 38.5°C), vomiting, elevated liver enzyme levels, asymptomatic thrombocytopenia, 2/8 patients experienced an increase of pre-existing tremor | prednisolone (1 mg/kg/day) 7/8 patients; methylprednisolone (20 mg/kg) 1/8 patients | reduced liver enzyme levels, reduced platelet count, thrombocytes normalized | sitting without support, no respiratory support and tube feeding | Friese et al., 2021[65] |
| 29 | | | | | | Group 1 $n$ = 7; Group 2 $n$ = 6 | 2–6 months | $n$ = 7 | $n$ = 7 | fever, vomiting, lack of appetite, mild thrombocytopenia, elevated liver enzyme levels | prednisolone (1 mg/kg/day) | reduced liver enzyme levels | OV after nusinersen did not provide supplementary benefits for motor function or respiratory status early OV treatment results in better outcomes | Mirea et al., 2021[54] |
| 30 | | | | | | $n$ = 1 | 2 months | $n$ = 1 | $n$ = 1 | elevated liver enzyme levels | prednisolone (1 mg/kg/day) | reduced liver enzyme levels | head control at 4 months of age, independent walking at 18 months, pulling to stand, walk, and sit independently, and is reaching for objects; eating orally, gaining weight, no respiratory concerns, can say up to 10 words | Nigro et al., 2023[134] |

(*Continued on next page*)

**Table 2.** ***Continued***

| No. | Disease | AAV gene therapy | Vector type | Dose | Route | No. of participants at time of treatment | Age at injection | No. of patients dosed | No. of patients receiving immunosuppressants | AEs associated with AAV gene therapy | Immunosuppressive protocol | Clinical evidence of immunosuppression effectiveness | Clinical evidence of AAV gene therapy effectiveness | Reference |
|---|---|---|---|---|---|---|---|---|---|---|---|---|---|---|
| 31 | SMA | onasmnogene abeparvovec (with nusinersen and risdiplam) | AAV9 | $1.1 \times 10^{14}$ vg/kg | intravenous | $n = 1$ | 4 months | $n = 1$ | $n = 1$ | moderate/severe TMA, hypertransaminasemia | prednisolone (1 mg/kg/day) | liver enzyme levels normalized | nusinersen: improvement in motor and bulbar function; onasmnogene abeparvovec: Sitting with support at 2 years old and acquired independent sitting at 27 months. Patient began to eat semisolids orally; risdiplam: further improvements in both motor and bulbar functions one year after risdiplam therapy | Bitetti et al., 2023[113] |
| 32 | Inherited Retinal Dystrophy (RPE-65 mediated) | VN | AAV2 | $1.5 \times 10^{11}$ vg per eye | Subretinal | $n = 3$ | 22 months, 2 years, 5 years | $n = 3$ | $n = 3$ | $n = 3$ acute subretinal deposits | prednisolone (1 mg/kg/day) | N/A | improved visual function with macular and inferior subretinal deposits improved or resolved | Lopez et al., 2023[135] |
| 33 | Inherited Retinal Dystrophy (RPE-65 mediated) | VN | AAV2 | $1. \times 10^{11}$ vg per eye | Subretinal | $n = 6$ | 18–49 years | $n = 6$ | $n$-6 | retinal atrophy in 10/12 eyes (8 mild/2 severe)<br>increased ocular pressure (3 patients: 6 eyes)<br>increased intraocular inflammation (2 eyes)<br>cataracts (4 eyes)<br>glaucoma surgery (2 patients: 4 eyes)<br>higher occurrence of retinal atrophy and increased IOP than previously reported | prednisolone (1 mg/kg/day) | N/A | best-corrected visual acuity remained stable (baseline: 1.28 (±0.71) vs. last follow-up: 1.46 (±0.60); $p = 0.25$). average white full-field stimulus testing showed a trend toward improvement (baseline: −4.41 (±10.62) dB vs. last follow-up: −11.98 (±13.83) dB; $p = 0.18$). | Kiraly et al., 2023[55] |
| 34 | Inherited Retinal Dystrophy (RPE-65 mediated) | VN | AAV2 | $1.5 \times 10^{11}$ vg per eye | Subretinal | $n = 1$ | 39 years | $n = 1$ | $n = 1$ | foveal ellipsoid zone loss | prednisolone (1 mg/kg/day) | N/A | the first eye showed improvement in rod photoreceptor function with increased peripheral and low luminance vision (baseline VA: 0.9 logMAR and 2-years post-operative VA: 0.7 logMAR. 2ND eye developed loss of foveal photoreceptors. FST improvements were maintained in both eyes. macular edema resolved by 6 weeks of VN | Jalil et al., 2023[136] |

(*Continued on next page*)

**Table 2. Continued**

| No. | Disease | AAV gene therapy | Vector type | Dose | Route | No. of participants at time of treatment | Age at injection | No. of patients dosed | No. of patients receiving immunosuppressants | AEs associated with AAV gene therapy | Immunosuppressive protocol | Clinical evidence of immunosuppression effectiveness | Clinical evidence of AAV gene therapy effectiveness | Reference |
|---|---|---|---|---|---|---|---|---|---|---|---|---|---|---|
| 35 | hemophilia B | Etranacogene dezaparvovec | AAV5 | $2 \times 10^{13}$ vg/kg | intravenous | $n = 3$ | 43, 47 and 50 years | $n = 3$ | $n = 3$ | headache<br>transient elevation of C-reactive protein | N/A | N/A | stable durable FIX activity remained after 3 years<br>discontinued FIX prophylaxis in all patients<br>clinical phenotype from severe/ moderately severe to mild/non-hemophilic<br>100% decrease in bleeds in 2/3 and 92% in patient 3 | Von Drygalski et al., 2019[106] |

FIX, factor IX; GGT, gamma-glutamyl transferase; NT-proBNP; ULN, upper limit of normal.

safety profile of OA. Common AEs included pyrexia, vomiting, thrombocytopenia, and elevated liver enzymes. AEs related to OA were frequent and sometimes serious. Although most AEs were satisfactorily managed in clinical trials, one death was reported in an observational study by Mirea et al. in 2021.[54]

Elevations in liver enzymes were successfully treated with prednisolone, which can also increase the response to treatment by suppressing the antigen-specific T cell response that clear transduced cells and thus result in a loss of transgene expression.[11] In the treatment of inherited retinal dystrophy, all patients reported subjective vision improvement after VN gene therapy. The overall safety and effectiveness of VN treatment align with previous VN clinical trials, excluding the higher occurrence of retinal atrophy and increased ocular pressure observed.[55]

### Treatment-associated AEs

AEs were observed in 30 of 38 clinical trials involving 19 AAV gene therapies (Table 3). Increased levels of liver enzymes or liver toxicity were the most frequently reported AE, which was recorded in 21 clinical trials (70%). For the purpose of this review, elevated liver enzymes or liver toxicity included any reference to elevated gamma-glutamyl transferase (GGT), ALT, AST, liver enzymes, liver toxicity, or hepatoxicity. Other frequent AEs included vomiting and nausea ($n$ = 11 [37%]), pyrexia ($n$ = 10 [33%]), and fatigue ($n$ = 6 [20%]). Although AEs were recorded in 30 of the 38 clinical trials, SAEs were only observed in 15 of the included studies (Table 3), with the highest frequency of SAEs observed in clinical trials for SMA ($n$ = 4), hemophilia A ($n$ = 3), hemophilia B ($n$ = 3), and DMD ($n$ = 2). Four deaths were reported in patients in both low- and high-dose groups because an SAE associated with the treatment protocol in a trial for X-linked myotubular myopathy with resamirigene bilparvovec, an AAV8 clinical vector (NCT03199469).[56] Of note, these patients had presented with cholestasis before vector dosing. Due to the clinical vector serotypes used for these diseases, the most common serotypes resulting in SAEs were AAV9 ($n$ = 4, for SMA), AAV5 ($n$ = 2 for hemophilia A and $n$ = 1 for hemophilia B), and rhAAV74 ($n$ = 2, for DMD) after systemic delivery. As detailed in Table 1, SAEs were associated with the systemic delivery of AAVs with the exception of X-linked retinitis pigmentosa. Elevations in liver enzyme levels were reported as SAEs after clinical vector administration in clinical trials for DMD ($n$ = 2), hemophilia A ($n$ = 2), hemophilia B ($n$ = 3), mucopolysaccharidosis (MPS) type IIIB ($n$ = 1), and SMA ($n$ = 4). Although most studies described management of elevated liver enzyme SAEs via treatment with corticosteroids (even when corticosteroids were administered prophylactically to patients), two studies (NCT03769116 [57] and EudraCT 2012-000856-33[58] did not describe any treatment for AEs observed in patients and one reported that there was no clear association between the resolution of elevated ALT levels and prednisolone use.[59] The immunosuppressive regimen performed in the trials was prophylactic and AEs recorded in patients were resolved without intervention. All other SAEs relating to clinical vector administration in the studies are detailed in Table 3. In contrast with clinical trials for the treatment of DMD, hemophilia A and B, MPS type IIIB, and

**Table 3. Treatment-associated AE overview**

| No. | Disease | AAV gene therapy | Serotype | No. of patients dosed | Treatment-related SAEs | Treatment-related AEs | Frequency of treatment-related AEs | Management of AEs | NCT (phase) | Reference |
|---|---|---|---|---|---|---|---|---|---|---|
| 1 | | Delandistrogene moxeparvovec | rAAVrh74 | 2 | None reported | bruising<br>decreased lymphocyte count<br>bleeding at femoral catheterization site<br>vomiting<br>NB: authors only described events as treatment-emergent and were not specified as treatment-related | 100% ($n$ = 2) | no treatment needed for AEs. prednisone administration to patients was prophylactic. | NCT03333590 (1/2) | Flanigan et al., 2022[64] |
| 2 | | Delandistrogene moxeparvovec | rAAVrh74 | 4 | None reported | vomiting ($n$ = 9)<br>nausea ($n$ = 1)<br>fatigue ($n$ = 1)<br>asthenia ($n$ = 1)<br>decreased appetite ($n$ = 2)<br>elevated liver enzyme levels ($n$ = 4) | 100% ($n$ = 4) | elevated γ-glutamyl transferase resolved with corticosteroids | NCT03375164 (1 and 2) | Mendell et al., 2020[66] |
| 3 | DMD | Delandistrogene moxeparvovec | rAAVrh74 | 20 | rhabdomyolysis<br>elevated liver enzyme levels<br>liver injury | vomiting<br>decreased appetite<br>nausea<br>elevated liver enzyme levels<br>abdominal pain<br>increased blood bilirubin<br>pain in extremity<br>rhabdomyolysis<br>pyrexia | AE: 100% ($n$ = 21)<br>SAE: 5% ($n$ = 1) | no treatment described for AEs<br>prednisone administration to patients was prophylactic. | NCT03769116 (2) | Mendell et al., 2023[57] |
| 4 | | Delandistrogene moxeparvovec | rAAVrh74 | 20 | elevated liver enzyme levels<br>vomiting | vomiting<br>decreased appetite<br>increased glutamate dehydrogenase<br>nausea<br>constipation<br>fatigue<br>elevated liver enzyme levels<br>increased blood creatine phosphokinase<br>thrombocytopenia<br>abdominal pain upper<br>increased blood lactate dehydrogenase<br>headache<br>hemoglobinuria<br>pyrexia<br>diarrhea | AE: 90% ($n$ = 18)<br>SAE: 10% ($n$ = 2) | patients received prophylactic prednisone or prednisolone (1 mg/kg) in addition to baseline corticosteroid dose, for a total ≤ 60 mg/day, which was continued for ≥60 days post-treatment and subsequently tapered, depending on serum γ-glutamyl transferase levels | NCT04626674 (1b) | Zaidman et al., 2023[103] |

(*Continued on next page*)

**Table 3. *Continued***

| No. | Disease | AAV gene therapy | Serotype | No. of patients dosed | Treatment-related SAEs | Treatment-related AEs | Frequency of treatment-related AEs | Management of AEs | NCT (phase) | Reference |
|---|---|---|---|---|---|---|---|---|---|---|
| 5 | hemophilia A | Valoctocogene roxaparvovec | AAV5 | 134 | elevated liver enzyme levels<br>headache<br>arthralgia<br>nausea | elevated liver enzyme levels<br>headache<br>arthralgia<br>nausea | AE: 92% ($n$ = 123)<br>SAE: 4% ($n$ = 5) | immunosuppressants were given in relation to an alanine transferase rise<br>median duration of elevation in ALT was 21 days<br>there was no apparent relationship between the development of anti-AAV5 antibodies and factor VIII activity | NCT03370913 (3) | Zaidman et al., 2023[103] |
| 6 | hemophilia A | Valoctocogene roxaparvovec | AAV5 | 9 | elevated liver enzyme levels | elevated liver enzyme levels | AE by year:<br>Y1: 86% ($n$ = 6)<br>Y2: 14% ($n$ = 1)<br>Y3: 14% ($n$ = 1)<br>Y4: 29% ($n$ = 2)<br><br>SAE by year:<br>Y1: 17% ($n$ = 1) | corticosteroids were used prophylactically or in response to elevated ALT levels (1.5× above baseline)<br>there was no clear association between the resolution of the elevated ALT level and prednisolone use | NCT02576795 (1/2) | Rangarajan et al., 2017, Long et al., 2021, Fong et al., 2022, Pasi et al., 2021[59;67;81;85] |
| 7 | hemophilia A | Giroctocogene fitelparvovec | AAV6 | 11 | Pyrexia<br>Hypotension | elevated liver enzyme levels<br>tachycardia<br>fatigue<br>myalgia | SAE: 27% ($n$ = 3)<br>AE:100% ($n$ = 11) | liver enzyme levels were managed with tapering corticosteroid administration<br>hypotension and pyrexia resolved with treatment with electrolytes, norepinephrine, ondansetron, glucose, and paracetamol | NCT03061201 (1/2) | Leavitt et al., 2024[104] |
| 8 | hemophilia B | BBM-H901 | dsAAV843 | 10 | None reported | pyrexia<br>elevated liver enzyme levels | pyrexia (10%, $n$ = 1)<br>aminotransferase elevations (10%, $n$ = 1) | glucocorticoid administration | NCT04135300 (1) | Xue et al., 2022[63] |
| 9 | hemophilia B | Etranacogene dezaparvovec | AAV5 | 3 | None reported | headache<br>mild elevation in C-reactive protein levels | 33% ($n$ = 1) | AEs resolved without intervention | NCT03489291 (2b) | Von Drygalski et al., 2019[106] |
| 10 | hemophilia B | Etranacogene dezaparvovec | AAV6 | 54 | None reported | Arthralgia<br>headache<br>fatigue<br>elevated liver enzyme levels<br>blood creatine kinase increase<br>back pain<br>influenza-like illness<br>diarrhea<br>nausea | 69% ($n$ = 37) | glucocorticoid administration for liver enzyme elevations | NCT03569891 (3) | Pipe et al., 2023[16] |
| 11 | hemophilia B | Verbrinacogene setparvovec | AAV2/S3 | 10 | elevated liver enzyme levels<br>decreased coagulation FIX | elevated liver enzyme levels<br>fatigue<br>increased coagulation | AEs: 80% ($n$ = 8)<br>SAE: 70% ($n$ = 7)<br>NB: AEs were not observed in patients | increase in liver enzyme levels were managed with intravenous | NCT03369444 and NCT03641703 (1/2) | Chowdary et al., 2022[48] |

(*Continued on next page*)

**Table 3. *Continued***

| No. | Disease | AAV gene therapy | Serotype | No. of patients dosed | Treatment-related SAEs | Treatment-related AEs | Frequency of treatment-related AEs | Management of AEs | NCT (phase) | Reference |
|---|---|---|---|---|---|---|---|---|---|---|
| | | | | | pulmonary sepsis<br>arteriovenous fistula thrombosis | FIX<br>muscle spasms/ musculoskeletal pain/ myalgia<br>dyspepsia/eructation<br>AV fistula thrombosis<br>decreased coagulation FIX<br>headache<br>pulmonary sepsis<br>somnolence | treated with low dose vector ($3.84 \times 10^{11}$ vg/kg, $n = 2$). | methylprednisolone and tacrolimus | | |
| 12 | | AMT-060 | AAV5 | 10 | elevated liver enzyme levels<br>Pyrexia | elevated liver enzyme levels<br>pyrexia<br>anxiety<br>drug ineffective<br>palpitations<br>headache<br>prostatitis<br>rash | AEs: 60% ($n = 6$)<br>SAEs: 30% ($n = 3$) | tapering course of prednisolone | NCT02396342 (1/2) | Majowicz et al., 2019, Miesbach et al., 2018[68;86] |
| 13 | | SPK-9001 | A novel, bioengineered single-stranded adeno-associated viral vector carrying human FIX variant | 10 | none reported | elevated liver enzyme levels | 10% ($n = 1$) | 2 patients required 60 mg prednisone in the context of ALT rises/ immune responses<br>this was tapered down over 119 and 130 days respectively | NCT02484092 (1/2) | George et al., 2017[105] |
| 14 | | BAX 335 (scAAV8.FIXR338) | AAV8 | 7 | None reported | fatigue<br>feeling flushed<br>headache<br>infleunza-like symptoms<br>ankle swelling<br>elevated liver enzyme levels<br>high blood pressure<br>abscess | 57% ($n = 4$) | prednisone administration upon detection of high liver enzyme levels | NCT01687608 (1/2) | Konkle et al., 2021[82] |
| 15 | | scAAV2/8-LP1-hFIXco | rAAVrh74 | 10 | elevated liver enzyme levels | lethargy<br>elevated liver enzyme levels<br>anemia | 100% ($n = 10$) | prednisone, 60 mg/ patient with subsequent tapering of the dose<br>patient 5: 9 weeks<br>patient 6: 4 weeks | NCT00979238 (1/2) | Nathwani et al., 2011, Nathwani et al., 2014[43;44] |
| 16 | LHON | scAAV2-P1ND4v2 | AAV2 | 28 | none reported | Uveitis | 29% ($n = 8$) | Topical prednisolone | NCT02161380 (1) | Lam et al., 2022[107] |

(*Continued on next page*)

**Table 3. *Continued***

| No. | Disease | AAV gene therapy | Serotype | No. of patients dosed | Treatment-related SAEs | Treatment-related AEs | Frequency of treatment-related AEs | Management of AEs | NCT (phase) | Reference |
|---|---|---|---|---|---|---|---|---|---|---|
| 17 | MPS type IIIB | rAAV2/5-hNaGlu | AAV2/5 | 4 | respiratory tract infection<br>elevated liver enzyme levels<br>diarrhea | upper respiratory tract infection<br>minor anesthesia-related diarrhea or gastroenteritis<br>elevated liver enzyme levels<br>behavior<br>anemia<br>transient hydroelectric disorder<br>tonsillectomy<br>adenoidectomy<br>minimum mitral insufficiency<br>transient loss of appetite<br>NB: authors only described events as treatment-emergent and were not specified as treatment-related | 100% ($n$ = 4) | no treatment described for AEs. prednisone administration to patients was prophylactic. | EudraCT, number 2012-000856-33, and the International Standard Clinical Trial Registry, number ISRCTN19853672 (1/2) | Tardieuc et al., 2017[58] |
| 18 | | rAAV2/5-hNaGlu | AAV2/5 | 4 | none reported | upper respiratory tract infection<br>diarrhea or gastroenteritis<br>elevated liver enzyme levels<br>anemia<br>behavior<br>bronchitis<br>cough<br>pyrexia<br>conjunctivitis<br>sleeping disorders<br>Atopic dermatitis<br>NB: Authors only described events as treatment-emergent and were not specified as treatment-related | 100% ($n$ = 4) | no treatment described for AEs<br>prednisone administration to patients was prophylactic. | NCT03300453 (1/2) | Gougeon et al., 2021, Deiva et al., 2021[49;50] |
| 19 | RPE65-Deficient LCA and Severe Early-Childhood Onset Retinal Dystrophy | rAAV2-CB-hRPE65 | AAV2 | 12 | none reported | ocular hyperemia<br>photopsia | 25% ($n$ = 3) | no treatment described for AEs<br>topical corticosteroid administration to patients was prophylactic | NCT00749957 (1/2) | Weleber et al., 2016[108] |
| 20 | RPE65-mediated inherited retinal dystrophy (LCA) | AAV8-coRPGR codon optimized RPGR | AAV8 | 18 | none reported | anterior uveitis<br>subretinal inflammation<br>NB: this was only observed in patients in medium and high dose groups | 33% ($n$ = 6) | no treatment described for AEs<br>prednisolone administration to patients was prophylactic | NCT03116113 (1/2) | Cehajic-Kapetanovic et al., 2020[60] |

(*Continued on next page*)

**Table 3. *Continued***

| No. | Disease | AAV gene therapy | Serotype | No. of patients dosed | Treatment-related SAEs | Treatment-related AEs | Frequency of treatment-related AEs | Management of AEs | NCT (phase) | Reference |
|---|---|---|---|---|---|---|---|---|---|---|
| 21 | | OA | AAV9 | 76 | subacute hepatopathy<br>elevated liver enzyme levels<br>acute liver dysfunction | pyrexia<br>vomiting<br>thrombocytopenia<br>rashes | 74% ($n$ = 56) | escalating prednisolone to 2 mg/kg per day for about 4 weeks from 1 mg/kg | N/A (observational study) | Weiβ et al., 2022[83] |
| 22 | | OA | AAV9 | 15 | none reported | liver toxicity<br>thrombocytopenia<br>increased troponin | 53% ($n$ = 8) | prednisolone, varying doses depending on AE and patient | NCT03505099 (3) | Strauss et al., 2022[24] |
| 23 | | OA | AAV9 | 32 | elevated liver enzyme levels ($n$ = 1) | hypertension<br>elevated liver enzyme levels<br>lymphadenopathy<br>pyrexia<br>vomiting<br>prolonged thromboplastin time<br>increased blood creatine phosphokinase<br>cardiac murmur<br>abnormal hair growth<br>hepatomegaly<br>pericardial effusion<br>sinus tachycardia | 37.5% ($n$ = 12) | increased prednisolone dose administered to patient with elevated liver enzymes<br>other AEs were resolved without intervention | NCT03381729 (1) | Finkel et al., 2023[70] |
| 24 | SMA | OA | AAV9 | 21 | None reported | vomiting<br>elevated liver enzyme levels | 100% ($n$ = 21) | antiemetic medication<br>oral and enteral feeding to maintain hydration<br>systemic corticosteroid administration | N/A (cohort study) | D'Silva et al., 2022[109] |
| 25 | | OA | AAV9 | 15 | elevated liver enzyme levels ($n$ = 2) | elevated liver enzyme levels below SAE cutoff point ($n$ = 2) | 27% ($n$ = 4) | prednisolone treatment | NCT02122952 (1) | Mendell et al., 2017[11] |
| 26 | | OA | AAV9 | 8 | None reported | elevated liver enzymes<br>pyrexia<br>vomiting<br>reduced appetite<br>exacerbation of hand tremor<br>thrombocytopenia<br>Increase in troponin I/T, CRP, and monocyte counts | 100% ($n$ = 8) | prednisolone dose increased above 1 mg/kg | N/A (retrospective analysis) | Friese et al., 2021[65] |
| 27 | | OA | AAV9 | 33 | pyrexia<br>elevated liver enzyme levels<br>gastroenteritis<br>rhinovirus infection<br>virus infection<br>feeding disorder<br>hypernatremia<br>thrombocytopenia<br>abnormal coagulation test | pyrexia<br>upper respiratory infection<br>elevated liver enzyme levels<br>vomiting<br>constipation<br>gastroenteritis<br>rhinovirus infection<br>virus infection<br>respiratory tract infection | AE: 73% ($n$ = 24)<br>SAE: 18% ($n$ = 6) | following the recommendation to increase prophylactic prednisolone dosing for the first 3 days from 1 to 2 mg/kg per day, p (27%) patients received an initial dose of 2 mg/kg per day<br>the duration of prednisolone dosing ranged from 54 to | NCT03306277 (3) | Day et al., 2021, Mercuri et al., 2021[12;13] |

(*Continued on next page*)

**Table 3. *Continued***

| No. | Disease | AAV gene therapy | Serotype | No. of patients dosed | Treatment-related SAEs | Treatment-related AEs | Frequency of treatment-related AEs | Management of AEs | NCT (phase) | Reference |
|---|---|---|---|---|---|---|---|---|---|---|
| | | | | | | cough<br>diarrhea<br>pneumonia<br>gastro-esophageal reflux disease<br>nasopharyngitis<br>hypertension | | 235 days (median 65.0 [IQR 13.0]; mean 80.9 [SD 41.2]) and mean daily dose ranged from 0.5 to 1.6 mg/kg per day<br>2 patients switched to an equivalent dose of hydrocortisone (using a conversion ratio of 1:4) as an alternative to prednisolone on day 165 and day 132 (extended use after the tapering period was prescribed to treat elevated liver enzymes). | | |
| 28 | X-linked myotubular myopathy | Resamirigene bilparvovec | AAV8 | 24 | increased total bilirubin values elevation in liver enzyme levels<br>NB: these resulted in death in 4 participants | pyrexia<br>creatine phosphokinase increase<br>respiratory tract infection | AE: 96% (*n* = 23)<br>SAE: 46% (*n* = 11) | prednisone administration was prophylactic.<br>participants presenting with SAEs resulting in death were provided with high-dose prednisolone and other immune-modulating therapies (e.g., prolonged or increased dose of prednisolone, anakinra, tocilizumab, and ruxolitinib) with no apparent benefit. | NCT03199469 (2/3) | Shieh et al., 2023[56] |
| 29 | X-linked retinitis pigmentosa | Cotoretigene toliparvovec | AAV8 | 18 | ocular inflammation leading to reduced visual acuity | noninfective retinitis<br>corneal deposits | AE: 39% (*n* = 7)<br>SAE: 11% (*n* = 2) | ocular inflammation-associated SAE were treated with corticosteroids in one participant | NCT03116113 (1) | von Krusenstiern et al., 2023[110] |
| 30 | X-linked retinoschisis | AAV8-RS1 | AAV8 | 11 | None reported | ocular inflammation<br>mild vitritis<br>anterior chamber inflammation | 55% (*n* = 6) | subject 9 was pretreated with prednisone and topical steroid at 2 days before dosing and continued beyond day 14<br>subjects 10 and 11 were treated with cyclosporine at 175 mg twice daily beginning 3 weeks before dosing, and MMF at 500 mg twice daily at 3 weeks and 1000 mg twice daily at 2 weeks before dosing plus prednisone at 60 mg 2 days before vector dosing | NCT02317887 (1/2) | Mishra et al., 2021[71] |

FIX, factor IX; LHON, Leber hereditary optic neuropathy.

SMA, a clinical trial investigating X-linked retinitis pigmentosa (NCT03116113) did not observe any type of SAEs associated with liver enzyme levels after subretinal injection of cotoretigene toliparvovec (BIIB112/AAV8-RPGR).[60] Instead, the SAEs recorded in the study were decreased visual acuity, noninfective retinitis, retinal detachment, and visual impairment. These inflammatory events were further managed with oral prednisolone.

The risk of adverse immune responses from gene therapy is generally related to the type of vector that is used as well as, dose, delivery route,[61] and transgene sequence, which has been found to occur in clinical trials for DMD gene therapy.[62]We have summarized the immune responses associated with several vectors (AAV1-2, 2/3, 2.5, 2/8, 5, 8, 9, rh8, rh.10, rh.74, 843, and γ-RV), routes (intramuscular, subretinal, intravitreal, intracranial, intraparenchymal, intracerebroventricular, low respiratory tract, and cell therapy intravenous) and immunosuppression protocols (Table 4).

#### Immunosuppression-associated AEs

In addition to treatment-related AEs, we also evaluated the frequency of AEs associated with immunosuppressive protocols, including prophylactic and therapeutic immunosuppression, that were used in the clinical trials. Of the 38 clinical trials that were assessed in this review, only 8 studies (21%) reported having any AEs in patients after the administration of immunosuppressive drugs (Table 4). Three of the clinical trials resulting in immunosuppression-associated AEs were studies for hemophilia B gene therapy (however, a different AAV gene therapy was used in each case) and another two were for DMD, while studies for hemophilia A, MPS type IIIB, and SMA presented with immunosuppression-associated AEs in one clinical trial each. The immunosuppressive protocols administered to patients were in response to the following most common AEs observed: increased liver enzyme levels, headache, nausea, and fever. Although the AEs resolved after immunosuppressive treatment, a variety of AEs were reported after immunosuppression. This was more apparent in a clinical trial using valoctocogene roxaparvovec (AAV5-hFVIII-SQ; NCT03370913), where 71% of patients showed AEs to glucocorticoids or other immunosuppressants,[15] and trials using verbrinacogene setparvovec (AAV2/S3) gene therapy (NCT03369444 and NCT03641703),[48] where AEs were recorded in all patients after prednisolone or methylprednisolone treatment and accounted for 24% of all AEs observed.[48] However, with the exception of clinical trial NCT03300453,[49,50] the remaining clinical trials listed in Table 4 reported that AEs after prednisone treatment were only documented in up to two patients.[63] The most common AEs observed in patients after immunosuppressive treatment were insomnia (NCT03370913,[57] NCT04135300,[63] NCT03369444, and NCT03641703[48] and acne (NCT03370913,[57] NCT04135300,[63] and NCT00979238[43,44]). Of note, the authors of NCT03369444 and NCT03641703 stated that the AEs recorded in the patients were consistent with the known safety profiles of glucocorticoids and tacrolimus.[48] In addition, one subject from clinical trials for DMD and SMA, respectively, presented with AEs after prednisone or prednisolone treatment. Clinical trial NCT03333590[64] recorded a patient with a cushingoid face and weight gain, whereas a study by Friese et al.[65] recorded a patient presenting with temporary arterial hypertension.

#### AAV gene therapy and associated immunological responses

The use of AAV gene therapy in subjects with pre-existing immunity or memory response to gene therapy-related viruses could affect the efficacy and the safety of the treatment, constituting one of the major obstacles for gene therapy. Innate and adaptive immunity against the vector capsid or transgenic product may contribute, depending on the magnitude, by varying degrees to immune-mediated rejection and immunotoxicity.[6]

Independent of the AAV serotypes and administration route used during these clinical trials, a significant increase in NAbs was reported. Moreover, the presence of an immunosuppressant regimen during these clinical trials could not prevent the development of anti-AAV antibodies. An evaluation of the cellular immune response showed a significant increase in interferon (IFN)-γ antigen-specific T cells after treatment, and an elevation of inflammatory cytokines (e.g., IFN-γ, TNF-α) in serum was associated with vector reactogenicity.[2,10,13,44,50,61,62,64,66–77] Interestingly, only two studies have reported Treg induction, although all patients developed high titers of binding IgG and NAbs.[63,72]

Currently, the European Medicines Agency[78] and FDA[79] require an evaluation of humoral immunity by determining both the titers and avidities of antibodies against the AAV transgene product during clinical development. For treatments that include redosing, a comprehensive evaluation of the cellular and humoral responses must be performed and documented with concurrent safety and efficacy data (see Immunogenicity).[80] Ultimately, however, few studies have reported an evaluation of the titers as the reciprocal of the highest sample dilution that resulted in inhibition of 50% against the AAV vectors used or transgene products.[10–13,16,44,63,67–69,81–86]

Efforts to suppress the immune response to AAV or transgene product have not been consistent. The most common immunosuppressants, corticosteroids, are not always successful in inhibiting the immune response to AAV or attenuating liver toxicity, and there is no correlation with vector or dosing route used. However, mycophenolate mofetil in combination with tacrolimus has provided a safety profile after a long-term follow-up in all children treated in NCT03300453.[49,50]

## DISCUSSION

The results of this review provide valuable insights into the immunosuppressive protocols and immunological responses associated with various gene therapy treatments. The immune response to AAV gene therapy is complex and can be triggered by vector capsids, genomes, and transgene protein products.[16] Immune responses to AAV are ubiquitous and have been seen across various disease states, routes of administration, and capsid serotypes.[21,72] The route of administration directly influences the vector dose and

**Table 4. Immunosuppression-associated AEs overview**

| No. | Disease | AAV gene therapy | Vector type | Dose | Route | No. of participants at time of treatment | Age at injection | No of patients dosed | No. of patients receiving immunosuppressants | AEs associated with AAV gene therapy | Immunosuppressive protocol | Clinical evidence of immunosuppression effectiveness | Clinical evidence of AAV gene therapy effectiveness | Reference |
|---|---|---|---|---|---|---|---|---|---|---|---|---|---|---|
| 1 | | | | | | $n$ = 99 | median age: 10 months | $n$ = 99 | 99 | asymptomatic thrombocytopenia<br>elevated troponin I levels<br>elevated liver enzyme levels<br>more pronounced transaminitis was observed in 70 patients | prednisolone (1 mg/kg/day) for 30 days; prednisolone (2 mg/kg/day) if transaminase levels exceeded 2-fold the ULNs; IV methylprednisolone in 5 patients with worsening acute transmanisits<br>$n$ = 1 received mycophenolate for chronic transaminitis | reduced liver enzyme levels | after OA infusion, mean ± SD change in CHOP-INTEND score was 11.0 ± 10.3 with increased score in 66/78 patients (84.6%); patients aged <6 months had a 13.9 points higher gain in CHOP-INTEND score than patients ≥2 years, indicative of improved motor function. | Gowda et al., 2024[111] |
| 2 | | | | | | $n$ = 168 | median age: 3 months | $n$ = 168 | N/A | hepatotoxicity ($n$ = 49/167; 29.3%), transient thrombocytopenia ($n$ = 23/167; 13.8%), cardiac AEs ($n$ = 22/167; 13.2%), and TMA ($n$ = 1; 0.6%) | prednisolone (1 mg/kg/day) | reduced liver enzyme levels | event-free survival dramatically improved in patients; improved CHOP-INTEND scores; achieved motor milestones | Servais et al., 2024[112] |
| 3 | SMA | onasmnogene abeparvovec | AAV9 | $1.1 \times 10^{14}$ vg/kg | intravenous | $n$ = 76 | median age: 16.8 months | $n$ = 76 | $n$ = 76 | SAEs were in 8 (11%) children, mostly subacute hepatopathy ($n$ = 7 [9%]).<br>pyrexia ($n$ = 47, 62%)<br>vomiting or loss of appetite ($n$ = 41, 54%)<br>minor upper airway infection ($n$ = 6, 8%)<br>petechiae ($n$ = 1, 1%)<br>liver enzyme elevation ($n$ = 56, 74%)<br>thrombocytopenia ($n$ = 59, 78%)<br>cardiac AEs ($n$ = 2) | prednisolone (1 mg/kg/day) for ≥30 days | liver enzyme levels normalized | significant improvements in CHOP-INTEND and HMFSE scores in 49 patients<br>Achievement of motor milestones | Weiß et al., 2022[83] |
| 4 | | | | | | $n$ = 9 | 1.7–48 months | $n$ = 9 | $n$ = 9 | fever ($n$ = 6, 66.7%)<br>pyrexia ($n$ = 47, 62%)<br>vomiting ($n$ = 6, 66.7%)<br>diarrhea ($n$ = 3, 33.3%)<br>thrombocytopenia ($n$ = 5, 55.6%)<br>hypertransaminasemia ($n$ = 7, 77.8%)<br>liver echogenicity changes ($n$ = 1, 11.1%)<br>Increased troponin I ($n$ = 9, 100%) | prednisolone (1 mg/kg/day) | liver enzyme levels normalized | all patients retained autonomous respiratory capacity without the need for tracheostomy or permanent ventilation and were autonomous in feeding. CHOP-INTEND scores increased in patients over time | Bitetti et al., 2013[113] |
| 5 | | | | | | $n$ = 1 | 13 months | $n$ = 1 | $n$ = 1 | unreported | prednisolone (1 mg/kg/day) | liver enzyme levels normalized | improved motor and respiratory function, decreased saliva aspirations | Nanri et al., 2024[114] |
| 6 | | | | | | $N$ = 1 | 4 months | $n$ = 1 | $n$ = 1 | fatal TMA | Oral steroid (1 mg/kg/day) | unreported | not reported due to patient fatality | Guillou et al., 2022[25] |
| 7 | | | | | | $n$ = 13 | 25.4–48.0 months | $n$ = 13 | unreported | no SAEs were related to gene therapy | unreported | unreported | no patients have required increased respiratory support, 2 | Mendell et al., 2021[14] |

(*Continued on next page*)

**Table 4. *Continued***

| No. | Disease | AAV gene therapy | Vector type | Dose | Route | No. of participants at time of treatment | Age at injection | No of patients dosed | No. of patients receiving immunosuppressants | AEs associated with AAV gene therapy | Immunosuppressive protocol | Clinical evidence of immunosuppression effectiveness | Clinical evidence of AAV gene therapy effectiveness | Reference |
|---|---|---|---|---|---|---|---|---|---|---|---|---|---|---|
| | | | | | | | | | | | | | patients can now stand with assistance | |
| 8 | | | | | | $n = 1$ | 4.5 years | $n = 1$ | $n = 1$ | fever, nausea, vomiting, elevated liver enzyme levels, hepatotoxicity, rise in NT-proBNP (heart failure), jaundice, TMA | prednisolone (1 mg/kg/day) | the elevated liver and heart enzymes more than halved after one week with increased dose of prednisolone on day 8 after gene therapy the patient developed TMA and this resolved within 1 week of eculizumab therapy | unreported | Witte et al., 2022[115] |
| 9 | | | | | | $n = 1$ | 7 weeks | $n = 1$ | $n = 1$ | thrombocytopenia, feeding intolerance, mild hepatic dysfunction | prednisolone (1 mg/kg/day) | transaminases were not markedly elevated, safe administration in a patient with initial AAV9 antibody titers >1:50 | walks independently, climbs stairs, has no scoliosis and does not need respiratory support | Eisenkölbl and Pühringer, 2024[116] |
| 10 | | | | | | $N = 8$ | 7-445 days | $n = 8$ | $n = 8$ | cardiac AEs ($n = 2$) | prednisolone (1 mg/kg/day) | reduced liver enzyme levels | improvement in CHOP-INTEND scores at 6 month review | Favia et al., 2024[117] |
| 11 | | | | | | $n = 46$ | 7–49.5 months | $n = 46$ | $n = 46$ | asymptomatic elevated liver enzyme levels | prednisolone (1 mg/kg/day) | only $n = 5$ had raised liver enzymes and these were asymptomatic commented that earlier treatment (i.e., before significant motor neuron loss) led to a much smaller proportion of patients needing >60 days of steroid treatment | improvement in motor milestones with no significant functional decline. Some patients also had improved oral feeding and reduced need for respiratory support | Waldrop et al., 2024[118] |
| 12 | | | | | | $n = 21$ | 1–23 months | $n = 21$ | $n = 21$ | modest elevated liver enzyme levels | prednisolone (1 mg/kg/day) | reduced liver enzyme levels | $n = 2$ experienced stabilization, $n = 17$ experienced improvement in motor function | Waldrop et al., 2020[119] |
| 13 | | | | | | $n = 11$ | 3.6 weeks | $n = 11$ | $n = 11$ | unreported | prednisolone (1 mg/kg/day) | N/A | reduced sleep disordered breathing | Chiang et al., 2023[120] |
| 14 | | | | | | $n = 33$ | unreported | $n = 33$ | unreported | unreported | unreported | unreported | increase in maximal mouth opening | Beri et al., 2023[121] |
| 15 | | | | | | $n = 25$ | 4–43 months | $n = 25$ | $n = 25$ | elevation of liver enzymes and thrombocytopenia; low-grade fever, 1/25 vomiting, 1/25 borderline high blood pressure | prednisolone (1 mg/kg/day) with increase to 2 mg/kg/day | reduced liver enzyme levels; improved platelet levels | improved motor functionals scores; significant improvements in CHOP-INTEND scores | Chencheri et al., 2023[122] |

(*Continued on next page*)

**Table 4. Continued**

| No. | Disease | AAV gene therapy | Vector type | Dose | Route | No. of participants at time of treatment | Age at injection | No of patients dosed | No. of patients receiving immunosuppressants | AEs associated with AAV gene therapy | Immunosuppressive protocol | Clinical evidence of immunosuppression effectiveness | Clinical evidence of AAV gene therapy effectiveness | Reference |
|---|---|---|---|---|---|---|---|---|---|---|---|---|---|---|
| 16 | | | | | | $n$ = 16 | 1.5–3.4 years | $n$ = 16 | unreported | unreported | unreported | unreported | 10/16 had significant kyphosis, 9/16 has scoliosis | Soini et al., 2023[123] |
| 17 | | | | | | $n$ = 46 (another 21 patients with ≥6 month follow-up after OA infusion.) | 22 days–58 months (1/67 child age of 72 months) | $n$ = 46 (for 12 months) | $n$ = 67 | thrombocytopenia,15 (22.4%) patients had pyrexia,14 (20.9%) vomiting or loss of appetite, elevated liver enzyme levels | prednisolone (1 mg/kg/day); adjusted to 2 mg/kg per day when increased AST and ALT levels (more than twice normal values) were detected | reduced liver enzyme levels | overall significant CHOP-INTEND improvement between T0 and T12 (sitting). 36/46 children with one-year follow-up (78%) did not need for nutritional support at baseline; after 12 months from the OA, they all remained orally fed. The remaining ten (22%) remained tube feeding 10 patients (22%) did not need non-invasive ventilation at baseline; nine of them (90%) remained on spontaneous breathing after 12 months, only one needed for non-invasive ventilation | Pane et al., 2023[124] |
| 18 | | | | | | $n$ = 2 | 18 and 21 days | $n$ = 2 | $n$ = 2 | necrotizing enterocolitis, hematochezia, elevated liver enzymes, vomiting, thrombocytosis in one patient, blood in stool | prednisolone (1 mg/kg/day) with increase to 2 mg/kg/day | reduced liver enzyme levels | unreported | Gaillard et al., 2023[125] |
| 19 | | | | | | $n$ = 25 | 11 days and 23 months | $n$ = 25 | $n$ = 25 | fever, respiratory distress, upper respiratory viral illness, thrombocytopenia, elevated liver enzyme levels | prednisolone (1 mg/kg/day) | reduced liver enzyme levels | no regression in motor abilities, gradual improvement in motor function and no recurrent infections and illnesses following OA better progress in motor function observed in patients who received OA earlier and who were presymptomatic | Tokatly Latzer et al., 2023[126] |
| 20 | | | | | | $n$ = 2 | 121 and 42 days | $n$ = 2 | unreported | unreported | unreported | unreported | patient 1 became bedridden despite receiving OA (extremities movement improved, predominantly in the upper extremities, and the paradoxical | Sawada et al., 2022[127] |

(Continued on next page)

**Table 4. Continued**

| No. | Disease | AAV gene therapy | Vector type | Dose | Route | No. of participants at time of treatment | Age at injection | No of patients dosed | No. of patients receiving immunosuppressants | AEs associated with AAV gene therapy | Immunosuppressive protocol | Clinical evidence of immunosuppression effectiveness | Clinical evidence of AAV gene therapy effectiveness | Reference |
|---|---|---|---|---|---|---|---|---|---|---|---|---|---|---|
| | | | | | | | | | | | | | respiration and tongue fasciculation disappeared the CHOP INTEND score decreased to 15 before treatment but improved to 34 after treatment he did not need respiratory support and could consume food orally at the age of 2 years and 1 month but had achieved no motor development milestones other than rolling over he is undergoing physical and occupational therapy). Patient 2 achieved normal motor development (head control, rolling over, sitting without support, standing with support, independent walking, beginning to run | |
| 21 | | | | | | *n* = 9 | 19–527 days | *n* = 9 | *n* = 9 | thrombocytopenia, elevated liver enzyme levels | prednisolone (1 mg/kg/day) | reduced liver enzyme levels | improvements of motor function 3/6 SMA type 1 patients required nutritional support, 4/6 developed scoliosis, 1/6 sleep disturbed breathing. SMA type 2 patient and two treated at pre-symptomatic stage did not require nutritional or respiratory support and did not develop a scoliosis | Stettner et al., 2023[128] |
| 22 | | | | | | *n* = 1 | 5 months | *n* = 1 | *n* = 1 | fever and loss of appetite, elevated liver enzyme levels, thrombocytopenia, hyper-ferritinemia | prednisolone (1 mg/kg/day) with increase to 2 mg/kg/day | reduced liver enzyme levels, normalized platelet count | improvement in motor function; acquired a stable sitting position, maintained kneeling position with anterior support, stand unaided with upper limbs support, no respiratory problems normal neurocognitive and speech profile, fed by | Tosi et al., 2022[129] |

(Continued on next page)

**Table 4. Continued**

| No. | Disease | AAV gene therapy | Vector type | Dose | Route | No. of participants at time of treatment | Age at injection | No of patients dosed | No. of patients receiving immunosuppressants | AEs associated with AAV gene therapy | Immunosuppressive protocol | Clinical evidence of immunosuppression effectiveness | Clinical evidence of AAV gene therapy effectiveness | Reference |
|---|---|---|---|---|---|---|---|---|---|---|---|---|---|---|
| | | | | | | | | | | | | | mouth and no swallowing problems | |
| 23 | | | | | | $n$ = 6 (5 type 1 and 1 type 2) | 7–24 months | $n$ = 6 | $n$ = 6 | elevated liver enzyme levels | prednisolone (1 mg/kg/day) | reduced GGT, ALT and AST levels | motor function improvements, no requirements for permanent ventilatory support and no case of mortality | Lee et al., 2022[130] |
| 24 | | | | | | $n$ = 7 | 7.5–19.2 months | $n$ = 7 | $n$ = 7 | fever and/or emesis, thrombocytopenia, elevated liver enzyme levels | prednisolone (1 mg/kg/day) | reduced ALT and AST levels, normal platelet count | motor function improvements | Matesanz et al., 2021[131] |
| 25 | | | | | | $n$ = 9 | ≤2 years | $n$ = 9 | $n$ = 9 | elevated liver enzyme levels, vomiting, reduced appetite, high prothrombin time, elevated bilirubin | prednisolone (1 mg/kg/day) | reduced liver enzyme levels | significant improvements in CHOP INTEND scores | Ali et al., 2021[132] |
| 26 | | | | | | $n$ = 10 | 19 months | $n$ = 10 | $n$ = 10 | hyperthermia, vomiting, lethargy and/or loose stool, thrombocytopenia, elevated liver enzyme levels | prednisolone (1 mg/kg/day) with increase to 2 mg/kg/day | reduced liver enzyme levels, reduced platelet count | relatively safe and effective with improvements of motor skills | Nevmerzhitskaya et al., 2021[133] |
| 27 | | | | | | $n$ = 21 | 0.65–24 months | $n$ = 21 | $n$ = 21 | vomiting, transaminitis and thrombocytopenia | prednisolone (1 mg/kg/day) | reduced liver enzyme levels, reduced platelet count | stabilization or improvement in bulbar or respiratory function | D'Silva et al., 2022[109] |
| 28 | | | | | | $n$ = 8 | 10–37 months | $n$ = 8 | $n$ = 8 | temporary increase in body temperature (above 38.5°C), vomiting, elevated liver enzyme levels, asymptomatic thrombocytopenia, 2/8 patients experienced an increase of pre-existing tremor | prednisolone (1 mg/kg/day) 7/8 patients; methylprednisolone (20 mg/kg) 1/8 patients | reduced liver enzyme levels, reduced platelet count, thrombocytes normalized | sitting without support, no respiratory support and tube feeding | Friese et al., 2021[65] |
| 29 | | | | | | Group 1 $n$ = 7; Group 2 $n$ = 6 | 2–6 months | $n$ = 7 | $n$ = 7 | fever, vomiting, lack of appetite, mild thrombocytopenia, elevated liver enzyme levels | prednisolone (1 mg/kg/day) | reduced liver enzyme levels | OV after nusinersen did not provide supplementary benefits for motor function or respiratory status. Early OV treatment results in better outcomes | Mirea et al., 2021[54] |
| 30 | | | | | | $n$ = 1 | 2 months | $n$ = 1 | $n$ = 1 | elevated liver enzyme levels | prednisolone (1 mg/kg/day) | reduced liver enzyme levels | head control at 4 months of age, independent walking at 18 months, pulling to stand, walk, and sit independently, and is reaching for objects; eating orally, gaining weight, no respiratory | Nigro et al., 2023[134] |

(Continued on next page)

**Table 4. *Continued***

| No. | Disease | AAV gene therapy | Vector type | Dose | Route | No. of participants at time of treatment | Age at injection | No of patients dosed | No. of patients receiving immunosuppressants | AEs associated with AAV gene therapy | Immunosuppressive protocol | Clinical evidence of immunosuppression effectiveness | Clinical evidence of AAV gene therapy effectiveness | Reference |
|---|---|---|---|---|---|---|---|---|---|---|---|---|---|---|
| | | | | | | | | | | | | | concerns, can say up to 10 words | |
| 31 | | | | | | $n$ = 1 | 4 months | $n$ = 1 | $n$ = 1 | moderate/severe TMA, hypertransaminasemia | prednisolone (1 mg/kg/day) | liver enzyme levels normalized | nusinersen: improvement in motor and bulbar function; onasmnogene abeparvovec: Sitting with support at 2 years old and acquired independent sitting at 27 months patient began to eat semisolids orally; risdiplam: further improvements in both motor and bulbar functions one year after risdiplam therapy | Bitetti et al., 2023[113] |

| No. | Disease | AAV gene therapy | Vector type | Dose | Route | No. of participants at time of treatment | Age at injection | No of patients dosed | No. of patients receiving immunosuppressants | AEs associated with AAV gene therapy | Immunosuppressive protocol | Clinical evidence of immunosuppression effectiveness | Clinical evidence of AAV gene therapy effectiveness | Reference |
|---|---|---|---|---|---|---|---|---|---|---|---|---|---|---|
| 1 | inherited retinal dystrophy (RPE-65 mediated) | VN | AAV2 | $1.5 \times 10^{11}$ vg per eye | Subretinal | $n$ = 3 | 22 months, 2 years, 5 years | $n$ = 3 | $n$ = 3 | $n$ = 3 acute subretinal deposits | prednisolone (1 mg/kg/day) | N/A | Improved visual function with macular and inferior subretinal deposits improved or resolved | Lopez et al., 2023[135] |
| 2 | inherited retinal dystrophy (RPE-65 mediated) | VN | AAV2 | $1.5 \times 10^{11}$ vg per eye | Subretinal | $n$ = 6 | 18–49 years | $n$ = 6 | $n$-6 | retinal atrophy in 10/12 eyes (8 mild/2 severe) increased ocular pressure (3 patients: 6 eyes) Increased intraocular inflammation (2 eyes) cataracts (4 eyes) glaucoma surgery (2 patients: 4 eyes) higher occurrence of retinal atrophy and increased IOP than previously reported | prednisolone (1 mg/kg/day) | N/A | best-corrected visual acuity remained stable (baseline: 1.28 (±0.71) vs. last follow-up: 1.46 (±0.60); $p$ = 0.25). Average white Full-Field Stimulus Testing (FST) showed a trend toward improvement (baseline: −4.41 (±10.62) dB vs. last follow-up: −11.98 (±13.83) dB; $p$ = 0.18). | Kiraly et al., 2023[55] |
| 3 | inherited retinal dystrophy (RPE-65 mediated) | VN | AAV2 | $1.5 \times 10^{11}$ vg per eye | subretinal | $n$ = 1 | 39 years | $n$ = 1 | $n$ = 1 | foveal ellipsoid zone loss | prednisolone (1 mg/kg/day) | N/A | the first eye showed improvement in rod photoreceptor function with increased peripheral and low luminance vision (baseline VA: 0.9 logMAR and 2-years post-operative VA: 0.7 logMAR. 2ND eye developed loss of foveal photoreceptors. FST improvements were maintained in both eyes. Macular oedema resolved by 6 weeks of VN | Jalil et al., 2023[136] |

(*Continued on next page*)

**Table 4. Continued**

| No. | Disease | AAV gene therapy | Vector type | Dose | Route | No. of participants at time of treatment | Age at injection | No of patients dosed | No. of patients receiving immunosuppressants | AEs associated with AAV gene therapy | Immunosuppressive protocol | Clinical evidence of immunosuppression effectiveness | Clinical evidence of AAV gene therapy effectiveness | Reference |
|---|---|---|---|---|---|---|---|---|---|---|---|---|---|---|
| 4 | hemophilia B | etranacogene dezaparvovec | AAV5 | $2 \times 10^{13}$ vg/kg | intravenous | *n* = 3 | 43, 47 and 50 years | *n* = 3 | *n* = 3 | headache<br>transient elevation of C-reactive protein | N/A | N/A | stable durable FIX activity remained after 3 years<br>discontinued FIX prophylaxis in all patients<br>clinical phenotype from severe/moderately severe to mild/nonhemophilic<br>100% decrease in bleeds in 2/3 and 92% in patient 3 | Von Drygalski et al., 2019[106] |

GGT, gamma-glutamyl transferase, FIX, factor IX; N/A, not applicable; ULN, upper limit of normal.

immunosuppressive protocols. For example, subretinal AAV vector administration performed in several clinical studies of gene transfer for RPE65 deficiency[7,9] was generally associated with little to no detectable immune response to the capsid or the transgene.

AEs were observed in 30 clinical trials, with the most frequent being increased liver enzyme levels, vomiting and nausea, fever, and fatigue. SAEs were observed in eight studies, with SMA and hemophilia B being the main diseases associated with SAEs. Corticosteroids, including prednisolone, were used to manage these events, even in studies where corticosteroids were administered prophylactically. Interestingly, a phase 1/2 clinical trial for hemophilia B gene therapy did not find a clear association between the resolution of elevated liver enzyme levels and prednisolone use.[59]

To decrease AAV immunogenicity, one focus is on decreasing different aspects of immune responses against the capsid to improve patient safety profile and safeguard long-term transgene expression.[73] The use of less seroprevalent capsids may reduce the recognition of preformed NAbs.[73] One particularly promising strategy is capsid engineering, which has the potential to develop next-generation vectors with multiple improvements compared to current vectors. Current AAV vectors still face limitations in delivering efficiently to specific tissues, often requiring high doses. Higher vector doses have been associated with hepatotoxicity, TMA, and other immune-mediated AEs.[73] Additionally, enhancing tropism and transduction efficiency alone would allow for lower doses and significantly decrease toxicity from AAV immunity.[21]

One important obstacle is neutralizing anti-AAV antibodies that display a major short- and long-term barrier in delivering gene therapy. Humoral responses against AAV capsid proteins are evaluated in patients before AAV treatment in some trials, and patients with detectable pre-existing antibodies to AAV are excluded. Rates of NAb prevalence in the general population are quoted as 30%–70% in literature against all various serotypes, with the highest prevalence against AAV2, followed by AAV1.[36,75,76] In addition, patients who develop *de novo* antibodies after systemic AAV gene therapy have no access to eventual redosing attempts in case of loss of therapy efficacy. Even though gene therapies are designed to be administered only once, the immune response is one factor that jeopardizes the long-term efficacy of transgene expression, which highlights the need for potential redosing strategies for patients. For example, in a recently approved therapy for hemophilia A (Roctavian), factor VIII activity levels declined over 3 years from 52.6 to 18.2 IU/dL. Interestingly, 79% of these 134 patients received corticosteroids in response to ALT elevations. Despite the introduction of corticosteroids, an overall decrease in factor VIII levels was present.[87] Not only do immune responses prevent the repeated administration of AAV, but AAV immunity can lead to severe toxicity and SAEs, potentially resulting in patient death.[21]

To enable redosing, pre-clinical work (including plasmapheresis monoclonal antibody directed to plasma cells and imlifidase

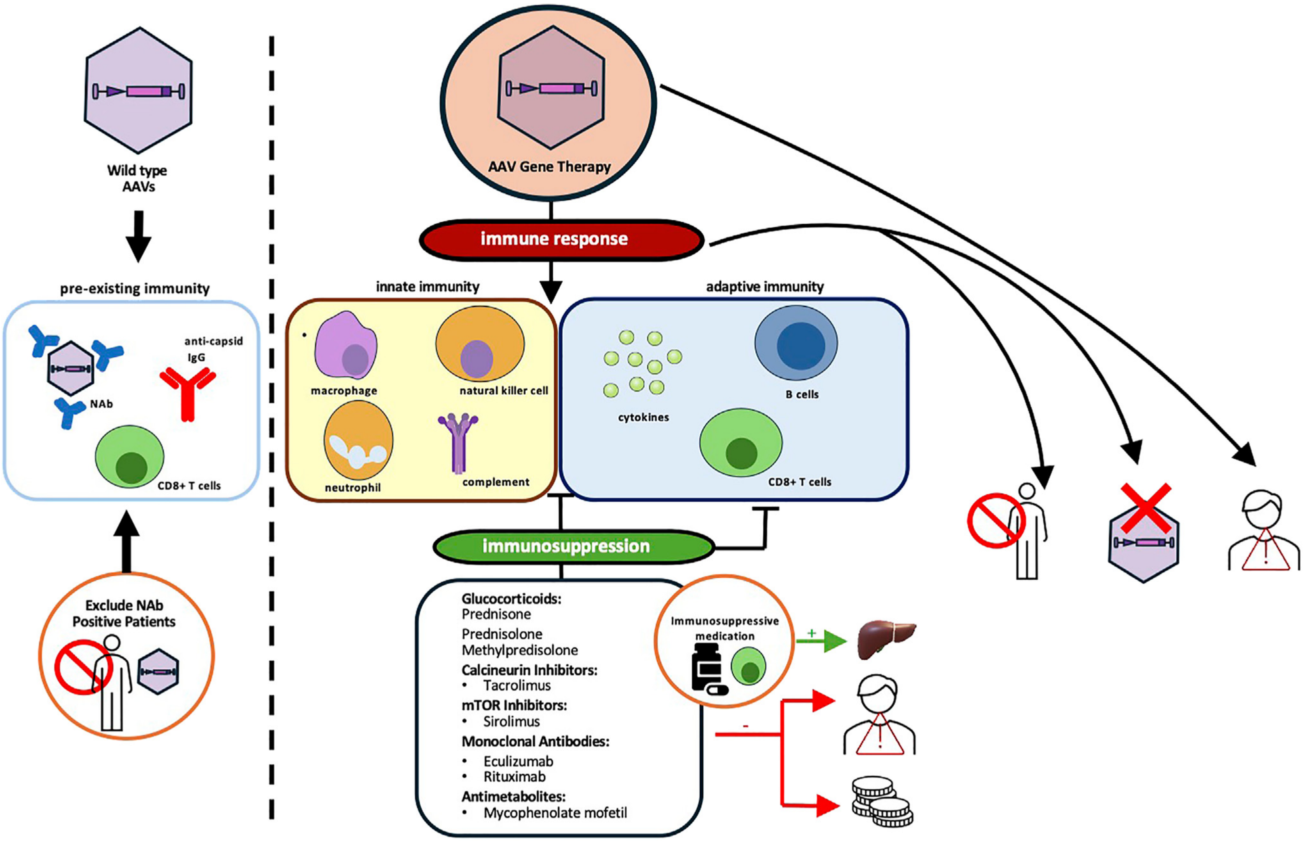


**Figure 2. Graphic summary of findings**

AAV-based gene therapy treatment can induce pre-existing immunity, innate immunity, and adaptive immune response against AAV vectors and transgenic products, inhibiting AAV expression. Patients with pre-existing immunity are unable to receive AAV-based gene therapy treatment. Immunosuppression protocols are used to suppress the immune response and elevate treatment-related AEs, such as the normalization of liver enzymes. Immunosuppression, although potentially essential for some patients to ensure therapeutic benefits of AAV-mediate gene transfer, comes at high costs.

injection) has been conducted to evaluate how animals with pre-existing AAV antibodies could be successfully and safely transfected with a transgene carried by an AAV vector.[88,89] In the version of a potential treatment with a second dose of gene therapy, protocols have been developed to tamper the immune reaction during the infusion of the first dose, such as co-administration of sirolimus nanoparticles.[73–77,87–90] Research findings have shown that incorporating sirolimus into synthetic vaccine particles, co-administered with AAV vectors, hinders the induction of cell-mediated and anti-capsid humoral responses. This results in the inhibition of CD8+ T cell infiltration in the liver, reduction of B and T cell activation, and suppression of memory T cell response in both mice and non-human primates.[88] To our best knowledge, this has not yet been translated *in vivo* in humans.

In this review, a variety of immunosuppressive treatments were noted, including different corticosteroids, tacrolimus, mycophenolate mofetil, cyclosporine, sirolimus, and rituximab. Corticosteroids were the most widely used immunosuppressants for AAV gene therapy clinical studies (Table 3), either alone or in combination with other drugs, for their global inhibitory effects on innate and adaptive immunity. Corticosteroids are used as immunosuppressants for approved gene therapy products[91] (VN[8] and OA[11]), due to their established safety profiles and effects in preventing or alleviating immune responses. However, the drawbacks of corticosteroids are immunosuppression-associated side effects and non-specific immunosuppression, in particular increased susceptibility to infection.[92,93] Therefore, careful monitoring and appropriate prophylactic measures should be taken to minimize the risk of infections in patients receiving immunosuppressive treatments. The doses of prednisolone seen in this review ranged from 1 to 2 mg/kg with a total daily dose in the range of 30–60 mg/day. The duration of corticosteroid treatment ranged dramatically between 7 and 133 days, with other immunosuppressants being used for up to 365 days. This highlights the disparity between studies on the longevity of immunosuppression and differing opinions of where the balance of reducing AAV gene therapy immunogenicity versus the side effects of long-term immunosuppression should be struck. Work is ongoing to refine the immunosuppression regimen with the goal of decreasing vector-related immune response in the early period after treatment. For example, Chowdary et al.[48]

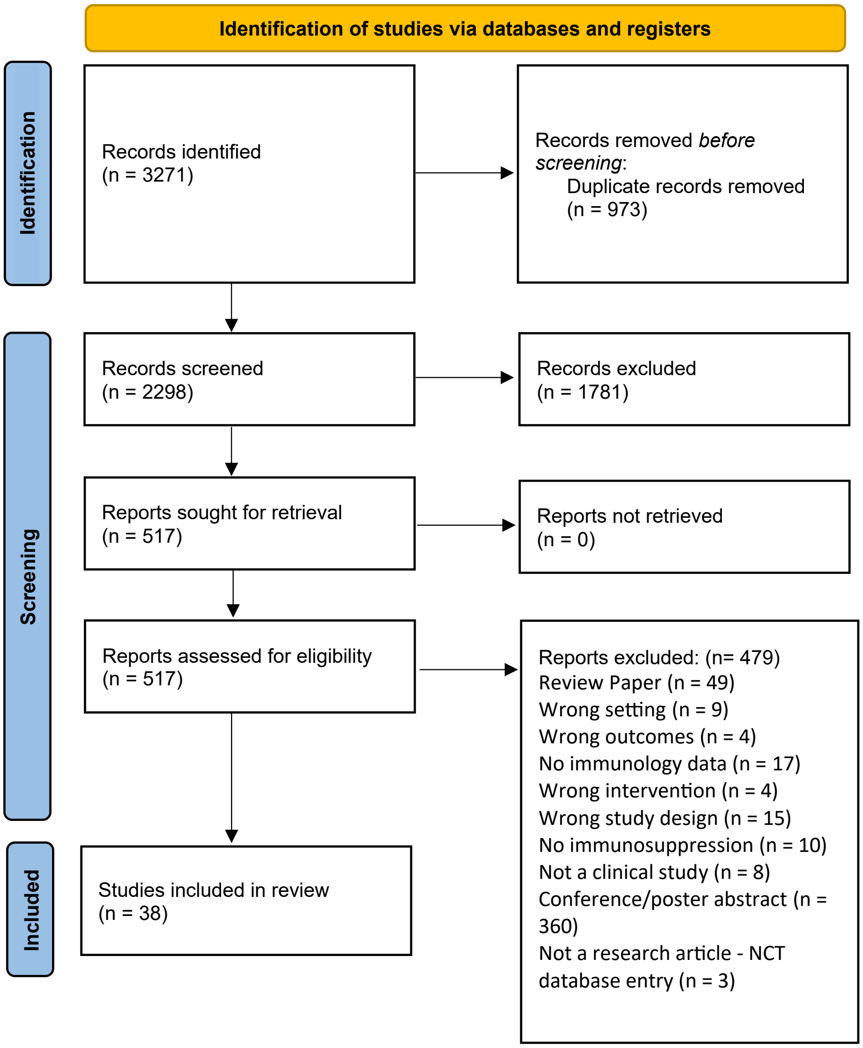


**Figure 3. PRISMA flow of the selection process of clinical trials**

adopted a prophylactic immunosuppression regimen to improve the predictability of the dose-response and to increase the chances that normal factor IX levels would be reached and maintained in patients with hemophilia B.

In a review from Oh et al.[93] investigating steroid-associated side effects in patients with primary proteinuric kidney disease, 62% of patients exposed to steroids developed one SAE. The rate of hypertension was 1.4 times higher than before steroid exposure. Interestingly, the risk of metabolic complications (e.g., diabetes mellitus, overweight, obesity) was also significantly higher after steroid therapy exposure. Taken together, a patient's adjusted risk of these complications following steroid exposure was 1.5–1.8 times that of the risk before exposure. Other studies, such as Movahedi et al., which analyzed UK and US national databases for nearly 22,000 patients with rheumatoid arthritis, showed that the hazard ratio of steroid-associated diabetes was 1.30 and 1.61, respectively.[94] Another small Japanese study showed corticosteroid-induced diabetes developed in 17–42 patients (40.5%) undergoing steroid therapy for primary renal disease.[95] In our analysis, often the duration and the dosing regimen of steroid exposure was not always mentioned, and the overall sample size of patients undergoing gene therapy is rather small. In the hemophilia study with valoctocogene roxaparvovec, which has the highest number of patients, the authors reported similar AE rates comparable with the aforementioned publications. In summary, steroid use is related to side effects in gene therapy. Due to the given limitations, the full spectrum and rate of AEs in the context of gene therapy cannot be retrieved based on the current available data, but the available data is in keeping with known side effect profiles seen in high-dose steroid use in other conditions.

Although a wide range of immunosuppressive agents were identified in the study, there is insufficient data available to determine if any of these conferred an additional safety benefit. Only one-quarter of studies reported antibody titers against the vector and transgene, and even fewer evaluated other immune cell responses, e.g., Tregs. Different immunosuppressive drugs have varying modes of action, for example, corticosteroids are non-specific,[91] mycophenolate motefil depletes both T and B cell populations,[50,93] sirolimus inhibits T and B cell activation and induces Tregs through targeting mTOR,[93] whereas rituximab is an anti-CD20 monoclonal antibody that depletes B cells by inducing apoptosis.[96] Therefore, better characterization of the different immune cell responses in the future would help identify if any of these agents are more efficacious in decreasing gene therapy immunogenicity and would also identify if additional cell populations need targeting. No agent identified in these studies specifically targeted innate immune responses, although this could be an important direction for the future. As well as engineering transgene to lower their immunogenic CpG content,[97] there are now C3 inhibitors, e.g., APL-9, which have been used during AAV delivery with the aim of dampening the complement pathway.[98] Some studies already used a combination of immunosuppressive agents to broaden the cell populations they covered; however, with this, the increased likelihood of side effects must be considered. Balancing the risk-benefit profile of immunosuppression continues to be a great challenge for the gene therapy field going forward. Recent prospective trials tested the combination of mTOR inhibitor sirolimus in combination with rituximab.[3,99] Preclinical hemophilia A

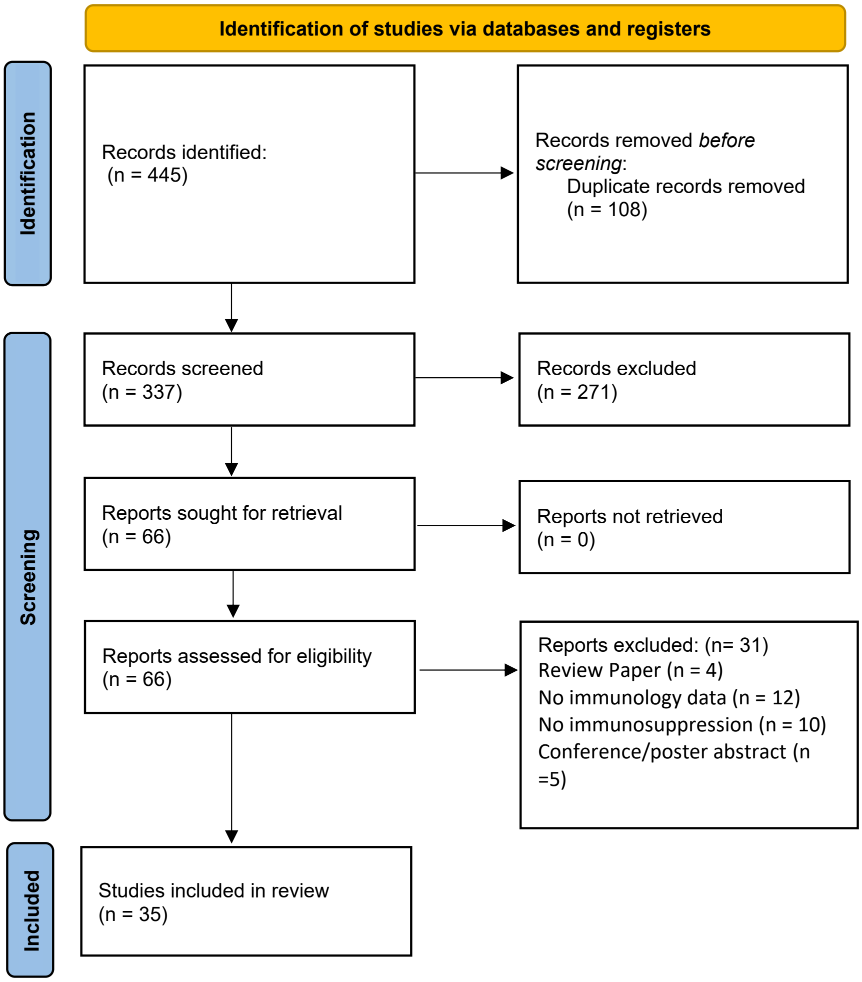


**Figure 4. PRISMA flow of the selection process of real-world studies**

mice models showed that under this combined immunosuppressive therapy, there was no increase in inhibitors following rechallenging with factor VIII protein.[100] The combined therapy with rituximab and sirolimus was also used in Pompe disease and GM2-gangliosidosis to enable re-administration of AAV.[3,101] Despite this first promising data, this drug combination must show its benefit in future trials for patients without sacrificing patient safety.

Gene therapy using AAV vectors shows promise for treating rare genetic diseases. However, the high costs and risks associated with developing these therapies must be considered in the real-world deployment of these therapies (Figure 2). Conducting robust clinical trials in rare diseases can present complexity and challenges but are necessary to assess the risks and benefits. Improving safety, immunosuppressive regimens, patient risk assessment, and comparing gene therapy to other treatments are key factors to consider.

### Conclusion

This review provides valuable information on the immunosuppressive protocols used alongside various gene therapy treatments. Understanding and minimizing immunological responses is crucial for optimizing treatment protocols and ensuring the safety and efficacy of gene therapy interventions. Further research and adherence to evaluation recommendations will contribute to the development of more effective and safer gene therapy approaches in the future.

## METHODS

An information specialist (E.H.) searched the following bibliographic databases on May 3, 2024, for studies published from database inception to the search date: Ovid Embase, Ovid MEDLINE, and the Cochrane Register of Controlled Trials. We searched using terms related to monogenic disorders, gene therapy, cell therapy with genetically modified cells, immune modulation, and immune suppression, limited to clinical trial records only. The search strategies for Ovid Embase and Ovid MEDLINE used adapted versions of the Cochrane Highly Sensitive Search Strategies for identifying controlled trials. The full strategies are available in the appendix. All references were exported to EndNote X21 (Thomson Reuters, New York, NY, USA), and duplicates were removed manually. Covidence software was used to manage screening and eligibility. A primary search of the indicated electronic databases resulted in a total of 3,271 articles (Figure 3). After the removal of duplicates, the remaining studies were screened based on their titles and abstracts by two independent researchers. A total of 517 articles remained after this screening stage and were then evaluated based on their full texts, resulting in 38 clinical trial studies in the final meta-analysis. We excluded literature reviews and conference articles.

In addition, 445 real-world studies, case reports, or observational studies were found through a systematic search in the same three databases and registry on May 3, 2024, for studies published from database inception to the search date. We searched using terms related to monogenic disorders, gene therapy, cell therapy with genetically modified cells, immune modulation, and immune suppression, limited to observational studies, case reports, and real-world studies. Conference abstracts and posters were included from Ovid Embase. Again, the full strategies are available in the Appendix. All references were exported to EndNote 21 (Thomson Reuters), and duplicates were removed manually. As shown in the PRISMA flow diagram[138] (Figure 4), 337 articles were screened after

duplications were eliminated. A total of 35 real-world studies were included in the meta-analysis.

## SUPPLEMENTAL INFORMATION

Supplemental information can be found online at https://doi.org/10.1016/j.ymthe.2024.07.016.

## ACKNOWLEDGMENTS

This project has received funding from the Innovative Medicines Initiative 2 Joint Undertaking (JU) under grant agreement no. 945473. The JU receives support from the European Union Horizon 2020 research and innovation program and EFPIA. M.A. is also supported by the European Research Council grant (ERC Advanced Award no. 294745), MRC DPFS Award (129016), JPND-MRC (MR/V000470/1), ARUK award (ARUK-PG2018B-005), LifeArc (No. 163978 and P2022-0004, Recipient: 176666), and MRC/LifeArc award (MR/V030140/1). J.K. is also supported by LifeArc/MRC/BBSRC (MR/V030140/1) and the MND Association (Kirby/Feb22/969-794). The Innovative Medicines Initiative (IMI; imi-europe.org) and the ARDAT (Accelerating Research & Development for Advanced Therapies) consortium was formed in 2020 to fund 5 years of research into AAV biology. The overall objective of ARDAT is to develop and provide the data and tools to fill gaps in our knowledge base in the field of immunology and gene therapy and so accelerate the research and development of new AAV therapies for rare diseases.

## AUTHOR CONTRIBUTIONS

B.V., I.S.H., R.H., C.P.W., E.H., F.M., C.B., J.S., M.L., J.E.M., M.D., P.J.S., J.K., M.A., and L.S. conceptualized, designed, extracted data from relevant studies, and assisted with the draft/approval of this manuscript. E.H. carried out a systematic search.
The ARDAT study group also includes Dan Levy, Veres Gabor, Jon Gilbin, Christopher Mann, Francesca Biondo, and Manuela Braun.

## DECLARATION OF INTERESTS

M.A. is the academic founder and Chief Scientific Officer of BlackfinBio. M.A. is a co-founder of Crucible Therapeutics. L.S. has given lectures/consultancy for Pfizer/RegenexBio/Sarepta/Novartis and Illumina. J.K. has received research funding from Quell Therapeutics.

## REFERENCES

1. Wagner, J.A., Reynolds, T., Moran, M.L., Moss, R.B., Wine, J.J., Flotte, T.R., and Gardner, P. (1998). Efficient and persistent gene transfer of AAV-CFTR in maxillary sinus. Lancet *351*, 1702–1703. https://doi.org/10.1016/S0140-6736(05)77740-0.
2. Zuckerman, J.B., Robinson, C.B., McCoy, K.S., Shell, R., Sferra, T.J., Chirmule, N., Magosin, S.A., Propert, K.J., Brown-Parr, E.C., Hughes, J.V., et al. (1999). A phase I study of adenovirus-mediated transfer of the human cystic fibrosis transmembrane conductance regulator gene to a lung segment of individuals with cystic fibrosis. Hum. Gene Ther. *10*, 2973–2985. https://doi.org/10.1089/10430349950016384. PMID: 10609658.
3. Flotte, T.R., Cataltepe, O., Puri, A., Batista, A.R., Moser, R., McKenna-Yasek, D., Douthwright, C., Gernoux, G., Blackwood, M., Mueller, C., et al. (2022). AAV gene therapy for Tay-Sachs disease. Nat. Med. *28*, 251–259. https://doi.org/10.1038/s41591-021-01664-4.
4. Marshall, E. (1999). Gene therapy death prompts review of adenovirus vector. Science *286*, 2244–2245. https://doi.org/10.1126/science.286.5448.2244.
5. Somanathan, S., Calcedo, R., and Wilson, J.M. (2020). Adenovirus-Antibody Complexes Contributed to Lethal Systemic Inflammation in a Gene Therapy Trial. Mol. Ther. *28*, 784–793. https://doi.org/10.1016/j.ymthe.2020.01.006.
6. Gaudet, D., Méthot, J., Déry, S., Brisson, D., Essiembre, C., Tremblay, G., Tremblay, K., de Wal, J., Twisk, J., van den Bulk, N., et al. (2013). Efficacy and long-term safety of alipogene tiparvovec (AAV1-LPLS447X) gene therapy for lipoprotein lipase deficiency: an open-label trial. Gene Ther. *20*, 361–369. https://doi.org/10.1038/gt.2012.43.
7. Maguire, A.M., Simonelli, F., Pierce, E.A., Pugh, E.N., Jr., Mingozzi, F., Bennicelli, J., Banfi, S., Marshall, K.A., Testa, F., Surace, E.M., et al. (2008). Safety and efficacy of gene transfer for Leber's congenital amaurosis. N. Engl. J. Med. *358*, 2240–2248. https://doi.org/10.1056/NEJMoa0802315.
8. Russell, S., Bennett, J., Wellman, J.A., Chung, D.C., Yu, Z.-F., Tillman, A., Wittes, J., Pappas, J., Elci, O., McCague, S., et al. (2017). Efficacy and safety of Voretigene NEPARVOVEC (AAV2-hrpe65v2) in patients with RPE65 -mediated inherited retinal dystrophy: A randomised, controlled, open-label, phase 3 trial. The Lancet *390*, 849–860. https://doi.org/10.1016/s0140-6736(17)31868-8.
9. Maguire, A.M., High, K.A., Auricchio, A., Wright, J.F., Pierce, E.A., Testa, F., Mingozzi, F., Bennicelli, J.L., Ying, G.S., Rossi, S., et al. (2009). Age-dependent effects of RPE65 gene therapy for Leber's congenital amaurosis: a phase 1 dose-escalation trial. Lancet *374*, 1597–1605.
10. Simonelli, F., Maguire, A.M., Testa, F., Pierce, E.A., Mingozzi, F., Bennicelli, J.L., Rossi, S., Marshall, K., Banfi, S., Surace, E.M., et al. (2010). Gene therapy for Leber's congenital amaurosis is safe and effective through 1.5 years after vector administration. Mol. Ther. *18*, 643–650. https://doi.org/10.1038/mt.2009.277.
11. Mendell, J.R., Al-Zaidy, S., Shell, R., Arnold, W.D., Rodino-Klapac, L.R., Prior, T.W., Lowes, L., Alfano, L., Berry, K., Church, K., et al. (2017). Single-Dose Gene-Replacement Therapy for Spinal Muscular Atrophy. N. Engl. J. Med. *377*, 1713–1722. https://doi.org/10.1056/NEJMoa1706198.
12. Day, J.W., Finkel, R.S., Chiriboga, C.A., Connolly, A.M., Crawford, T.O., Darras, B.T., Iannaccone, S.T., Kuntz, N.L., Peña, L.D.M., Shieh, P.B., et al. (2021). Onasemnogene abeparvovec gene therapy for symptomatic infantile-onset spinal muscular atrophy in patients with two copies of SMN2 (STR1VE): an open-label, single-arm, multicentre, phase 3 trial. Lancet Neurol. *20*, 284–293. https://doi.org/10.1016/S1474-4422(21)00001-6.
13. Mercuri, E., Muntoni, F., Baranello, G., Masson, R., Boespflug-Tanguy, O., Bruno, C., Corti, S., Daron, A., Deconinck, N., Servais, L., et al. (2021). Onasemnogene abeparvovec gene therapy for symptomatic infantile-onset spinal muscular atrophy type 1 (STR1VE-EU): an open-label, single-arm, multicentre, phase 3 trial. Lancet Neurol. *20*, 832–841.
14. Mendell, J.R., Al-Zaidy, S.A., Lehman, K.J., McColly, M., Lowes, L.P., Alfano, L.N., Reash, N.F., Iammarino, M.A., Church, K.R., Kleyn, A., et al. (2021). Five-year extension results of the phase 1 START trial of onasemnogene abeparvovec in spinal muscular atrophy. JAMA Neurol. *78*, 834–841.
15. Mahlangu, J., Kaczmarek, R., von Drygalski, A., Shapiro, S., Chou, S.C., Ozelo, M.C., Kenet, G., Peyvandi, F., Wang, M., Madan, B., et al. (2023). Two-Year Outcomes of Valoctocogene Roxaparvovec Therapy for Hemophilia A. N. Engl. J. Med. *388*, 694–705. https://doi.org/10.1056/NEJMoa2211075.
16. Pipe, S.W., Leebeek, F.W.G., Recht, M., Key, N.S., Castaman, G., Miesbach, W., Lattimore, S., Peerlinck, K., Van der Valk, P., Coppens, M., et al. (2023). Gene Therapy with Etranacogene Dezaparvovec for Hemophilia B. N. Engl. J. Med. *388*, 706–718. https://doi.org/10.1056/NEJMoa2211644.
17. (2023). Sarepta Therapeutics announces FDA approval of ELEVIDYS, the first gene therapy to treat Duchenne muscular dystrophy (Sarepta Therapeutics). https://investorrelations.sarepta.com/news-releases/news-release-details/sarepta-therapeutics-announces-fda-approval-elevidys-first-gene.
18. (2023). FDA approves first gene therapy for treatment of certain patients with Duchenne muscular dystrophy (FDA). https://www.fda.gov/news-events/press-announcements/fda-approves-first-gene-therapy-treatment-certain-patients-duchenne-muscular-dystrophy.

19. Au, H.K.E., Isalan, M., and Mielcarek, M. (2021). Gene Therapy Advances: A Meta-Analysis of AAV Usage in Clinical Settings. Front. Med. *8*, 809118. https://doi.org/10.3389/fmed.2021.809118.

20. Arabi, F., Mansouri, V., and Ahmadbeigi, N. (2022). Gene therapy clinical trials, where do we go? An overview. Biomed. Pharmacother. *153*, 113324. https://doi.org/10.1016/j.biopha.2022.113324.

21. Horton, R.H., Saade, D., Markati, T., Harriss, E., Bönnemann, C.G., Muntoni, F., and Servais, L. (2022). A systematic review of adeno-associated virus gene therapies in neurology: The need for consistent safety monitoring of a promising treatment. J. Neurol. Neurosurg. Psychiatry *93*, 1276–1288. https://doi.org/10.1136/jnnp-2022-329431.

22. Servais, L., Horton, R., Saade, D., Bonnemann, C., and Muntoni, F.; 261st ENMC workshop study group (2023). 261st ENMC International Workshop: Management of safety issues arising following AAV gene therapy. 17th-19th June 2022, Hoofddorp, the Netherlands. Neuromuscul. Disord. *33*, 884–896. https://doi.org/10.1016/j.nmd.2023.09.008.

23. Mercuri, E., Deconinck, N., Mazzone, E.S., Nascimento, A., Oskoui, M., Saito, K., Vuillerot, C., Baranello, G., Boespflug-Tanguy, O., Goemans, N., et al. (2022). Safety and efficacy of once-daily risdiplam in type 2 and non-ambulant type 3 spinal muscular atrophy (sunfish part 2): A phase 3, double-blind, randomised, placebo-controlled trial. Lancet Neurol. *21*, 42–52. https://doi.org/10.1016/s1474-4422(21)00367-7.

24. Strauss, K.A., Farrar, M.A., Muntoni, F., Saito, K., Mendell, J.R., Servais, L., McMillan, H.J., Finkel, R.S., Swoboda, K.J., Kwon, J.M., et al. (2022). Onasemnogene abeparvovec for presymptomatic infants with three copies of SMN2 at risk for spinal muscular atrophy: the Phase III SPR1NT trial. Nat. Med. *28*, 1390–1397. https://doi.org/10.1038/s41591-022-01867-3.

25. Guillou, J., de Pellegars, A., Porcheret, F., Frémeaux-Bacchi, V., Allain-Launay, E., Debord, C., Denis, M., Péréon, Y., Barnérias, C., Desguerre, I., et al. (2022). Fatal thrombotic microangiopathy case following adeno-associated viral SMN gene therapy. Blood Adv. *6*, 4266–4270. https://doi.org/10.1182/bloodadvances.2021006419.

26. Chand, D.H., Zaidman, C., Arya, K., Millner, R., Farrar, M.A., Mackie, F.E., Goedeker, N.L., Dharnidharka, V.R., Dandamudi, R., and Reyna, S.P. (2021). Thrombotic Microangiopathy Following Onasemnogene Abeparvovec for Spinal Muscular Atrophy: A Case Series. J. Pediatr. *231*, 265–268. https://doi.org/10.1016/j.jpeds.2020.11.054.

27. Arnold, D.M., Patriquin, C.J., and Nazy, I. (2017). Thrombotic microangiopathies: A general approach to diagnosis and management. Can. Med. Assoc. J. *189*, E153–E159. https://doi.org/10.1503/cmaj.160142.

28. Li, C., and Samulski, R.J. (2020). Engineering adeno-associated virus vectors for gene therapy. Nat. Rev. Genet. *21*, 255–272. https://doi.org/10.1038/s41576-019-0205-4.

29. Boutin, S., Monteilhet, V., Veron, P., Leborgne, C., Benveniste, O., Montus, M.F., and Masurier, C. (2010). Prevalence of serum IGG and neutralizing factors against adeno-associated virus (AAV) types 1, 2, 5, 6, 8, and 9 in the healthy population: Implications for gene therapy using AAV vectors. Hum. Gene Ther. *21*, 704–712. https://doi.org/10.1089/hum.2009.182.

30. Ronzitti, G., Gross, D.-A., and Mingozzi, F. (2020). Human immune responses to adeno-associated virus (AAV) vectors. Front. Immunol. *11*, 670. https://doi.org/10.3389/fimmu.2020.00670.

31. Ho, A., Orton, R., Tayler, R., Asamaphan, P., Herder, V., Davis, C., Tong, L., Smollett, K., Manali, M., Allan, J., et al. (2023). Adeno-associated virus 2 infection in children with non-A-E hepatitis. Nature *617*, 555–563. https://doi.org/10.1038/s41586-023-05948-2.

32. Morfopoulou, S., Buddle, S., Torres Montaguth, O.E., Atkinson, L., Guerra-Assunção, J.A., Moradi Marjaneh, M., Zennezini Chiozzi, R., Storey, N., Campos, L., Hutchinson, J.C., et al. (2023). Genomic investigations of unexplained acute hepatitis in children. Nature *617*, 564–573. https://doi.org/10.1038/s41586-023-06003-w.

33. Servellita, V., Sotomayor Gonzalez, A., Lamson, D.M., Foresythe, A., Huh, H.J., Bazinet, A.L., Bergman, N.H., Bull, R.L., Garcia, K.Y., Goodrich, J.S., et al. (2023). Adeno-associated virus type 2 in US children with acute severe hepatitis. Nature *617*, 574–580. https://doi.org/10.1038/s41586-023-05949-1.

34. Schulz, M., Levy, D.I., Petropoulos, C.J., Bashirians, G., Winburn, I., Mahn, M., Somanathan, S., Cheng, S.H., and Byrne, B.J. (2023). Binding and neutralizing anti-AAV antibodies: Detection and implications for rAAV-mediated gene therapy. Mol. Ther. *31*, 616–630. https://doi.org/10.1016/j.ymthe.2023.01.010.

35. Li, C., Narkbunnam, N., Samulski, R.J., Asokan, A., Hu, G., Jacobson, L.J., Manco-Johnson, M.J., and Monahan, P.E.; Joint Outcome Study Investigators (2012). Neutralizing antibodies against adeno-associated virus examined prospectively in pediatric patients with hemophilia. Gene Ther. *19*, 288–294. https://doi.org/10.1038/gt.2011.90.

36. Calcedo, R., Vandenberghe, L.H., Gao, G., Lin, J., and Wilson, J.M. (2009). Worldwide epidemiology of neutralizing antibodies to adeno-associated viruses. J. Infect. Dis. *199*, 381–390. https://doi.org/10.1086/595830.

37. Li, C., Hirsch, M., DiPrimio, N., Asokan, A., Goudy, K., Tisch, R., and Samulski, R.J. (2009). Cytotoxic-T-lymphocyte-mediated elimination of target cells transduced with engineered adeno-associated virus type 2 vector in vivo. J. Virol. *83*, 6817–6824. https://doi.org/10.1128/jvi.00278-09.

38. Manno, C.S., Pierce, G.F., Arruda, V.R., Glader, B., Ragni, M., Rasko, J.J., Ozelo, M.C., Hoots, K., Blatt, P., Konkle, B., et al. (2006). Successful transduction of lid in hemophilia by Aav-Factor IX and limitations imposed by the host immune response. Nat. Med. *12*, 342–347. https://doi.org/10.1038/nm1358.

39. Fitzpatrick, Z., Leborgne, C., Barbon, E., Masat, E., Ronzitti, G., van Wittenberghe, L., Vignaud, A., Collaud, F., Charles, S., Simon Sola, M., et al. (2018). Influence of pre-existing anti-capsid neutralizing and binding antibodies on AAV vector transduction. Mol. Ther. Methods Clin. Dev. *9*, 119–129. https://doi.org/10.1016/j.omtm.2018.02.003.

40. Harbison, C.E., Weichert, W.S., Gurda, B.L., Chiorini, J.A., Agbandje-McKenna, M., and Parrish, C.R. (2012). Examining the cross-reactivity and neutralization mechanisms of a panel of mAbs against adeno-associated virus serotypes 1 and 5. J. Gen. Virol. *93*, 347–355. https://doi.org/10.1099/vir.0.035113-0.

41. Wobus, C.E., Hügle-Dörr, B., Girod, A., Petersen, G., Hallek, M., and Kleinschmidt, J.A. (2000). Monoclonal antibodies against the adeno-associated virus type 2 (AAV-2) capsid: epitope mapping and identification of capsid domains involved in AAV-2-cell interaction and neutralization of AAV-2 infection. J. Virol. *74*, 9281–9293. https://doi.org/10.1128/jvi.74.19.9281-9293.2000.

42. Tseng, Y.S., and Agbandje-McKenna, M. (2014). Mapping the AAV capsid host antibody response toward the development of second generation gene delivery vectors. Front. Immunol. *5*, 9. https://doi.org/10.3389/fimmu.2014.00009.

43. Nathwani, A.C., Tuddenham, E.G.D., Rangarajan, S., Rosales, C., McIntosh, J., Linch, D.C., Chowdary, P., Riddell, A., Pie, A.J., Harrington, C., et al. (2011). Adenovirus-associated virus vector-mediated gene transfer in hemophilia B. N. Engl. J. Med. *365*, 2357–2365. https://doi.org/10.1056/NEJMoa1108046.

44. Nathwani, A.C., Reiss, U.M., Tuddenham, E.G.D., Rosales, C., Chowdary, P., McIntosh, J., Della Peruta, M., Lheriteau, E., Patel, N., Raj, D., et al. (2014). Long-term safety and efficacy of factor IX gene therapy in hemophilia B. N. Engl. J. Med. *371*, 1994–2004. https://doi.org/10.1056/nejmoa1407309.

45. Tardieu, M., Zérah, M., Husson, B., de Bournonville, S., Deiva, K., Adamsbaum, C., Vincent, F., Hocquemiller, M., Broissand, C., Furlan, V., et al. (2014). Intracerebral administration of adeno-associated viral vector serotype RH.10 carrying human *sgsh* and *sumf1* cdnas in children with mucopolysaccharidosis type IIIA disease: Results of a phase I/II trial. Hum. Gene Ther. *25*, 506–516. https://doi.org/10.1089/hum.2013.238.

46. Coutinho, A.E., and Chapman, K.E. (2011). The anti-inflammatory and immunosuppressive effects of glucocorticoids, recent developments and mechanistic insights. Mol. Cell. Endocrinol. *335*, 2–13. https://doi.org/10.1016/j.mce.2010.04.005.

47. Broering, R., Montag, M., Jiang, M., Lu, M., Sowa, J.P., Kleinehr, K., Gerken, G., and Schlaak, J.F. (2011). Corticosteroids shift the Toll-like receptor response pattern of primary-isolated murine liver cells from an inflammatory to an anti-inflammatory state. Int. Immunol. *23*, 537–544. https://doi.org/10.1093/intimm/dxr048.

48. Chowdary, P., Shapiro, S., Makris, M., Evans, G., Boyce, S., Talks, K., Dolan, G., Reiss, U., Phillips, M., Riddell, A., et al. (2022). Phase 1–2 trial of AAVS3 gene therapy in patients with hemophilia B. N. Engl. J. Med. *387*, 237–247. https://doi.org/10.1056/nejmoa2119913.

49. Gougeon, M.L., Poirier-Beaudouin, B., Ausseil, J., Zérah, M., Artaud, C., Heard, J.M., Deiva, K., and Tardieu, M. (2021). Cell-Mediated Immunity to NAGLU Transgene Following Intracerebral Gene Therapy in Children With Mucopolysaccharidosis Type IIIB Syndrome. Front. Immunol. *12*, 655478. https://doi.org/10.3389/fimmu.2021.655478.

50. Deiva, K., Ausseil, J., de Bournonville, S., Zérah, M., Husson, B., Gougeon, M.-L., Poirier-Beaudouin, B., Zafeiriou, D., Parenti, G., Heard, J.M., and Tardieu, M. (2021). Intracerebral Gene therapy in four children with sanfilippo B syndrome: 5.5-year follow up results. Hum. Gene Ther. *32*, 1251–1259. https://doi.org/10.1089/hum.2021.135.

51. Kraaijeveld, R., Li, Y., Yan, L., de Leur, K., Dieterich, M., Peeters, A.M.A., Wang, L., Shi, Y., and Baan, C.C. (2019). Inhibition of T Helper Cell Differentiation by Tacrolimus or Sirolimus Results in Reduced B-Cell Activation: Effects on T Follicular Helper Cells. Transpl. Proc. *51*, 3463–3473. https://doi.org/10.1016/j.transproceed.2019.08.039.

52. Prasad, S., Dimmock, D.P., Greenberg, B., Walia, J.S., Sadhu, C., Tavakkoli, F., and Lipshutz, G.S. (2022). Immune Responses and Immunosuppressive Strategies for Adeno-Associated Virus-Based Gene Therapy for Treatment of Central Nervous System Disorders: Current Knowledge and Approaches. Hum. Gene Ther. *33*, 1228–1245. https://doi.org/10.1089/hum.2022.138.

53. Salabarria, S.M., Nair, J., Clement, N., Smith, B.K., Raben, N., Fuller, D.D., Byrne, B.J., and Corti, M. (2020). Advancements in AAV-mediated Gene Therapy for Pompe Disease. J. Neuromuscul. Dis. *7*, 15–31. https://doi.org/10.3233/JND-190426.

54. Mirea, A., Shelby, E.S., Axente, M., Badina, M., Padure, L., Leanca, M., Dima, V., and Sporea, C. (2021). Combination Therapy with Nusinersen and Onasemnogene Abeparvovec-xioi in Spinal Muscular Atrophy Type I. J. Clin. Med. *10*, 5540. https://doi.org/10.3390/jcm10235540.

55. Kiraly, P., Cottriall, C.L., Taylor, L.J., Jolly, J.K., Cehajic-Kapetanovic, J., Yusuf, I.H., Martinez-Fernandez de la Camara, C., Shanks, M., Downes, S.M., MacLaren, R.E., and Fischer, M.D. (2023). Outcomes and Adverse Effects of Voretigene Neparvovec Treatment for Biallelic *RPE65*-Mediated Inherited Retinal Dystrophies in a Cohort of Patients from a Single Center. Biomolecules *13*, 1484. https://doi.org/10.3390/biom13101484.

56. Shieh, P.B., Kuntz, N.L., Dowling, J.J., Müller-Felber, W., Bönnemann, C.G., Seferian, A.M., Servais, L., Smith, B.K., Muntoni, F., Blaschek, A., et al. (2023). Safety and efficacy of gene replacement therapy for X-linked myotubular myopathy (ASPIRO): a multinational, open-label, dose-escalation trial. Lancet Neurol. *22*, 1125–1139. https://doi.org/10.1016/S1474-4422(23)00313-7.

57. Mendell, J.R., Shieh, P.B., McDonald, C.M., Sahenk, Z., Lehman, K.J., Lowes, L.P., Reash, N.F., Iammarino, M.A., Alfano, L.N., Sabo, B., et al. (2023). Expression of SRP-9001 dystrophin and stabilization of motor function up to 2 years post-treatment with delandistrogene moxeparvovec gene therapy in individuals with Duchenne muscular dystrophy. Front. Cell Dev. Biol. *11*, 1167762. https://doi.org/10.3389/fcell.2023.1167762.

58. Tardieu, M., Zérah, M., Gougeon, M.L., Ausseil, J., de Bournonville, S., Husson, B., Zafeiriou, D., Parenti, G., Bourget, P., Poirier, B., et al. (2017). Intracerebral gene therapy in children with mucopolysaccharidosis type IIIB syndrome: an uncontrolled phase 1/2 clinical trial. Lancet Neurol. *16*, 712–720. https://doi.org/10.1016/S1474-4422(17)30169-2.

59. Rangarajan, S., Walsh, L., Lester, W., Perry, D., Madan, B., Laffan, M., Yu, H., Vettermann, C., Pierce, G.F., Wong, W.Y., and Pasi, K.J. (2017). AAV5–factor VIII gene transfer in severe hemophilia a. N. Engl. J. Med. *377*, 2519–2530. https://doi.org/10.1056/nejmoa1708483.

60. Cehajic-Kapetanovic, J., Xue, K., Martinez-Fernandez de la Camara, C., Nanda, A., Davies, A., Wood, L.J., Salvetti, A.P., Fischer, M.D., Aylward, J.W., Barnard, A.R., et al. (2020). Initial results from a first-in-human gene therapy trial on X-linked retinitis pigmentosa caused by mutations in RPGR. Nat. Med. *26*, 354–359. https://doi.org/10.1038/s41591-020-0763-1.

61. Verdera, H.C., Kuranda, K., and Mingozzi, F. (2020). AAV vector immunogenicity in humans: A long journey to successful Gene Transfer. Mol. Ther. *28*, 723–746. https://doi.org/10.1016/j.ymthe.2019.12.010.

62. Bönnemann, C.G., Belluscio, B.A., Braun, S., Morris, C., Singh, T., and Muntoni, F. (2023). Dystrophin Immunity after Gene Therapy for Duchenne's Muscular Dystrophy. N. Engl. J. Med. *388*, 2294–2296. https://doi.org/10.1056/NEJMc2212912.

63. Xue, F., Li, H., Wu, X., Liu, W., Zhang, F., Tang, D., Chen, Y., Wang, W., Chi, Y., Zheng, J., et al. (2022). Safety and activity of an engineered, liver-tropic adeno-associated virus vector expressing a hyperactive Padua factor ix administered with prophylactic glucocorticoids in patients with haemophilia B: A single-centre, single-arm, phase 1, pilot trial. Lancet Haematol. *9*, e504–e513. https://doi.org/10.1016/s2352-3026(22)00113-2.

64. Flanigan, K.M., Vetter, T.A., Simmons, T.R., Iammarino, M., Frair, E.C., Rinaldi, F., Chicoine, L.G., Harris, J., Cheatham, J.P., Cheatham, S.L., et al. (2022). A first-in-human phase I/IIA gene transfer clinical trial for Duchenne muscular dystrophy using rAAVrh74.MCK.GALGT2. Mol. Ther. Methods Clin. Dev. *27*, 47–60. https://doi.org/10.1016/j.omtm.2022.08.009.

65. Friese, J., Geitmann, S., Holzwarth, D., Müller, N., Sassen, R., Baur, U., Adler, K., and Kirschner, J. (2021). Safety Monitoring of Gene Therapy for Spinal Muscular Atrophy with Onasemnogene Abeparvovec -A Single Centre Experience. J. Neuromuscul. Dis. *8*, 209–216. https://doi.org/10.3233/JND-200593.

66. Mendell, J.R., Sahenk, Z., Lehman, K., Nease, C., Lowes, L.P., Miller, N.F., Iammarino, M.A., Alfano, L.N., Nicholl, A., Al-Zaidy, S., et al. (2020). Assessment of systemic delivery of Raavrh74.mhck7.micro-dystrophin in children with Duchenne muscular dystrophy. JAMA Neurol. *77*, 1122–1131. https://doi.org/10.1001/jamaneurol.2020.1484.

67. Long, B.R., Veron, P., Kuranda, K., Hardet, R., Mitchell, N., Hayes, G.M., Wong, W.Y., Lau, K., Li, M., Hock, M.B., et al. (2021). Early phase clinical immunogenicity of Valoctocogene Roxaparvovec, an AAV5-mediated gene therapy for hemophilia A. Mol. Ther. *29*, 597–610. https://doi.org/10.1016/j.ymthe.2020.12.008.

68. Majowicz, A., Nijmeijer, B., Lampen, M.H., Spronck, L., de Haan, M., Petry, H., van Deventer, S.J., Meyer, C., Tangelder, M., and Ferreira, V. (2019). Therapeutic hfix activity achieved after single AAV5-hfix treatment in hemophilia B patients and nhps with pre-existing Anti-AAV5 Nabs. Mol. Ther. Methods Clin. Dev. *14*, 27–36. https://doi.org/10.1016/j.omtm.2019.05.009.

69. Hauswirth, W.W., Aleman, T.S., Kaushal, S., Cideciyan, A.V., Schwartz, S.B., Wang, L., Conlon, T.J., Boye, S.L., Flotte, T.R., Byrne, B.J., and Jacobson, S.G. (2008). Treatment of leber congenital amaurosis due to*rpe65*mutations by ocular subretinal injection of adeno-associated virus gene vector: Short-term results of a phase I trial. Hum. Gene Ther. *19*, 979–990. https://doi.org/10.1089/hum.2008.107.

70. Finkel, R.S., Darras, B.T., JR., Mendell, J.R., Day, J.W., Kuntz, N.L., Connolly, A.M., Zaidman, C.M., Crawford, T.O., Butterfield, R.J., Shieh, P.B., et al. (2023). Intrathecal Onasemnogene Abeparvovec for sitting, nonambulatory patients with spinal muscular atrophy: Phase I ascending-dose study (strong). J. Neuromuscul. Dis. *10*, 389–404. https://doi.org/10.3233/jnd-221560.

71. Mishra, A., Vijayasarathy, C., Cukras, C.A., Wiley, H.E., Sen, H.N., Zeng, Y., Wei, L.L., and Sieving, P.A. (2021). Immune function in X-linked retinoschisis subjects in an AAV8-RS1 phase I/IIA gene therapy trial. Mol. Ther. *29*, 2030–2040. https://doi.org/10.1016/j.ymthe.2021.02.013.

72. Mueller, C., Chulay, J.D., Trapnell, B.C., Humphries, M., Carey, B., Sandhaus, R.A., McElvaney, N.G., Messina, L., Tang, Q., Rouhani, F.N., et al. (2013). Human Treg responses allow sustained recombinant adeno-associated virus–mediated transgene expression. J. Clin. Invest. *123*, 5310–5318. https://doi.org/10.1172/jci70314.

73. Muhuri, M., Maeda, Y., Ma, H., Ram, S., Fitzgerald, K.A., Tai, P.W., and Gao, G. (2021). Overcoming innate immune barriers that impede AAV gene therapy vectors. J. Clin. Invest. *131*, e143780. https://doi.org/10.1172/jci143780.

74. MacLaren, R.E., Groppe, M., Barnard, A.R., Cottriall, C.L., Tolmachova, T., Seymour, L., Clark, K.R., During, M.J., Cremers, F.P.M., Black, G.C.M., et al. (2014). Retinal gene therapy in patients with CHOROIDEREMIA: Initial findings from a phase 1/2 clinical trial. The Lancet *383*, 1129–1137. https://doi.org/10.1016/s0140 6736(13)62117-0.

75. Perocheau, D.P., Cunningham, S., Lee, J., Antinao Diaz, J., Waddington, S.N., Gilmour, K., Eaglestone, S., Lisowski, L., Thrasher, A.J., Alexander, I.E., et al. (2019). Age-related seroprevalence of antibodies against AAV-LK03 in a UK population cohort. Hum. Gene Ther. *30*, 79–87. https://doi.org/10.1089/hum.2018.098.

76. Wang, L., Calcedo, R., Wang, H., Bell, P., Grant, R., Vandenberghe, L.H., Sanmiguel, J., Morizono, H., Batshaw, M.L., and Wilson, J.M. (2010). The pleiotropic effects of

natural AAV infections on liver-directed gene transfer in macaques. Mol. Ther. *18*, 126–134. https://doi.org/10.1038/mt.2009.245.

77. Annex I summary of Product Characteristics - European Medicines Agency. https://www.ema.europa.eu/en/documents/product-information/roctavian-epar-product-information_en.pdf. accessed date August 02, 2024.

78. Guideline on the Quality, Non-clinical and Clinical Aspects of Gene Therapy Medicinal Products. (EMA/CAT/80183/2014). European Medicines Agency. https://www.ema.europa.eu/en/documents/scientific-guideline/guideline-quality-non-clinical-aspects-gene-therapy-medicinal-products_en.pdf. accessed date August 02, 2024.

79. Center for Drug Evaluation and Research (no date) Guidance for Industry, U.S. Food and Drug Administration. https://www.fda.gov/regulatory-information/search-fda-guidance-documents/immunogenicity-testing-therapeutic-protein-products-developing-and-validating-assays-anti-drug. accessed date August 02, 2024.

80. Mendell, J.R., Sahenk, Z., and Rodino-Klapac, L.R. (2019). Duchenne muscular dystrophy exon-skipping trials. Muscle Gene Ther. 727–743. https://doi.org/10.1007/978-3-030-03095-7_42.

81. Fong, S., Yates, B., Sihn, C.-R., Mattis, A.N., Mitchell, N., Liu, S., Russell, C.B., Kim, B., Lawal, A., Rangarajan, S., et al. (2022). Interindividual variability in transgene mrna and protein production following adeno-associated virus gene therapy for hemophilia A. Nat. Med. *28*, 789–797. https://doi.org/10.1038/s41591-022-01751-0.

82. Konkle, B.A., Walsh, C.E., Escobar, M.A., Josephson, N.C., Young, G., von Drygalski, A., McPhee, S.W.J., Samulski, R.J., Bilic, I., de la Rosa, M., et al. (2021). BAX 335 hemophilia B gene therapy clinical trial results: Potential impact of CPG sequences on gene expression. Blood *137*, 763–774. https://doi.org/10.1182/blood.2019004625.

83. Weiß, C., Ziegler, A., Becker, L.L., Johannsen, J., Brennenstuhl, H., Schreiber, G., Flotats-Bastardas, M., Stoltenburg, C., Hartmann, H., Illsinger, S., et al. (2022). Gene replacement therapy with onasemnogene abeparvovec in children with spinal muscular atrophy aged 24 months or younger and bodyweight up to 15 kg: an observational cohort study. Lancet Child Adolesc. Health *6*, 17–27. https://doi.org/10.1016/S2352-4642(21)00287-X.

84. Rakoczy, E.P., Lai, C.-M., Magno, A.L., Wikstrom, M.E., French, M.A., Pierce, C.M., Schwartz, S.D., Blumenkranz, M.S., Chalberg, T.W., Degli-Esposti, M.A., and Constable, I.J. (2015). Gene therapy with recombinant adeno-associated vectors for neovascular age-related macular degeneration: 1 Year follow-up of a phase 1 randomised clinical trial. Lancet *386*, 2395–2403. https://doi.org/10.1016/s0140-6736(15)00345-1.

85. Pasi, K.J., Laffan, M., Rangarajan, S., Robinson, T.M., Mitchell, N., Lester, W., Symington, E., Madan, B., Yang, X., Kim, B., et al. (2021). Persistence of haemostatic response following gene therapy with Valoctocogene Roxaparvovec in severe haemophilia a. Haemophilia *27*, 947–956. https://doi.org/10.1111/hae.14391.

86. Miesbach, W., Meijer, K., Coppens, M., Kampmann, P., Klamroth, R., Schutgens, R., Tangelder, M., Castaman, G., Schwäble, J., Bonig, H., et al. (2018). Gene therapy with adeno-associated virus vector 5-human factor IX in adults with hemophilia B. Blood *131*, 1022–1031. https://doi.org/10.1182/blood-2017-09-804419.

87. Chandra, S., Long, B.R., Fonck, C., Melton, A.C., Arens, J., Woloszynek, J., and O'Neill, C.A. (2023). Safety findings of dosing gene therapy vectors in NHP with pre-existing or treatment-emergent anti-capsid antibodies. Toxicol. Pathol. *51*, 246–256. https://doi.org/10.1177/01926233231202995.

88. Campbell, E.S.B., Goens, M.M., Cao, W., Thompson, B., Susta, L., Banadyga, L., and Wootton, S.K. (2023). Recent advancements in aav-vectored immunoprophylaxis in the nonhuman primate model. Biomedicines *11*, 2223. https://doi.org/10.3390/biomedicines11082223.

89. Meliani, A., Boisgerault, F., Hardet, R., Marmier, S., Collaud, F., Ronzitti, G., Leborgne, C., Costa Verdera, H., Simon Sola, M., Charles, S., et al. (2018). Antigen-selective modulation of AAV immunogenicity with tolerogenic rapamycin nanoparticles enables successful vector re-administration. Nat. Commun. *9*, 4098. https://doi.org/10.1038/s41467-018-06621-3.

90. Rhen, T., and Cidlowski, J.A. (2005). Anti-inflammatory action of glucocorticoids — new mechanisms for old drugs. N. Engl. J. Med. *353*, 1711–1723. https://doi.org/10.1056/nejmra050541.

91. Youssef, J., Novosad, S.A., and Winthrop, K.L. (2016). Infection risk and safety of corticosteroid use. Rheum. Dis. Clin. North Am. *42*, 157–176. https://doi.org/10.1016/j.rdc.2015.08.004.

92. Li, X., Wei, X., Lin, J., and Ou, L. (2022). A versatile toolkit for overcoming AAV immunity. Front. Immunol. *13*, 991832. https://doi.org/10.3389/fimmu.2022.991832.

93. Oh, G.J., Waldo, A., Paez-Cruz, F., Gipson, P.E., Pesenson, A., Selewski, D.T., Kamil, E.S., Massengill, S.F., Lafayette, R.A., Modes, M., et al. (2019). Steroid-Associated Side Effects in Patients With Primary Proteinuric Kidney Disease. Kidney Int. Rep. *4*, 1608–1616. https://doi.org/10.1016/j.ekir.2019.08.019.

94. Movahedi, M., Beauchamp, M.E., Abrahamowicz, M., Ray, D.W., Michaud, K., Pedro, S., and Dixon, W.G. (2016). Risk of Incident Diabetes Mellitus Associated With the Dosage and Duration of Oral Glucocorticoid Therapy in Patients With Rheumatoid Arthritis. Arthritis Rheumatol. *68*, 1089–1098.

95. Uzu, T., Harada, T., Sakaguchi, M., Kanasaki, M., Isshiki, K., Araki, S., Sugiomoto, T., Koya, D., Haneda, M., Kashiwagi, A., and Yamauchi, A. (2007). Glucocorticoid-induced diabetes mellitus: prevalence and risk factors in primary renal diseases. Nephron. Clin. Pract. *105*, c54–c57.

96. Smith, C.J., Ross, N., Kamal, A., Kim, K.Y., Kropf, E., Deschatelets, P., Francois, C., Quinn, W.J., 3rd, Singh, I., Majowicz, A., et al. (2022). Pre-existing humoral immunity and complement pathway contribute to immunogenicity of adeno-associated virus (AAV) vector in human blood. Front. Immunol. *13*, 999021. https://doi.org/10.3389/fimmu.2022.999021.

97. Bertolini, T.B., Shirley, J.L., Zolotukhin, I., Li, X., Kaisho, T., Xiao, W., Kumar, S.R.P., and Herzog, R.W. (2021). Effect of CPG depletion of Vector Genome on CD8+ T cell responses in Aav Gene therapy. Front. Immunol. *12*, 672449. https://doi.org/10.3389/fimmu.2021.672449.

98. Doshi, B.S., Raffini, L.J., and George, L.A. (2020). Combined anti-CD20 and mTOR inhibition with factor VIII for immune tolerance induction in hemophilia A patients with refractory inhibitors. J. Thromb. Haemost. *18*, 848–852. https://doi.org/10.1111/jth.14740.

99. Biswas, M., Palaschak, B., Kumar, S.R.P., Rana, J., and Markusic, D.M. (2020). B cell depletion eliminates FVIII memory B cells and enhances AAV8-Cof8 immune tolerance induction when combined with rapamycin. Front. Immunol. *11*, 1293. https://doi.org/10.3389/fimmu.2020.01293.

100. Byrne, B.J., Fuller, D.D., Smith, B.K., Clement, N., Coleman, K., Cleaver, B., Vaught, L., Falk, D.J., McCall, A., and Corti, M. (2019). Pompe disease gene therapy: Neural manifestations require consideration of CNS directed therapy. Ann. Transl. Med. *7*, 290. https://doi.org/10.21037/atm.2019.05.56.

101. Lefebvre, C., Glanville, J., Briscoe, S., Littlewood, A., and Marshall, C. (2019). Technical Supplement to Chapter 4: Searching for and selecting studies. In Cochrane Handbook for Systematic Reviews of Interventions Version 6, J.P.T. Higgins, J. Thomas, J. Chandler, M.S. Cumpston, T. Li, M.J. Page, and V.A. Welch, eds. (Cochrane), www.training.cochrane.org/handbook.

102. Bowles, D.E., McPhee, S.W.J., Li, C., Gray, S.J., Samulski, J.J., Camp, A.S., Li, J., Wang, B., Monahan, P.E., Rabinowitz, J.E., et al. (2012). Phase 1 gene therapy for Duchenne muscular dystrophy using a translational optimized AAV vector. Mol. Ther. *20*, 443–455. https://doi.org/10.1038/mt.2011.237.

103. Zaidman, C.M., Proud, C.M., McDonald, C.M., Lehman, K.J., Goedeker, N.L., Mason, S., Murphy, A.P., Guridi, M., Wang, S., Reid, C., et al. (2023). Delandistrogene moxeparvovec gene therapy in ambulatory patients (aged ≥4 to <8 years) with Duchenne muscular dystrophy: 1-year interim results from study SRP-9001-103 (ENDEAVOR). Ann. Neurol. *94*, 955–968.

104. Leavitt, A.D., Konkle, B.A., Stine, K.C., Visweshwar, N., Harrington, T.J., Giermasz, A., Arkin, S., Fang, A., Plonski, F., Yver, A., et al. (2024). Giroctocogene fitelparvovec gene therapy for severe hemophilia A: 104-week analysis of the phase 1/2 Alta study. Blood *143*, 796–806. https://doi.org/10.1182/blood.2022018971.

105. George, L.A., Sullivan, S.K., Giermasz, A., Rasko, J.E.J., Samelson-Jones, B.J., Ducore, J., Cuker, A., Sullivan, L.M., Majumdar, S., Teitel, J., et al. (2017). Hemophilia B Gene Therapy with a High-Specific-Activity Factor IX Variant. N. Engl. J. Med. *377*, 2215–2227. https://doi.org/10.1056/NEJMoa1708538.

106. Von Drygalski, A., Giermasz, A., Castaman, G., Key, N.S., Lattimore, S., Leebeek, F.W.G., Miesbach, W., Recht, M., Long, A., Gut, R., et al. (2019). Etranacogene dezaparvovec (AMT-061 phase 2b): normal/near normal FIX activity and bleed cessation in hemophilia B. Blood Adv. *3*, 3241–3247. Erratum in: Blood Adv. 2020 Aug 11;4, :3668. doi: 10.1182/bloodadvances.2020002987. https://doi.org/10.1182/bloodadvances.2019000811.

107. Lam, B.L., Feuer, W.J., Davis, J.L., Porciatti, V., Yu, H., Levy, R.B., Vanner, E., and Guy, J. (2022). Leber Hereditary Optic Neuropathy Gene Therapy: Adverse Events and Visual Acuity Results of All Patient Groups. Am. J. Ophthalmol. *241*, 262–271. https://doi.org/10.1016/j.ajo.2022.02.023.

108. Weleber, R.G., Pennesi, M.E., Wilson, D.J., Kaushal, S., Erker, L.R., Jensen, L., McBride, M.T., Flotte, T.R., Humphries, M., Calcedo, R., et al. (2016). Results at 2 Years after Gene Therapy for RPE65-Deficient Leber Congenital Amaurosis and Severe Early-Childhood-Onset Retinal Dystrophy. Ophthalmology *123*, 1606–1620. https://doi.org/10.1016/j.ophtha.2016.03.003.

109. D'Silva, A.M., Holland, S., Kariyawasam, D., Herbert, K., Barclay, P., Cairns, A., MacLennan, S.C., Ryan, M.M., Sampaio, H., Smith, N., et al. (2022). Onasemnogene abeparvovec in spinal muscular atrophy: an Australian experience of safety and efficacy. Ann. Clin. Transl. Neurol. *9*, 339–350. https://doi.org/10.1002/acn3.51519.

110. von Krusenstiern, L., Liu, J., Liao, E., Gow, J.A., Chen, G., Ong, T., Lotery, A.J., Jalil, A., Lam, B.L., and MacLaren, R.E.; XIRIUS Part 1 Study GroupXOLARIS Study Group (2023 Mar 1). Changes in Retinal Sensitivity Associated With Cotoretigene Toliparvovec in X-Linked Retinitis Pigmentosa With RPGR Gene Variations. JAMA Ophthalmol. *141*, 275–283. Erratum in: JAMA Ophthalmol. 2023;141:293. doi: 10.1001/jamaophthalmol.2023.0716. https://doi.org/10.1001/jamaophthalmol.2022.6254.

111. Gowda, V., Atherton, M., Murugan, A., Servais, L., Sheehan, J., Standing, E., Manzur, A., Scoto, M., Baranello, G., Munot, P., et al. (2024). Efficacy and safety of onasemnogene abeparvovec in children with spinal muscular atrophy type 1: real-world evidence from 6 infusion centres in the United Kingdom. Lancet Reg. Health Eur. *37*, 100817. https://doi.org/10.1016/j.lanepe.2023.100817.

112. Servais, L., Day, J.W., De Vivo, D.C., Kirschner, J., Mercuri, E., Muntoni, F., Proud, C.M., Shieh, P.B., Tizzano, E.F., Quijano-Roy, S., et al. (2024). Real-World Outcomes in Patients with Spinal Muscular Atrophy Treated with Onasemnogene Abeparvovec Monotherapy: Findings from the RESTORE Registry. J. Neuromuscul. Dis. *11*, 425–442. https://doi.org/10.3233/JND-230122.

113. Bitetti, I., Lanzara, V., Margiotta, G., and Varone, A. (2023). Onasemnogene abeparvovec gene replacement therapy for the treatment of spinal muscular atrophy: a real-world observational study. Gene Ther. *30*, 592–597. https://doi.org/10.1038/s41434-022-00341-6.

114. Nanri, D., Yuge, K., Goto, K., Kimura, T., Yae, Y., Mizuochi, T., Sato, R., Itonaga, T., Maeda, T., and Yamashita, Y. (2024). Onasemnogene Abeparvovec Treatment after Nusinersen in an Infant with Spinal Muscular Atrophy Type 1. Kurume Med. J. *69*, 255–259. https://doi.org/10.2739/kurumemedj.MS6934008.

115. Witte, D., Hartmann, H., Drube, J., Haffner, D., and Illsinger, S. (2022). Thrombotische Mikroangiopathie (TMA) nach Genersatztherapie (GRT) bei spinaler Muskelatrophie: Ein Fallbericht und Therapieoption [Thrombotic Microangiopathy (TMA) after Gene Replacemant Therapy (GRT) due to Spinal Muscular Atrophy: Case Summary and Recommendations for Treatment]. Klin. Padiatr. *234*, 42–47. German. https://doi.org/10.1055/a-1538-4936.

116. Eisenkölbl, A., and Pühringer, M. (2024). Repeated AAV9 Titer Determination in a Presymptomatic SMA Patient with Three SMN2 Gene Copies - A Case Report. J. Neuromuscul. Dis. https://doi.org/10.3233/JND-221659.

117. Favia, M., Tarantino, D., Cerbo, L.D., Sabia, A., Campopiano, R., and Pani, M. (2024). Onasemnogene Abeparvovec: Post-infusion Efficacy and Safety in Patients With Spinal Muscular Atrophy (SMA)—A Fondazione Policlinico Gemelli IRCCS Experience. Hosp. Pharm. *59*, 39–46.

118. Waldrop, M.A., Chagat, S., Storey, M., Meyer, A., Iammarino, M., Reash, N., Alfano, L., Lowes, L., Noritz, G., Prochoroff, A., et al. (2024). Continued safety and long-term effectiveness of onasemnogene abeparvovec in Ohio. Neuromuscul. Disord. *34*, 41–48. https://doi.org/10.1016/j.nmd.2023.11.010.

119. Waldrop, M.A., Karingada, C., Storey, M.A., Powers, B., Iammarino, M.A., Miller, N.F., Alfano, L.N., Noritz, G., Rossman, I., Ginsberg, M., et al. (2020). Gene Therapy for Spinal Muscular Atrophy: Safety and Early Outcomes. Pediatrics *146*, e20200729. https://doi.org/10.1542/peds.2020-0729.

120. Chiang, J., Xiao, L., Nigro, E., St-Laurent, A., Weinstock, L., Law, E., Janevski, J., Kuyntjes, S., Cithiravel, N., Tran, T., et al. (2023). Sleep disordered breathing in infants identified through newborn screening with spinal muscular atrophy. Sleep Med. *111*, 161–169. https://doi.org/10.1016/j.sleep.2023.09.019.

121. Beri, N., Kapoor, L., Parashar, D., and Mundada, V. (2023). Effect on maximal mouth opening in children with spinal muscular atrophy treated with onasemnogene abeparvovec. Arch. Dis. Child. *108*, 866–867. https://doi.org/10.1136/archdischild-2023-325788.

122. Chencheri, N., Alexander, G., Nugud, A., Majadas, E., Salim, H., Prudhomme, K., DeJager, N., Janardhanan, V.S., and Elbashir, H. (2023). Gene transfer therapy in children with spinal muscular atrophy: A single-center experience with a cohort of 25 children. Muscle Nerve *68*, 269–277. https://doi.org/10.1002/mus.27926.

123. Soini, V., Schreiber, G., Wilken, B., and Hell, A.K. (2023). Early Development of Spinal Deformities in Children Severely Affected with Spinal Muscular Atrophy after Gene Therapy with Onasemnogene Abeparvovec-Preliminary Results. Children (Basel) *10*, 998. https://doi.org/10.3390/children10060998.

124. Pane, M., Coratti, G., Sansone, V.A., Messina, S., Catteruccia, M., Bruno, C., Sframeli, M., Albamonte, E., Pedemonte, M., Brolatti, N., et al. (2023). Italian EAP Working Group. Type I spinal muscular atrophy patients treated with nusinersen: 4-year follow-up of motor, respiratory and bulbar function. Eur. J. Neurol. *30*, 1755–1763. https://doi.org/10.1111/ene.15768.

125. Gaillard, J., Gu, A.R., and Neil Knierbein, E.E. (2023). Necrotizing Enterocolitis following Onasemnogene Abeparvovec for Spinal Muscular Atrophy: A Case Series. J. Pediatr. *260*, 113493. https://doi.org/10.1016/j.jpeds.2023.113493.

126. Tokatly Latzer, I., Sagi, L., Lavi, R., Aharoni, S., Bistritzer, J., Noyman, I., Ginsburg, M., Lev-Or, A., Katzenellenbogen, S., Nevo, Y., and Fattal-Valevski, A. (2023). Real-Life Outcome After Gene Replacement Therapy for Spinal Muscular Atrophy: A Multicenter Experience. Pediatr. Neurol. *144*, 60–68. https://doi.org/10.1016/j.pediatrneurol.2023.04.007.

127. Sawada, T., Kido, J., Sugawara, K., Yoshida, S., Ozasa, S., Nomura, K., Okada, K., Fujiyama, N., and Nakamura, K. (2022). Newborn screening for spinal muscular atrophy in Japan: One year of experience. Mol. Genet. Metab. Rep. *32*, 100908. https://doi.org/10.1016/j.ymgmr.2022.100908.

128. Stettner, G.M., Hasselmann, O., Tscherter, A., Galiart, E., Jacquier, D., and Klein, A. (2023). Treatment of spinal muscular atrophy with Onasemnogene Abeparvovec in Switzerland: a prospective observational case series study. BMC Neurol. *23*, 88. https://doi.org/10.1186/s12883-023-03133-6.

129. Tosi, M., Catteruccia, M., Cherchi, C., Mizzoni, I., and D'Amico, A. (2022). Switching therapies: safety profile of Onasemnogene abeparvovec-xioi in a SMA1 patient previously treated with Risdiplam. Acta Myol. *41*, 117–120. https://doi.org/10.36185/2532-1900-077.

130. Lee, S., Lee, Y.J., Kong, J., Ryu, H.W., Shim, Y.K., Han, J.Y., Woo, H., Kim, S.Y., Cho, A., Lim, B.C., and Chae, J.H. (2022). Short-term clinical outcomes of onasemnogene abeparvovec treatment for spinal muscular atrophy. Brain Dev. *44*, 287–293. https://doi.org/10.1016/j.braindev.2021.12.006.

131. Matesanz, S.E., Battista, V., Flickinger, J., Jones, J.N., and Kichula, E.A. (2021). Clinical Experience With Gene Therapy in Older Patients With Spinal Muscular Atrophy. Pediatr. Neurol. *118*, 1–5. https://doi.org/10.1016/j.pediatrneurol.2021.01.012.

132. Ali, H.G., Ibrahim, K., Elsaid, M.F., Mohamed, R.B., Abeidah, M.I.A., Al Rawwas, A.O., Elshafey, K., Almulla, H., El-Akouri, K., Almulla, M., et al. (2021). Gene therapy for spinal muscular atrophy: the Qatari experience. Gene Ther. *28*, 676–680. https://doi.org/10.1038/s41434-021-00273-7.

133. Nevmerzhitskaya, K.S., Sapego, E.Y., and Morozova, D.A. (2021). Short-term safety and efficacy of Onasemnogene Abeparvovec in 10 patients with Spinal Muscular Atrophy: Cohort study. Vopr. Sovr. Pediatr. *20*, 589–594. https://doi.org/10.15690/vsp.v20i6s.2367.

134. Nigro, E., Grunebaum, E., Kamath, B., Licht, C., Malcolmson, C., Jeewa, A., Campbell, C., McMillan, H., Chakraborty, P., Tarnopolsky, M., and Gonorazky, H. (2023). Case report: A case of spinal muscular atrophy in a preterm infant: risks

and benefits of treatment. Front. Neurol. *14*, 1230889. https://doi.org/10.3389/fneur.2023.1230889.

135. Lopez, J., Borchert, M., Lee, T.C., and Nagiel, A. (2023). Subretinal deposits in young patients treated with voretigene neparvovec-rzyl for RPE65-mediated retinal dystrophy. Br. J. Ophthalmol. *107*, 299–301. https://doi.org/10.1136/bjo-2022-321488.

136. Jalil, A., Ivanova, T., Moussa, G., Parry, N.R.A., and Black, G.C.M. (2023). Retinal gene therapy in RPE-65 gene mediated inherited retinal dystrophy. Eye *37*, 1874–1877. https://doi.org/10.1038/s41433-022-02262-5.

137. Ozelo, M.C., Mahlangu, J., Pasi, K.J., Giermasz, A., Leavitt, A.D., Laffan, M., Symington, E., Quon, D.V., Wang, J.D., Peerlinck, K., et al. (2022). GENEr8-1 Trial Group. Valoctocogene Roxaparvovec Gene Therapy for Hemophilia A. N. Engl. J. Med. *386*, 1013–1025. https://doi.org/10.1056/NEJMoa2113708.

138. Page, M.J., McKenzie, J.E., Bossuyt, P.M., Boutron, I., Hoffmann, T.C., Mulrow, C.D., Shamseer, L., Tetzlaff, J.M., Akl, E.A., Brennan, S.E., et al. (2021). The PRISMA 2020 statement: an updated guideline for reporting systematic reviews. BMJ *372*, n71. https://doi.org/10.1136/bmj.n71.

YMTHE, Volume *32*

## Supplemental Information

# A systematic review of immunosuppressive protocols used in AAV gene therapy for monogenic disorders

**Besarte Vrellaku, Ilda Sethw Hassan, Rebecca Howitt, Christopher P. Webster, Eli Harriss, Fraser McBlane, Corinne Betts, Jorge Schettini, Mattia Lion, John E. Mindur, Michael Duerr, Pamela J. Shaw, Janine Kirby, Mimoun Azzouz, and Laurent Servais**

Table S1: Search Results for immunogenicity and immune monitoring from clinical trials.

| | Search results on 15/11/2021 | Search results on 29/03/2023 | Search results on 05/03/2024 |
|---|---|---|---|
| Ovid Embase | 1837 | 2243 | 2586 |
| Ovid Medline | 406 | 480 | 536 |
| Cochrane CENTRAL | 48 | 59 | 728 |
| Total | 2291 | 2782 | 3850 |
| Total after deduplication | 1923 | 2302 | - |
| Unique since 15/11/2021 | - | 396 | - |
| Unique since 29/03/2023 | - | - | **952** |

**Database: Embase 1974 to present**

**Search Strategy:**

**1** exp gene therapy/ (102165)

**2** targeted gene repair/ (445)

**3** ("DNA therap*" or (gene* adj2 therap*) or "gene* transfer*" or transgene or "gene replacement*" or "gene-edit*" or "gene silencing" or "gene repair*" or "gene correction*").ti,ab. (212907)

**4** 1 or 2 or 3 (251960)

**5** (Randomized controlled trial/ or Controlled clinical study/ or random$.ti,ab. or randomization/ or intermethod comparison/ or placebo.ti,ab. or (compare or compared or comparison).ti. or ((evaluated or evaluate or evaluating or assessed or assess) and (compare or compared or comparing or comparison)).ab. or (open adj label).ti,ab. or ((double or single or doubly or singly) adj (blind or blinded or blindly)).ti,ab. or double blind procedure/ or parallel group$1.ti,ab. or (crossover or cross over).ti,ab. or ((assign$ or match or matched or allocation) adj5 (alternate or group$1 or intervention$1 or patient$1 or subject$1 or participant$1)).ti,ab. or (assigned or allocated).ti,ab. or (controlled adj7 (study or design or trial)).ti,ab. or (volunteer or volunteers).ti,ab. or human experiment/ or trial.ti.) not (((random$ adj sampl$ adj7 ("cross section$" or questionnaire$1 or survey$ or database$1)).ti,ab. not (comparative study/ or controlled study/ or randomi?ed controlled.ti,ab. or randomly assigned.ti,ab.)) or (Cross-sectional study/ not (randomized controlled trial/ or controlled clinical study/ or controlled study/ or randomi?ed controlled.ti,ab. or control group$1.ti,ab.)) or (((case adj control$) and random$) not randomi?ed controlled).ti,ab. or (Systematic review not (trial or study)).ti. or (nonrandom$ not random$).ti,ab. or "Random field$".ti,ab. or (random cluster adj3 sampl$).ti,ab. or ((review.ab. and review.pt.) not trial.ti.) or ("we searched".ab. and (review.ti. or review.pt.)) or "update review".ab. or (databases adj4 searched).ab.) (6133950)

**6** clinical trial/ or phase 1 clinical trial/ or phase 2 clinical trial/ or phase 3 clinical trial/ (1222173)

**7** ("clinical trial*" or "phase i" or "phase 1" or "phase ii" or "phase 2" or "phase iii" or "phase 3" or "interventional trial*" or "paediatric trial*" or "pediatric trial*" or "pivotal trial*" or "open label*" or "orphan drug*" or "rare disease*").ti,ab. or rare disease/ or orphan drug/ (1137498)

**8** 5 or 6 or 7 (7071304)

**9** epidural drug administration/ or intracisternal drug administration/ or intrathecal drug administration/ (27063)
**10** intraocular drug administration/ or intracorneal drug administration/ or intravitreal drug administration/ or subretinal drug administration/ (10616)
**11** exp intraspinal drug administration/ (29464)
**12** exp intraocular drug administration/ (12131)
**13** drug infusion/ (17165)
**14** exp intracerebral drug administration/ (31720)
**15** intravenous drug administration/ (384430)
**16** (Intravenous* or intra-venous* or IV or systemic or infusion* or intramuscular* or intra-muscular* or IM or intrathecal* or intra-thecal* or "cerebrospinal fluid*" or CSF or intra-CSF or epidural* or spinal* or ophthalmol* or subretinal* or sub-retinal* or intravitreal* or intra-vitreal* or intraorbital* or intra-orbital* or intracerebroventricular* or intra-cerebroventricular* or ICV or "i.c.v. injection*" or ICVI or intracerebral* or intra-cerebral* or IC or intraventricular* or intra-ventricular* or intraparenchyma* or intra-parenchyma* or "intra-cisterna magna" or ICM).ti,ab. (3195173)
**17** 9 or 10 or 11 or 12 or 13 or 14 or 15 or 16 (3407200)
**18** rare disease/ (50265)
**19** orphan drug/ (4013)
**20** exp spinal muscular atrophy/ (71765)
**21** centronuclear myopathy/ (1323)
**22** myotonic dystrophy/ (9744)
**23** exp muscular dystrophy/ (52657)
**24** spastic paraplegia/ (5101)
**25** exp gangliosidosis/ (4390)
**26** multiple sclerosis/ (161878)
**27** parkinson disease/ or autosomal dominant parkinson disease/ (196772)
**28** Alzheimer disease/ (257013)
**29** juvenile neuronal ceroid lipofuscinosis/ (295)
**30** giant axonal neuropathy/ (320)
**31** hereditary motor sensory neuropathy/ (13412)
**32** exp hemophilia/ (46582)
**33** exp sickle cell anemia/ (47775)
**34** glycogen storage disease type 2/ (5209)
**35** Canavan disease/ (818)
**36** Fabry disease/ (9593)
**37** exp mucopolysaccharidosis/ (14039)
**38** familial hypercholesterolemia/ (12739)
**39** exp congestive cardiomyopathy/ (55227)
**40** cystic fibrosis/ (86136)
**41** alpha 1 antitrypsin deficiency/ (6642)
**42** ciliary dyskinesia/ (3703)
**43** exp macular degeneration/ (27716)
**44** Leber congenital amaurosis/ (2306)
**45** exp monogenic disorder/ (573870)
**46** ("rare disease*" or "orphan drug*" or "monogenic disease*" or "monogenic disorder*" or "spinal muscular atrophy" or "spine muscle atrophy" or "myotubular myopathy" or "centronuclear myopathy" or "myotonic dystrophy" or "curschmann steinert disease" or "deleage disease" or "dystrophic myotonica" or "dystrophic myotonia" or "myotonia atrophica" or "myotonia dystrophica" or "myotonic atrophia" or "myotonic atrophy" or

"myotonic dystrophia" or "myotonic muscular dystrophy" or "myotonic muscular dystrophy" or "proximal myotonic myopathy" or "steinert disease" or "steinert myopathy" or "muscular dystrophy" or "muscle dystrophy" or "muscle dystrophia" or "motor neuron* disease" or MND or "Spastic paraplegia" or "spastic paraparesis" or gangliosidosis or "ganglioside lipidosis" or gangliosidoses or "multiple sclerosis" or "chariot disease" or "disseminated sclerosis" or "insular sclerosis" or MS or Parkinson* or "paralysis agitans" or Alzheimer* or Alzeimer* or "diffuse cortical sclerosis" or Batten* or "juvenile neuronal ceroid lipofuscinosis" or "Giant axonal neuropathy" or GAN or GAN-1 or "Amyotrophic lateral sclerosis" or ALS or "lou gehrig*" or Charcot-Marie-Tooth or "hereditary motor sensory neuropathy" or "charcot-marie" or "dejerine sottas" or "familial spastic para*" or "hereditary spastic para*" or Struempell or strumpel* or SCID-X or Haemophili* or hemophili* or "sickle cell" or drepanocyt* or "haemoglobin ss" or "hemoglobin ss" or "sickle anaemia" or "sickle anemia" or Pompe or "glycogen storage disease type 2" or "cardiomuscular glycogenosis" or "diffuse glycogenosis" or "glycogen storage disease type ii" or (glycogenos* adj2 (ii or targeted gene repair)) or Canavan* or "AADC deficien*" or Fabry* or "alpha galactosidase deficiency syndrome" or Mucopolysaccharidos* or "familial hypercholesterol*" or "buerger gruetz" or "burger grutz" or "familial hyperbetalipoprotein*" or "amilial hypercholester*" or "dilated cardiomyopathy" or "congestic cardiomyopathy" or "congestive heart disease" or "congestive myocardiopathy" or "cystic fibrosis" or "cystic pancreas fibrosis" or "fibrocystic disease" or "Alpha-1 antitrypsin deficiency" or "alpha 1 proteinase inhibitor deficiency" or "alpha1 antitrypsin deficiency" or "antitrypsin alpha 1 deficiency" or "ciliary dyskinesia" or "ciliary immotility" or "ciliatry motility disorders" or ciliostasis or "macula* degeneration" or "macula* atrophy" or "macular dystrophy" or LCA or Leber).ti,ab. (1546055)
**47** exp retina dystrophy/ (5296)
**48** retinitis pigmentosa/ (13362)
**49** color blindness/ (2021)
**50** Duchenne muscular dystrophy/ (20330)
**51** Becker muscular dystrophy/ (3590)
**52** sarcoglycanopathy/ (348)
**53** Friedreich ataxia/ (5028)
**54** metachromatic leukodystrophy/ (2429)
**55** adenylosuccinate lyase/ (454)
**56** happy puppet syndrome/ (3332)
**57** Werdnig Hoffmann disease/ (1410)
**58** spinal muscular atrophy type 2/ (635)
**59** Kugelberg Welander disease/ (714)
**60** limb girdle muscular dystrophy/ (4278)
**61** GM1 gangliosidosis/ (1100)
**62** aromatic levo amino acid decarboxylase/ (3428)
**63** globoid cell leukodystrophy/ (2158)
**64** Rett syndrome/ (6379)
**65** (LCA* or SMA or DMD or XMTM or LGMD or CMT or PD or MPS or Ho-FH or DCM or CF or AATD or PCD).ti,ab. (548959)
**66** ("retinal dystroph*" or "Retinitis Pigmentosa" or RP or "retinal pigment* dystroph*" or "tapeto retinal degeneration" or "tapetoretinal degeneration" or "tapetoretinal dystrophy").ti,ab. (79169)
**67** (achromatognosia or achromatopia or achromatopsia or "colour blind*" or "color blind*" or "color agnosia" or "colour agnosia" or "scoterythrous vision" or "duchenne syndrome" or "duchenne type muscular dystrophy" or "duchenne muscular dystrophy" or "morbus

duchenne" or "pseudo hypertrophic myopathic progressive muscular dystrophy").ti,ab. (18435)
**68** ("backer muscular dystrophy" or "becker muscular dystrophy" or "dystrophic becker disease" or "becker dystrophy" or "becker type muscular dystrophy").ti,ab. (2569)
**69** (sarcoglycanopath* or Friedreich* or "hereditary spinal ataxia" or "spinal hereditary ataxia" or "spinal heredoataxia").ti,ab. (4744)
**70** ("cerebroside sulfatase deficiency syndrome" or "cerebroside sulfate storage disease" or "cerebroside sulphate storage disease" or "metachrom* leucodystrophy" or "metachrom* leukodystrophy" or "sulfatide lipidosis" or sulfatidosis or "sulphatide lipidosis").ti,ab. (1839)
**71** ("adenylosuccinate lyase" or adenylosuccinase or "adenylosuccinate amp lyase" or "adenylsuccinate lyase" or "e.c. 4.3.2.2" or "succinyladenylate lyase").ti,ab. (381)
**72** ("Angelman syndrome" or "happy puppet syndrome").ti,ab. (2211)
**73** ("hereditary progressive spinal muscular atrophy" or "hereditary spinal progressive muscular atrophy" or "hoffmann werdnig" or "werdnig hoffman" or ((infantile or "type 1" or "type I") adj3 "musc* atrophy")).ti,ab. (1090)
**74** (Dubowitz or (("type 2" or "type II") adj3 "musc* atrophy")).ti,ab. (702)
**75** (kugelberg* or ((benign or juvenile or "type 3" or "type III") adj3 "musc* atrophy")).ti,ab. (584)
**76** ("limb girdle dystroph*" or "limb-girdle muscular dystroph*").ti,ab. (3733)
**77** ("GM1 gangliosidosis" or "gangliosidosis G(M1)" or "gangliosidosis gm 1" or "gangliosidosis GM1" or "gm 1 gangliosidosis").ti,ab. (924)
**78** ("Aromatic l-amino acid decarboxylase" or "3,4 dihydroxy l phenylalanine carboxylyase" or "3,4 dihydroxyphenylalanine decarboxylase" or "5 hydroxy levo tryptophan carboxylyase" or "5 hydroxytrypthophan decarboxylase" or "5 hydroxytryptophan carboxylyase" or "5 hydroxytryptophan decarboxylase" or "5 hydroxytryptophane decarboxylase" or "aromatic amino acid decarboxylase*" or "aromatic aminoacid decarboxylase*" or "aromatic l amino acid carboxy lyase" or "aromatic l amino acid decarboxylase*" or "aromatic l aminoacid decarboxylase" or "aromatic levo aminoacid decarboxylase" or "dihydroxyphenylalanine decarboxylase" or "DOPA decarboxylase" or "e.c. 4.1.1.28" or "hydroxytryptophan decarboxylase" or "l 3,4 dihydroxyphenylalanine decarboxylase" or "l dopa decarboxylase" or "l tryptophan carboxylyase" or "levo dopa decarboxylase" or "levodopa decarboxylase" or "tryptophan decarboxylase").ti,ab. (4204)
**79** (krabbe or "beta galactosidase deficiency syndrome" or "galactosylceramidase deficiency syndrome" or "galactosylceramide lipidosis" or "globoid cell dystrophy" or "globoid cell leucodystrophy" or "globoid cell leukodystrophy" or "mckusick 24520").ti,ab. (1625)
**80** (rett or rett's).ti,ab. (5514)
**81** 18 or 19 or 20 or 21 or 22 or 23 or 24 or 25 or 26 or 27 or 28 or 29 or 30 or 31 or 32 or 33 or 34 or 35 or 36 or 37 or 38 or 39 or 40 or 41 or 42 or 43 or 44 or 45 or 46 or 47 or 48 or 49 or 50 or 51 or 52 or 53 or 54 or 55 or 56 or 57 or 58 or 59 or 60 or 61 or 62 or 63 or 64 or 65 or 66 or 67 or 68 or 69 or 70 or 71 or 72 or 73 or 74 or 75 or 76 or 77 or 78 or 79 or 80 (2541161)
**82** exp pharmacovigilance/ (23261)
**83** (Pharmacovigilan* or pharmaco-vigilan* or toxicit* or SUSAR or SAE or AR or SAR or "adverse reaction*" or "adverse event*" or antibodies or immunosuppress* or immunotherap* or "immune management" or immunomodulat* or "subclinical immune response*" or immunologic* or aminotransaminase* or ((liver or hepatic) adj2 (damag* or injur* or inflamm*)) or leukemia or leukaemia or cancer* or tumor* or tumour* or humoral or "cellular immunity" or "innate immunity" or immunogenicity or "complement activation" or cytokine* or "pre-existing immunity" or ADA or cell-mediated or binding-antibod* or "induced response*" or pre-existing or preexisting or boosted or "capsid response*" or

redosing or "cell-mediated immun*" or "T-cell immunity" or tolerance or tolerization or TNF-alpha or prednisolone or eculizumab or "complement activation" or transaminitis or hypertransaminasemia or thrombocytopenia or microangiopathy).ti,ab. (8022126)
**84** exp immunosuppressive treatment/ (260883)
**85** exp leukemia/ (378133)
**86** exp neoplasm/ (5678672)
**87** exp liver injury/ (81741)
**88** exp immunogenicity/ (89304)
**89** exp cytokine/ (1961953)
**90** prednisolone/ (153065)
**91** eculizumab/ (9820)
**92** exp thrombocytopenia/ (232160)
**93** exp microangiopathy/ or hypertransaminasemia/ (79139)
**94** spinal ganglion/ (26536)
**95** exp hemolytic anemia/ (140088)
**96** kidney injury/ or acute kidney failure/ (175604)
**97** exp liver toxicity/ (103332)
**98** (TMA or "dorsal root ganglion" or "dorsal root spinal ganglion" or "spinal ganglia" or "spine ganglion" or DRG or "hemolytic anemia" or "hemolytic anaemia" or "haemolytic anemia" or "haemolytic anaemia" or "acute kidney damage" or "acute kidney injury" or "acute kidney failure" or "acute renal damage" or "acute renal failure" or "elevated liver enzyme*" or "elevated hepatic enzyme*" or hepatotoxic* or "hepato toxic*" or "liver cell toxicit*" or "liver intoxication" or "liver poison*" or "liver toxic*" or "abnormal T2 hyperintensit*").ti,ab. (222334)
**99** 82 or 83 or 84 or 85 or 86 or 87 or 88 or 89 or 90 or 91 or 92 or 93 or 94 or 95 or 96 or 97 or 98 (10809812)
**100** 4 and 8 and 17 and 81 and 99 (2586)

This search strategy uses an adapted version of the RCT filter for Ovid MEDLINE from the Cochrane Highly Sensitive Search Strategy for identifying controlled trials in MEDLINE, courtesy of: https://libraryguides.mcgill.ca/epib629/rct-filters#s-lg-box-wrapper-13740118
**Source: Box 3.c., Technical Supplement to Chapter 4: Searching for and Selecting Studies.** Cochrane Handbook for Systematic Reviews of Interventions Version 6.

**Database: Medline (Ovid MEDLINE® Epub Ahead of Print, In-Process & Other Non-Indexed Citations, Ovid MEDLINE® Daily and Ovid MEDLINE®) 1946 to present**
**Search Strategy:**
**1** exp Genetic Therapy/ (54409)
**2** ("DNA therap*" or (gene* adj2 therap*) or "gene* transfer*" or transgene or "gene replacement*" or "gene-edit*" or "gene silencing" or "gene repair*" or "gene correction*").ti,ab. (162791)
**3** 1 or 2 (181624)
**4** ((randomized controlled trial or controlled clinical trial).pt. or randomized.ab. or randomised.ab. or placebo.ab. or drug therapy.fs. or randomly.ab. or trial.ab. or groups.ab.) not (exp animals/ not humans.sh.) (5177269)
**5** clinical trial/ or clinical trial, phase i/ or clinical trial, phase ii/ or clinical trial, phase iii/ (602002)

**6** ("clinical trial*" or "phase i" or "phase 1" or "phase ii" or "phase 2" or "phase iii" or "phase 3" or "interventional trial*" or "paediatric trial*" or "pediatric trial*" or "pivotal trial*" or "open label*" or "orphan drug*" or "rare disease*").ti,ab. (709665)
**7** Rare Diseases/ (14380)
**8** Orphan Drug Production/ (1465)
**9** 4 or 5 or 6 or 7 or 8 (5540535)
**10** Epidural Space/ (4886)
**11** exp administration, intravenous/ or administration, ophthalmic/ (150896)
**12** exp injections, intraocular/ or injections, intravenous/ or exp injections, spinal/ (110553)
**13** (Intravenous* or intra-venous* or IV or systemic or infusion* or intramuscular* or intra-muscular* or IM or intrathecal* or intra-thecal* or "cerebrospinal fluid*" or CSF or intra-CSF or epidural* or spinal* or ophthalmol* or subretinal* or sub-retinal* or intravitreal* or intra-vitreal* or intraorbital* or intra-orbital* or intracerebroventricular* or intra-cerebroventricular* or ICV or "i.c.v. injection*" or ICVI or intracerebral* or intra-cerebral* or IC or intraventricular* or intra-ventricular* or intraparenchyma* or intra-parenchyma* or "intra-cisterna magna" or ICM).ti,ab. (2316440)
**14** 10 or 11 or 12 or 13 (2362480)
**15** Rare Diseases/ (14380)
**16** Orphan Drug Production/ (1465)
**17** exp Muscular Atrophy, Spinal/ (6639)
**18** exp Myopathies, Structural, Congenital/ (1624)
**19** exp Muscular Dystrophies/ (30356)
**20** exp Paraplegia/ (13603)
**21** exp Gangliosidoses/ (2695)
**22** exp Multiple Sclerosis/ (71739)
**23** Parkinson Disease/ (84852)
**24** Alzheimer Disease/ (123730)
**25** Neuronal Ceroid-Lipofuscinoses/ (2273)
**26** exp "Hereditary Sensory and Motor Neuropathy"/ (8261)
**27** hemophilia a/ or hemophilia b/ (25211)
**28** exp Anemia, Sickle Cell/ (26145)
**29** Glycogen Storage Disease Type II/ (1995)
**30** Canavan Disease/ (313)
**31** Fabry Disease/ (4224)
**32** exp Mucopolysaccharidoses/ (7094)
**33** Hyperlipoproteinemia Type II/ (7796)
**34** Cardiomyopathy, Dilated/ (17408)
**35** Cystic Fibrosis/ (40582)
**36** alpha 1-Antitrypsin Deficiency/ (3789)
**37** exp Ciliary Motility Disorders/ (2699)
**38** exp Macular Degeneration/ (31035)
**39** Leber Congenital Amaurosis/ (586)
**40** ("rare disease*" or "orphan drug*" or "monogenic disease*" or "monogenic disorder*" or "spinal muscular atrophy" or "spine muscle atrophy" or "myotubular myopathy" or "centronuclear myopathy" or "myotonic dystrophy" or "curschmann steinert disease" or "deleage disease" or "dystrophic myotonica" or "dystrophic myotonia" or "myotonia

atrophica" or "myotonia dystrophica" or "myotonic atrophia" or "myotonic atrophy" or "myotonic dystrophia" or "myotonic muscular dystrophy" or "myotonic muscular dystrophy" or "proximal myotonic myopathy" or "steinert disease" or "steinert myopathy" or "muscular dystrophy" or "muscle dystrophy" or "muscle dystrophia" or "motor neuron* disease" or MND or "Spastic paraplegia" or "spastic paraparesis" or gangliosidosis or "ganglioside lipidosis" or gangliosidoses or "multiple sclerosis" or "chariot disease" or "disseminated sclerosis" or "insular sclerosis" or MS or Parkinson* or "paralysis agitans" or Alzheimer* or Alzeimer* or "diffuse cortical sclerosis" or Batten* or "juvenile neuronal ceroid lipofuscinosis" or "Giant axonal neuropathy" or GAN or GAN-1 or "Amyotrophic lateral sclerosis" or ALS or "lou gehrig*" or Charcot-Marie-Tooth or "hereditary motor sensory neuropathy" or "charcot-marie" or "dejerine sottas" or "familial spastic para*" or "hereditary spastic para*" or Struempell or strumpel* or SCID-X or Haemophili* or hemophili* or "sickle cell" or drepanocyt* or "haemoglobin ss" or "hemoglobin ss" or "sickle anaemia" or "sickle anemia" or Pompe or "glycogen storage disease type 2" or "cardiomuscular glycogenosis" or "diffuse glycogenosis" or "glycogen storage disease type ii" or (glycogenos* adj2 (ii or targeted gene repair)) or Canavan* or "AADC deficien*" or Fabry* or "alpha galactosidase deficiency syndrome" or Mucopolysaccharidos* or "familial hypercholesterol*" or "buerger gruetz" or "burger grutz" or "familial hyperbetalipoprotein*" or "amilial hypercholester*" or "dilated cardiomyopathy" or "congestic cardiomyopathy" or "congestive heart disease" or "congestive myocardiopathy" or "cystic fibrosis" or "cystic pancreas fibrosis" or "fibrocystic disease" or "Alpha-1 antitrypsin deficiency" or "alpha 1 proteinase inhibitor deficiency" or "alpha1 antitrypsin deficiency" or "antitrypsin alpha 1 deficiency" or "ciliary dyskinesia" or "ciliary immotility" or "ciliatry motility disorders" or ciliostasis or "macula* degeneration" or "macula* atrophy" or "macular dystrophy" or LCA or Leber).ti,ab. (1119488)
**41** exp Retinal Dystrophies/ (10905)
**42** exp Retinitis Pigmentosa/ (10845)
**43** Color Vision Defects/ (4266)
**44** Friedreich Ataxia/ (2824)
**45** Leukodystrophy, Metachromatic/ (1330)
**46** Adenylosuccinate Lyase/ (267)
**47** Angelman Syndrome/ (1406)
**48** Leukodystrophy, Globoid Cell/ (1091)
**49** Rett Syndrome/ (3003)
**50** (LCA* or SMA or DMD or XMTM or LGMD or CMT or PD or MPS or Ho-FH or DCM or CF or AATD or PCD).ti,ab. (340964)
**51** ("retinal dystroph*" or "Retinitis Pigmentosa" or RP or "retinal pigment* dystroph*" or "tapeto retinal degeneration" or "tapetoretinal degeneration" or "tapetoretinal dystrophy").ti,ab. (49225)
**52** (achromatognosia or achromatopia or achromatopsia or "colour blind*" or "color blind*" or "color agnosia" or "colour agnosia" or "scoterythrous vision" or "duchenne syndrome" or "duchenne type muscular dystrophy" or "duchenne muscular dystrophy" or "morbus duchenne" or "pseudo hypertrophic myopathic progressive muscular dystrophy").ti,ab. (13233)
**53** ("backer muscular dystrophy" or "becker muscular dystrophy" or "dystrophic becker disease" or "becker dystrophy" or "becker type muscular dystrophy").ti,ab. (1795)

**54** (sarcoglycanopath* or Friedreich* or "hereditary spinal ataxia" or "spinal hereditary ataxia" or "spinal heredoataxia").ti,ab. (3759)
**55** ("cerebroside sulfatase deficiency syndrome" or "cerebroside sulfate storage disease" or "cerebroside sulphate storage disease" or "metachrom* leucodystrophy" or "metachrom* leukodystrophy" or "sulfatide lipidosis" or sulfatidosis or "sulphatide lipidosis").ti,ab. (1483)
**56** ("adenylosuccinate lyase" or adenylosuccinase or "adenylosuccinate amp lyase" or "adenylsuccinate lyase" or "e.c. 4.3.2.2" or "succinyladenylate lyase").ti,ab. (346)
**57** ("Angelman syndrome" or "happy puppet syndrome").ti,ab. (1726)
**58** ("hereditary progressive spinal muscular atrophy" or "hereditary spinal progressive muscular atrophy" or "hoffmann werdnig" or "werdnig hoffman" or ((infantile or "type 1" or "type I") adj3 "musc* atrophy")).ti,ab. (680)
**59** (Dubowitz or (("type 2" or "type II") adj3 "musc* atrophy")).ti,ab. (472)
**60** (kugelberg* or ((benign or juvenile or "type 3" or "type III") adj3 "musc* atrophy")).ti,ab. (515)
**61** ("limb girdle dystroph*" or "limb-girdle muscular dystroph*").ti,ab. (2360)
**62** ("GM1 gangliosidosis" or "gangliosidosis G(M1)" or "gangliosidosis gm 1" or "gangliosidosis GM1" or "gm 1 gangliosidosis").ti,ab. (784)
**63** ("Aromatic l-amino acid decarboxylase" or "3,4 dihydroxy l phenylalanine carboxylyase" or "3,4 dihydroxyphenylalanine decarboxylase" or "5 hydroxy levo tryptophan carboxylyase" or "5 hydroxytrypthophan decarboxylase" or "5 hydroxytryptophan carboxylyase" or "5 hydroxytryptophan decarboxylase" or "5 hydroxytryptophane decarboxylase" or "aromatic amino acid decarboxylase*" or "aromatic aminoacid decarboxylase*" or "aromatic l amino acid carboxy lyase" or "aromatic l amino acid decarboxylase*" or "aromatic l aminoacid decarboxylase" or "aromatic levo aminoacid decarboxylase" or "dihydroxyphenylalanine decarboxylase" or "DOPA decarboxylase" or "e.c. 4.1.1.28" or "hydroxytryptophan decarboxylase" or "l 3,4 dihydroxyphenylalanine decarboxylase" or "l dopa decarboxylase" or "l tryptophan carboxylyase" or "levo dopa decarboxylase" or "levodopa decarboxylase" or "tryptophan decarboxylase").ti,ab. (3606)
**64** (krabbe or "beta galactosidase deficiency syndrome" or "galactosylceramidase deficiency syndrome" or "galactosylceramide lipidosis" or "globoid cell dystrophy" or "globoid cell leucodystrophy" or "globoid cell leukodystrophy" or "mckusick 24520").ti,ab. (1224)
**65** (rett or rett's).ti,ab. (4265)
**66** 15 or 16 or 17 or 18 or 19 or 20 or 21 or 22 or 23 or 24 or 25 or 26 or 27 or 28 or 29 or 30 or 31 or 32 or 33 or 34 or 35 or 36 or 37 or 38 or 39 or 40 or 41 or 42 or 43 or 44 or 45 or 46 or 47 or 48 or 49 or 50 or 51 or 52 or 53 or 54 or 55 or 56 or 57 or 58 or 59 or 60 or 61 or 62 or 63 or 64 or 65 (1506235)
**67** pharmacovigilance/ (3515)
**68** (Pharmacovigilan* or pharmaco-vigilan* or toxicit* or SUSAR or SAE or AR or SAR or "adverse reaction*" or "adverse event*" or antibodies or immunosuppress* or immunotherap* or "immune management" or immunomodulat* or "subclinical immune response*" or immunologic* or aminotransaminase* or ((liver or hepatic) adj2 (damag* or injur* or inflamm*)) or leukemia or leukaemia or cancer* or tumor* or tumour* or humoral or "cellular immunity" or "innate immunity" or immunogenicity or "complement activation" or cytokine* or "pre-existing immunity" or ADA or cell-mediated or binding-antibod* or "induced response*" or pre-existing or preexisting or boosted or "capsid response*" or redosing or "cell-mediated immun*" or "T-cell immunity" or tolerance or tolerization or

TNF-alpha or prednisolone or eculizumab or "complement activation" or transaminitis or hypertransaminasemia or thrombocytopenia or microangiopathy).ti,ab. (5956431)
**69** exp Immunosuppressive Agents/ (348675)
**70** exp Leukemia/ (257251)
**71** exp Neoplasms/ (3941650)
**72** exp "Chemical and Drug Induced Liver Injury"/ (34033)
**73** exp Immunogenetic Phenomena/ (49969)
**74** exp Cytokines/ (805065)
**75** exp Prednisolone/ (54238)
**76** exp Thrombocytopenia/ (54496)
**77** Ganglia, Spinal/ (20340)
**78** exp Anemia, Hemolytic/ (82573)
**79** exp Acute Kidney Injury/ (57686)
**80** (TMA or "dorsal root ganglion" or "dorsal root spinal ganglion" or "spinal ganglia" or "spine ganglion" or DRG or "hemolytic anemia" or "hemolytic anaemia" or "haemolytic anemia" or "haemolytic anaemia" or "acute kidney damage" or "acute kidney injury" or "acute kidney failure" or "acute renal damage" or "acute renal failure" or "elevated liver enzyme*" or "elevated hepatic enzyme*" or hepatotoxic* or "hepato toxic*" or "liver cell toxicit*" or "liver intoxication" or "liver poison*" or "liver toxic*" or "abnormal T2 hyperintensit*").ti,ab. (148467)
**81** 67 or 68 or 69 or 70 or 71 or 72 or 73 or 74 or 75 or 76 or 77 or 78 or 79 or 80 (7851538)
**82** 3 and 9 and 14 and 66 and 81 (536)

**Cochrane Central Register of Controlled Trials**
**Issue 2 of 12, February 2024**
#1 MeSH descriptor: [Genetic Therapy] explode all trees 358
#2 ((DNA NEXT therap*) or (gene* near/2 therap*) or (gene* NEXT transfer*) or transgene or (gene NEXT replacement*) or (gene* next edit*) or "gene silencing" or (gene NEXT repair*) or (gene NEXT correction*)):ti,ab,kw 18478
#3 #1 OR #2 18491
#4 MeSH descriptor: [Infusion Pumps] explode all trees 1637
#5 MeSH descriptor: [Epidural Space] explode all trees 294
#6 MeSH descriptor: [Administration, Intravenous] explode all trees 22798
#7 MeSH descriptor: [Administration, Ophthalmic] explode all trees 180
#8 MeSH descriptor: [Injections, Intraocular] explode all trees 1494
#9 MeSH descriptor: [Injections, Intravenous] explode all trees 9025
#10 MeSH descriptor: [Injections, Spinal] explode all trees 1823
#11 (Intravenous* or intra-venous* or IV or systemic or infusion* or intramuscular* or intra-muscular* or IM or intrathecal* or (intra NEXT thecal*) or (cerebrospinal NEXT fluid*) or CSF or intra-CSF or epidural* or spinal* or ophthalmol* or subretinal* or sub-retinal* or intravitreal* or intra-vitreal* or intraorbital* or intra-orbital* or intracerebroventricular* or intra-cerebroventricular* or ICV or (i.c.v. NEXT injection*) or ICVI or intracerebral* or intra-cerebral* or IC or intraventricular* or (intra NEXT ventricular*) or intraparenchyma* or (intra NEXT parenchyma*) or "intra-cisterna magna" or ICM):ti,ab,kw 338369
#12 #4 or #5 or #6 or #7 or #8 or #9 or #10 or #11 338571
#13 MeSH descriptor: [Rare Diseases] explode all trees 77
#14 MeSH descriptor: [Orphan Drug Production] explode all trees 19

#15 MeSH descriptor: [Muscular Atrophy, Spinal] explode all trees 148
#16 MeSH descriptor: [Myopathies, Structural, Congenital] explode all trees 6
#17 MeSH descriptor: [Muscular Dystrophies] explode all trees 659
#18 MeSH descriptor: [Paraplegia] explode all trees 279
#19 MeSH descriptor: [Gangliosidoses] explode all trees 11
#20 MeSH descriptor: [Multiple Sclerosis] explode all trees 5178
#21 MeSH descriptor: [Parkinson Disease] explode all trees 6009
#22 MeSH descriptor: [Alzheimer Disease] explode all trees 5232
#23 MeSH descriptor: [Neuronal Ceroid-Lipofuscinoses] explode all trees 11
#24 MeSH descriptor: [Hereditary Sensory and Motor Neuropathy] explode all trees 119
#25 MeSH descriptor: [Hemophilia A] explode all trees 641
#26 MeSH descriptor: [Anemia, Sickle Cell] explode all trees 1061
#27 MeSH descriptor: [Glycogen Storage Disease Type II] explode all trees 56
#28 MeSH descriptor: [Canavan Disease] explode all trees 3
#29 MeSH descriptor: [Fabry Disease] explode all trees 110
#30 MeSH descriptor: [Mucopolysaccharidoses] explode all trees 106
#31 MeSH descriptor: [Hyperlipoproteinemia Type II] explode all trees 664
#32 MeSH descriptor: [Cardiomyopathy, Dilated] explode all trees 669
#33 MeSH descriptor: [Cystic Fibrosis] explode all trees 2368
#34 MeSH descriptor: [alpha 1-Antitrypsin Deficiency] explode all trees 120
#35 MeSH descriptor: [Ciliary Motility Disorders] explode all trees 32
#36 MeSH descriptor: [Macular Degeneration] explode all trees 3542
#37 MeSH descriptor: [Leber Congenital Amaurosis] explode all trees 14
#38 ((rare NEXT disease*) or (orphan NEXT drug*) or (monogenic NEXT disease*) or (monogenic NEXT disorder*) or "spinal muscular atrophy" or "spine muscle atrophy" or "myotubular myopathy" or "centronuclear myopathy" or "myotonic dystrophy" or "curschmann steinert disease" or "deleage disease" or "dystrophic myotonica" or "dystrophic myotonia" or "myotonia atrophica" or "myotonia dystrophica" or "myotonic atrophia" or "myotonic atrophy" or "myotonic dystrophia" or "myotonic muscular dystrophy" or "myotonic muscular dystrophy" or "proximal myotonic myopathy" or "steinert disease" or "steinert myopathy" or "muscular dystrophy" or "muscle dystrophy" or "muscle dystrophia" or (neuron* NEXT disease) or MND or "Spastic paraplegia" or "spastic paraparesis" or gangliosidosis or "ganglioside lipidosis" or gangliosidoses or "multiple sclerosis" or "chariot disease" or "disseminated sclerosis" or "insular sclerosis" or MS or Parkinson* or "paralysis agitans" or Alzheimer* or Alzeimer* or "diffuse cortical sclerosis" or Batten* or "juvenile neuronal ceroid lipofuscinosis" or "Giant axonal neuropathy" or GAN or GAN-1 or "Amyotrophic lateral sclerosis" or ALS or (lou NEXT gehrig*) or Charcot-Marie-Tooth or "hereditary motor sensory neuropathy" or charcot-marie or "dejerine sottas" or (spastic NEXT para*) or Struempell or strumpel* or SCID-X or Haemophili* or hemophili* or "sickle cell" or drepanocyt* or "haemoglobin ss" or "hemoglobin ss" or "sickle anaemia" or "sickle anemia" or Pompe or "glycogen storage disease type 2" or "cardiomuscular glycogenosis" or "diffuse glycogenosis" or "glycogen storage disease type ii" or (glycogenos* NEAR/2 (ii or targeted gene repair)) or Canavan* or (AADC NEXT deficien*) or Fabry* or "alpha galactosidase deficiency syndrome" or Mucopolysaccharidos* or (familial NEXT hypercholesterol*) or "buerger gruetz" or "burger grutz" or (familial NEXT hyperbetalipoprotein*) or (amilial NEXT hypercholester*) or "dilated cardiomyopathy" or "congestic cardiomyopathy" or "congestive heart disease" or "congestive myocardiopathy" or "cystic fibrosis" or "cystic pancreas fibrosis" or "fibrocystic disease" or "Alpha-1 antitrypsin deficiency" or "alpha 1 proteinase inhibitor deficiency" or "alpha1 antitrypsin deficiency" or "antitrypsin alpha 1 deficiency" or "ciliary dyskinesia" or "ciliary immotility" or "ciliatry

motility disorders" or ciliostasis or (macula* NEXT degeneration) or (macula* NEXT atrophy) or "macular dystrophy" or LCA or Leber):ti,ab,kw 79421
#39 MeSH descriptor: [Retinal Dystrophies] explode all trees 164
#40 MeSH descriptor: [Retinitis Pigmentosa] explode all trees 157
#41 MeSH descriptor: [Color Vision Defects] explode all trees 50
#42 MeSH descriptor: [Friedreich Ataxia] explode all trees 98
#43 MeSH descriptor: [Leukodystrophy, Metachromatic] explode all trees 6
#44 MeSH descriptor: [Adenylosuccinate Lyase] explode all trees 0
#45 MeSH descriptor: [Angelman Syndrome] explode all trees 27
#46 MeSH descriptor: [Leukodystrophy, Globoid Cell] explode all trees 2
#47 MeSH descriptor: [Rett Syndrome] explode all trees 64
#48 (LCA* or SMA or DMD or XMTM or LGMD or CMT or PD or MPS or Ho-FH or DCM or CF or AATD or PCD):ti,ab,kw 54647
#49 ((retinal NEXT dystroph*) or "Retinitis Pigmentosa" or RP or (pigment* NEXT dystroph*) or "tapeto retinal degeneration" or "tapetoretinal degeneration" or "tapetoretinal dystrophy"):ti,ab,kw 3262
#50 (achromatognosia or achromatopia or achromatopsia or (colour NEXT blind*) or (color NEXT blind*) or "color agnosia" or "colour agnosia" or "scoterythrous vision" or "duchenne syndrome" or "duchenne type muscular dystrophy" or "duchenne muscular dystrophy" or "morbus duchenne" or "pseudo hypertrophic myopathic progressive muscular dystrophy"):ti,ab,kw 914
#51 ("backer muscular dystrophy" or "becker muscular dystrophy" or "dystrophic becker disease" or "becker dystrophy" or "becker type muscular dystrophy"):ti,ab,kw 84
#52 (sarcoglycanopath* or Friedreich* or "hereditary spinal ataxia" or "spinal hereditary ataxia" or "spinal heredoataxia"):ti,ab,kw 177
#53 ("cerebroside sulfatase deficiency syndrome" or "cerebroside sulfate storage disease" or "cerebroside sulphate storage disease" or (metachrom* NEXT leucodystrophy) or (metachrom* NEXT leukodystrophy) or "sulfatide lipidosis" or sulfatidosis or "sulphatide lipidosis"):ti,ab,kw 9
#54 ("adenylosuccinate lyase" or adenylosuccinase or "adenylosuccinate amp lyase" or "adenylsuccinate lyase" or "e.c. 4.3.2.2" or "succinyladenylate lyase"):ti,ab,kw 1
#55 ("Angelman syndrome" or "happy puppet syndrome"):ti,ab,kw 42
#56 ("hereditary progressive spinal muscular atrophy" or "hereditary spinal progressive muscular atrophy" or "hoffmann werdnig" or "werdnig hoffman" or ((infantile or "type 1" or "type I") near/4 atrophy)):ti,ab,kw 33
#57 (Dubowitz or (("type 2" or "type II") near/4 atrophy)):ti,ab,kw 78
#58 (kugelberg* or ((benign or juvenile or "type 3" or "type III") near/4 atrophy)):ti,ab,kw 74
#59 ((girdle NEXT dystroph*) or ("limb-girdle muscular" NEXT dystroph*)):ti,ab,kw 41
#60 ("GM1 gangliosidosis" or "gangliosidosis G(M1)" or "gangliosidosis gm 1" or "gangliosidosis GM1" or "gm 1 gangliosidosis"):ti,ab,kw 7
#61 ("Aromatic l-amino acid decarboxylase" or "3,4 dihydroxy l phenylalanine carboxylyase" or "3,4 dihydroxyphenylalanine decarboxylase" or "5 hydroxy levo tryptophan carboxylyase" or "5 hydroxytrypthophan decarboxylase" or "5 hydroxytryptophan carboxylyase" or "5 hydroxytryptophan decarboxylase" or "5 hydroxytryptophane decarboxylase" or ("aromatic amino acid" NEXT decarboxylase*) or ("aromatic aminoacid" NEXT decarboxylase*) or "aromatic l amino acid carboxy lyase" or ("aromatic l amino acid" NEXT decarboxylase*) or "aromatic l aminoacid decarboxylase" or "aromatic levo aminoacid decarboxylase" or "dihydroxyphenylalanine decarboxylase" or "DOPA decarboxylase" or "e.c. 4.1.1.28" or "hydroxytryptophan decarboxylase" or "l 3,4

dihydroxyphenylalanine decarboxylase" or "l dopa decarboxylase" or "l tryptophan carboxylyase" or "levo dopa decarboxylase" or "levodopa decarboxylase" or "tryptophan decarboxylase"):ti,ab,kw 241

#62 (krabbe or "beta galactosidase deficiency syndrome" or "galactosylceramidase deficiency syndrome" or "galactosylceramide lipidosis" or "globoid cell dystrophy" or "globoid cell leucodystrophy" or "globoid cell leukodystrophy" or "mckusick 24520"):ti,ab,kw 8

#63 (rett or rett's):ti,ab,kw 121

#64 #13 or #14 or #15 or #16 or #17 or #18 or #19 or #20 or #21 or #22 or #23 or #24 or #25 or #26 or #27 or #28 or #29 or #30 or #31 or #32 or #33 or #34 or #35 or #36 or #37 or #38 or #39 or #40 or #41 or #42 or #43 or #44 or #45 or #46 or #47 or #48 or #49 or #50 or #51 or #52 or #53 or #54 or #55 or #56 or #57 or #58 or #59 or #60 or #61 or #62 or #63 125949

#65 MeSH descriptor: [Pharmacovigilance] explode all trees 42

#66 (Pharmacovigilan* or pharmaco-vigilan* or toxicit* or SUSAR or SAE or AR or SAR or (adverse NEXT reaction*) or (adverse NEXT event*) or antibodies or immunosuppress* or immunotherap* or "immune management" or immunomodulat* or ("subclinical immune" NEXT response*) or immunologic* or aminotransaminase* or ((liver or hepatic) NEAR/2 (damag* or injur* or inflamm*)) or leukemia or leukaemia or cancer* or tumor* or tumour* or humoral or "cellular immunity" or "innate immunity" or immunogenicity or "complement activation" or cytokine* or "pre-existing immunity" or ADA or cell-mediated or (binding NEXT antibod*) or (induced NEXT response*) or pre-existing or preexisting or boosted or (capsid NEXT response*) or redosing or (cell-mediated NEXT immun*) or "T-cell immunity" or tolerance or tolerization or TNF-alpha or prednisolone or eculizumab or "complement activation" or transaminitis or hypertransaminasemia or thrombocytopenia or microangiopathy):ti,ab,kw 526912

#67 MeSH descriptor: [Immunosuppressive Agents] explode all trees 6937

#68 MeSH descriptor: [Leukemia] explode all trees 6529

#69 MeSH descriptor: [Neoplasms] explode all trees 123839

#70 MeSH descriptor: [Chemical and Drug Induced Liver Injury] explode all trees 474

#71 MeSH descriptor: [Immunogenetic Phenomena] explode all trees 135

#72 MeSH descriptor: [Cytokines] explode all trees 27506

#73 MeSH descriptor: [Prednisolone] explode all trees 5996

#74 MeSH descriptor: [Thrombocytopenia] explode all trees 1790

#75 MeSH descriptor: [Ganglia, Spinal] explode all trees 71

#76 MeSH descriptor: [Anemia, Hemolytic] explode all trees 1840

#77 MeSH descriptor: [Acute Kidney Injury] explode all trees 2347

#78 (TMA or "dorsal root ganglion" or "dorsal root spinal ganglion" or "spinal ganglia" or "spine ganglion" or DRG or "hemolytic anemia" or "hemolytic anaemia" or "haemolytic anemia" or "haemolytic anaemia" or "acute kidney damage" or "acute kidney injury" or "acute kidney failure" or "acute renal damage" or "acute renal failure" or ("elevated liver" NEXT enzyme*) or ("elevated hepatic" NEXT enzyme*) or hepatotoxic* or (hepato NEXT toxic*) or ("liver cell" NEXT toxicit*) or "liver intoxication" or (liver NEXT poison*) or (liver NEXT toxic*) or ("abnormal T2" NEXT hyperintensit*)):ti,ab,kw 11367

#79 #65 or #66 or #67 or #68 or #69 or #70 or #71 or #72 or #73 or #74 or #75 or #76 or #77 or #78 560567

#80 #3 and #12 and #64 and #79 732

**Table S2. Search Results for Real-World Studies.**

| | |
|---|---|
| Ovid Embase | 433 |
| Ovid Medline | 44 |
| Cochrane CENTRAL | 31 |
| Total | 508 |
| Total after deduplication | 445 |

**Database: Embase 1974 to present**

Database: Medline (Ovid MEDLINE® Epub Ahead of Print, In-Process & Other Non-Indexed Citations, Ovid MEDLINE® Daily and Ovid MEDLINE®) 1946 to present

Search Strategy:

--------------------------------------------------------------------------------

1 exp gene therapy/ (102165)
2 targeted gene repair/ (445)
3 ("DNA therap*" or (gene* adj2 therap*) or "gene* transfer*" or transgene or "gene replacement*" or "gene-edit*" or "gene silencing" or "gene repair*" or "gene correction*").ti,ab. (212907)
4 1 or 2 or 3 (251960)
5 epidural drug administration/ or intracisternal drug administration/ or intrathecal drug administration/ (27063)
6 intraocular drug administration/ or intracorneal drug administration/ or intravitreal drug administration/ or subretinal drug administration/ (10616)
7 exp intraspinal drug administration/ (29464)
8 exp intraocular drug administration/ (12131)
9 drug infusion/ (17165)
10 exp intracerebral drug administration/ (31720)
11 intravenous drug administration/ (384430)
12 (Intravenous* or intra-venous* or IV or systemic or infusion* or intramuscular* or intra-muscular* or IM or intrathecal* or intra-thecal* or "cerebrospinal fluid*" or CSF or intra-CSF or epidural* or spinal* or ophthalmol* or subretinal* or sub-retinal* or intravitreal* or intra-vitreal* or intraorbital* or intra-orbital* or intracerebroventricular* or intra-cerebroventricular* or ICV or "i.c.v. injection*" or ICVI or intracerebral* or intra-cerebral* or IC or intraventricular* or intra-ventricular* or intraparenchyma* or intra-parenchyma* or "intra-cisterna magna" or ICM).ti,ab. (3195173)
13 5 or 6 or 7 or 8 or 9 or 10 or 11 or 12 (3407200)
14 rare disease/ (50265)
15 orphan drug/ (4013)
16 exp spinal muscular atrophy/ (71765)
17 centronuclear myopathy/ (1323)
18 myotonic dystrophy/ (9744)
19 exp muscular dystrophy/ (52657)
20 spastic paraplegia/ (5101)
21 exp gangliosidosis/ (4390)

22 multiple sclerosis/ (161878)
23 parkinson disease/ or autosomal dominant parkinson disease/ (196772)
24 Alzheimer disease/ (257013)
25 juvenile neuronal ceroid lipofuscinosis/ (295)
26 giant axonal neuropathy/ (320)
27 hereditary motor sensory neuropathy/ (13412)
28 exp hemophilia/ (46582)
29 exp sickle cell anemia/ (47775)
30 glycogen storage disease type 2/ (5209)
31 Canavan disease/ (818)
32 Fabry disease/ (9593)
33 exp mucopolysaccharidosis/ (14039)
34 familial hypercholesterolemia/ (12739)
35 exp congestive cardiomyopathy/ (55227)
36 cystic fibrosis/ (86136)
37 alpha 1 antitrypsin deficiency/ (6642)
38 ciliary dyskinesia/ (3703)
39 exp macular degeneration/ (27716)
40 Leber congenital amaurosis/ (2306)
41 exp monogenic disorder/ (573870)
42 ("rare disease*" or "orphan drug*" or "monogenic disease*" or "monogenic disorder*" or "spinal muscular atrophy" or "spine muscle atrophy" or "myotubular myopathy" or "centronuclear myopathy" or "myotonic dystrophy" or "curschmann steinert disease" or "deleage disease" or "dystrophic myotonica" or "dystrophic myotonia" or "myotonia atrophica" or "myotonia dystrophica" or "myotonic atrophia" or "myotonic atrophy" or "myotonic dystrophia" or "myotonic muscular dystrophy" or "myotonic muscular dystrophy" or "proximal myotonic myopathy" or "steinert disease" or "steinert myopathy" or "muscular dystrophy" or "muscle dystrophy" or "muscle dystrophia" or "motor neuron* disease" or MND or "Spastic paraplegia" or "spastic paraparesis" or gangliosidosis or "ganglioside lipidosis" or gangliosidoses or "multiple sclerosis" or "chariot disease" or "disseminated sclerosis" or "insular sclerosis" or MS or Parkinson* or "paralysis agitans" or Alzheimer* or Alzeimer* or "diffuse cortical sclerosis" or Batten* or "juvenile neuronal ceroid lipofuscinosis" or "Giant axonal neuropathy" or GAN or GAN-1 or "Amyotrophic lateral sclerosis" or ALS or "lou gehrig*" or Charcot-Marie-Tooth or "hereditary motor sensory neuropathy" or "charcot-marie" or "dejerine sottas" or "familial spastic para*" or "hereditary spastic para*" or Struempell or strumpel* or SCID-X or Haemophili* or hemophili* or "sickle cell" or drepanocyt* or "haemoglobin ss" or "hemoglobin ss" or "sickle anaemia" or "sickle anemia" or Pompe or "glycogen storage disease type 2" or "cardiomuscular glycogenosis" or "diffuse glycogenosis" or "glycogen storage disease type ii" or (glycogenos* adj2 (ii or targeted gene repair)) or Canavan* or "AADC deficien*" or Fabry* or "alpha galactosidase deficiency syndrome" or Mucopolysaccharidos* or "familial hypercholesterol*" or "buerger gruetz" or "burger grutz" or "familial hyperbetalipoprotein*" or "amilial hypercholester*" or "dilated cardiomyopathy" or "congestic cardiomyopathy" or "congestive heart disease" or "congestive myocardiopathy" or "cystic fibrosis" or "cystic pancreas fibrosis" or "fibrocystic disease" or "Alpha-1 antitrypsin deficiency" or "alpha 1 proteinase inhibitor deficiency" or "alpha1 antitrypsin deficiency" or "antitrypsin alpha 1 deficiency" or "ciliary dyskinesia" or "ciliary immotility" or "ciliatry motility disorders" or ciliostasis or "macula* degeneration" or "macula* atrophy" or "macular dystrophy" or LCA or Leber).ti,ab. (1546055)
43 exp retina dystrophy/ (5296)

44 retinitis pigmentosa/ (13362)
45 color blindness/ (2021)
46 Duchenne muscular dystrophy/ (20330)
47 Becker muscular dystrophy/ (3590)
48 sarcoglycanopathy/ (348)
49 Friedreich ataxia/ (5028)
50 metachromatic leukodystrophy/ (2429)
51 adenylosuccinate lyase/ (454)
52 happy puppet syndrome/ (3332)
53 Werdnig Hoffmann disease/ (1410)
54 spinal muscular atrophy type 2/ (635)
55 Kugelberg Welander disease/ (714)
56 limb girdle muscular dystrophy/ (4278)
57 GM1 gangliosidosis/ (1100)
58 aromatic levo amino acid decarboxylase/ (3428)
59 globoid cell leukodystrophy/ (2158)
60 Rett syndrome/ (6379)
61 (LCA* or SMA or DMD or XMTM or LGMD or CMT or PD or MPS or Ho-FH or DCM or CF or AATD or PCD).ti,ab. (548959)
62 ("retinal dystroph*" or "Retinitis Pigmentosa" or RP or "retinal pigment* dystroph*" or "tapeto retinal degeneration" or "tapetoretinal degeneration" or "tapetoretinal dystrophy").ti,ab. (79169)
63 (achromatognosia or achromatopia or achromatopsia or "colour blind*" or "color blind*" or "color agnosia" or "colour agnosia" or "scoterythrous vision" or "duchenne syndrome" or "duchenne type muscular dystrophy" or "duchenne muscular dystrophy" or "morbus duchenne" or "pseudo hypertrophic myopathic progressive muscular dystrophy").ti,ab. (18435)
64 ("backer muscular dystrophy" or "becker muscular dystrophy" or "dystrophic becker disease" or "becker dystrophy" or "becker type muscular dystrophy").ti,ab. (2569)
65 (sarcoglycanopath* or Friedreich* or "hereditary spinal ataxia" or "spinal hereditary ataxia" or "spinal heredoataxia").ti,ab. (4744)
66 ("cerebroside sulfatase deficiency syndrome" or "cerebroside sulfate storage disease" or "cerebroside sulphate storage disease" or "metachrom* leucodystrophy" or "metachrom* leukodystrophy" or "sulfatide lipidosis" or sulfatidosis or "sulphatide lipidosis").ti,ab. (1839)
67 ("adenylosuccinate lyase" or adenylosuccinase or "adenylosuccinate amp lyase" or "adenylsuccinate lyase" or "e.c. 4.3.2.2" or "succinyladenylate lyase").ti,ab. (381)
68 ("Angelman syndrome" or "happy puppet syndrome").ti,ab. (2211)
69 ("hereditary progressive spinal muscular atrophy" or "hereditary spinal progressive muscular atrophy" or "hoffmann werdnig" or "werdnig hoffman" or ((infantile or "type 1" or "type I") adj3 "musc* atrophy")).ti,ab. (1090)
70 (Dubowitz or (("type 2" or "type II") adj3 "musc* atrophy")).ti,ab. (702)
71 (kugelberg* or ((benign or juvenile or "type 3" or "type III") adj3 "musc* atrophy")).ti,ab. (584)
72 ("limb girdle dystroph*" or "limb-girdle muscular dystroph*").ti,ab. (3733)
73 ("GM1 gangliosidosis" or "gangliosidosis G(M1)" or "gangliosidosis gm 1" or "gangliosidosis GM1" or "gm 1 gangliosidosis").ti,ab. (924)
74 ("Aromatic l-amino acid decarboxylase" or "3,4 dihydroxy l phenylalanine carboxylyase" or "3,4 dihydroxyphenylalanine decarboxylase" or "5 hydroxy levo tryptophan carboxylyase" or "5 hydroxytrypthophan decarboxylase" or "5 hydroxytryptophan carboxylyase" or "5 hydroxytryptophan decarboxylase" or "5 hydroxytryptophane decarboxylase" or "aromatic

amino acid decarboxylase*" or "aromatic aminoacid decarboxylase*" or "aromatic l amino acid carboxy lyase" or "aromatic l amino acid decarboxylase*" or "aromatic l aminoacid decarboxylase" or "aromatic levo aminoacid decarboxylase" or "dihydroxyphenylalanine decarboxylase" or "DOPA decarboxylase" or "e.c. 4.1.1.28" or "hydroxytryptophan decarboxylase" or "l 3,4 dihydroxyphenylalanine decarboxylase" or "l dopa decarboxylase" or "l tryptophan carboxylyase" or "levo dopa decarboxylase" or "levodopa decarboxylase" or "tryptophan decarboxylase").ti,ab. (4204)
75 (krabbe or "beta galactosidase deficiency syndrome" or "galactosylceramidase deficiency syndrome" or "galactosylceramide lipidosis" or "globoid cell dystrophy" or "globoid cell leucodystrophy" or "globoid cell leukodystrophy" or "mckusick 24520").ti,ab. (1625)
76 (rett or rett's).ti,ab. (5514)
77 14 or 15 or 16 or 17 or 18 or 19 or 20 or 21 or 22 or 23 or 24 or 25 or 26 or 27 or 28 or 29 or 30 or 31 or 32 or 33 or 34 or 35 or 36 or 37 or 38 or 39 or 40 or 41 or 42 or 43 or 44 or 45 or 46 or 47 or 48 or 49 or 50 or 51 or 52 or 53 or 54 or 55 or 56 or 57 or 58 or 59 or 60 or 61 or 62 or 63 or 64 or 65 or 66 or 67 or 68 or 69 or 70 or 71 or 72 or 73 or 74 or 75 or 76 (2541161)
78 exp pharmacovigilance/ (23261)
79 (Pharmacovigilan* or pharmaco-vigilan* or toxicit* or SUSAR or SAE or AR or SAR or "adverse reaction*" or "adverse event*" or antibodies or immunosuppress* or immunotherap* or "immune management" or immunomodulat* or "subclinical immune response*" or immunologic* or aminotransaminase* or ((liver or hepatic) adj2 (damag* or injur* or inflamm*)) or leukemia or leukaemia or cancer* or tumor* or tumour* or humoral or "cellular immunity" or "innate immunity" or immunogenicity or "complement activation" or cytokine* or "pre-existing immunity" or ADA or cell-mediated or binding-antibod* or "induced response*" or pre-existing or preexisting or boosted or "capsid response*" or redosing or "cell-mediated immun*" or "T-cell immunity" or tolerance or tolerization or TNF-alpha or prednisolone or eculizumab or "complement activation" or transaminitis or hypertransaminasemia or thrombocytopenia or microangiopathy).ti,ab. (8022126)
80 exp immunosuppressive treatment/ (260883)
81 exp leukemia/ (378133)
82 exp neoplasm/ (5678672)
83 exp liver injury/ (81741)
84 exp immunogenicity/ (89304)
85 exp cytokine/ (1961953)
86 prednisolone/ (153065)
87 eculizumab/ (9820)
88 exp thrombocytopenia/ (232160)
89 exp microangiopathy/ or hypertransaminasemia/ (79139)
90 spinal ganglion/ (26536)
91 exp hemolytic anemia/ (140088)
92 kidney injury/ or acute kidney failure/ (175604)
93 exp liver toxicity/ (103332)
94 (TMA or "dorsal root ganglion" or "dorsal root spinal ganglion" or "spinal ganglia" or "spine ganglion" or DRG or "hemolytic anemia" or "hemolytic anaemia" or "haemolytic anemia" or "haemolytic anaemia" or "acute kidney damage" or "acute kidney injury" or "acute kidney failure" or "acute renal damage" or "acute renal failure" or "elevated liver enzyme*" or "elevated hepatic enzyme*" or hepatotoxic* or "hepato toxic*" or "liver cell toxicit*" or "liver intoxication" or "liver poison*" or "liver toxic*" or "abnormal T2 hyperintensit*").ti,ab. (222334)
95 78 or 79 or 80 or 81 or 82 or 83 or 84 or 85 or 86 or 87 or 88 or 89 or 90 or 91 or 92 or 93

or 94 (10809812)
96 4 and 13 and 77 and 95 (5391)
97 case report/ (2972354)
98 observational study/ (361533)
99 ("case report*" or real-world or observational).ti,ab,kf. (1237582)
100 97 or 98 or 99 (3729719)
101 96 and 100 (433)

**Database: Medline (Ovid MEDLINE® Epub Ahead of Print, In-Process & Other Non-Indexed Citations, Ovid MEDLINE® Daily and Ovid MEDLINE®) 1946 to present**
Search Strategy:

--------------------------------------------------------------------------------

1 exp Genetic Therapy/ (54409)
2 ("DNA therap*" or (gene* adj2 therap*) or "gene* transfer*" or transgene or "gene replacement*" or "gene-edit*" or "gene silencing" or "gene repair*" or "gene correction*").ti,ab. (162791)
3 1 or 2 (181624)
4 Epidural Space/ (4886)
5 exp administration, intravenous/ or administration, ophthalmic/ (150896)
6 exp injections, intraocular/ or injections, intravenous/ or exp injections, spinal/ (110553)
7 (Intravenous* or intra-venous* or IV or systemic or infusion* or intramuscular* or intra-muscular* or IM or intrathecal* or intra-thecal* or "cerebrospinal fluid*" or CSF or intra-CSF or epidural* or spinal* or ophthalmol* or subretinal* or sub-retinal* or intravitreal* or intra-vitreal* or intraorbital* or intra-orbital* or intracerebroventricular* or intra-cerebroventricular* or ICV or "i.c.v. injection*" or ICVI or intracerebral* or intra-cerebral* or IC or intraventricular* or intra-ventricular* or intraparenchyma* or intra-parenchyma* or "intra-cisterna magna" or ICM).ti,ab. (2316440)
8 4 or 5 or 6 or 7 (2362480)
9 Rare Diseases/ (14380)
10 Orphan Drug Production/ (1465)
11 exp Muscular Atrophy, Spinal/ (6639)
12 exp Myopathies, Structural, Congenital/ (1624)
13 exp Muscular Dystrophies/ (30356)
14 exp Paraplegia/ (13603)
15 exp Gangliosidoses/ (2695)
16 exp Multiple Sclerosis/ (71739)
17 Parkinson Disease/ (84852)
18 Alzheimer Disease/ (123730)
19 Neuronal Ceroid-Lipofuscinoses/ (2273)
20 exp "Hereditary Sensory and Motor Neuropathy"/ (8261)
21 hemophilia a/ or hemophilia b/ (25211)
22 exp Anemia, Sickle Cell/ (26145)
23 Glycogen Storage Disease Type II/ (1995)
24 Canavan Disease/ (313)

25 Fabry Disease/ (4224)
26 exp Mucopolysaccharidoses/ (7094)
27 Hyperlipoproteinemia Type II/ (7796)
28 Cardiomyopathy, Dilated/ (17408)
29 Cystic Fibrosis/ (40582)
30 alpha 1-Antitrypsin Deficiency/ (3789)
31 exp Ciliary Motility Disorders/ (2699)
32 exp Macular Degeneration/ (31035)
33 Leber Congenital Amaurosis/ (586)
34 ("rare disease*" or "orphan drug*" or "monogenic disease*" or "monogenic disorder*" or "spinal muscular atrophy" or "spine muscle atrophy" or "myotubular myopathy" or "centronuclear myopathy" or "myotonic dystrophy" or "curschmann steinert disease" or "deleage disease" or "dystrophic myotonica" or "dystrophic myotonia" or "myotonia atrophica" or "myotonia dystrophica" or "myotonic atrophia" or "myotonic atrophy" or "myotonic dystrophia" or "myotonic muscular dystrophy" or "myotonic muscular dystrophy" or "proximal myotonic myopathy" or "steinert disease" or "steinert myopathy" or "muscular dystrophy" or "muscle dystrophy" or "muscle dystrophia" or "motor neuron* disease" or MND or "Spastic paraplegia" or "spastic paraparesis" or gangliosidosis or "ganglioside lipidosis" or gangliosidoses or "multiple sclerosis" or "chariot disease" or "disseminated sclerosis" or "insular sclerosis" or MS or Parkinson* or "paralysis agitans" or Alzheimer* or Alzeimer* or "diffuse cortical sclerosis" or Batten* or "juvenile neuronal ceroid lipofuscinosis" or "Giant axonal neuropathy" or GAN or GAN-1 or "Amyotrophic lateral sclerosis" or ALS or "lou gehrig*" or Charcot-Marie-Tooth or "hereditary motor sensory neuropathy" or "charcot-marie" or "dejerine sottas" or "familial spastic para*" or "hereditary spastic para*" or Struempell or strumpel* or SCID-X or Haemophili* or hemophili* or "sickle cell" or drepanocyt* or "haemoglobin ss" or "hemoglobin ss" or "sickle anaemia" or "sickle anemia" or Pompe or "glycogen storage disease type 2" or "cardiomuscular glycogenosis" or "diffuse glycogenosis" or "glycogen storage disease type ii" or (glycogenos* adj2 (ii or targeted gene repair)) or Canavan* or "AADC deficien*" or Fabry* or "alpha galactosidase deficiency syndrome" or Mucopolysaccharidos* or "familial hypercholesterol*" or "buerger gruetz" or "burger grutz" or "familial hyperbetalipoprotein*" or "amilial hypercholester*" or "dilated cardiomyopathy" or "congestic cardiomyopathy" or "congestive heart disease" or "congestive myocardiopathy" or "cystic fibrosis" or "cystic pancreas fibrosis" or "fibrocystic disease" or "Alpha-1 antitrypsin deficiency" or "alpha 1 proteinase inhibitor deficiency" or "alpha1 antitrypsin deficiency" or "antitrypsin alpha 1 deficiency" or "ciliary dyskinesia" or "ciliary immotility" or "ciliatry motility disorders" or ciliostasis or "macula* degeneration" or "macula* atrophy" or "macular dystrophy" or LCA or Leber).ti,ab. (1119488)
35 exp Retinal Dystrophies/ (10905)
36 exp Retinitis Pigmentosa/ (10845)
37 Color Vision Defects/ (4266)
38 Friedreich Ataxia/ (2824)
39 Leukodystrophy, Metachromatic/ (1330)
40 Adenylosuccinate Lyase/ (267)
41 Angelman Syndrome/ (1406)
42 Leukodystrophy, Globoid Cell/ (1091)

43 Rett Syndrome/ (3003)
44 (LCA* or SMA or DMD or XMTM or LGMD or CMT or PD or MPS or Ho-FH or DCM or CF or AATD or PCD).ti,ab. (340964)
45 ("retinal dystroph*" or "Retinitis Pigmentosa" or RP or "retinal pigment* dystroph*" or "tapeto retinal degeneration" or "tapetoretinal degeneration" or "tapetoretinal dystrophy").ti,ab. (49225)
46 (achromatognosia or achromatopia or achromatopsia or "colour blind*" or "color blind*" or "color agnosia" or "colour agnosia" or "scoterythrous vision" or "duchenne syndrome" or "duchenne type muscular dystrophy" or "duchenne muscular dystrophy" or "morbus duchenne" or "pseudo hypertrophic myopathic progressive muscular dystrophy").ti,ab. (13233)
47 ("backer muscular dystrophy" or "becker muscular dystrophy" or "dystrophic becker disease" or "becker dystrophy" or "becker type muscular dystrophy").ti,ab. (1795)
48 (sarcoglycanopath* or Friedreich* or "hereditary spinal ataxia" or "spinal hereditary ataxia" or "spinal heredoataxia").ti,ab. (3759)
49 ("cerebroside sulfatase deficiency syndrome" or "cerebroside sulfate storage disease" or "cerebroside sulphate storage disease" or "metachrom* leucodystrophy" or "metachrom* leukodystrophy" or "sulfatide lipidosis" or sulfatidosis or "sulphatide lipidosis").ti,ab. (1483)
50 ("adenylosuccinate lyase" or adenylosuccinase or "adenylosuccinate amp lyase" or "adenylsuccinate lyase" or "e.c. 4.3.2.2" or "succinyladenylate lyase").ti,ab. (346)
51 ("Angelman syndrome" or "happy puppet syndrome").ti,ab. (1726)
52 ("hereditary progressive spinal muscular atrophy" or "hereditary spinal progressive muscular atrophy" or "hoffmann werdnig" or "werdnig hoffman" or ((infantile or "type 1" or "type I") adj3 "musc* atrophy")).ti,ab. (680)
53 (Dubowitz or (("type 2" or "type II") adj3 "musc* atrophy")).ti,ab. (472)
54 (kugelberg* or ((benign or juvenile or "type 3" or "type III") adj3 "musc* atrophy")).ti,ab. (515)
55 ("limb girdle dystroph*" or "limb-girdle muscular dystroph*").ti,ab. (2360)
56 ("GM1 gangliosidosis" or "gangliosidosis G(M1)" or "gangliosidosis gm 1" or "gangliosidosis GM1" or "gm 1 gangliosidosis").ti,ab. (784)
57 ("Aromatic l-amino acid decarboxylase" or "3,4 dihydroxy l phenylalanine carboxylyase" or "3,4 dihydroxyphenylalanine decarboxylase" or "5 hydroxy levo tryptophan carboxylyase" or "5 hydroxytrypthophan decarboxylase" or "5 hydroxytryptophan carboxylyase" or "5 hydroxytryptophan decarboxylase" or "5 hydroxytryptophane decarboxylase" or "aromatic amino acid decarboxylase*" or "aromatic aminoacid decarboxylase*" or "aromatic l amino acid carboxy lyase" or "aromatic l amino acid decarboxylase*" or "aromatic l aminoacid decarboxylase" or "aromatic levo aminoacid decarboxylase" or "dihydroxyphenylalanine decarboxylase" or "DOPA decarboxylase" or "e.c. 4.1.1.28" or "hydroxytryptophan decarboxylase" or "l 3,4 dihydroxyphenylalanine decarboxylase" or "l dopa decarboxylase" or "l tryptophan carboxylyase" or "levo dopa decarboxylase" or "levodopa decarboxylase" or "tryptophan decarboxylase").ti,ab. (3606)
58 (krabbe or "beta galactosidase deficiency syndrome" or "galactosylceramidase deficiency syndrome" or "galactosylceramide lipidosis" or "globoid cell dystrophy" or "globoid cell leucodystrophy" or "globoid cell leukodystrophy" or "mckusick 24520").ti,ab. (1224)
59 (rett or rett's).ti,ab. (4265)
60 9 or 10 or 11 or 12 or 13 or 14 or 15 or 16 or 17 or 18 or 19 or 20 or 21 or 22 or 23 or 24

or 25 or 26 or 27 or 28 or 29 or 30 or 31 or 32 or 33 or 34 or 35 or 36 or 37 or 38 or 39 or 40 or 41 or 42 or 43 or 44 or 45 or 46 or 47 or 48 or 49 or 50 or 51 or 52 or 53 or 54 or 55 or 56 or 57 or 58 or 59 (1506235)
61 pharmacovigilance/ (3515)
62 (Pharmacovigilan* or pharmaco-vigilan* or toxicit* or SUSAR or SAE or AR or SAR or "adverse reaction*" or "adverse event*" or antibodies or immunosuppress* or immunotherap* or "immune management" or immunomodulat* or "subclinical immune response*" or immunologic* or aminotransaminase* or ((liver or hepatic) adj2 (damag* or injur* or inflamm*)) or leukemia or leukaemia or cancer* or tumor* or tumour* or humoral or "cellular immunity" or "innate immunity" or immunogenicity or "complement activation" or cytokine* or "pre-existing immunity" or ADA or cell-mediated or binding-antibod* or "induced response*" or pre-existing or preexisting or boosted or "capsid response*" or redosing or "cell-mediated immun*" or "T-cell immunity" or tolerance or tolerization or TNF-alpha or prednisolone or eculizumab or "complement activation" or transaminitis or hypertransaminasemia or thrombocytopenia or microangiopathy).ti,ab. (5956431)
63 exp Immunosuppressive Agents/ (348675)
64 exp Leukemia/ (257251)
65 exp Neoplasms/ (3941650)
66 exp "Chemical and Drug Induced Liver Injury"/ (34033)
67 exp Immunogenetic Phenomena/ (49969)
68 exp Cytokines/ (805065)
69 exp Prednisolone/ (54238)
70 exp Thrombocytopenia/ (54496)
71 Ganglia, Spinal/ (20340)
72 exp Anemia, Hemolytic/ (82573)
73 exp Acute Kidney Injury/ (57686)
74 (TMA or "dorsal root ganglion" or "dorsal root spinal ganglion" or "spinal ganglia" or "spine ganglion" or DRG or "hemolytic anemia" or "hemolytic anaemia" or "haemolytic anemia" or "haemolytic anaemia" or "acute kidney damage" or "acute kidney injury" or "acute kidney failure" or "acute renal damage" or "acute renal failure" or "elevated liver enzyme*" or "elevated hepatic enzyme*" or hepatotoxic* or "hepato toxic*" or "liver cell toxicit*" or "liver intoxication" or "liver poison*" or "liver toxic*" or "abnormal T2 hyperintensit*").ti,ab. (148467)
75 61 or 62 or 63 or 64 or 65 or 66 or 67 or 68 or 69 or 70 or 71 or 72 or 73 or 74 (7851538)
76 3 and 8 and 60 and 75 (1510)
77 case reports/ (2388500)
78 Observational Study/ (152822)
79 ("case report*" or real-world or observational).ti,ab,kf. (885009)
80 77 or 78 or 79 (2902175)
81 76 and 80 (44)

**Cochrane Central Register of Controlled Trials**
**Issue 2 of 12, February 2024**

#1 MeSH descriptor: [Genetic Therapy] explode all trees 358

#2 ((DNA NEXT therap*) or (gene* near/2 therap*) or (gene* NEXT transfer*) or transgene or (gene NEXT replacement*) or (gene* next edit*) or "gene silencing" or (gene NEXT repair*) or (gene NEXT correction*)):ti,ab,kw 18478
#3 #1 OR #2 18491
#4 MeSH descriptor: [Infusion Pumps] explode all trees 1637
#5 MeSH descriptor: [Epidural Space] explode all trees 294
#6 MeSH descriptor: [Administration, Intravenous] explode all trees 22798
#7 MeSH descriptor: [Administration, Ophthalmic] explode all trees 180
#8 MeSH descriptor: [Injections, Intraocular] explode all trees 1494
#9 MeSH descriptor: [Injections, Intravenous] explode all trees 9025
#10 MeSH descriptor: [Injections, Spinal] explode all trees 1823
#11 (Intravenous* or intra-venous* or IV or systemic or infusion* or intramuscular* or intra-muscular* or IM or intrathecal* or (intra NEXT thecal*) or (cerebrospinal NEXT fluid*) or CSF or intra-CSF or epidural* or spinal* or ophthalmol* or subretinal* or sub-retinal* or intravitreal* or intra-vitreal* or intraorbital* or intra-orbital* or intracerebroventricular* or intra-cerebroventricular* or ICV or (i.c.v. NEXT injection*) or ICVI or intracerebral* or intra-cerebral* or IC or intraventricular* or (intra NEXT ventricular*) or intraparenchyma* or (intra NEXT parenchyma*) or "intra-cisterna magna" or ICM):ti,ab,kw 338369
#12 #4 or #5 or #6 or #7 or #8 or #9 or #10 or #11 338571
#13 MeSH descriptor: [Rare Diseases] explode all trees 77
#14 MeSH descriptor: [Orphan Drug Production] explode all trees 19
#15 MeSH descriptor: [Muscular Atrophy, Spinal] explode all trees 148
#16 MeSH descriptor: [Myopathies, Structural, Congenital] explode all trees 6
#17 MeSH descriptor: [Muscular Dystrophies] explode all trees 659
#18 MeSH descriptor: [Paraplegia] explode all trees 279
#19 MeSH descriptor: [Gangliosidoses] explode all trees 11
#20 MeSH descriptor: [Multiple Sclerosis] explode all trees 5178
#21 MeSH descriptor: [Parkinson Disease] explode all trees 6009
#22 MeSH descriptor: [Alzheimer Disease] explode all trees 5232
#23 MeSH descriptor: [Neuronal Ceroid-Lipofuscinoses] explode all trees 11
#24 MeSH descriptor: [Hereditary Sensory and Motor Neuropathy] explode all trees 119
#25 MeSH descriptor: [Hemophilia A] explode all trees 641
#26 MeSH descriptor: [Anemia, Sickle Cell] explode all trees 1061
#27 MeSH descriptor: [Glycogen Storage Disease Type II] explode all trees 56
#28 MeSH descriptor: [Canavan Disease] explode all trees 3
#29 MeSH descriptor: [Fabry Disease] explode all trees 110
#30 MeSH descriptor: [Mucopolysaccharidoses] explode all trees 106
#31 MeSH descriptor: [Hyperlipoproteinemia Type II] explode all trees 664
#32 MeSH descriptor: [Cardiomyopathy, Dilated] explode all trees 669
#33 MeSH descriptor: [Cystic Fibrosis] explode all trees 2368
#34 MeSH descriptor: [alpha 1-Antitrypsin Deficiency] explode all trees 120
#35 MeSH descriptor: [Ciliary Motility Disorders] explode all trees 32
#36 MeSH descriptor: [Macular Degeneration] explode all trees 3542
#37 MeSH descriptor: [Leber Congenital Amaurosis] explode all trees 14
#38 ((rare NEXT disease*) or (orphan NEXT drug*) or (monogenic NEXT disease*) or (monogenic NEXT disorder*) or "spinal muscular atrophy" or "spine muscle atrophy" or "myotubular myopathy" or "centronuclear myopathy" or "myotonic dystrophy" or "curschmann steinert disease" or "deleage disease" or "dystrophic myotonica" or "dystrophic myotonia" or "myotonia atrophica" or "myotonia dystrophica" or "myotonic atrophia" or

"myotonic atrophy" or "myotonic dystrophia" or "myotonic muscular dystrophy" or "myotonic muscular dystrophy" or "proximal myotonic myopathy" or "steinert disease" or "steinert myopathy" or "muscular dystrophy" or "muscle dystrophy" or "muscle dystrophia" or (neuron* NEXT disease) or MND or "Spastic paraplegia" or "spastic paraparesis" or gangliosidosis or "ganglioside lipidosis" or gangliosidoses or "multiple sclerosis" or "chariot disease" or "disseminated sclerosis" or "insular sclerosis" or MS or Parkinson* or "paralysis agitans" or Alzheimer* or Alzeimer* or "diffuse cortical sclerosis" or Batten* or "juvenile neuronal ceroid lipofuscinosis" or "Giant axonal neuropathy" or GAN or GAN-1 or "Amyotrophic lateral sclerosis" or ALS or (lou NEXT gehrig*) or Charcot-Marie-Tooth or "hereditary motor sensory neuropathy" or charcot-marie or "dejerine sottas" or (spastic NEXT para*) or Struempell or strumpel* or SCID-X or Haemophili* or hemophili* or "sickle cell" or drepanocyt* or "haemoglobin ss" or "hemoglobin ss" or "sickle anaemia" or "sickle anemia" or Pompe or "glycogen storage disease type 2" or "cardiomuscular glycogenosis" or "diffuse glycogenosis" or "glycogen storage disease type ii" or (glycogenos* NEAR/2 (ii or targeted gene repair)) or Canavan* or (AADC NEXT deficien*) or Fabry* or "alpha galactosidase deficiency syndrome" or Mucopolysaccharidos* or (familial NEXT hypercholesterol*) or "buerger gruetz" or "burger grutz" or (familial NEXT hyperbetalipoprotein*) or (amilial NEXT hypercholester*) or "dilated cardiomyopathy" or "congestic cardiomyopathy" or "congestive heart disease" or "congestive myocardiopathy" or "cystic fibrosis" or "cystic pancreas fibrosis" or "fibrocystic disease" or "Alpha-1 antitrypsin deficiency" or "alpha 1 proteinase inhibitor deficiency" or "alpha1 antitrypsin deficiency" or "antitrypsin alpha 1 deficiency" or "ciliary dyskinesia" or "ciliary immotility" or "ciliatry motility disorders" or ciliostasis or (macula* NEXT degeneration) or (macula* NEXT atrophy) or "macular dystrophy" or LCA or Leber):ti,ab,kw 79421

#39 MeSH descriptor: [Retinal Dystrophies] explode all trees 164

#40 MeSH descriptor: [Retinitis Pigmentosa] explode all trees 157

#41 MeSH descriptor: [Color Vision Defects] explode all trees 50

#42 MeSH descriptor: [Friedreich Ataxia] explode all trees 98

#43 MeSH descriptor: [Leukodystrophy, Metachromatic] explode all trees 6

#44 MeSH descriptor: [Adenylosuccinate Lyase] explode all trees 0

#45 MeSH descriptor: [Angelman Syndrome] explode all trees 27

#46 MeSH descriptor: [Leukodystrophy, Globoid Cell] explode all trees 2

#47 MeSH descriptor: [Rett Syndrome] explode all trees 64

#48 (LCA* or SMA or DMD or XMTM or LGMD or CMT or PD or MPS or Ho-FH or DCM or CF or AATD or PCD):ti,ab,kw 54647

#49 ((retinal NEXT dystroph*) or "Retinitis Pigmentosa" or RP or (pigment* NEXT dystroph*) or "tapeto retinal degeneration" or "tapetoretinal degeneration" or "tapetoretinal dystrophy"):ti,ab,kw 3262

#50 (achromatognosia or achromatopia or achromatopsia or (colour NEXT blind*) or (color NEXT blind*) or "color agnosia" or "colour agnosia" or "scoterythrous vision" or "duchenne syndrome" or "duchenne type muscular dystrophy" or "duchenne muscular dystrophy" or "morbus duchenne" or "pseudo hypertrophic myopathic progressive muscular dystrophy"):ti,ab,kw 914

#51 ("backer muscular dystrophy" or "becker muscular dystrophy" or "dystrophic becker disease" or "becker dystrophy" or "becker type muscular dystrophy"):ti,ab,kw 84

#52 (sarcoglycanopath* or Friedreich* or "hereditary spinal ataxia" or "spinal hereditary ataxia" or "spinal heredoataxia"):ti,ab,kw 177

#53 ("cerebroside sulfatase deficiency syndrome" or "cerebroside sulfate storage disease" or "cerebroside sulphate storage disease" or (metachrom* NEXT leucodystrophy) or

(metachrom* NEXT leukodystrophy) or "sulfatide lipidosis" or sulfatidosis or "sulphatide lipidosis"):ti,ab,kw 9
#54 ("adenylosuccinate lyase" or adenylosuccinase or "adenylosuccinate amp lyase" or "adenylsuccinate lyase" or "e.c. 4.3.2.2" or "succinyladenylate lyase"):ti,ab,kw 1
#55 ("Angelman syndrome" or "happy puppet syndrome"):ti,ab,kw 42
#56 ("hereditary progressive spinal muscular atrophy" or "hereditary spinal progressive muscular atrophy" or "hoffmann werdnig" or "werdnig hoffman" or ((infantile or "type 1" or "type I") near/4 atrophy)):ti,ab,kw 33
#57 (Dubowitz or (("type 2" or "type II") near/4 atrophy)):ti,ab,kw 78
#58 (kugelberg* or ((benign or juvenile or "type 3" or "type III") near/4 atrophy)):ti,ab,kw 74
#59 ((girdle NEXT dystroph*) or ("limb-girdle muscular" NEXT dystroph*)):ti,ab,kw 41
#60 ("GM1 gangliosidosis" or "gangliosidosis G(M1)" or "gangliosidosis gm 1" or "gangliosidosis GM1" or "gm 1 gangliosidosis"):ti,ab,kw 7
#61 ("Aromatic l-amino acid decarboxylase" or "3,4 dihydroxy l phenylalanine carboxylyase" or "3,4 dihydroxyphenylalanine decarboxylase" or "5 hydroxy levo tryptophan carboxylyase" or "5 hydroxytrypthophan decarboxylase" or "5 hydroxytryptophan carboxylyase" or "5 hydroxytryptophan decarboxylase" or "5 hydroxytryptophane decarboxylase" or ("aromatic amino acid" NEXT decarboxylase*) or ("aromatic aminoacid" NEXT decarboxylase*) or "aromatic l amino acid carboxy lyase" or ("aromatic l amino acid" NEXT decarboxylase*) or "aromatic l aminoacid decarboxylase" or "aromatic levo aminoacid decarboxylase" or "dihydroxyphenylalanine decarboxylase" or "DOPA decarboxylase" or "e.c. 4.1.1.28" or "hydroxytryptophan decarboxylase" or "l 3,4 dihydroxyphenylalanine decarboxylase" or "l dopa decarboxylase" or "l tryptophan carboxylyase" or "levo dopa decarboxylase" or "levodopa decarboxylase" or "tryptophan decarboxylase"):ti,ab,kw 241
#62 (krabbe or "beta galactosidase deficiency syndrome" or "galactosylceramidase deficiency syndrome" or "galactosylceramide lipidosis" or "globoid cell dystrophy" or "globoid cell leucodystrophy" or "globoid cell leukodystrophy" or "mckusick 24520"):ti,ab,kw 8
#63 (rett or rett's):ti,ab,kw 121
#64 #13 or #14 or #15 or #16 or #17 or #18 or #19 or #20 or #21 or #22 or #23 or #24 or #25 or #26 or #27 or #28 or #29 or #30 or #31 or #32 or #33 or #34 or #35 or #36 or #37 or #38 or #39 or #40 or #41 or #42 or #43 or #44 or #45 or #46 or #47 or #48 or #49 or #50 or #51 or #52 or #53 or #54 or #55 or #56 or #57 or #58 or #59 or #60 or #61 or #62 or #63 125949
#65 MeSH descriptor: [Pharmacovigilance] explode all trees 42
#66 (Pharmacovigilan* or pharmaco-vigilan* or toxicit* or SUSAR or SAE or AR or SAR or (adverse NEXT reaction*) or (adverse NEXT event*) or antibodies or immunosuppress* or immunotherap* or "immune management" or immunomodulat* or ("subclinical immune" NEXT response*) or immunologic* or aminotransaminase* or ((liver or hepatic) NEAR/2 (damag* or injur* or inflamm*)) or leukemia or leukaemia or cancer* or tumor* or tumour* or humoral or "cellular immunity" or "innate immunity" or immunogenicity or "complement activation" or cytokine* or "pre-existing immunity" or ADA or cell-mediated or (binding NEXT antibod*) or (induced NEXT response*) or pre-existing or preexisting or boosted or (capsid NEXT response*) or redosing or (cell-mediated NEXT immun*) or "T-cell immunity" or tolerance or tolerization or TNF-alpha or prednisolone or eculizumab or "complement activation" or transaminitis or hypertransaminasemia or thrombocytopenia or microangiopathy):ti,ab,kw 526912
#67 MeSH descriptor: [Immunosuppressive Agents] explode all trees 6937

#68 MeSH descriptor: [Leukemia] explode all trees 6529
#69 MeSH descriptor: [Neoplasms] explode all trees 123839
#70 MeSH descriptor: [Chemical and Drug Induced Liver Injury] explode all trees 474
#71 MeSH descriptor: [Immunogenetic Phenomena] explode all trees 135
#72 MeSH descriptor: [Cytokines] explode all trees 27506
#73 MeSH descriptor: [Prednisolone] explode all trees 5996
#74 MeSH descriptor: [Thrombocytopenia] explode all trees 1790
#75 MeSH descriptor: [Ganglia, Spinal] explode all trees 71
#76 MeSH descriptor: [Anemia, Hemolytic] explode all trees 1840
#77 MeSH descriptor: [Acute Kidney Injury] explode all trees 2347
#78 (TMA or "dorsal root ganglion" or "dorsal root spinal ganglion" or "spinal ganglia" or "spine ganglion" or DRG or "hemolytic anemia" or "hemolytic anaemia" or "haemolytic anemia" or "haemolytic anaemia" or "acute kidney damage" or "acute kidney injury" or "acute kidney failure" or "acute renal damage" or "acute renal failure" or ("elevated liver" NEXT enzyme*) or ("elevated hepatic" NEXT enzyme*) or hepatotoxic* or (hepato NEXT toxic*) or ("liver cell" NEXT toxicit*) or "liver intoxication" or (liver NEXT poison*) or (liver NEXT toxic*) or ("abnormal T2" NEXT hyperintensit*)):ti,ab,kw 11367
#79 #65 or #66 or #67 or #68 or #69 or #70 or #71 or #72 or #73 or #74 or #75 or #76 or #77 or #78 560567
#80 #3 and #12 and #64 and #79 732
#81 (realworld* OR (real NEXT world*)):ti,ab,kw 8856
#82 (observational OR (case NEXT report*)):ti,ab,kw 37146
#83 #81 or #82 44896
#84 #80 and #83 32
